# Functional genetics-directed identification of novel pharmacological inhibitors of FAS- and TNF-dependent apoptosis that protect mice from acute liver failure

**DOI:** 10.1038/cddis.2016.45

**Published:** 2016-03-17

**Authors:** A P Komarov, E A Komarova, K Green, L R Novototskaya, P S Baker, A Eroshkin, A L Osterman, A A Chenchick, C Frangou, A V Gudkov

**Affiliations:** 1 Cellecta, Inc., Mountain View, CA, USA; 2 Department of Cell Stress Biology, Roswell Park Cancer Institute, Buffalo, NY, USA; 3 Buffalo BioLabs, LLC, Buffalo, NY, USA; 4 Infectious and Inflammatory Disease Center, Sanford-Burnham-Prebys Medical Discovery Institute, La Jolla, CA, USA

## Abstract

shRNA-mediated gene-silencing technology paired with cell-based functional readouts reveals potential targets directly, providing an opportunity to identify drugs against the target without knowing the precise role of the target in the pathophysiological processes of interest. By screening a lentiviral shRNA library targeting for major components of human signaling pathways and known drug targets, we identified and validated both canonical as well as 52 novel mediators of FAS and TNF ligand-induced apoptosis. Presence of potential therapeutic targets among these mediators was confirmed by demonstration of *in vivo* activity of siRNAs against four identified target candidates that protected mice from acute liver failure (ALF), a life-threatening disease with known involvement of death receptor (DR)-mediated apoptosis. Network-based modeling was used to predict small-molecule inhibitors for several candidate apoptosis mediators, including somatostatin receptor 5 (SSTR5) and a regulatory subunit of PP2A phosphatase, PPP2R5A. Remarkably, pharmacological inhibition of either SSTR5 or PPP2R5A reduced apoptosis induced by either FASL or TNF in cultured cells and dramatically improved survival in several mouse models of ALF. These results demonstrate the utility of loss-of-function genetic screens and network-based drug-repositioning methods for expedited identification of targeted drug candidates and revealed pharmacological agents potentially suitable for treatment of DR-mediated pathologies.

Identification of targets and drugs are usually disconnected processes, with the search for drugs beginning only after extensive validation of targets and investigation of the mechanisms underlying their ‘druggability'. We hypothesized that functional genomics-based target discovery technologies combined with availability of databases containing numerous pharmacological agents with known targets but no current utility can enable one to greatly expedite this process. To test this idea, we used as a model a death receptor (DR) -mediated pathology to search for effective drug candidates among pharmacological modulators of products of gene essential for FAS- and TNF-mediated apoptosis and identified via functional screening of shRNA library.

In addition to its established role in autoimmunity and tumor surveillance,^1, 2^ the prototypic DR FAS (also called CD95 or APO-1) has an important role in the pathogenesis of numerous diseases.^3, 4, 5, 6^ Particularly in the liver, high expression of FAS has been implicated in the pathogenesis of viral hepatitis, inflammatory hepatitis, Wilson's disease, alcoholic liver disease, and chemotherapy-induced liver damage.^7, 8, 9^ FAS-mediated apoptosis also occurs in transplantation-associated liver damage: ischemia/re-perfusion injury and graft rejection.^5, 10, 11^ The devastating effect of FAS activation in the liver is illustrated by the biological effect of FAS ligand (FASL) or agonistic anti-FAS antibodies (Ab). Injection of either agent into mice leads to massive apoptosis of hepatocytes followed by acute liver failure (ALF) and animal death.^12^

Another DR ligand, TNF, also has an important role in liver pathology. Treatment of mice with TNF in combination with a global inhibitor of transcription such as d-galactosamine or actinomycin D induces lethal hepatitis.^13^ Another well-established mouse model of ALF consists of combined treatment with d-galactosamine and bacterial lipopolysaccharide (LPS), both inducing TNF expression and an acute inflammatory response that is predominantly directed toward the liver.^14^

Several recent studies have reported that hepatocyte-specific delivery of small interfering RNAs (siRNAs) targeting FAS or caspase-8 in mice provided protection against FAS-mediated ALF and reduced the severity of liver fibrosis in a model of concanavalin A (ConA)-induced hepatitis.^15, 16, 17^ Although these strategies for prevention of liver damage are not likely to progress to the clinic because of problems associated with delivery, stability and off-target gene-silencing of siRNAs, they provide strong rationale for further investigation into targeting apoptosis for treatment of ALF.

Beyond its potential as a treatment modality, RNAi is a useful tool for identifying and validating new therapeutic targets. In this study, we established an RNAi screening strategy to systematically identify genetic modifiers of FAS- and TNF-mediated apoptosis for potential use as therapeutic targets in treatment of pathologies associated with the activation of DR-mediated apoptosis. Using this approach, we identified both canonical components and novel factors that, upon RNAi-mediated knockdown, suppress FAS- and/or TNF-mediated apoptosis *in vitro*. Four of these genes were further validated *in vivo* through demonstration that siRNA-mediated reduction of their expression blocked FAS agonistic Ab-induced mouse death from ALF. Computational prediction of drug–target interactions using network-driven shRNA data prioritization and integration allowed us to ‘reposition' existing pharmacological agents for inhibition of two candidate targets, SSTR5 and PPP2R5A. These agents completely abrogated otherwise lethal liver failure induced by FAS agonistic Ab or ConA administration in mice thus demonstrating their potential for prevention or treatment of ALF and other conditions associated with DR-mediated apoptosis known to be involved in pathogenesis of neuronal,^18^ cardiac,^19^ pulmonary,^20^ renal ^21^ and other diseases.^22, 23^

## Results

### Pathway Decipher: a novel shRNA library resource for identification of potential therapeutic targets

**Figure 1:**
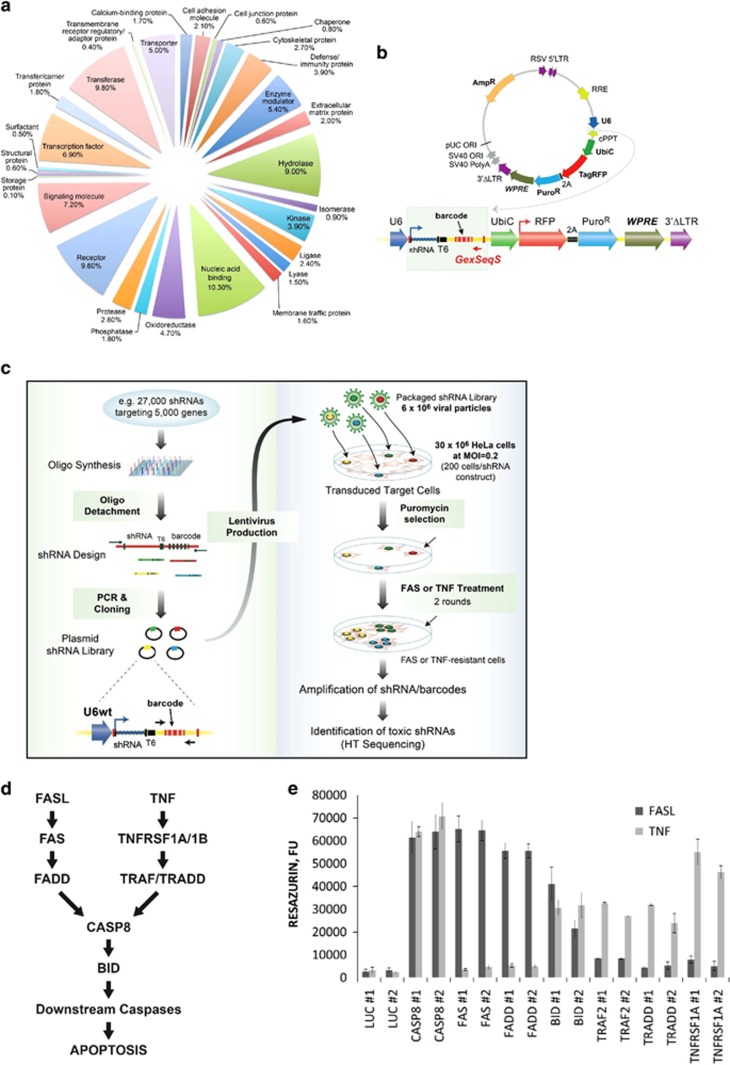
RNAi-based phenotypic screening approach used to identify modulators of FAS- and TNF-induced apoptosis. (**a**) Pathway Decipher genes (*n*=5046) separated into annotated functional groups, with the sizes of different pie segments indicating the relative frequency of genes within the corresponding groups. (**b**) Lentiviral expression vector used for construction of the 27 K pooled shRNA Library targeting Pathway Decipher genes. (**c**) Construction of the 27 K Pathway Decipher shRNA Library and scheme of shRNA library screening for inhibition of FASL- or TNF-induced apoptosis. (**d**) Canonical FAS and TNF receptor-mediated apoptotic pathways. (**e**) Effects of shRNAs targeting genes for canonical FAS and TNF pathway members on viability of cells treated with FASL or TNF. shRNAs (two per target gene) were cloned into the lentiviral expression vector shown in **b** in an arrayed library format and transduced into individual HeLa cell populations. After puromycin selection, transduced cells were exposed to FASL (50 ng/ml, dark gray bars) or TNF (50 ng/ml, light gray bars) in the presence of cycloheximide (2.5 *μ*g/ml). Cell viability was measured 24 h later by Resazurin assay, with fluorescence units quantitatively correlated with cell viability. shRNAs against luciferase (LUC) were used as negative controls. The bars indicate relative survival of cells (%) versus untreated control cells

To systematically probe key molecules involved in ALF, we built a focused shRNA library (herein denoted Pathway Decipher) targeting 5046 rationally selected human genes (~24% of human protein-encoding genes). Pathway Decipher contains genes that belong to 402 manually curated pathways and ~200 canonical pathways, as well as those with associations to 2669 discrete disease processes and 943 that encode known drug targets (Figure 1a and Supplementary Table 1). The Pathway Decipher library consists of 27 000 bar-coded shRNAs targeting the 5046 genes described above, with a minimum of five unique shRNAs per gene. The shRNAs were generated using on-chip oligonucleotide synthesis and inserted into a lentiviral vector optimized for strong shRNA expression, viral titer, and overall vector stability/function (Figure 1b). In this vector, the RNA polymerase III promoter of the human U6 small nuclear RNA gene (U6) drives high levels of shRNA expression, while the human Ubiquitin C core promoter provides constitutive expression of both the gene encoding mCherry fluorescent protein and the puromycin resistance (PuroR) gene translated as a fused polypeptide through a self-processing porcine Teschovirus (pTV1) 2A-like cleavage peptide. The vector also provides external DNA ‘barcodes' located downstream of the shRNA cassette for quantitation of the relative abundance of individual clones via deep barcode sequencing (Figure 1b).

### An RNAi screen for genes encoding mediators of FAS- and/or TNF-dependent apoptosis

To expediently identify mediators of FAS- and/or TNF-dependent apoptosis, we used HeLa cells which are known to be susceptible to apoptosis induced by human anti-FAS Ab or DR-ligand(s) (i.e., FASL or TNF) and designed a pooled RNAi loss-of-function screening approach based on direct phenotypic selection of viable cells (Figure 1c). The feasibility of this approach was confirmed in pilot studies showing that shRNAs targeting known modulators of FAS and/or TNF canonical signaling pathways (e.g., CASP8, BID, FAS, TRAF2, TRADD and so on) strongly inhibited FASL- and TNF-induced HeLa cell death as expected (Figures 1d and e).

For the screen, three batches of HeLa cells were independently infected with the pooled Pathway Decipher shRNA library under conditions enabling at least 200 independent integrations per individual shRNA with the majority of transduced cells carrying a single provirus and expressing a single shRNA insert. Non-transduced HeLa cells were eliminated by puromycin selection. Each of the three batches of library-transduced HeLa cells was divided equally into three samples: untreated control (collected immediately after completion of puromycin selection), anti-FAS Ab-treated and TNF-treated. Conditions of treatment with death ligands included the use of translation inhibitor cycloheximide what helped to strongly reduce the background of surviving cells presumably due to the inhibition of a pro-survival activity of NF-*κ*B. The representation of different shRNAs in each sample of HeLa cells was determined through nested-PCR amplification and deconvolution by high-throughput sequencing of the genetic barcodes uniquely identifying each individual library-derived shRNA. The relative representation of clones was highly reproducible in three experimental replicas (*R*^2^=0.96, *P*<0.0001) (Supplementary Fig. 1).

**Figure 2:**
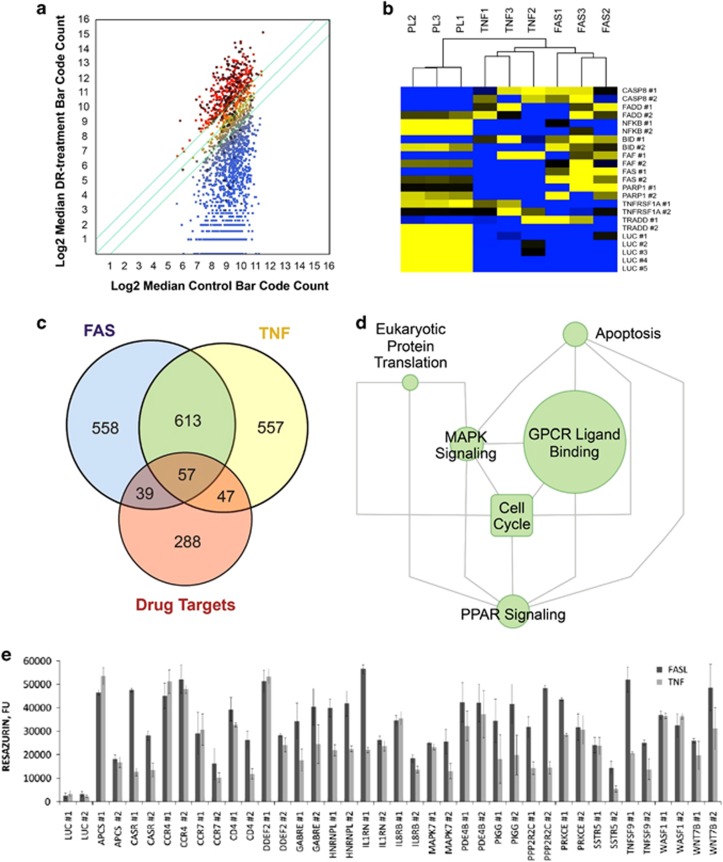
Identification of hit genes from primary screens with the 27 K Pathway Decipher shRNA library. (**a**) Scatter plot of gene targets statistically over-represented in anti-FAS Ab- and/or TNF-treated cells (three replicates each; *y*-axis) relative to median control value (DR-untreated replicates, *n*=3; *x*-axis) at the end of the culture period from primary screens. Data is visualized in the scatter plot on a log_2_ scale. The middle diagonal line is the identity line, or the *x*=*y* line. The other two lines delineate genes with at least a twofold change in the intensity value in one of the data sets. Enriched genes (>2-fold, *P*<0.05) are indicated in red, and those that were enriched in *both* FAS and TNF screens are highlighted in black. To detect hits in the primary screens, *P*-values for each shRNA were calculated using a modified Rank-product test and by comparing the phenotypes produced by shRNAs targeting each gene with the phenotypes produced by negative-control shRNAs. (**b**) Pseudo-color representation of screen results. Apoptosis-related genes were used to evaluate assay performance. Internal library shRNA controls targeting genes known to be involved in DR-mediated apoptosis (positive controls) or the luciferase gene (negative controls) were used for data normalization. FAS, FADD, CASP8 and BID shRNAs showed the expected inhibition of FASL-induced apoptosis. (**c**) Venn diagram summarizing the number of genes statistically over-represented in FASL- and TNF-induced apoptosis shRNA screens and their relation to each other as well as known drug targets. (**d**) Graphical depiction of enriched pathway modules obtained by edge-betweeness network clustering for the altered genes from FAS and TNF shRNA screens FI networks. Each node is a pathway and nodes are linked by directed edges representing parent-to-child relationships. Node size is proportional to the number of proteins annotated within that term. (**e**) *In vitro* validation of genes identified as novel candidate apoptosis modulators using transduction of individual shRNAs into HeLa cells followed by FASL or TNF treatment. For validation experiments, we generated 200 unique lentiviral constructs containing 200 different shRNAS corresponding to 100 candidate genes selected in the primary screen (two shRNAs per gene) (Supplementary Figure 2). These were tested as an arrayed shRNA sub-library; representative shRNA hits are shown on the graph. shRNAs were confirmed as hits if they increased survival of FASL- or TNF-treated cells by at least twofold relative to controls expressing shRNA targeting luciferase (LUC). shRNAs against canonical members of the FAS and TNF pathways isolated in the screen served as positive controls in this assay. The bars indicate relative survival of cells (%) *versus* untreated control cells

Next, we created a list of shRNAs that appeared to be candidate suppressors of FAS- or TNF-dependent cell death because their relative abundance was increased in cell populations that survived DR-stimulation (Figure 2a and Supplementary Table 2). Internal library shRNA controls targeting genes known to be involved in DR-mediated apoptosis (positive controls) or the luciferase gene (negative controls) were used for data normalization (Figure 2b). As summarized in Figure 2c, comparisons of differential shRNA representation (>2-fold change, with *P*<0.001) in FASL- or TNF-treated cells revealed a subset of 1871 shRNAs that became over-represented following selection. We assessed whether any enriched biological themes (particularly GO terms and pathways) were statistically over- or under-represented in the pool of shRNAs recovered from DR-treated cells compared with control cells and visualized hits on curated pathway gene maps (Supplementary Methods).^24^ Enriched functionally related gene groups and pathways (FDR<0.001) included FAS and TNF apoptosis pathways as expected, and ERK, MAPK and chemokine signaling pathways (Supplementary Tables 3-6).

According to the estimates of shRNA library manufacturer (Cellecta Inc.), up to 20% of all hits may target mRNAs of one or two additional genes leading to false-positive hits. To reduce probability of picking such hits during our screening we applied the following criteria for selecting target gene candidates: (i) an enrichment ratio of >2 in FAS- or TNF-treated cells *versus* untreated control cells; (ii) ⩾2 independent shRNA clones for a given gene target with the desired enrichment ratio; and (iii) reproducibility of both prior conditions in all three experimental replicas. Among 433 genes that met our cutoff criteria we were pleased to find all major components of conventional FAS and TNF signaling pathways (see below).

To prioritize candidate gene hits, we first examined whether they could be parsed into distinct pathways and/or complexes based on functional interactions. Briefly, two functional interaction (FI) networks were independently generated from the obtained FAS and TNF RNAi data sets and network subtraction allowed us to identify both overlapping and unique gene clusters. As summarized in Figure 2d, network comparison identified multiple overlapping FI sub-networks that may represent key mediators of DR-mediated apoptosis (Supplementary Table 7). To prioritize/rank candidate genes associated with DR-mediated apoptosis, we used a feed-forward-based network approach to identify key upstream and downstream signaling nodes in the network vicinity of our candidate genes.^25^ Key upstream signaling nodes mapped to FAS, TNF, IL-1, cAMP, cGMP and MAPK signal transduction pathways (Supplementary Table 8).

To confirm the validity of our gene prioritization approach, we individually tested the effects of shRNAs targeting a subset of putative pro-apoptotic genes identified in our screen and assigned to several network sub-clusters identified above (Figure 2d). To accurately assess assay performance, shRNAs targeting luciferase (LUC) and seven canonical FAS/TNF pathway components were used as negative and positive controls, respectively (Figure 2e and Supplementary Fig. 2). In total, 102 shRNAs (i.e., shRNAs targeting 52% of the tested candidate genes) produced an enrichment ratio of >4 in FAS- or TNF-treated cells compared with negative-control shLUC-transduced cells after 7 days of outgrowth (*P*-value of <0.00005; Figure 2e and Supplementary Fig. 2). These results suggest that a significant proportion of genes identified in our primary screens are likely to have roles in DR-signaling pathways and may, therefore, be potential targets for therapeutic modulation of apoptosis.

### *In vivo* validation of candidate genes as potential targets for pharmacological protection against FAS-induced ALF

**Figure 3:**
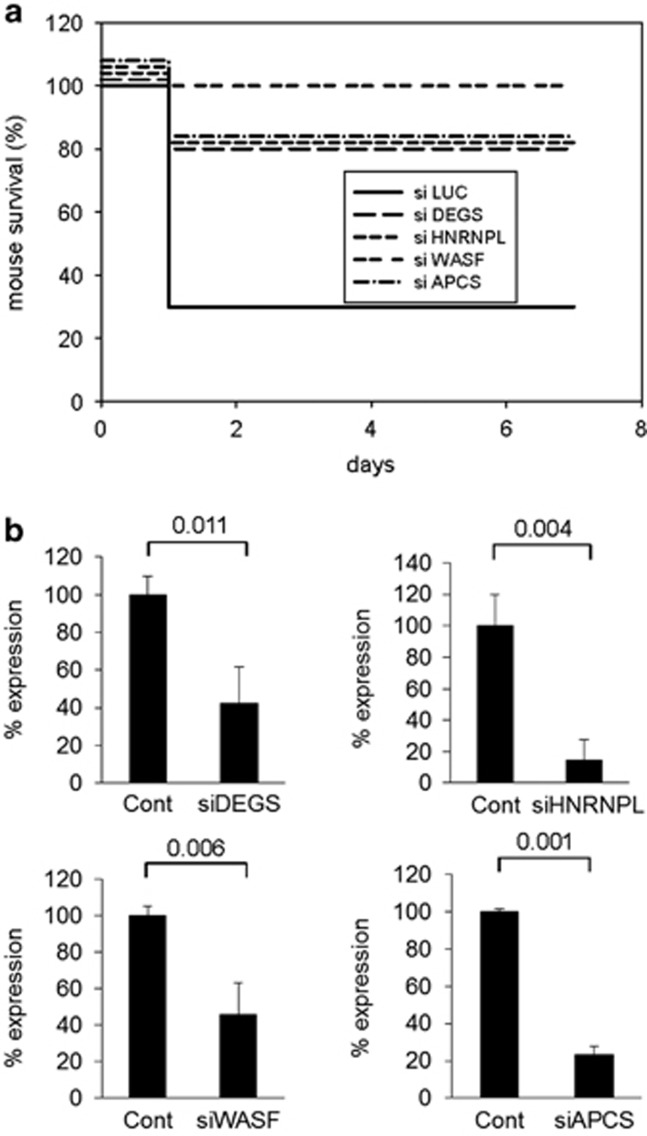
Introduction of selected siRNAs *in vivo* protected mice from FAS agonistic antibody (JO2)-induced death. (**a**) Seven-day survival (%) of NIH Swiss mice pretreated with siRNAs before administration of FAS agonistic Ab (JO2). siRNAs (3.5 mg/kg) targeting selected genes DEGS1, HNRNPL, WASF1, and APCS were complexed with Invivofectamine 2.0 Reagent and delivered by tail vein injection to livers of NIH Swiss mice (10 per group). LUC siRNA was used as a negative control. Seventy-two hours after siRNA introduction, mice were injected i.p. with JO2 (240 *μ*g/kg) and survival was monitored for 7 days. *P*-values for differences in the proportion of mice surviving on day 7 post JO2 administration were calculated using Fisher's Exact Test (two-tailed) (*P*=0.01 for WASF siRNA, *P*=0.03 for all other tested siRNAs). (**b**) Effectiveness of siRNAs targeting WASF, APCS, HNRNPL and DEGS in knocking down gene expression. RT–PCR of total liver RNA was used to determine the percentage of initial gene expression (with control siRNA targeting luciferase set at 100%) remaining in mice injected with gene-specific siRNAs. NIH Swiss mice (*n*=10 mice/group) were treated as in **a** and liver RNA was prepared from surviving mice on day 7 after JO2 injection. RT–PCR results were quantified using GelQuant.NET software provided by biochemlabsolutions.com. *P*-values were calculated using Student's *t*-test. Error bars indicate S.D

Mouse models established to simulate and study apoptotic pathways leading to ALF *in vivo* include treatment with concanavalin A (ConA), galactosamine, LPS, TNF, or anti-FAS agonistic Ab JO2.^12, 13, 14^ From our primary screens and analyses outlined above, we identified 102 different shRNAs targeting potential mediators of DR-dependent apoptosis. Since this exceeds the number of genes that we could individually validate *in vivo*, we chose four candidate genes (WASF1, HNRNPL, DEGS and APCS) for which relevance to FAS- or TNF-dependent apoptosis was not previously reported. To validate these genes in the mouse model of anti-FAS Ab-induced ALF,^12, 16, 26^ Stealth RNAi siRNA duplex oligoribonucleotides were synthesized to target the mouse orthologs of the genes. As the control we used siRNA targeting Firefly Luciferase gene. siRNAs were complexed with Invivofectamine 2.0 reagent and administered to NIH Swiss mice by tail vein injection, which results in delivery to the liver.^27, 28^ 72 h later, the mice were given an intraperitoneal (i.p.) injection of anti-FAS Ab (JO2, 240 *μ*g/kg) and their survival was monitored for 7 days. All groups pretreated with siRNAs showed significantly (*P*<0.05) improved survival, with 7-day survival rates of 80% (for HNRNPL, DEGS and APCS) to 100% (for WASF1) compared with 30% in the control group injected with siRNA targeting Luciferase gene (Figure 3a). Effective knockdown of siRNA-targeted genes in mice was confirmed by RT–PCR using total liver RNA prepared 7 days after anti-FAS Ab injection (Figure 3b). Collectively, these results provide *in vivo* confirmation of the involvement of the identified genes in the FAS apoptotic pathway leading to ALF and also validate the bioinformatics workflow and candidate gene selection criteria employed in these studies.

### Pharmacological inhibitors of candidate apoptosis modulators suppress FAS/TNF-induced cell death *in vitro*

**Figure 4:**
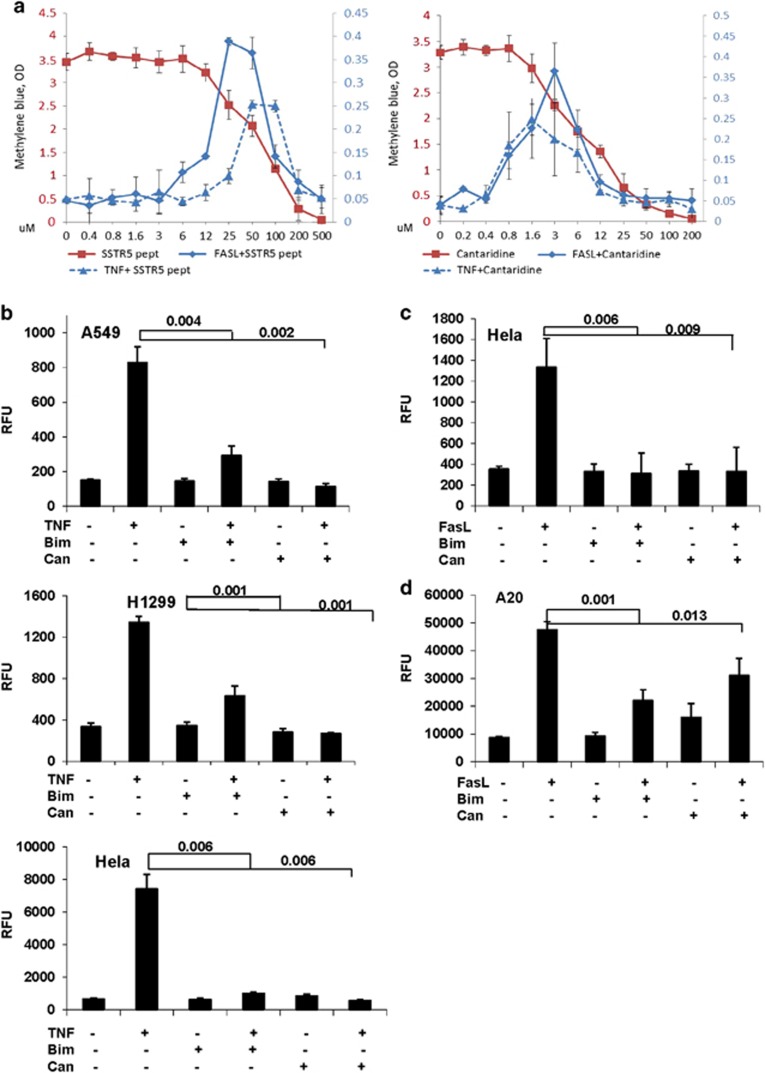
Reagents inhibiting shRNA target proteins identified in the screen suppress FASL/TNF-induced cell death *in vitro*. (**a**) Known pharmacological inhibitors of non-canonical targets identified in the library screen were tested for their effect on survival of cultured cells exposed to FASL or TNF. The tested inhibitors included Bim23056 (an SSTR5-blocking peptide, left) and cantharidin (a small-molecule PPP2R inhibitor, right). HeLa cells were pretreated for 12 h with the indicated concentrations of inhibitors and then treated with FASL (50 ng/ml, solid blue line) or TNF (50 ng/ml, blue dashed line) in the presence of cycloheximide (CHX, 2.5 mg/ml). The red lines correspond to treatment of cells with inhibitors alone (without FASL or CHX), plotted on the red *y*-axis on the left. Treatment with inhibitors and FAS/CHX is plotted on the blue *y*-axis on the right. Cell viability was determined 24 h after FASL addition by quantitation of methylene blue staining (optical density (OD) at 540 nm). Error bars indicate standard deviations for the means of three independent experiments. (**b–d**) Chemical inhibition of candidate targets SSTR5 and PPP2R5A leads to suppression of TNF- and FASL-induced activation of caspases 3/7 and 8 *in vitro*. (**b**) Caspase-3/7 activity was determined by Caspase-Glo assay (a luminogenic assay with a DEVD-containing substrate; RFU, relative fluorescence units) in A549 (top), H1299 (middle) and HeLa (bottom) cells left untreated, treated with TNF (50 ng/ml)+cycloheximide (2.5 *μ*g/ml), Bim23056 (200 *μ*M) or cantharidin (0.8 *μ*M) alone, or pretreated with Bim23056 (200 *μ*M) or cantharidin (0.8 *μ*M) for 0.5 h before addition of TNF+cycloheximide. Caspase activity was determined 4 h after TNF application. (**c**) Caspase-3/7 activity was determined in HeLa cells as described in **b** except that FASL (50 ng/ml) was used instead of TNF and caspase activity was measured at 6 h after FAS application. (**d**) Caspase-8 activity was determined in A20 cells as in **c** with the only difference being use of an LETD-containing luminogenic substrate. For (**b–d)**, the values shown are the means of at least three independent experiments. Error bars indicate standard deviations. *P-*values were calculated using a moderated Student's *t*-test with FDR correction

Network-based methods have been successfully used to identify association levels between genes and diseases and to predict drug–target (DT) interactions that can guide drug repositioning (i.e., use of an existing drug against new targets and/or indications).^29, 30^ We built a bipartite-graph composed of all FDA-approved drugs and proteins linked by drug–target binary associations. Computational drug–target prediction was performed by utilization of several local and global network prioritization methods using RNAi hits as an input. We used overlapping of a complementary target protein (TP) network with our F1 network and flow-based network propagation to prioritize drug targets by their proximity to canonical FAS/TNF genes. The predictions of these methods were combined using a Gaussian Naive Bayes (GNB) classifier, resulting in a set of prioritized drug targets (see Methods and Supplementary Table 9).^31^ Based on our DT networks, we identified inhibitory agents targeting two gene products identified as candidate modulators of FAS/TNF-dependent apoptosis in our screen: somatostatin receptor 5 (SSTR5) (targeted by BIM 23056, a SSTR5-blocking peptide^32^) and PPP2R5A, the regulatory subunit of PP2A protein serine/threonine phosphatase (targeted by cantharidin, a chemical inhibitor/natural compound isolated from the blister beetle^33^). Next, we tested the effect of these two agents on FAS- and TNF-induced apoptosis in a panel of cell lines (HeLa, H1299 and A549, representing cervical adenocarcinoma, non-small cell lung carcinoma, and lung carcinoma cells, respectively) by pretreating the cells with inhibitors for 12 h before addition of FASL (HeLa) or TNF (HeLa, H1299, and A549) and then assessing cell viability 24 h later. In all cases, the tested agents dramatically increased cell survival within a specific concentration range at which they were not highly toxic themselves (Figure 4a). Suppression of DR-mediated apoptosis by Bim23056 and cantharidin was also detected on a biochemical level as significantly reduced FASL/TNF-induced activation of the apical initiator caspase, caspase-8,^34, 35^ and downstream effector caspases-3/7 ^36^ in multiple cell lines (Figures 4b and d).

### Pharmacological inhibitors of new candidate apoptosis modulators SSTR5 and PPP2R5A protect mice against lethal ALF

**Figure 5:**
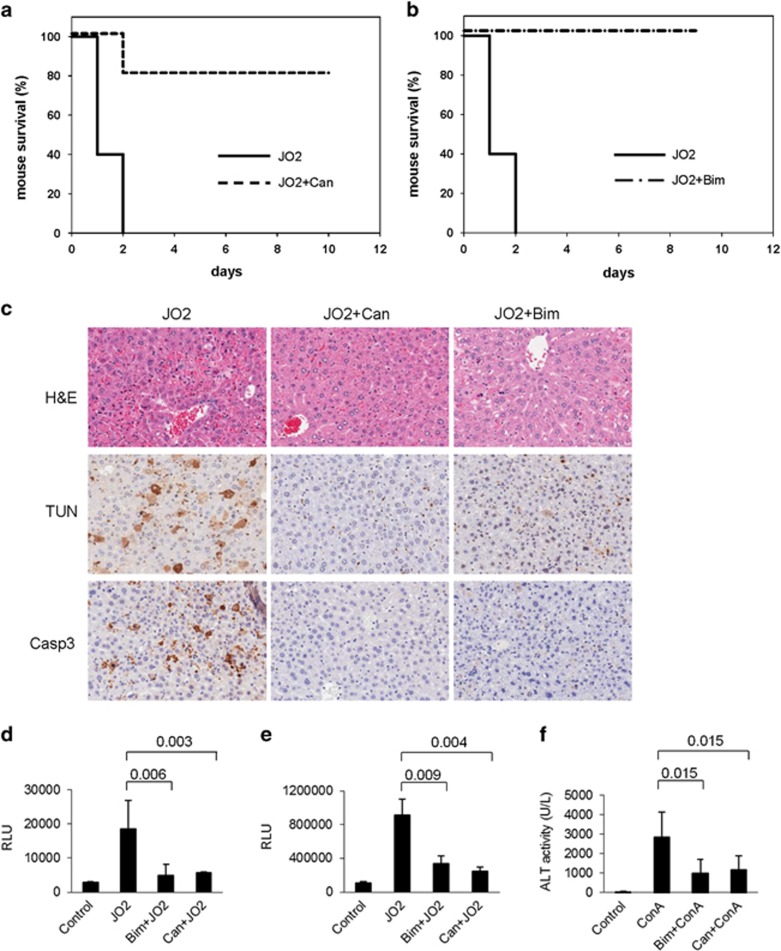
Chemical inhibition of candidate targets SSTR5 and PPP2R5A increases mouse survival and reduces liver damage following FAS agonistic antibody (JO2) administration. Kaplan–Meier survival curves for NIH Swiss mice (*n*=10 per group) injected i.p. with (**a**) Bim 23056 (1 mg/kg) 1 h before injection of JO2 (240 *μ*g/kg; *P*=0.0007; *n*=10 per group) or (**b**) cantharidin (0.25 mg/kg; *P*=0.0007; *n*=10 per group) 4 h before JO2. *P*-values are for comparison of survival at 48 h between groups pretreated with inhibitor before JO2 or given JO2 alone using Fisher's exact test. (**c**) NIH Swiss mice were pretreated with PBS (control, left), cantharidin (0.25 mg/kg, middle) or Bim 23056 (1 mg/kg, right) for 1 h, 1 or 4 h, respectively, before injection of FAS agonistic JO2 antibody (240 *μ*g/kg). Five hours later, liver tissue was collected and processed for H&E staining (top row) and detection of apoptotic cells by TUNEL assay (middle) and cells with activated caspase-3 by immunostaining with antibodies against cleaved Casp-3 (Asp175) (bottom). Representative photographs from groups of three mice are shown. (**d–f**) Chemical inhibition of candidate targets SSTR5 and PPP2R5A *in vivo* leads to suppression of FAS-mediated caspase activation in the liver and release of ALT from the liver into the bloodstream. (**d** and **e**) Bim 23056 (1 mg/kg) and cantharidin (0.06 mg/kg) were injected i.p. into NIH Swiss mice 1 h before i.p. injection of JO2 (240 mg/kg). Cytosolic liver extracts were prepared 3 h later and used for measurement of caspase 8 (**d**) and caspase-3/7 (**e**) activity with luminogenic LETD- or DEVD-containing substrates. (**f**) ALT activity was determined in serum samples collected from NIH Swiss mice pretreated with cantharidin (0.125 mg/kg) or Bim 23056 (2 mg/kg) 1 h before i.v. injection of ConA (15 mg/kg) or treated with ConA alone. Serum was collected 20 h after ConA injection. For **d**–**f**, ‘control' samples were from animals that received no treatments and the values shown are the means of at least three independent experiments. Error bars indicate standard deviations. *P*-values were calculated using a moderated student's *t*-test

To assess the effects of SSTR5 inhibitor Bim 23056 and PPP2R5A inhibitor cantharidin on the toxicity of anti-FAS agonistic Ab *in vivo*, we used a dose of JO2 Ab (240 *μ*g/kg, three times the LD50) that caused death of 100% of treated NIH Swiss mice from ALF within 48 h of JO2 administration. The optimal inhibitor doses and times of administration relative to JO2 injection were established in *in vitro* (e.g., Figure 4) and in *in vivo* experiments (data not shown). Injection of mice with 1 mg/kg BIM 23056 1 h before JO2 Ab increased mouse survival at 48 h after JO2 injection from 0 to 80% (*P*=0.0007; *n*=10/group) and no additional mortality was observed up to the end of the 10-day observation period (Figure 5a). Similarly, injection of 0.25 mg/kg cantharidin 4 h before JO2 injection increased mouse survival at 48 h (as well as day 10) from 0 to 100% (*P*=0.0001; *n*=10/group) (Figure 5b). Thus, inhibition of either SSTR5 or PPP2R5A had potent protective effects against lethal ALF *in vivo*.

The hepatoprotective effects of Bim 23056 and cantharidin in JO2-injected NIH Swiss mice were further characterized by analyzing liver sections for tissue and cellular morphology, presence of apoptotic cells, and *in situ* caspase activity. Hematoxylin-eosin (H&E) stained liver sections revealed that while control mice treated with JO2 alone had extensive liver damage (e.g., extensive hemorrhaging, confluent hepatocyte necrosis, and inflammatory cell infiltration), this damage was effectively minimized by pretreatment of mice with either Bim 23056 or cantharidin (Figure 5c). Furthermore, compared with liver sections from mice given JO2 alone, those from mice pretreated with BIM 23056 or cantharidin had fewer apoptotic hepatocytes detected by TUNEL staining and reduced caspase-3 activation detected by immunostaining (Figure 5c). Consistent with this, cytosolic liver extracts prepared from mice treated with Bim 23056 or cantharidin before JO2 Ab injection showed reduced activity of caspase-8 and caspases-3/7 compared with those prepared from mice given JO2 alone (Figures 5d and e).

Finally, as an independent means of confirming the capacity of Bim 23056 and cantharidin to inhibit FAS-dependent apoptosis in the liver, we used a second mouse model of FAS-mediated fulminant hepatitis induced by intravenous (i.v.) injection of the lectin ConA, a T-cell mitogen that rapidly induces liver injury.^15^ Anti-FAS siRNA was used to confirm that ConA-induced liver damage in this model is indeed FAS-mediated.^16^ Serum levels of alanine transaminase (ALT) released from damaged hepatocytes indicate the extent of liver damage in the ConA-induced hepatitis model. Significantly, serum ALT levels were reduced in mice pretreated with Bim 23056 or cantharidin (Figure 5f), thus providing additional support for roles of their targets (SSTR5 and PPP2R5A, respectively) in FAS-mediated apoptosis.

## Discussion

In this work, we applied an RNAi-based screening approach, coupled with computational network modeling, to identify regulators of DR-mediated apoptosis pathways *en masse* (Supplementary Fig. 3). It involved functional selection of clones from a complex shRNA library that allowed cells to survive exposure to FASL and/or TNF, the inducers of two different (although overlapping in some components) DR-mediated apoptotic pathways; identified shRNAs did not have any effect on intrinsic staurosporine-induced apoptosis (Supplementary Fig. 4). This was directly followed by *in vivo* testing of available pharmacological agents against candidate targets identified in the screen (including systemically administered synthetic siRNAs). Our idea of testing known inhibitors of screen-identified targets represents an integrated target-drug discovery strategy allowing for independent validation and rapid prioritization of targets based on their significance as components of the pathway of interest. The approach also creates a solid foundation for subsequent mechanistic studies aimed at defining the precise role of each identified target within a given pathway. In our paper, we concentrated on the first direction, providing the list of potential targets enabling us to make a shortcut towards pharmacological inhibitors of FAS and TNF pathways with subsequent validation in mouse models of ALF. Similarity of components of FAS and TNF pathways in different cell types made it possible to use as a screening readout, instead of liver cells, a well-established FAS and TNF sensitive cell system (HeLa). Further validation of hits isolated in HeLa cells demonstrated that this presumption worked well. First, the set of isolated target candidates contained the vast majority of known components of DR-dependent apoptotic pathways. Next, four of the candidate modulators of DR-mediated apoptosis that were identified in the screen but had not been previously implicated in cell death pathways (HNRNPL, DEGS, APCS, and WASF1) were validated by showing that siRNA-mediated knockdown of gene expression inhibited FAS/TNF-induced apoptosis in cultured cells and prevented death of mice treated with FAS agonistic Abs. For two additional candidates, SSTR5 and PPP2R5A, existing pharmacological inhibitors were computationally predicted through DT network models and found to block FAS-dependent apoptosis in cultured cells and to reduce the lethality of anti-FAS Ab and ConA treatment in mice. Notably, in these *in vivo* ALF models, improvement of animal survival following inhibitor treatment was accompanied by less severe histomorphological changes and reduced caspase activity and presence of apoptotic cells in liver tissue.

SSTR5 (Somatostatin receptor 5, inhibited by the synthetic peptide Bim 23056 (ref. 32)) belongs to a family of five G protein-coupled receptors that mediate the action of somatostatin.^37^ Somatostatin was originally isolated from the hypothalamus as an inhibitor of growth hormone release and is known to have diverse physiological functions including inhibition of secretory and proliferative processes.^38^ PPP2R5A belongs to a family of regulatory subunits of the PP2A family of ser/thr protein phosphatases generally involved in negative regulation of cell division. Activity of PPP2R5A is inhibited by cantharidin, a natural compound isolated from the blister beetle,^39^ which has been used as a medicinal agent since antiquity and is now under clinical evaluation as an antitumor agent.^40^ Although neither SSTR5 nor PPP2R5A has been previously implicated in modulation of DR-mediated apoptotic signaling, the data reported here clearly indicate their key roles in the FAS and TNF pathways and their potential as therapeutic targets.

Тo test feasibility of our methodology we chose a mouse model of ALF, a complex disease that can be provoked by viral infection, exposure to hepatotoxic agents or ischemic conditions.^41^ Although a complete understanding of the pathophysiology of ALF has yet to be achieved, it is known that on the molecular level, FAS/FASL and TNF/TNFR apoptotic pathways have key roles in the disease.^7, 8, 9, 42^ Therefore, targeting of DR apoptotic pathways represents a promising potential strategy for therapeutic intervention in ALF.^43, 44^ Although selective or pan-caspase inhibitors can efficiently block extrinsic (DR-mediated) apoptosis, in several recent animal studies these agents could only delay but not prevent cell death mediated by the intrinsic (mitochondrial) apoptotic pathway.^45, 46, 47, 48^ This suggests that the search for potential targets for therapeutic intervention should be focused on events in apoptotic pathways that lie upstream of caspase activation and the results of our work have brought us to them. More studies are required to determine whether isolated targets and pharmacological agents may have clinical potential. Even though their value for treatment ALF is questionable because of the acute nature of the disease (which may be too late for treatment by apoptosis inhibitors at the time of manifestation of its symptoms), treatment of other pathologies may benefit from therapies involving identified targets. These may include less rapidly developing and earlier diagnosed neurodegenerative,^18^ cardiac,^19^ renal,^21^ pulmonary^20^ and other disorders^22^ associated with DR-mediated apoptosis.

In summary, our results provide strong validation of the developed shRNA screening-network modeling methodology for identification of apoptosis modulators and presents several new candidate targets, as well as two ‘repositioned' drugs, for further investigation as treatment strategies for ALF. More broadly, this study exemplifies how RNAi technology and supporting computational network approaches can be used to systematically identify and evaluate new therapeutic targets for complex human diseases.

## Materials and Methods

### Reagents

Cantharidin, concanavalin A, and cycloheximide were purchased from Sigma Aldrich (St.Louis, MI, USA); Bim23056, from Tocris Bioscience (Bristol, UK); TNFα (human recombinant) and FASL from Peprotech (Rocky Hill, NJ, USA); JO2 antibody from Becton Dickinson Biosciences (Franklin Lakes, NJ, USA); anti-caspase-3 (Asp175) antibody from Cell Signaling Technology (Danvers, MA, USA).

### Cell cultures

Tumor cell lines, including HeLa, H1299, A549 and A20, were grown in DMEM supplemented with 10% FBS.

### Mice

Female NIH Swiss and BALB/c mice (age 8–10 weeks) were purchased from NCI (Frederick, MD, USA). Mice were housed under aseptic conditions and fed radiation-sterilized food. The animals were treated in accordance with a protocol approved by the RPCI Institutional Animal Care and Use Committee.

### Construction of the 27 K Pathway Decipher shRNA library

Selection of the 5046 Pathway Decipher genes is described in Supplementary Methods. To generate a library of shRNAs targeting Pathway Decipher genes with fivefold redundancy, ~27 000 unique shRNA-encoding oligonucleotides were synthesized on a custom array (Agilent, Santa Clara, CA, USA), collected into a single pool, PCR amplified to generate terminal adaptor sequences with BpiI-compatible sequences, and then cloned into the lentiviral vector pRSI-U6wt-UbiC-tagRFP-2A-Puro downstream of the U6 RNA pol III promoter. To maximize target gene knockdown and minimize off-target effects, unique 21-base shRNA guide sequences complementary to target transcripts were first selected from experimentally confirmed shRNA sequences from the RNAi Consortium Project (TRC) and then ‘triaged' using a support vector machine (SVM) off-target search classifier (unpublished data). Each shRNA in the library has a unique target gene-homologous sequence and a unique barcode for identification and quantification of the shRNA's representation in the library or in genomic DNA isolated from library-transduced cells by high-throughput (HT) sequencing (Illumina, San Diego, CA, USA; Hi-Seq Sequencer). After library construction, the relative representation of each shRNA sequence was determined to confirm presence of at least 90% of the designed shRNAs (within a 10-fold distribution range) in the final library (data not shown).

### Screening of the 27 K Pathway Decipher shRNA library for inhibition of FAS- and TNFR-mediated apoptosis

The 27 K shRNA library was packaged using HEK-293 T cells co-transfected with psPAX2 and pMD2 (Addgene, Cambridge, MA USA) to produce VSV-g pseudotyped lentiviral particles. The packaged library was extracted from the cell culture supernatant 48–72 h post transfection, titered, and then used to transduce HeLa cells. For each screening, nine replicate cultures, each containing at least 30 million cells, were transduced at MOI=0.2, which was sufficient to cover the library complexity with 200-fold redundancy and ensure that most cells acquired only one unique bar-coded shRNA construct. Two days after transduction, puromycin was added to the cell culture media at 1 *μ*g/ml. Two days later, viable puromycin-resistant cells were plated in fresh medium and three of the library-transduced cell replicates were treated with FASL (50 ng/ml) and three replicates were treated with TNF-*α* (50 ng/ml) to induce apoptosis, and three were collected to represent the control untreated replicates. At 24 h, FASL and TNF were removed from the media. Surviving cells were grown for 3 more days to allow for recovery. After treatment, shRNA-specific barcodes were PCR amplified from total genomic DNA isolated from each pool of surviving cells using primers that are specific for vector sequences flanking the barcode site and produce amplicons with incorporated primer binding sites for Illumina HTS2/Gex1 primers. The Illumina-specific primers were then used for a second round of PCR, so that the amplified products could be sequenced using the Single-Read Cluster Generation Kit (Illumina). More than 2x10^7^ sequencing runs were read for each sample.

### Arrayed sub-library of shRNAs for individual validation assay

To confirm the validity of our strategy, an arrayed sub-library of 200 shRNAs targeting 100 non-canonical FAS/TNF pathway targets identified as hits in our screen was constructed (two different shRNA constructs per target). Eighteen ‘control' shRNAs were also included in the sub-library: two targeting luciferase (LUC, negative controls) and 16 targeting eight canonical FAS/TNF pathway components identified in the screen. The sub-library employed a vector and experimental strategy identical to that described above for the original 27 K Pathway Decipher library screen, except that it was executed using an arrayed format allowing individual shRNA confirmation rather than the pooled format used for cell-based shRNA screening. Individual shRNAs of the arrayed sub-library were packaged in HEK293T cells and used to transduce HeLa cells. Transduced cells were selected in puromycin and then challenged with FASL (50 ng/ml, PeproTech, Rocky Hill, NJ, USA) or TNF (50 ng/ml, PeproTech) together with cycloheximide (2.5 *μ*g/ml). Cyclohexamide was included to increase cytotoxicity through inhibition of survival proteins such as NF-*κ*B. Cell viability was determined 24 h post-challenge by Resazurin fluorescence assay. An identical assay was performed in H1299 cells with TNF treatment (H1299 cells do not express FAS).

### Integrative informatics and pathway analysis

For network and pathway enrichment analysis, we used the Reactome FI and Cytoscape plugin. Significantly enriched pathway gene sets were exported and analyzed using Enrichment Map to determine relationships between pathways.^49^ To investigate functional network and gene ontology relationships, candidate genes were first categorized using GO analysis performed with DAVID software. The reference population was defined by our gene study set (see above) and an adjusted *P-*value of 0.05 (Benjamini and Hochberg correction) was used as the threshold for identification of significant GO terms.

### Functional interaction network construction

The functional interaction (FI) network used in this study was described in Wu *et al.*^50^ Briefly, we compiled pairwise protein relationships extracted from protein–protein interactions from human, yeast, worm, and fly gene co-expression data sets, Gene Ontology annotations, domain–domain interactions, and text-mined protein interactions. A Bayes classifier was used to predict functional interactions for protein pairs, and the predicted FIs were merged with FIs extracted from curated pathways in Reactome,^51^ KEGG,^52^ NCI-PID,^53^ Panther ^54^ and CellMap (http://cancer.cellmap.org/cellmap).

We obtained the protein–protein interaction network from the Human Protein Reference Database (HPRD). This network contained 9667 proteins and 76 132 binary edges. We obtained KEGG, Biocarta, and Reactome gene sets from MsigDB^55^ and all conserved sub-networks in the human protein–protein interaction network. To reduce bias to disease proteins in the protein–protein interaction network, we used the extended protein–protein interaction network suggested by.^50^ The extended protein–protein interaction network is generated by combining the HPRD, OPHID, BIND, and MINT databases, and has a similar degree of interactions for both disease and non-disease proteins.

### Drug–target network construction

We used the DTome web-based tool for drug–target interactome construction (http://bioinfo.mc.vanderbilt.edu/DTome). The DTome tool utilizes web-based queries to search candidate drugs and then construct a DTome network by extracting and integrating four types of interactions: adverse drug interactions, drug–target (DT) interactions, drug-gene associations, and target-/gene-protein interactions. A DT network is a bipartite graph in which every link connects a drug to a protein if the protein is a known target of the drug. To generate a DT network, all FDA-approved drugs and their known targets were extracted from the Integrity database (http://integrity.thomson-pharma.com), which is comprised of a large collection of drugs annotated with information on their respective targets. In the ‘drug network,' nodes represent drugs and two drugs are connected to each other if they share at least one TP. In the complementary TP network, nodes are proteins and two proteins are connected if they are both targeted by at least one common drug, hence providing a protein-centric view of pharmacological space.

### MCL network clustering

We chose MCL (Markov Cluster Algorithm, CWI, Amsterdam, The Netherlands) as the network-clustering algorithm in order to take advantage of edge weights. We weighted each interaction edge according to the absolute value of the PCC of the expression levels of the two genes connected by the edge. To control the size of network modules generated from the MCL clustering, we used 6.0 as the inflation coefficient and average PCC>0.27. For permutation testing, we randomly swapped expression values for all genes, or randomly selected genes from the FI network.

### Key nodes searching algorithm

The Explain key nodes searching algorithm (BIOBASE Biological Databases, www.biobase-international.com) was used to identify common signaling molecules in the network vicinity of the query (input) gene list. The underlying application searches the network in a specified range starting from each input molecule in order to find the most proximal molecule that is connected to a maximal number of input molecules. This is achieved by scoring each node that was visited on the path from any input molecule. Since the resulting score may be determined by a generally high level of connectivity of some molecules, the total number of connections reaching every node is also taken into account by the algorithm and penalized. This leads to a preference for molecules that are specific for input genes.

### Cell viability assays

*Methylene blue assay*. Viable attached cells were quantified by staining plates with 0.5% methylene blue, after which the dye was eluted from the attached cells into 100 *μ*l of 0.1% hydrochloric acid and its optical density was measured at 540 nm.

*Resazurin assay*. The CellTiter-Blue Cell Viability Assay (Promega, Madison, WI, USA) was performed as recommended by the manufacturer. In this assay, conversion of the resazurin dye to a fluorescent product is directly proportional to the metabolic capacity (and thus, viability) of a cell population.

### *In vivo* validation of non-canonical FAS pathway targets in mice by systemic siRNA administration followed by challenge with anti-FAS agonistic antibodies

To confirm the role of selected genes in FAS-mediated apoptosis *in vivo*, we generated siRNAs against the mouse orthologs of four selected human genes (APCS, WASF, DEGS, and HNRNPL) and the control Luciferase (LUC) gene. siRNAs were produced as Stealth RNAi siRNA Duplex Oligoribonucleotides (Invitrogen) with the following sequences: 5'-APCS: CCUGCGACAGGAGCAGGAUAACUAU-3', 5'-AUAGUUAUCCUGCUCCUGUCGCAGG: WASF: CCCUUACAGGGAUGAUGGUAAGGAA-3', 5'-UUCCUUACCAUCAUCCCUGUAAGGG: DEGS: GCUUCUCGUCCAGCUGGCUUCAUUU-3', 5'-AAAUGAAGCCAGCUGGACGAGAAGC: HNRNPL: GGAGCGUAAACAGCGUGCUUCUGUU-3', 5'-AACAGAAGCACGCUGUUUACGCUCC: LUC: GCCAUUCUAUCCUCUAGAGGAUGGAA-3', 5'-TTCCAUCCUCUAGAGGAUAGAAUGGC-3'.

Twenty nanomoles of each siRNA was resuspended in RNase-free water and complexed with Invivofectamine 2.0 Reagent (Invitrogen–Thermo Fisher Scientific, Waltham, MA USA) according to Invitrogen's protocol for *in vivo* siRNA delivery. The Invivofectamine-siRNA mix was injected (3.5 mg/kg) into the tail vein of 8-week-old NIH Swiss female mice. This injection route delivers siRNAs preferentially to the liver.^27^ Control mice were injected with siRNAs without Invivofectamine. For each siRNA, 10 mice were injected with Invivofectamine and 10 mice were injected without Invivofectamine. To induce ALF, 72 h after siRNA introduction, mice were injected i.p. with anti-FAS agonistic Ab (JO2, 240 *μ*g/kg, PeproTech). Mouse survival was monitored for 7 days after JO2 injection. At the end of the monitoring period, liver tissue was collected after euthanasia from 3 surviving mice of each group and frozen for analysis of the effectiveness of siRNA-mediated inhibition of gene expression by RT–PCR.

### RT–PCR

To test the effectiveness of *in vivo* administered siRNAs in reducing levels of their target mRNAs in mouse livers, we isolated total liver RNA from mice that survived the experiment described above (after delivery of siRNA targeting 4 genes WASF1, HNRNP, DEGS, APCS) and control (siRNA LUC) mice (three mice per group) using Trizol reagent (Invitrogen). cDNA was prepared using iScript (Bio-Rad, Hercules, CA, USA) and PCR amplified using Taq PCR Master Mix (USB Affymetrix, Santa Clara, CA, USA) and primers designed from sequences in the EMBL/GenBank/DDBJ database. The following primers were used (written 5' to 3'): DEGS1: Forward: 5'-TAGCCGCGTGTCCCGAGAGG-3', Reverse: 5'-AACCAGCGGTTCCACAGGGC-3'; WASF1: 5'-CGCCATCCATCCACCCTGCC-3' (F), 5'-ACTCCACAGCAATGCGGCGA-3' (R); APCS: 5'-CCACTTGGGAGTCCTCCTCTGGC-3' (F), 5'-TCAATCCCAGACACGGGGCCT-3' (R); HNRPL: 5'-CCGGAGCGTAAACAGCGTGC-3' (F), 5'-CCGCTGGCGTTTGTTGGGGT-3' (R); and GAPDH: 5'-CTCAACTACATGGTCTACATG-3' (F), 5'-TGGCATGGACTGTGGTCATGAG-3' (R).

PCR products were analyzed by electrophoresis in 1% agarose gels stained with ethidium bromide and bands were quantified using GelQuant.NET software (biochemlabsolutions.com). Mean (*n*=3 mice) expression levels for each mRNA normalized to mean GAPDH expression are shown as ‘% remaining expression' after siRNA treatment (set at 100% for LUC control siRNA treatment). The standard error of the mean was calculated.

### Testing of pharmacological inhibitors of non-canonical FAS pathway targets in the mouse JO2-induced ALF model

We performed preliminary toxicological testing of the chosen compounds (found by PubMed search) Bim 23056 and cantharidin, (inhibitors of SSTR5 and PPP2R5A, respectively) to determine their maximal tolerable doses in female NIH Swiss mice (8–10 weeks old). We then determined the dose range for each compound that protected female NIH Swiss mice from anti-FAS Ab JO2-induced ALF and the optimal time window for their application relative to JO2 injection (data not shown). Based on the results of these pilot experiments, groups of 10 female NIH Swiss mice were pretreated with PBS (control), Bim23056 (1 mg/kg) or cantharidin (0.25 mg/kg) by i.p. injection 1 h, 4, or 2 h, respectively, before i.p. injection of JO2 antibody (240 *μ*g/kg). Mouse survival was determined 48 h after JO2 injection and analyzed by Fisher's Exact Test (2-tailed). Survival continued to be monitored up to day 10 after JO2 injection, but no additional mortality was observed after 48 h. This experiment was performed three times.

In addition, NIH Swiss mice (three per group) pretreated as described above with PBS (control), Bim-23056 (1 mg/kg) or cantharidin (0.25 mg/kg) were injected with JO2 antibody (240 *μ*g/kg), and 5 h later, euthanized for collection of liver tissue. Formalin-fixed, paraffin-embedded liver sections were used for histological examination of liver injury (hematoxylin-eosin staining), detection of apoptotic cells by TUNEL assay (TdT),^56^ and assessment of caspase-3 activation by immunostaining with antibodies against cleaved Casp-3 (Asp175) (Cell Signaling Technology, Inc.).

### Induction of acute hepatitis with concanavalin (ConA) and measurement of ALT activity in mouse serum samples

Female NIH Swiss mice received no pretreatment or were pretreated with Bim 23056 (2 mg/kg) or cantharidin (0.25 mg/kg) by i.v. injection 1 h before i.v. injection of ConA (15 mg/kg; Sigma-Aldrich) via the tail vein. After 20 h, activity of the liver enzyme alanine aminotransferase (ALT) was measured in mouse serum samples using ALT/SGPT Bio-Chemistry reagent (Stanbio Laboratory, Boerne, TX, USA) according to the manufacturer's instructions. Briefly, ALT-working reagent and serum samples were mixed at a 10:1 ratio in cuvettes and then incubated in a 37 °C water bath for 3 min. Absorbance at 340 nm was monitored for at least 5 min using a Beckman Coulter DU800 spectrophotometer (BioSpec Products, Inc., Bartlesville, OK, USA). The decrease in absorbance per minute (delta A, indicative of ALT activity) was determined and used to calculate ALT activity in samples according to the formula: units per liter (U/l)=delta A/Min x1746.

### Caspase-Glo 3/7 and 8 assays

#### *In vitro* assays

Apoptosis was induced by adding a mixture of TNF (50 ng/ml) and cycloheximide (2.5 *μ*g/ml) or FASL (50 ng/ml) and cycloheximide (2.5 *μ*g/ml) to cultures of cells that were not pretreated (control) or were pretreated with Bim 23056 (200 *μ*M) or cantharidin (0.8 *μ*M) for 0.5 h. A549, H1299 and HeLa cells were used for TNF experiments and HeLa and A20 cells were used for FASL experiments (A20 cells express higher level of FAS than HeLa cells and therefore show a stronger cytotoxic effect). Four hours after TNF/FASL+cyclohexamide application, caspases-3/7 and caspase-8 activity was measured in cell lysates using Caspase-Glo assay kits (Promega, Madison, WI, USA) according to the manufacturer's protocol. The readout for the assay is luminescence produced upon cleavage of a proluminescent substrate containing DEVD or LETD cleavage sites specifically recognized by caspases-3/7 and caspase-8, respectively. Luminescence was measured using a plate-reading luminometer (Victor, Perkin Elmer, Waltham, MA, USA).

#### *In vivo* assays

Cytosolic extracts from frozen liver tissues of mice pretreated (i.p. injection) with Bim 23056 (1 mg/kg), cantharidin (0.06 mg/kg) or PBS (control) 1 h before i.p. injection of JO2 (240 *μ*g/kg) were prepared 3 h later by homogenization (using a bio-pulverizer (BioSpec)) in hypotonic extraction buffer (25 mM HEPES, pH 7.5, 5 mM MgCl_2_ 1 mM EGTA, and 1 mM Pefablock) with protease inhibitor cocktail. Extracts were centrifuged (13 000 r.p.m., 4 °C, 15 min) and the total protein concentration of the supernatant was adjusted to 1 mg/ml by addition of extraction buffer. Equal volumes of Caspase-Glo assay reagent (Promega) and 10 *μ*g/ml cytosolic extract were added to wells of 96-well plates and incubated for 30 min at room temperature. Luminescence indicative of caspases-3/7 or caspase-8 activity (see above) was measured using a plate-reading luminometer (Victor x3 Multilabel plate Reader, Perkin-Elmer, Waltham, MA, USA).

## Abbreviations

Ab: antibody
ALT: alanine transaminase
ALF: acute liver failure
APO-1: apoptosis antigen 1
A549: lung carcinoma cell line
Bim 23056: SSTR-blocking synthetic peptide
ConA: concanavalin A
CD95: cluster of differentiation 95 (FAS receptor)
cAMP: cyclic adenosine monophosphate
cGMP: cyclic guanosine monophosphate
CHX: cycloheximide
DR: death receptor
DT: drug–target
FASL: FAS ligand
FI: functional interaction
GNB: Gaussian Naive Bayes
H@E: hematoxylin-eosin
IL-1: interleukin 1
JO2: anti-FAS agonistic antibody
LD50: the median lethal dose
LPS: lipopolysaccharide
LUC: luciferase
MAPK: mitogen-activated protein kinases
PPP2R5A: regulatory subunit of the PP2A family of ser/thr protein phosphatases
PuroR: puromycin resistance
pTV1: porcine Teschovirus
RT–PCR: reverse transcription polymerase chain reaction
shRNA: short hairpin RNA
siRNA: small interfering RNA
SSTR5: somatostatin receptor 5
SVM: support vector machine
TNF: tumor necrosis factor
TNFR: tumor necrosis factor receptor
TP: target protein
TUNEL: terminal deoxynucleotidyl transferase dUTP nick end labeling (used as an apoptosis detection assay)
