# Supplementary material for: Functional genetics-directed identification of novel pharmacological inhibitors of FAS- and TNF-dependent apoptosis that protect mice from acute liver failure
Source: Cell Death Dis. 2016 Mar 17;7(3):e2145–. doi: 10.1038/cddis.2016.45 (PMC4823946; doi:10.1038/cddis.2016.45)
Supplement: Supplementary Tables [file cddis201645x1.pdf]

Komarov et al. Functional genetics-directed identification of novel pharmacological inhibitors of FAS- and TNF-dependent apoptosis that protect mice from acute liver failure

Supplemental Table 1:

Pathway Decipher gene annotations, disease & drug processes

Protein class	Actual	n	R	N	Expected	Ratio	p-value	z-score	In data set	In protein function	Protein function in database
**Ligands**	318	5488	526	25169	114.7	2.773	1.728E-83	21.7	0.0579	0.6046	0.0209
**Kinases**	339	5488	652	25169	142.2	2.385	9.492E-66	18.91	0.0618	0.5199	0.0259
**Enzymes**	1361	5488	2716	25169	592.2	2.298	0.000E+00	37.82	0.248	0.5011	0.1079
**Phosphatases**	99	5488	228	25169	49.71	1.991	2.022E-13	7.941	0.018	0.4342	0.0091
**Transcription factors**	393	5488	956	25169	208.5	1.885	8.491E-43	14.74	0.0716	0.4111	0.038
**Receptors**	550	5488	1581	25169	344.7	1.595	1.137E-34	12.91	0.1002	0.3479	0.0628
**Proteases**	177	5488	561	25169	122.3	1.447	3.914E-08	5.654	0.0323	0.3155	0.0223
**Other**	2289	5488	18001	25169	3925	0.5832	0.000E+00	-55.34	0.4171	0.1272	0.7152

Columns have the following meaning:

Protein class a broadly defined protein function

Actual number of network objects from the activated dataset(s) for a given protein class

n number of network objects in the activated dataset(s)

R number of network objects of a given protein class in the complete database or background list

N total number of network objects in the complete database or background list

Expected mean value for hypergeometric distribution (n*R/N)

Ratio connectivity ratio (Actual/Expected)

z-score z-score ((Actual-Expected)/sqrt(variance))

p-value probability to have the given value of Actual or higher (or lower for negative z-score)

In data set fraction of network objects with a selected function in the activated dataset

In protein function fraction of network with a selected function in the activated dataset among network objects with this function in the complete database or background list

Protein function in database fraction of network objects with a selected function in the complete database or background list

Supplemental Table 1.

Komarov et al. Functional genetics-directed identification of novel pharmacological inhibitors of FAS- and TNF-dependent apoptosis that protect mice from acute liver failure

Supplemental Table 2:

List of shRNA target genes identified as candidate apoptosis modulators in FAS and TNF screens

A

GENE NAME	RefSeq ID	FASL	TNFa
LUC CONTROL 1		1.0	1.0
LUC CONTROL 2		1.2	0.8
CASP8 1	NM_001080125.1	23.6	20.0
CASP8 2	NM_001080125.1	24.5	22.2
CASP8 3	NM_001080125.1	24.3	19.2
FAS 1	NM_152872.1	25.0	1.1
FAS 2	NM_152872.1	24.7	1.4
FAS 3	NM_152872.1	22.7	1.5
FADD 1	NM_003824.2	21.3	1.7
FADD 2	NM_003824.2	21.3	1.6
BID 1	NM_197966.1	15.7	9.6
BID 2	NM_197966.1	8.2	9.9
TRAF2 1	NM_021138.3	3.3	10.2
TRAF2 2	NM_021138.3	3.3	8.5
TRADD 1	NM_003789.3	1.7	10.0
TRADD 2	NM_003789.3	2.1	7.5
TNFRSF1A 1	NM_001065.2	3.0	17.2
TNFRSF1A 2	NM_001065.2	2.0	14.5

B

GENE NAME	RefSeq ID	FASL	TNFa
AGXT2L1 1	NM_031279.2	21.4	2.0
AGXT2L1 2	NM_031279.2	9.9	2.0
AMY1C 1	NM_001008219.1	11.4	2.5
AMY1C 2	NM_001008219.1	9.9	1.2
ATG4B 1	NM_013325.4	4.3	1.6
ATG4B 2	NM_013325.4	19.0	1.3
BCHE 1	NM_000055.1	21.4	1.5
BCHE 2	NM_000055.1	14.0	1.4
CD1C 1	NM_001765.2	19.4	0.8
CD1C 2	NM_001765.2	20.1	1.1
CNTN1 1	NM_001843.2	9.4	0.8
CNTN1 2	NM_001843.2	6.7	1.2
CYP2C9 1	NM_000771.2	15.9	1.1
CYP2C9 2	NM_000771.2	14.8	1.0
DEGS1 1	NM_003676.2	16.0	1.8
DEGS1 2	NM_003676.2	14.5	1.1
ESR2 1	NM_001040275.1	9.1	0.6
ESR2 2	NM_001040275.1	19.6	0.8
MTIF2 1	NM_001005369.1	21.4	1.1
MTIF2 2	NM_001005369.1	8.7	0.7
MLH3 1	NM_014381.2	16.7	0.9
MLH3 2	NM_014381.2	16.3	1.0
NR1H2 1	NM_007121.2	8.6	0.7
NR1H2 2	NM_007121.2	10.0	0.8
POLR3A 1	NM_007055.2	8.7	0.5
POLR3A 2	NM_007055.2	7.9	1.1
TAP1 1	NM_000593.5	18.1	1.5
TAP1 2	NM_000593.5	7.0	1.7
UBE2F 1	NM_080678.1	7.0	1.1
UBE2F 2	NM_080678.1	6.7	1.0

C

GENE NAME	RefSeq ID	FASL	TNFa
ADORA1 1	NM_000674.2	10.3	7.4
ADORA1 2	NM_000674.2	4.4	1.1
APCS 1	NM_001639.2	17.9	16.8
APCS 2	NM_001639.2	7.0	5.2
CASR 1	NM_000388.2	18.2	4.0
CASR 2	NM_000388.2	10.8	4.2
CCR4 1	NM_005508.4	17.3	16.1
CCR4 2	NM_005508.4	20.0	15.0
CD4 1	NM_000616.3	15.1	10.3
CD4 2	NM_000616.3	10.1	3.7
DDEF2 1	NM_003887.1	19.7	16.7
DDEF2 2	NM_003887.1	10.9	7.6
FGFR1 1	NM_023106.2	6.4	6.4
FGFR1 2	NM_023106.2	6.7	3.3
GABRE 1	NM_004961.3	13.1	5.5
GABRE 2	NM_004961.3	15.5	7.7
HNRNPL 1	NM_001005335.1	15.3	6.9
HNRNPL 2	NM_001005335.1	16.0	7.0
IL1RN 1	NM_173842.1	21.6	6.9
IL1RN 2	NM_173842.1	10.1	7.4
IL8RB 1	NM_001557.2	13.3	11.1
IL8RB 2	NM_001557.2	7.1	4.3
MAML1 1	XM_937023.1	13.1	7.2
MAML1 2	XM_937023.1	10.4	6.9
MAPK7 1	NM_002749.2	9.6	7.3
MAPK7 2	NM_002749.2	9.8	4.1
PDE4B 1	NM_001037340.1	16.3	10.1
PDE4B 2	NM_001037340.1	16.1	11.7
PIGG 1	NM_017733.2	13.2	5.7
PIGG 2	|NM_017733.2	16.0	6.2
PPP2R2C 1	NM_020416.3	12.2	4.5
PPP2R2C 2	NM_020416.3	18.6	4.5
PRKCE 1	NM_005400.2	16.7	9.0
PRKCE 2	NM_005400.2	12.2	9.6
SSTR5 1	NM_001053.1	9.2	7.4
SSTR5 2	NM_001053.1	5.6	1.7
TNFSF9 1	NM_003811.2	20.0	6.5
TNFSF9 2	NM_003811.2	9.6	4.3
WASF1 1	NM_001024936.1	14.2	11.4
WASF1 2	NM_001024936.1	12.4	11.4
WNT7B 1	NM_058238.1	10.0	6.2
WNT7B 2	NM_058238.1	18.7	9.8

Supplemental Table2.

Komarov et al. Functional genetics-directed identification of novel pharmacological inhibitors of FAS- and TNF-dependent apoptosis that protect mice from acute liver failure

Supplemental Table 3:

DR shRNA screen-enriched pathways

Supplemental Table 3: DR shRNA screen-enriched pathways

Target Modules	
**Enriched Pathways**	**Targets in Pathway Module**
Molecular Mechanisms of Cancer	PRKAR2B,JAK3,PRKAG2,MAP2K1,FADD,ELK1,PAK3,CFLAR,PRKACG,ATR,PRKCE,JAK2,CDKN2C,CTNNB1,RAF1,SMAD2,FZD10,RASGRF2,RAP1B,GNAT1,FAS,BID,JUN,GNAI1,MAPK14,AKT1,WNT5A,GNA12,TAB2,RAC2,CBL,TP53,GNAI2,CDKN1A,GRB2,CREBBP,GNA14,ADCY2,CASP7,MAPK3,PLCB1,NFKB1,CDK7,NOTCH1,GNAT2,WNT7B,RELA,GNAS,HIF1A,BCL2,BMP1,MAP2K2,HRAS,CASP8,PRKCG
Acute Phase Response Signaling	JUN,TRAF2,MAPK14,C4BPB,AKT1,C3,TNFRSF1A,MAP2K1,C1R,PLG,ELK1,STAT3,FN1,IL1RN,IL1R1,GRB2,F2,MAPK3,TRAF6,ALB,IRAK1,NFKB1,JAK2,RAF1,TRADD,RELA,TNFRSF11B,AGT,APCS,NGFR,HRG,MAP2K2,APOA1,AHSG,HRAS
Role of Macrophages, Fibroblasts and Endothelial Cells in Rheumatoid Arthritis	JUN,IL1RL1,CXCL12,TRAF2,MAPK14,AKT1,CALM1 (includes others),NFATC2,TNFRSF1A,WNT5A,MAP2K1,TLR8,STAT3,FN1,IL1RN,IL1R1,C5AR1,CREBBP,TLR2,LTB,IL18R1,MAPK3,TRAF6,PRKCE,PLCB1,IRAK1,NFKB1,TNFSF13B,CCL5,JAK2,CTNNB1,RAF1,WNT7B,MMP13,TRADD,RELA,CXCL8,TNFRSF11B,FZD10,NGFR,MAP2K2,TLR10,HRAS,PRKCG
GNRH Signaling	JUN,GNAI1,ITPR2,MAPK14,PRKAR2B,PRKAG2,MAP2K1,DNM1,ELK1,PAK3,GNAI2,PRKACG,GRB2,MAPK7,GNA14,ADCY2,CREBBP,MAPK3,PLCB1,PRKCE,NFKB1,EGFR,RAF1,MAP3K12,EGR1,RELA,GNAS,ITPR3,MAP2K2,HRAS,PRKCG
G-Protein Coupled Receptor Signaling	GNAI1,ADORA2A,AKT1,PRKAR2B,PRKAG2,MAP2K1,PDE4B,AGTR2,STAT3,DRD2,GNAI2,PRKACG,GRB2,GNA14,ADCY2,CREBBP,CCR4,GRM5,MAPK3,PRKCE,PLCB1,NFKB1,DRD5,HTR1A,ADRB3,RAF1,ADRB1,ADRA2B,CHRM2,RELA,ADRBK1,GNAS,CXCR2,MAP2K2,ADRA2A,HRAS,ADORA1,OPRD1,PRKCG
Renin-Angiotensin Signaling	JUN,ITPR2,MAPK14,PRKAR2B,SHC2,PRKAG2,MAP2K1,AGTR2,ELK1,PAK3,STAT3,PRKACG,GRB2,ADCY2,PTPN6,MAPK3,PRKCE,NFKB1,JAK2,CCL5,RAF1,RELA,AGT,GNAS,ITPR3,MAP2K2,HRAS,PRKCG
Glucocorticoid Receptor Signaling	JUN,TRAF2,MAPK14,AKT1,NFATC2,PRKAA1,JAK3,PRKAG2,MAP2K1,YWHAH,ELK1,STAT3,IL1RN,HSPA8,STAT5A,PRKACG,CDKN1A,GRB2,CREBBP,ESR1,TAF1,MAPK3,TRAF6,NFKB1,JAK2,CCL5,CDK7,RAF1,SMAD2,RELA,CXCL8,AR,AGT,GTF2F2,BCL2,MAP2K2,HSP90AB1,CD247,HRAS
Gap Junction Signaling	GNAI1,ITPR2,AKT1,TUBA8,PRKAR2B,PRKG2,CSNK1G2,PRKAG2,MAP2K1,TUBB2A,DRD2,GNAI2,PRKACG,GRB2,MAPK7,ADCY2,CSNK1G1,MAPK3,PLCB1,PRKCE,EGFR,CTNNB1,RAF1,ADRB1,SP1,GNAS,ITPR3,MAP2K2,SP3,ACTA1,HRAS,PRKCG
PPAR Signaling	JUN,IL1RL1,TRAF2,TNFRSF1A,PPARA,MAP2K1,PPARG,IL1RN,IL1R1,STAT5A,GRB2,RXRA,CREBBP,MAPK3,TRAF6,NFKB1,PDGFRB,RAF1,RELA,TNFRSF11B,PPARD,NGFR,MAP2K2,HSP90AB1,HRAS
PPARα/RXRα Activation	JUN,IL1RL1,MAPK14,PRKAR2B,PRKAA1,PPARA,PRKAG2,MAP2K1,CYP2C9,IL1R1,PRKACG,GRB2,RXRA,GNA14,ADCY2,CREBBP,MAPK3,TRAF6,PLCB1,NFKB1,JAK2,RAF1,SMAD2,RELA,ACVR1B,GNAS,MAP2K2,APOA1,HSP90AB1,HRAS,ACVR1
CREB Signaling in Neurons	GNAI1,ITPR2,AKT1,CALM1 (includes others),PRKAR2B,GNA12,GNB1,PRKAG2,MAP2K1,ELK1,GNAI2,PRKACG,GRIN2D,GRB2,GNA14,ADCY2,CREBBP,GRM5,MAPK3,PLCB1,PRKCE,GRIA1,GNAT2,RAF1,GNAS,ITPR3,MAP2K2,GNAT1,HRAS,PRKCG
NF-κB Signaling	FGFR1,TRAF2,AKT1,TNFRSF1A,TAB2,FADD,TLR8,IL1RN,IL1R1,PRKACG,CREBBP,FGFR4,TLR2,TRAF6,IRAK1,TANK,NFKB1,TNFSF13B,EGFR,PDGFRB,RAF1,UBE2V1,TRADD,RELA,TNFRSF11B,NGFR,TLR10,HRAS,CASP8
IL-15 Signaling	TRAF2,NFKB1,MAPK14,JAK2,AKT1,RAF1,JAK3,IL15RA,MAP2K1,IL2RG,RELA,CXCL8,STAT3,STAT5A,BCL2,STAT6,MAP2K2,HRAS,AXL,MAPK3
Colorectal Cancer Metastasis Signaling	JUN,AKT1,TNFRSF1A,PRKAR2B,WNT5A,JAK3,GNB1,PRKAG2,MAP2K1,TP53,ARRB1,TLR8,STAT3,PRKACG,GRB2,ADCY2,TLR2,MAPK3,NFKB1,EGFR,JAK2,CTNNB1,WNT7B,SMAD2,MLH1,MMP13,RELA,ADRBK1,GNAS,FZD10,MAP2K2,TLR10,HRAS
IL-6 Signaling	JUN,IL1RL1,TRAF2,NFKB1,MAPK14,JAK2,AKT1,TNFRSF1A,CD14,RAF1,MAP2K1,ELK1,RELA,CXCL8,TNFRSF11B,STAT3,IL1RN,IL1R1,NGFR,MAP2K2,GRB2,HRAS,TRAF6,MAPK3
Hepatic Fibrosis / Hepatic Stellate Cell Activation	CCR7,IL1RL1,FGFR1,COL7A1,EDNRA,TNFRSF1A,TNFSF9,COL5A3,TNFSF13,IFNAR1,IL10RA,FN1,IL1R1,LTB,NFKB1,TNFSF13B,PDGFRB,EGFR,CCL5,CD14,SMAD2,MMP13,RELA,CXCL8,COL4A6,AGT,TNFRSF11B,NGFR,BCL2,FAS
Hepatic Cholestasis	JUN,IL1RL1,TRAF2,RARA,TNFRSF1A,PRKAR2B,PPARA,PRKAG2,IL1RN,IL1R1,PRKACG,RXRA,ADCY2,FGFR4,ESR1,TRAF6,PRKCE,IRAK1,NFKB1,CD14,RELA,CXCL8,TNFRSF11B,GNAS,NGFR,CNTF,CYP7A1,PRKCG

Supplemental Table 3: DR shRNA screen-enriched pathways

Role of NFAT in Regulation of the Immune Response	JUN,GNAI1,ITPR2,AKT1,CALM1 (includes others),NFATC2,HLA-DQA1,GNA12,GNB1,CSNK1G2,MAP2K1,GNAI2,GRB2,CD4,GNA14,CSNK1G1,MAPK3,PLCB1,NFKB1,GNAT2,RAF1,RELA,GNAS,ITPR3,MAP2K2,GNAT1,CD247,HRAS
Cardiac Hypertrophy Signaling	JUN,GNAI1,MAPK14,AKT1,CALM1 (includes others),PRKAR2B,GNA12,GNB1,PRKAG2,MAP2K1,ELK1,GNAI2,PRKACG,GRB2,GNA14,ADCY2,CREBBP,EIF2B2,MAPK3,PLCB1,ADRB3,GNAT2,RAF1,ADRB1,MAP3K12,ADRA2B,GNAS,MAP2K2,GNAT1,ADRA2A,HRAS
CXCR4 Signaling	JUN,GNAI1,CXCL12,ITPR2,AKT1,GNA12,GNB1,MAP2K1,ELK1,PAK3,GNAI2,CD4,GNA14,ADCY2,MAPK3,PLCB1,PRKCE,GNAT2,RAF1,EGR1,GNAS,ITPR3,MAP2K2,GNAT1,HRAS,PRKCG
cAMP-mediated signaling	GNAI1,ADORA2A,CALM1 (includes others),PRKAR2B,MAP2K1,PDE4B,AGTR2,STAT3,DRD2,GNAI2,PRKACG,ADCY2,CREBBP,CCR4,MAPK3,DRD5,HTR1A,ADRB3,RAF1,ADRB1,ADRA2B,CHRM2,ADRBK1,GNAS,CXCR2,CNGA3,MAP2K2,ADRA2A,ADORA1,OPRD1
Gαi Signaling	GNAI1,HTR1A,PRKAR2B,RAF1,GNB1,ADRA2B,PRKAG2,CHRM2,AGTR2,STAT3,DRD2,GNAI2,GNAS,CXCR2,PRKACG,GRB2,ADCY2,CCR4,ADRA2A,HRAS,OPRD1,ADORA1,MAPK3
Thrombin Signaling	GNAI1,ITPR2,MAPK14,AKT1,GNA12,GNB1,MAP2K1,ELK1,GATA3,GNAI2,GRB2,GNA14,ADCY2,F2,MAPK3,PLCB1,PRKCE,NFKB1,EGFR,GNAT2,RAF1,RELA,GNAS,ITPR3,MAP2K2,GNAT1,HRAS,PRKCG
Breast Cancer Regulation by Stathmin1	GNAI1,ITPR2,CALM1 (includes others),TUBA8,PRKAR2B,GNB1,PRKAG2,MAP2K1,TP53,TUBB2A,GNAI2,PPP2R5A,PRKACG,CDKN1A,GRB2,ADCY2,MAPK3,PLCB1,PRKCE,RAF1,PPP1CA,PPP2R2C,GNAS,ITPR3,MAP2K2,HRAS,STMN1,PRKCG
Regulation of the Epithelial-Mesenchymal Transition Pathway	FGFR1,AKT1,WNT5A,JAK3,MAP2K1,STAT3,FRS2,GRB2,FGFR4,DVL3,MAPK3,NFKB1,EGFR,PDGFRB,JAK2,NOTCH1,CTNNB1,RAF1,WNT7B,SMAD2,EGR1,RELA,FZD10,HIF1A,MAML1,MAP2K2,HRAS
Production of Nitric Oxide and Reactive Oxygen Species in Macrophages	JUN,MAPK14,AKT1,TNFRSF1A,JAK3,PPARA,MAP2K1,PPP2R5A,CREBBP,PTPN6,TLR2,MAPK3,ALB,PRKCE,NFKB1,JAK2,PPP1CA,MAP3K12,SPI1,PPP2R2C,RELA,TNFRSF11B,NGFR,RAP1B,APOA1,APOE,PRKCG
Endothelin-1 Signaling	JUN,GNAI1,PLA2G2D,ITPR2,MAPK14,EDNRA,GNA12,SHC2,PLD1,GNAI2,GRB2,MAPK7,GNA14,ADCY2,CASP7,MAPK3,PLCB1,PRKCE,GNAT2,RAF1,GNAS,ITPR3,LCAT,GNAT1,HRAS,CASP8,PRKCG
Erythropoietin Signaling	JUN,PRKCE,NFKB1,JAK2,AKT1,RAF1,MAP2K1,CBL,EPOR,ELK1,RELA,STAT5A,MAP2K2,GRB2,PTPN6,HRAS,PRKCG,MAPK3
IL-1 Signaling	GNAI1,JUN,IRAK1,NFKB1,MAPK14,PRKAR2B,GNAT2,GNA12,GNB1,TAB2,PRKAG2,RELA,GNAI2,GNAS,IL1R1,PRKACG,ADCY2,GNA14,GNAT1,TRAF6

Supplemental Table 3: DR shRNA screen-enriched pathways

FAS Pathway Maps								
#	Maps	Total	pValue	Min FDR	p-value	FDR	In Data	Network Objects from Active Data
0	Apoptosis and survival_Apoptotic TNF-family pathways	33	6.780E-04	2.340E-01	6.780E-04	2.340E-01	8	GITR, Caspase-8, NGFR(TNFRSF16), FasR(CD95), Bid, tBid, TRADD, FADD
2	Muscle contraction_Role of kappa-type opioid receptor in heart	8	8.197E-04	2.340E-01	8.197E-04	2.340E-01	4	PKC-epsilon, Beta-3 adrenergic receptor, Beta-1 adrenergic receptor, G-protein alpha-s
3	Apoptosis and survival_Caspase cascade	29	1.520E-03	2.893E-01	1.520E-03	2.893E-01	7	PARP-1, Caspase-8, FasR(CD95), Bid, tBid, TRADD, FADD
4	Immune response_Classical complement pathway	32	2.797E-03	3.194E-01	2.797E-03	3.194E-01	7	C3a, C3dg, iC3b, C3, C3b, C3c, C1qRp
5	Immune response_Lectin induced complement pathway	32	2.797E-03	3.194E-01	2.797E-03	3.194E-01	7	C3a, C3dg, iC3b, C3, C3b, C3c, C1qRp
6	Apoptosis and survival_Role of IAP-proteins in apoptosis	26	4.229E-03	3.450E-01	4.229E-03	3.450E-01	6	Caspase-8, FasR(CD95), Bid, tBid, TRADD, FADD
7	Apoptosis and survival_Cytoplasmic/mitochondrial transport of proapoptotic proteins Bid, Bmf and Bim	26	4.229E-03	3.450E-01	4.229E-03	3.450E-01	6	Caspase-8, FasR(CD95), Bid, tBid, TRADD, FADD
8	Development_Beta adrenergic receptors in brown adipocyte differentiation	27	5.162E-03	3.528E-01	5.162E-03	3.528E-01	6	FASN, A-FABP, Beta-3 adrenergic receptor, Beta-1 adrenergic receptor, G-protein alpha-s, G3P2
9	Apoptosis and survival_Ceramides signaling pathway	28	6.239E-03	3.528E-01	6.239E-03	3.528E-01	6	Caspase-8, FasR(CD95), Bid, tBid, TRADD, FADD
10	Apoptosis and survival_TNF-alpha-induced Caspase-8 signaling	28	6.239E-03	3.528E-01	6.239E-03	3.528E-01	6	Caspase-8, Cathepsin B, Bid, tBid, TRADD, FADD
11	Beta-adrenergic-dependent CFTR expression	7	6.797E-03	3.528E-01	6.797E-03	3.528E-01	3	Beta-3 adrenergic receptor, Beta-1 adrenergic receptor, G-protein alpha-s
12	Immune response_Alternative complement pathway	30	8.867E-03	3.975E-01	8.867E-03	3.975E-01	6	C3a, C3dg, iC3b, C3, C3b, C3c
13	Phospholipid metabolism p.2	8	1.038E-02	3.975E-01	1.038E-02	3.975E-01	3	PLD1, LPP1, CHKA
14	Glutathione metabolism / Human version	31	1.044E-02	3.975E-01	1.044E-02	3.975E-01	6	GSTM5, GSTA5, GSTM1, GCL reg, GSTA3, GSTK1
15	Glutathione metabolism	31	1.044E-02	3.975E-01	1.044E-02	3.975E-01	6	GSTM5, GSTA5, GSTM1, GCL reg, GSTA3, GSTK1
16	Glutathione metabolism / Rodent version	32	1.221E-02	4.356E-01	1.221E-02	4.356E-01	6	GSTM5, GSTA5, GSTM1, GCL reg, GSTA3, GSTK1
17	Apoptosis and survival_TNFR1 signaling pathway	35	1.874E-02	6.294E-01	1.874E-02	6.294E-01	6	Caspase-8, jBid, Bid, tBid, TRADD, FADD
18	Cytoskeleton remodeling_ESR1 action on cytoskeleton remodeling and cell migration	10	2.029E-02	6.421E-01	2.029E-02	6.421E-01	3	ESR1 (membrane), ESR1 (nuclear), EBP50
19	Apoptosis and survival_FAS signaling cascades	36	2.136E-02	6.421E-01	2.136E-02	6.421E-01	6	PARP-1, Caspase-8, FasR(CD95), Bid, tBid, FADD
20	Apoptosis and survival_Granzyme B signaling	27	2.285E-02	6.523E-01	2.285E-02	6.523E-01	5	PARP-1, Caspase-8, Bid, tBid, FGFR1
21	Immune response_T cell subsets: cell surface markers	28	2.646E-02	7.194E-01	2.646E-02	7.194E-01	5	IL-15RA, GITR, CD4, CCR4, CCR6
22	Regulation of lipid metabolism_Regulation of lipid metabolism by niacin and isoprenaline	20	3.157E-02	7.695E-01	3.157E-02	7.695E-01	4	Adenylate cyclase type II, Beta-3 adrenergic receptor, Beta-1 adrenergic receptor, G-protein alpha-s
23	Immune response_IL-15 signaling via JAK-STAT cascade	20	3.157E-02	7.695E-01	3.157E-02	7.695E-01	4	sIL-15RA, IL-15RA, JAK3, JAK2
24	DeltaF508-CFTR traffic / ER-to-Golgi in CF	5	3.369E-02	7.695E-01	3.369E-02	7.695E-01	2	PIST (CAL), EBP50
25	wtCFTR traffic / ER-to-Golgi (normal)	5	3.369E-02	7.695E-01	3.369E-02	7.695E-01	2	PIST (CAL), EBP50
26	Immune response_Innate immune response to RNA viral infection	21	3.717E-02	8.163E-01	3.717E-02	8.163E-01	4	Caspase-8, TANK, TRADD, FADD
27	Immune response_Th17, Th22 and Th9 cell differentiation	31	3.940E-02	8.331E-01	3.940E-02	8.331E-01	5	GATA-3, IL-9, CD4, PU.1, IL-22
28	Immune response_IL-22 signaling pathway	22	4.330E-02	8.748E-01	4.330E-02	8.748E-01	4	JAK3, CD4, JAK2, IL-22

Supplemental Table 3: DR shRNA screen-enriched pathways

29	Regulation of AKT(PKB)/ GSK3 beta cascade in bipolar disorder	32	4.443E-02	8.748E-01	4.443E-02	8.748E-01	5	ESR1 (membrane), HTR1A, FGFR1, Epo receptor, G-protein alpha-i2
30	Development_Hedgehog and PTH signaling pathways in bone and cartilage development	23	4.997E-02	9.213E-01	4.997E-02	9.213E-01	4	Bone sialoprotein, Parathyroid hormone, G-protein alpha-s, PTHR1
31	Cytoskeleton remodeling_Thyroliberin in cytoskeleton remodeling	14	5.129E-02	9.213E-01	5.129E-02	9.213E-01	3	PKC-epsilon, G-protein alpha-s, G-protein alpha-i2
32	Development_EPO-induced PI3K/AKT pathway and Ca(2+) influx	24	5.716E-02	9.213E-01	5.716E-02	9.213E-01	4	IP3R2, JAK2, Epo receptor, G-protein alpha-i2
33	DNA damage_Inhibition of telomerase activity and cellular senescence	15	6.128E-02	9.213E-01	6.128E-02	9.213E-01	3	p130, p21, p107
34	Mechanism of action of CCR4 antagonists in asthma and atopic dermatitis (Variant 2)	1	6.192E-02	9.213E-01	6.192E-02	9.213E-01	1	CCR4
35	Immune response_MIF - the neuroendocrine-macrophage connector	16	7.210E-02	9.213E-01	7.210E-02	9.213E-01	3	Adenylate cyclase type II, PU.1, G-protein alpha-s
36	Development_MAG-dependent inhibition of neurite outgrowth	16	7.210E-02	9.213E-01	7.210E-02	9.213E-01	3	NGFR (CTF), NGFR(TNFRSF16), NGFR (ICD)
37	Immune response_Th17 cell differentiation	26	7.313E-02	9.213E-01	7.313E-02	9.213E-01	4	JAK3, CD4, JAK2, IL-22
38	Immune response_IL-9 signaling pathway	26	7.313E-02	9.213E-01	7.313E-02	9.213E-01	4	IL-9, JAK3, IL-22, TRADD
39	Regulation of CFTR activity (normal and CF)	26	7.313E-02	9.213E-01	7.313E-02	9.213E-01	4	PKC-epsilon, Protein kinase G 2, G-protein alpha-s, EBP50
40	NAD metabolism	48	7.318E-02	9.213E-01	7.318E-02	9.213E-01	6	VPARP, PARP-1, 5'-NTD, PLAP-like, TDO2, ALPP
41	Development_Beta-adrenergic receptors signaling via cAMP	27	8.187E-02	9.213E-01	8.187E-02	9.213E-01	4	BETA-PIX, Beta-3 adrenergic receptor, Beta-1 adrenergic receptor, G-protein alpha-s
42	Apoptosis and survival_Regulation of Apoptosis by Mitochondrial Proteins	27	8.187E-02	9.213E-01	8.187E-02	9.213E-01	4	Caspase-8, FasR(CD95), Bid, tBid
43	Immune response_Differentiation of natural regulatory T cells	27	8.187E-02	9.213E-01	8.187E-02	9.213E-01	4	GITR, JAK3, CD4, JAK2
44	Amitraz-induced inhibition of Insulin secretion	8	8.345E-02	9.213E-01	8.345E-02	9.213E-01	2	Alpha-2A adrenergic receptor, G-protein alpha-i2
45	wtCFTR and deltaF508-CFTR traffic / Generic schema (normal and CF)	17	8.371E-02	9.213E-01	8.371E-02	9.213E-01	3	PIST (CAL), DAB2, EBP50
46	Development_Delta-type opioid receptor signaling via G-protein alpha-14	17	8.371E-02	9.213E-01	8.371E-02	9.213E-01	3	PKC-epsilon, Delta-type opioid receptor, JAK2
47	Immune response_Naive CD4+ T cell differentiation	39	8.999E-02	9.213E-01	8.999E-02	9.213E-01	5	GATA-3, IL-9, CD4, PU.1, IL-22
48	Immune response_Th1 and Th2 cell differentiation	29	1.008E-01	9.213E-01	1.008E-01	9.213E-01	4	GATA-3, CD4, JAK2, CD137 ligand(TNFSF9)
49	Transcription_Role of AP-1 in regulation of cellular metabolism	29	1.008E-01	9.213E-01	1.008E-01	9.213E-01	4	p21, FasR(CD95), GCL reg, FXYD2
50	Beta-alanine metabolism/ Rodent version	9	1.030E-01	9.213E-01	1.030E-01	9.213E-01	2	GAD2, CPGL2

Supplemental Table 3: DR shRNA screen-enriched pathways

TNF Pathway Maps								
#	Maps	Total	pValue	Min FDR	p-value	FDR	In Data	Network Objects from Active Data
1	Immune response_Alternative complement pathway	30	1.521E-03	1.393E-01	1.521E-03	1.393E-01	6	C3a, C3dg, iC3b, C3, C3b, C3c
2	Glutathione metabolism / Human version	31	1.819E-03	1.393E-01	1.819E-03	1.393E-01	6	GSTM5, GSTA5, GSTM1, GCL reg, GSTA3, GSTK1
3	Glutathione metabolism	31	1.819E-03	1.393E-01	1.819E-03	1.393E-01	6	GSTM5, GSTA5, GSTM1, GCL reg, GSTA3, GSTK1
4	Immune response_Lectin induced complement pathway	32	2.160E-03	1.393E-01	2.160E-03	1.393E-01	6	C3a, C3dg, iC3b, C3, C3b, C3c
5	Immune response_Classical complement pathway	32	2.160E-03	1.393E-01	2.160E-03	1.393E-01	6	C3a, C3dg, iC3b, C3, C3b, C3c
6	Glutathione metabolism / Rodent version	32	2.160E-03	1.393E-01	2.160E-03	1.393E-01	6	GSTM5, GSTA5, GSTM1, GCL reg, GSTA3, GSTK1
7	Apoptosis and survival_Apoptotic TNF-family pathways	33	2.547E-03	1.408E-01	2.547E-03	1.408E-01	6	GITR, Bid, tBid, Caspase-8, NGFR(TNFRSF16), FADD
8	Apoptosis and survival_TNF-alpha-induced Caspase-8 signaling	28	6.340E-03	3.067E-01	6.340E-03	3.067E-01	5	Cathepsin B, Bid, tBid, Caspase-8, FADD
9	Apoptosis and survival_TNFR1 signaling pathway	35	1.642E-02	6.479E-01	1.642E-02	6.479E-01	5	jBid, Bid, tBid, Caspase-8, FADD
10	Glycine, serine, cysteine and threonine metabolism/ Rodent version	36	1.842E-02	6.479E-01	1.842E-02	6.479E-01	5	CHKA, BHMT, KBL, GAMT, BHMT2
11	Glycine, serine, cysteine and threonine metabolism	36	1.842E-02	6.479E-01	1.842E-02	6.479E-01	5	CHKA, BHMT, KBL, GAMT, BHMT2
12	Apoptosis and survival_Cytoplasmic/mitochondrial transport of proapoptotic proteins Bid, Bmf and Bim	26	2.433E-02	7.096E-01	2.433E-02	7.096E-01	4	Bid, tBid, Caspase-8, FADD
13	Apoptosis and survival_Role of IAP-proteins in apoptosis	26	2.433E-02	7.096E-01	2.433E-02	7.096E-01	4	Bid, tBid, Caspase-8, FADD
14	Apoptosis and survival_Granzyme B signaling	27	2.762E-02	7.096E-01	2.762E-02	7.096E-01	4	Bid, tBid, FGFR1, Caspase-8
15	Development_MAG-dependent inhibition of neurite outgrowth	16	2.976E-02	7.096E-01	2.976E-02	7.096E-01	3	NGFR (CTF), NGFR(TNFRSF16), NGFR (ICD)
16	Apoptosis and survival_Ceramides signaling pathway	28	3.117E-02	7.096E-01	3.117E-02	7.096E-01	4	Bid, tBid, Caspase-8, FADD
17	Immune response_T cell subsets: cell surface markers	28	3.117E-02	7.096E-01	3.117E-02	7.096E-01	4	CD4, GITR, CCR4, CCR6
18	Apoptosis and survival_Caspase cascade	29	3.497E-02	7.520E-01	3.497E-02	7.520E-01	4	Bid, tBid, Caspase-8, FADD
19	Mechanism of action of CCR4 antagonists in asthma and atopic dermatitis (Variant 2)	1	4.351E-02	8.343E-01	4.351E-02	8.343E-01	1	CCR4
20	Phospholipid metabolism p.2	8	4.429E-02	8.343E-01	4.429E-02	8.343E-01	2	CHKA, PLD1
21	G-protein signaling_RAC1 in cellular process	19	4.685E-02	8.343E-01	4.685E-02	8.343E-01	3	PLD1, PAK3, WASF1(WAVE1)
22	Beta-alanine metabolism/ Rodent version	9	5.535E-02	8.343E-01	5.535E-02	8.343E-01	2	GAD2, CPGL2
23	Immune response_Innate immune response to RNA viral infection	21	6.040E-02	8.343E-01	6.040E-02	8.343E-01	3	Caspase-8, TANK, FADD
24	Transcription_Transcription regulation of aminoacid metabolism	21	6.040E-02	8.343E-01	6.040E-02	8.343E-01	3	GCL reg, NFE2L1, NF-E2 (45 kDa)
25	Beta-alanine metabolism	10	6.725E-02	8.343E-01	6.725E-02	8.343E-01	2	GAD2, CPGL2
26	Apoptosis and survival_FAS signaling cascades	36	6.882E-02	8.343E-01	6.882E-02	8.343E-01	4	Bid, tBid, Caspase-8, FADD
27	Oxidative stress_Role of Sirtuin1 and PGC1-alpha in activation of antioxidant defense system	37	7.466E-02	8.343E-01	7.466E-02	8.343E-01	4	GSTM5, GSTA5, GCL reg, MT-TRX
28	Development_Hedgehog and PTH signaling pathways in bone and cartilage development	23	7.556E-02	8.343E-01	7.556E-02	8.343E-01	3	Parathyroid hormone, PTHR1, Bone sialoprotein
29	LRRK2 in neuronal apoptosis in Parkinson's disease	11	7.991E-02	8.343E-01	7.991E-02	8.343E-01	2	Caspase-8, FADD

Supplemental Table 3: DR shRNA screen-enriched pathways

30	Colorectal cancer (general schema)	24	8.372E-02	8.343E-01	8.372E-02	8.343E-01	3	IL8RB, FZD2, WNT5A
31	Development_Regulation of lung epithelial progenitor cell differentiation	24	8.372E-02	8.343E-01	8.372E-02	8.343E-01	3	FZD2, p130, FOXP1
32	Mechanism of Maraviroc action in HIV Infections	2	8.513E-02	8.343E-01	8.513E-02	8.343E-01	1	CD4
33	Mitochondrial unsaturated fatty acid beta-oxidation	12	9.324E-02	8.343E-01	9.324E-02	8.343E-01	2	DCI, ACADM
34	Transport_HDL-mediated reverse cholesterol transport	12	9.324E-02	8.343E-01	9.324E-02	8.343E-01	2	LCAT, APOA1
35	Methionine metabolism	13	1.072E-01	8.343E-01	1.072E-01	8.343E-01	2	BHMT, BHMT2
36	Development_FGF2-dependent induction of EMT	13	1.072E-01	8.343E-01	1.072E-01	8.343E-01	2	FRS2, FGFR1
37	Development_Beta adrenergic receptors in brown adipocyte differentiation	27	1.103E-01	8.343E-01	1.103E-01	8.343E-01	3	G3P2, A-FABP, Beta-3 adrenergic receptor
38	Apoptosis and survival_Regulation of Apoptosis by Mitochondrial Proteins	27	1.103E-01	8.343E-01	1.103E-01	8.343E-01	3	Bid, tBid, Caspase-8
39	Influence of low doses of Arsenite on glucose uptake in adipocytes	14	1.216E-01	8.343E-01	1.216E-01	8.343E-01	2	p21, A-FABP
40	Role of ZNF202 in regulation of expression of genes involved in atherosclerosis	14	1.216E-01	8.343E-01	1.216E-01	8.343E-01	2	PU.1, LCAT
41	Arsenite metabolism and transport	3	1.250E-01	8.343E-01	1.250E-01	8.343E-01	1	Aquaporin 7
42	Phospholipid metabolism p.3	3	1.250E-01	8.343E-01	1.250E-01	8.343E-01	1	LCAT
43	Transcription_Role of AP-1 in regulation of cellular metabolism	29	1.295E-01	8.343E-01	1.295E-01	8.343E-01	3	p21, GCL reg, FXYD2
44	Neurophysiological process_Delta-type opioid receptor in the nervous system	15	1.364E-01	8.343E-01	1.364E-01	8.343E-01	2	Adenylate cyclase type II, Delta-type opioid receptor
45	DNA damage_Inhibition of telomerase activity and cellular senescence	15	1.364E-01	8.343E-01	1.364E-01	8.343E-01	2	p21, p130
46	Development_Mu-type opioid receptor signaling via Beta-arrestin	15	1.364E-01	8.343E-01	1.364E-01	8.343E-01	2	GRK5, Delta-type opioid receptor
47	Development_Delta- and kappa-type opioid receptors signaling via beta-arrestin	15	1.364E-01	8.343E-01	1.364E-01	8.343E-01	2	GRK5, Delta-type opioid receptor
48	Development_Lipoxin inhibitory action on PDGF, EGF and LTD4 signaling	15	1.364E-01	8.343E-01	1.364E-01	8.343E-01	2	PLD1, CysLT1 receptor
49	Immune response_Th17, Th22 and Th9 cell differentiation	31	1.499E-01	8.343E-01	1.499E-01	8.343E-01	3	CD4, PU.1, GATA-3
50	Immune response_T regulatory cell-mediated modulation of	31	1.499E-01	8.343E-01	1.499E-01	8.343E-01	3	DAB2, 5'-NTD, CCR4

Supplemental Table 3: DR shRNA screen-enriched pathways

FAS and TNF Pathway Maps								
#	Maps	Total	pValue	Min FDR	p-value	FDR	In Data	Network Objects from Active Data
1	Immune response_Alternative complement pathway	30	4.744E-04	7.373E-02	4.744E-04	7.373E-02	6	C3a, C3dg, iC3b, C3, C3b, C3c
2	Immune response_Lectin induced complement pathway	32	6.839E-04	7.373E-02	6.839E-04	7.373E-02	6	C3a, C3dg, iC3b, C3, C3b, C3c
3	Immune response_Classical complement pathway	32	6.839E-04	7.373E-02	6.839E-04	7.373E-02	6	C3a, C3dg, iC3b, C3, C3b, C3c
4	Apoptosis and survival_Apoptotic TNF-family pathways	33	8.124E-04	7.373E-02	8.124E-04	7.373E-02	6	GITR, Bid, tBid, Caspase-8, NGFR(TNFRSF16), FADD
5	Apoptosis and survival_TNF-alpha-induced Caspase-8 signaling	28	2.454E-03	1.781E-01	2.454E-03	1.781E-01	5	Cathepsin B, Bid, tBid, Caspase-8, FADD
6	Apoptosis and survival_TNFR1 signaling pathway	35	6.673E-03	4.037E-01	6.673E-03	4.037E-01	5	jBid, Bid, tBid, Caspase-8, FADD
7	Apoptosis and survival_Cytoplasmic/mitochondrial transport of proapoptotic proteins Bid, Bmf and Bim	26	1.164E-02	4.765E-01	1.164E-02	4.765E-01	4	Bid, tBid, Caspase-8, FADD
8	Apoptosis and survival_Role of IAP-proteins in apoptosis	26	1.164E-02	4.765E-01	1.164E-02	4.765E-01	4	Bid, tBid, Caspase-8, FADD
9	Apoptosis and survival_Granzyme B signaling	27	1.330E-02	4.765E-01	1.330E-02	4.765E-01	4	Bid, tBid, FGFR1, Caspase-8
10	Apoptosis and survival_Ceramides signaling pathway	28	1.511E-02	4.765E-01	1.511E-02	4.765E-01	4	Bid, tBid, Caspase-8, FADD
11	Immune response_T cell subsets: cell surface markers	28	1.511E-02	4.765E-01	1.511E-02	4.765E-01	4	CD4, GITR, CCR4, CCR6
12	Development_MAG-dependent inhibition of neurite outgrowth	16	1.666E-02	4.765E-01	1.666E-02	4.765E-01	3	NGFR (CTF), NGFR(TNFRSF16), NGFR (ICD)
13	Apoptosis and survival_Caspase cascade	29	1.707E-02	4.765E-01	1.707E-02	4.765E-01	4	Bid, tBid, Caspase-8, FADD
14	G-protein signaling_RAC1 in cellular process	19	2.673E-02	6.931E-01	2.673E-02	6.931E-01	3	PLD1, PAK3, WASF1(WAVE1)
15	Immune response_Innate immune response to RNA viral infection	21	3.489E-02	7.505E-01	3.489E-02	7.505E-01	3	Caspase-8, TANK, FADD
16	Mechanism of action of CCR4 antagonists in asthma and atopic dermatitis (Variant 2)	1	3.493E-02	7.505E-01	3.493E-02	7.505E-01	1	CCR4
17	Apoptosis and survival_FAS signaling cascades	36	3.515E-02	7.505E-01	3.515E-02	7.505E-01	4	Bid, tBid, Caspase-8, FADD
18	Colorectal cancer (general schema)	24	4.925E-02	7.536E-01	4.925E-02	7.536E-01	3	IL8RB, FZD2, WNT5A
19	Development_Regulation of lung epithelial progenitor cell differentiation	24	4.925E-02	7.536E-01	4.925E-02	7.536E-01	3	FZD2, p130, FOXP1
20	LRRK2 in neuronal apoptosis in Parkinson's disease	11	5.414E-02	7.536E-01	5.414E-02	7.536E-01	2	Caspase-8, FADD
21	Mitochondrial unsaturated fatty acid beta-oxidation	12	6.352E-02	7.536E-01	6.352E-02	7.536E-01	2	DCI, ACADM
22	Transport_HDL-mediated reverse cholesterol transport	12	6.352E-02	7.536E-01	6.352E-02	7.536E-01	2	LCAT, APOA1
23	Development_Beta adrenergic receptors in brown adipocyte differentiation	27	6.605E-02	7.536E-01	6.605E-02	7.536E-01	3	G3P2, A-FABP, Beta-3 adrenergic receptor
24	Apoptosis and survival_Regulation of Apoptosis by Mitochondrial Proteins	27	6.605E-02	7.536E-01	6.605E-02	7.536E-01	3	Bid, tBid, Caspase-8
25	Mechanism of Maraviroc action in HIV Infections	2	6.865E-02	7.536E-01	6.865E-02	7.536E-01	1	CD4
26	Development_FGF2-dependent induction of EMT	13	7.340E-02	7.536E-01	7.340E-02	7.536E-01	2	FRS2, FGFR1

27	Influence of low doses of Arsenite on glucose uptake in adipocytes	14	8.373E-02	7.536E-01	8.373E-02	7.536E-01	2	p21, A-FABP
28	Role of ZNF202 in regulation of expression of genes involved in atherosclerosis	14	8.373E-02	7.536E-01	8.373E-02	7.536E-01	2	PU.1, LCAT
29	Immune response_Th17, Th22 and Th9 cell differentiation	31	9.194E-02	7.536E-01	9.194E-02	7.536E-01	3	CD4, PU.1, GATA-3
30	Immune response_T regulatory cell-mediated modulation of antigen-presenting cell functions	31	9.194E-02	7.536E-01	9.194E-02	7.536E-01	3	DAB2, 5'-NTD, CCR4
31	Neurophysiological process_Delta-type opioid receptor in the nervous system	15	9.447E-02	7.536E-01	9.447E-02	7.536E-01	2	Adenylate cyclase type II, Delta-type opioid receptor
32	DNA damage_Inhibition of telomerase activity and cellular senescence	15	9.447E-02	7.536E-01	9.447E-02	7.536E-01	2	p21, p130
33	Development_Mu-type opioid receptor signaling via Beta-arrestin	15	9.447E-02	7.536E-01	9.447E-02	7.536E-01	2	GRK5, Delta-type opioid receptor
34	Development_Delta- and kappa-type opioid receptors signaling via beta-arrestin	15	9.447E-02	7.536E-01	9.447E-02	7.536E-01	2	GRK5, Delta-type opioid receptor
35	Development_Lipoxin inhibitory action on PDGF, EGF and LTD4 signaling	15	9.447E-02	7.536E-01	9.447E-02	7.536E-01	2	PLD1, CysLT1 receptor
36	Regulation of AKT(PKB)/ GSK3 beta cascade in bipolar disorder	32	9.899E-02	7.536E-01	9.899E-02	7.536E-01	3	HTR1A, FGFR1, Epo receptor
37	Arsenite metabolism and transport	3	1.012E-01	7.536E-01	1.012E-01	7.536E-01	1	Aquaporin 7
38	Phospholipid metabolism p.3	3	1.012E-01	7.536E-01	1.012E-01	7.536E-01	1	LCAT
39	Impaired Lipoxin A4 signaling in CF	16	1.056E-01	7.536E-01	1.056E-01	7.536E-01	2	PLD1, CysLT1 receptor
40	Immune response_MIF - the neuroendocrine-macrophage connector	16	1.056E-01	7.536E-01	1.056E-01	7.536E-01	2	PU.1, Adenylate cyclase type II
41	wtCFTR and deltaF508-CFTR traffic / Generic schema (normal and CF)	17	1.170E-01	7.536E-01	1.170E-01	7.536E-01	2	DAB2, PIST (CAL)
42	Immune response_Inhibitory PD-1 signaling in T cells	36	1.292E-01	7.536E-01	1.292E-01	7.536E-01	3	CD4, GATA-3, CD8 alpha
43	Neurophysiological process_Bitter taste signaling	4	1.326E-01	7.536E-01	1.326E-01	7.536E-01	1	IP3R3
44	Neurophysiological process_Sweet taste signaling	4	1.326E-01	7.536E-01	1.326E-01	7.536E-01	1	IP3R3
45	Apoptosis and survival_Role of PKR in stress-induced apoptosis	37	1.372E-01	7.536E-01	1.372E-01	7.536E-01	3	p21, Caspase-8, PKR
46	Development_Keratinocyte differentiation	37	1.372E-01	7.536E-01	1.372E-01	7.536E-01	3	p21, TGM1, CaSR
47	Cell cycle_Role of 14-3-3 proteins in cell cycle regulation	19	1.408E-01	7.536E-01	1.408E-01	7.536E-01	2	ATR, 14-3-3 gamma
48	DNA damage_ATM / ATR regulation of G2 / M checkpoint	19	1.408E-01	7.536E-01	1.408E-01	7.536E-01	2	p21, ATR
49	Immune response_iC3b-induced phagocytosis via alpha-M/beta-2 integrin	20	1.530E-01	7.536E-01	1.530E-01	7.536E-01	2	iC3b, PLD1
50	Regulation of lipid metabolism_Regulation of lipid metabolism by niacin and isoprenaline	20	1.530E-01	7.536E-01	1.530E-01	7.536E-01	2	Adenylate cyclase type II, Beta-3 adrenergic receptor

Komarov et al. Functional genetics-directed identification of novel pharmacological inhibitors of FAS- and TNF-dependent apoptosis that protect mice from acute liver failure

Supplemental Table 4:

DR shRNA screen-enriched GO biological processes

Supplemental Table 4: DR shRNA screen-enriched GO biological processes

FAS GO Processes								
#	Processes	Total	pValue	Min FDR	p-value	FDR	In Data	Network Objects from Active Data
1	vagina development	12	9.629E-07	2.193E-03	9.629E-07	2.193E-03	7	ESR1 (mitochondrial), ESR1 (membrane), ESR1 (nuclear), WNT5A, ESR2 (mitochondrial), ESR2 (membrane), ESR2
2	Sertoli cell development	12	9.629E-07	2.193E-03	9.629E-07	2.193E-03	7	ESR1 (mitochondrial), ESR1 (membrane), ESR1 (nuclear), SOX9, ESR2 (mitochondrial), ESR2 (membrane), ESR2
3	intracellular estrogen receptor signaling pathway	9	2.067E-06	3.138E-03	2.067E-06	3.138E-03	6	ESR1 (mitochondrial), ESR1 (membrane), ESR1 (nuclear), ESR2 (mitochondrial), ESR2 (membrane), ESR2
4	uterus development	19	3.711E-06	4.226E-03	3.711E-06	4.226E-03	8	GATA-3, ESR1 (mitochondrial), ESR1 (membrane), ESR1 (nuclear), WNT5A, ESR2 (mitochondrial), ESR2 (membrane), ESR2
5	Sertoli cell proliferation	11	1.033E-05	7.453E-03	1.033E-05	7.453E-03	6	ESR1 (mitochondrial), ESR1 (membrane), ESR1 (nuclear), ESR2 (mitochondrial), ESR2 (membrane), ESR2
6	female genitalia development	16	1.145E-05	7.453E-03	1.145E-05	7.453E-03	7	ESR1 (mitochondrial), ESR1 (membrane), ESR1 (nuclear), WNT5A, ESR2 (mitochondrial), ESR2 (membrane), ESR2
7	Sertoli cell differentiation	16	1.145E-05	7.453E-03	1.145E-05	7.453E-03	7	ESR1 (mitochondrial), ESR1 (membrane), ESR1 (nuclear), SOX9, ESR2 (mitochondrial), ESR2 (membrane), ESR2
8	response to genistein	5	4.507E-05	2.567E-02	4.507E-05	2.567E-02	4	GSTM1, ESR2 (mitochondrial), ESR2 (membrane), ESR2
9	cellular response to estradiol stimulus	20	6.393E-05	3.236E-02	6.393E-05	3.236E-02	7	ESR1 (mitochondrial), ESR1 (membrane), ESR1 (nuclear), HSD11B1, ESR2 (mitochondrial), ESR2 (membrane), ESR2
10	positive regulation of epidermal growth factor receptor signaling pathway	21	9.136E-05	4.065E-02	9.136E-05	4.065E-02	7	ESR1 (mitochondrial), ESR1 (membrane), ESR1 (nuclear), Alpha-2A adrenergic receptor, ESR2 (mitochondrial), ESR2 (membrane), ESR2
11	prostate gland epithelium morphogenesis	28	9.813E-05	4.065E-02	9.813E-05	4.065E-02	8	ESR1 (mitochondrial), ESR1 (membrane), ESR1 (nuclear), SOX9, FRS2, ESR2 (mitochondrial), ESR2 (membrane), ESR2
12	positive regulation of ERBB signaling pathway	22	1.277E-04	4.207E-02	1.277E-04	4.207E-02	7	ESR1 (mitochondrial), ESR1 (membrane), ESR1 (nuclear), Alpha-2A adrenergic receptor, ESR2 (mitochondrial), ESR2 (membrane), ESR2
13	prostate gland morphogenesis	29	1.290E-04	4.207E-02	1.290E-04	4.207E-02	8	ESR1 (mitochondrial), ESR1 (membrane), ESR1 (nuclear), SOX9, FRS2, ESR2 (mitochondrial), ESR2 (membrane), ESR2
14	prostate gland morphogenetic growth	6	1.293E-04	4.207E-02	1.293E-04	4.207E-02	4	ESR1 (mitochondrial), ESR1 (membrane), ESR1 (nuclear), SOX9
15	cellular response to magnetism	3	1.710E-04	5.193E-02	1.710E-04	5.193E-02	3	ESR2 (mitochondrial), ESR2 (membrane), ESR2
16	intracellular steroid hormone receptor signaling pathway	47	2.020E-04	5.655E-02	2.020E-04	5.655E-02	10	ESR1 (mitochondrial), ESR1 (membrane), ESR1 (nuclear), LPP1, JAK2, CAR, 14-3-3 eta, ESR2 (mitochondrial), ESR2 (membrane), ESR2
17	regulation of response to cytokine stimulus	75	2.110E-04	5.655E-02	2.110E-04	5.655E-02	13	RIPK2, LXR-beta, ERK5 (MAPK7), IFNA1, IFNA2, IFNA16, IL1RN, Gas6, JAK2, WNT5A, FADD, IFNA21, APOA1
18	hormone-mediated apoptotic signaling pathway	7	2.885E-04	6.571E-02	2.885E-04	6.571E-02	4	Parathyroid hormone, ESR2 (mitochondrial), ESR2 (membrane), ESR2
19	response to hydroxyisoflavone	7	2.885E-04	6.571E-02	2.885E-04	6.571E-02	4	GSTM1, ESR2 (mitochondrial), ESR2 (membrane), ESR2
20	epithelial cell proliferation involved in mammary gland duct elongation	7	2.885E-04	6.571E-02	2.885E-04	6.571E-02	4	ESR1 (mitochondrial), ESR1 (membrane), ESR1 (nuclear), WNT5A
21	cellular response to estrogen stimulus	25	3.114E-04	6.644E-02	3.114E-04	6.644E-02	7	ESR1 (mitochondrial), ESR1 (membrane), ESR1 (nuclear), HSD11B1, ESR2 (mitochondrial), ESR2 (membrane), ESR2

Supplemental Table 4: DR shRNA screen-enriched GO biological processes

22	response to organic cyclic compound	793	3.208E-04	6.644E-02	3.208E-04	6.644E-02	66	C3, TPH2, IP3R3, CYP2C9, RIPK2, GATA-3, LXR-beta, GSTA5, Osteoprotegerin, Alpha 1-antitrypsin, ESR1 (mitochondrial), ESR1 (membrane), ESR1 (nuclear), FZD2, PKC-epsilon, HNMT, Caspase-8, PKC-gamma, MT-TRX, BChE, A-FABP, LDHA, CYP7A1, IFNA1, ALK-2, Cathepsin B, p21, IP3R2, HSD17B3, DAB2, IFNA16, IL1RN, WNT7B, Parathyroid hormone, PDE4B, LCAT, GNAS1, G-protein alpha-s, NOR1, FasR(CD95), ACTA1, CAR, Bid, ACADM, SSTR5, HSD11B1, GSTM1, CNGA3, WNT5A, NRF1, FosB, PKR, IL-22, ATP12A, HEM2, nAChR alpha-3, Activin beta B, HLA-DQA1, DHFR, RAP-1B, CaSR, IFNA21, APOA1, ESR2 (mitochondrial), ESR2 (membrane), ESR2
23	positive regulation of phospholipase C activity	60	3.910E-04	7.554E-02	3.910E-04	7.554E-02	11	IP3R3, ESR1 (mitochondrial), ESR1 (membrane), ESR1 (nuclear), PKC-epsilon, PKC-gamma, Adenylate cyclase type II, Fc epsilon RI beta, IP3R2, FGFR1, PKA-cat gamma
24	prostate gland development	42	3.979E-04	7.554E-02	3.979E-04	7.554E-02	9	ESR1 (mitochondrial), ESR1 (membrane), ESR1 (nuclear), SOX9, FRS2, WNT5A, ESR2 (mitochondrial), ESR2 (membrane), ESR2
25	adenylate cyclase-modulating G-protein coupled receptor signaling pathway	112	4.157E-04	7.577E-02	4.157E-04	7.577E-02	16	Transducin, rod-specific, GRK5, Adenylate cyclase type II, Alpha-2A adrenergic receptor, Delta-type opioid receptor, Beta-3 adrenergic receptor, Beta-1 adrenergic receptor, Parathyroid hormone, HTR1A, GNAS1, G-protein alpha-s, PTHR1, SSTR5, G-protein alpha-i2, EBP50, CaSR
26	regulation of phospholipase C activity	61	4.539E-04	7.861E-02	4.539E-04	7.861E-02	11	IP3R3, ESR1 (mitochondrial), ESR1 (membrane), ESR1 (nuclear), PKC-epsilon, PKC-gamma, Adenylate cyclase type II, Fc epsilon RI beta, IP3R2, FGFR1, PKA-cat gamma
27	regulation of cytokine-mediated signaling pathway	72	5.357E-04	7.861E-02	5.357E-04	7.861E-02	12	RIPK2, LXR-beta, IFNA1, IFNA2, IFNA16, IL1RN, Gas6, JAK2, WNT5A, FADD, IFNA21, APOA1
28	prostate epithelial cord arborization involved in prostate glandular acinus morphogenesis	8	5.517E-04	7.861E-02	5.517E-04	7.861E-02	4	ESR1 (mitochondrial), ESR1 (membrane), ESR1 (nuclear), FRS2
29	branch elongation involved in mammary gland duct branching	8	5.517E-04	7.861E-02	5.517E-04	7.861E-02	4	ESR1 (mitochondrial), ESR1 (membrane), ESR1 (nuclear), WNT5A
30	prostate glandular acinus morphogenesis	8	5.517E-04	7.861E-02	5.517E-04	7.861E-02	4	ESR1 (mitochondrial), ESR1 (membrane), ESR1 (nuclear), FRS2
31	regulation of lipase activity	93	5.538E-04	7.861E-02	5.538E-04	7.861E-02	14	IP3R3, LXR-beta, ESR1 (mitochondrial), ESR1 (membrane), ESR1 (nuclear), PKC-epsilon, PKC-gamma, Adenylate cyclase type II, Fc epsilon RI beta, IP3R2, JAK2, FGFR1, PKA-cat gamma, APOA1
32	positive regulation of fibroblast proliferation	44	5.734E-04	7.861E-02	5.734E-04	7.861E-02	9	TGIF, ESR1 (mitochondrial), ESR1 (membrane), ESR1 (nuclear), NGFR(TNFRSF16), p21, Gas6, WNT5A, FosB
33	G-protein coupled receptor signaling pathway	413	6.291E-04	7.861E-02	6.291E-04	7.861E-02	39	Transducin, rod-specific, C3, IP3R3, IL8RB, GRK5, GPR50, CCKBR, GPRC5B, ESR1 (mitochondrial), ESR1 (membrane), ESR1 (nuclear), FZD2, CysLT1 receptor, PAX8, Adenylate cyclase type II, Fc epsilon RI beta, Alpha-2A adrenergic receptor, CCR2, Delta-type opioid receptor, Beta-3 adrenergic receptor, GABA-A receptor alpha-2 subunit, LPP1, Beta-1 adrenergic receptor, Parathyroid hormone, HTR1A, FZD10, JAK2, GNAS1, G-protein alpha-s, FRS2, PTHR1, SSTR5, CCR4, G-protein alpha-i2, GABA-A receptor epsilon subunit, EBP50, CaSR, CCR6, APOA1
34	non-canonical Wnt signaling pathway via JNK cascade	4	6.556E-04	7.861E-02	6.556E-04	7.861E-02	3	FZD10, DVL-3, WNT5A
35	epithelial cell maturation involved in prostate gland development	4	6.556E-04	7.861E-02	6.556E-04	7.861E-02	3	ESR2 (mitochondrial), ESR2 (membrane), ESR2
36	adenylate cyclase-activating adrenergic receptor signaling pathway	4	6.556E-04	7.861E-02	6.556E-04	7.861E-02	3	Beta-1 adrenergic receptor, GNAS1, G-protein alpha-s
37	positive regulation of retinoic acid receptor signaling pathway	4	6.556E-04	7.861E-02	6.556E-04	7.861E-02	3	ESR1 (mitochondrial), ESR1 (membrane), ESR1 (nuclear)
38	response to magnetism	4	6.556E-04	7.861E-02	6.556E-04	7.861E-02	3	ESR2 (mitochondrial), ESR2 (membrane), ESR2

39	adrenergic receptor signaling pathway	14	6.829E-04	7.885E-02	6.829E-04	7.885E-02	5	Alpha-2A adrenergic receptor, Beta-3 adrenergic receptor, Beta-1 adrenergic receptor, GNAS1, G-protein alpha-s
40	positive regulation of lipase activity	74	6.923E-04	7.885E-02	6.923E-04	7.885E-02	12	IP3R3, LXR-beta, ESR1 (mitochondrial), ESR1 (membrane), ESR1 (nuclear), PKC-epsilon, PKC-gamma, Adenylate cyclase type II, Fc epsilon RI beta, IP3R2, FGFR1, PKA-cat gamma
41	glutathione metabolic process	37	7.858E-04	8.732E-02	7.858E-04	8.732E-02	8	Glyoxalase II, GSTM5, GSTA5, PNKD, GSTM1, GCL reg, GSTA3, GSTK1
42	response to lipid	745	8.797E-04	9.506E-02	8.797E-04	9.506E-02	61	C3, TPH2, CYP2C9, RIPK2, GATA-3, LXR-beta, Osteoprotegerin, Alpha 1-antitrypsin, ESR1 (mitochondrial), ESR1 (membrane), ESR1 (nuclear), FZD2, PKC-epsilon, HNMT, Caspase-8, NGFR(TNFRSF16), BChE, A-FABP, LDHA, CYP7A1, ALK-2, p21, HSD17B3, DAB2, IL1RN, WNT7B, Parathyroid hormone, PDE4B, FZD10, CD180, DBCCR1, LCAT, JAK2, GNAS1, G-protein alpha-s, SOX9, NOR1, FasR(CD95), ACTA1, CAR, Bid, ACADM, SSTR5, HSD11B1, GSTM1, CNGA3, WNT5A, NRF1, FosB, PKR, IL-22, HEM2, Activin beta B, HLA-DQA1, ABCC5, CaSR, APOA1, ESR2 (mitochondrial), ESR2 (membrane), ESR2, ABL2
43	response to steroid hormone	449	8.971E-04	9.506E-02	8.971E-04	9.506E-02	41	C3, TPH2, GATA-3, LXR-beta, Osteoprotegerin, Alpha 1-antitrypsin, ESR1 (mitochondrial), ESR1 (membrane), ESR1 (nuclear), HNMT, Caspase-8, BChE, A-FABP, LDHA, ALK-2, p21, HSD17B3, DAB2, IL1RN, WNT7B, LCAT, NOR1, FasR(CD95), ACTA1, CAR, Bid, ACADM, SSTR5, HSD11B1, GSTM1, CNGA3, WNT5A, NRF1, FosB, IL-22, HEM2, HLA-DQA1, APOA1, ESR2 (mitochondrial), ESR2 (membrane), ESR2
44	genitalia development	38	9.481E-04	9.631E-02	9.481E-04	9.631E-02	8	ESR1 (mitochondrial), ESR1 (membrane), ESR1 (nuclear), HSD17B3, WNT5A, ESR2 (mitochondrial), ESR2 (membrane), ESR2
45	positive regulation of extrinsic apoptotic signaling pathway	47	9.512E-04	9.631E-02	9.512E-04	9.631E-02	9	Caspase-8, JAK3, FasR(CD95), Bid, Sno-N, FAF1, DEDD2, FADD, Bcl-G
46	extrinsic apoptotic signaling pathway via death domain receptors	30	1.037E-03	1.027E-01	1.037E-03	1.027E-01	7	Caspase-8, NGFR(TNFRSF16), FasR(CD95), Bid, TRADD, DEDD2, FADD
47	adenylate cyclase-activating G-protein coupled receptor signaling pathway	48	1.115E-03	1.081E-01	1.115E-03	1.081E-01	9	Adenylate cyclase type II, Delta-type opioid receptor, Beta-3 adrenergic receptor, Beta-1 adrenergic receptor, Parathyroid hormone, GNAS1, G-protein alpha-s, PTHR1, EBP50
48	phospholipase C-activating G-protein coupled receptor signaling pathway	58	1.181E-03	1.121E-01	1.181E-03	1.121E-01	10	IL8RB, CCKBR, ESR1 (mitochondrial), ESR1 (membrane), ESR1 (nuclear), Alpha-2A adrenergic receptor, Delta-type opioid receptor, PTHR1, EBP50, CaSR
49	cellular response to alcohol	91	1.431E-03	1.330E-01	1.431E-03	1.330E-01	13	ESR1 (mitochondrial), ESR1 (membrane), ESR1 (nuclear), FZD2, PKC-epsilon, CYP7A1, IP3R2, HSD11B1, Activin beta B, CaSR, ESR2 (mitochondrial), ESR2 (membrane), ESR2
50	positive regulation of phospholipase activity	70	1.501E-03	1.351E-01	1.501E-03	1.351E-01	11	IP3R3, ESR1 (mitochondrial), ESR1 (membrane), ESR1 (nuclear), PKC-epsilon, PKC-gamma, Adenylate cyclase type II, Fc epsilon RI beta, IP3R2, FGFR1, PKA-cat gamma

Supplemental Table 4: DR shRNA screen-enriched GO biological processes

TNF GO Processes								
#	Processes	Total	pValue	Min FDR	p-value	FDR	In Data	Network Objects from Active Data
1	response to genistein	5	9.696E-06	3.770E-02	9.696E-06	3.770E-02	4	GSTM1, ESR2 (mitochondrial), ESR2 (membrane), ESR2
2	negative regulation of androgen receptor signaling pathway	11	2.844E-05	4.141E-02	2.844E-05	4.141E-02	5	DAB2, NODAL, ESR2 (mitochondrial), ESR2 (membrane), ESR2
3	cellular response to magnetism	3	5.354E-05	4.141E-02	5.354E-05	4.141E-02	3	ESR2 (mitochondrial), ESR2 (membrane), ESR2
4	glutathione metabolic process	37	5.514E-05	4.141E-02	5.514E-05	4.141E-02	8	Glyoxalase II, PNKD, GSTM5, GSTA5, GSTM1, GCL reg, GSTA3, GSTK1
5	hormone-mediated apoptotic signaling pathway	7	6.390E-05	4.141E-02	6.390E-05	4.141E-02	4	Parathyroid hormone, ESR2 (mitochondrial), ESR2 (membrane), ESR2
6	response to hydroxyisoflavone	7	6.390E-05	4.141E-02	6.390E-05	4.141E-02	4	GSTM1, ESR2 (mitochondrial), ESR2 (membrane), ESR2
7	regulation of response to cytokine stimulus	75	1.020E-04	5.666E-02	1.020E-04	5.666E-02	11	IFNA1, IFNA2, IFNA16, IL1RN, Gas6, RIPK2, ERK5 (MAPK7), WNT5A, FADD, IFNA21, APOA1
8	non-canonical Wnt signaling pathway via JNK cascade	4	2.082E-04	8.093E-02	2.082E-04	8.093E-02	3	FZD10, DVL-3, WNT5A
9	epithelial cell maturation involved in prostate gland development	4	2.082E-04	8.093E-02	2.082E-04	8.093E-02	3	ESR2 (mitochondrial), ESR2 (membrane), ESR2
10	response to magnetism	4	2.082E-04	8.093E-02	2.082E-04	8.093E-02	3	ESR2 (mitochondrial), ESR2 (membrane), ESR2
11	regulation of androgen receptor signaling pathway	16	2.302E-04	8.135E-02	2.302E-04	8.135E-02	5	DAB2, NODAL, ESR2 (mitochondrial), ESR2 (membrane), ESR2
12	response to phenylpropanoid	17	3.161E-04	9.284E-02	3.161E-04	9.284E-02	5	GSTA5, GSTM1, ESR2 (mitochondrial), ESR2 (membrane), ESR2
13	negative regulation of intracellular steroid hormone receptor signaling pathway	17	3.161E-04	9.284E-02	3.161E-04	9.284E-02	5	DAB2, NODAL, ESR2 (mitochondrial), ESR2 (membrane), ESR2
14	regulation of cytokine-mediated signaling pathway	72	3.343E-04	9.284E-02	3.343E-04	9.284E-02	10	IFNA1, IFNA2, IFNA16, IL1RN, Gas6, RIPK2, WNT5A, FADD, IFNA21, APOA1
15	non-canonical Wnt signaling pathway via MAPK cascade	5	5.058E-04	1.277E-01	5.058E-04	1.277E-01	3	FZD10, DVL-3, WNT5A
16	regulation of embryonic development	63	5.369E-04	1.277E-01	5.369E-04	1.277E-01	9	IL1RN, GATA-3, DVL-3, ERK5 (MAPK7), FZD2, WNT5A, FGFR1, NODAL, APOA1
17	uterus development	19	5.583E-04	1.277E-01	5.583E-04	1.277E-01	5	GATA-3, WNT5A, ESR2 (mitochondrial), ESR2 (membrane), ESR2
18	Sertoli cell development	12	7.779E-04	1.592E-01	7.779E-04	1.592E-01	4	SOX9, ESR2 (mitochondrial), ESR2 (membrane), ESR2
19	vagina development	12	7.779E-04	1.592E-01	7.779E-04	1.592E-01	4	WNT5A, ESR2 (mitochondrial), ESR2 (membrane), ESR2
20	regulation of cell-cell adhesion involved in gastrulation	6	9.835E-04	1.821E-01	9.835E-04	1.821E-01	3	IL1RN, ERK5 (MAPK7), APOA1
21	negative regulation of heterotypic cell-cell adhesion	6	9.835E-04	1.821E-01	9.835E-04	1.821E-01	3	IL1RN, ERK5 (MAPK7), APOA1
22	regulation of type I interferon-mediated signaling pathway	32	1.088E-03	1.922E-01	1.088E-03	1.922E-01	6	IFNA1, IFNA2, IFNA16, WNT5A, FADD, IFNA21
23	regulation of gastrulation	23	1.428E-03	2.353E-01	1.428E-03	2.353E-01	5	IL1RN, ERK5 (MAPK7), FGFR1, NODAL, APOA1
24	cochlea morphogenesis	14	1.482E-03	2.353E-01	1.482E-03	2.353E-01	4	SOX9, DVL-3, FZD2, WNT5A
25	response to organic cyclic compound	793	1.513E-03	2.353E-01	1.513E-03	2.353E-01	46	IFNA1, Cathepsin B, p21, IP3R2, C3, TPH2, DAB2, IFNA16, IL1RN, WNT7B, Parathyroid hormone, IP3R3, PDE4B, CYP2C9, LCAT, RIPK2, GATA-3, ACTA1, GSTA5, Bid, Alpha 1-antitrypsin, FZD2, ACADM, SSTR5, HSD11B1, GSTM1, WNT5A, HNMT, Caspase-8, NRF1, FosB, PKR, MT-TRX, ATP12A, PDXK, HEM2, nAChR alpha-3, GPR81, DHFR, CaSR, A-FABP, IFNA21, APOA1, ESR2 (mitochondrial), ESR2 (membrane), ESR2
26	glutathione biosynthetic process	7	1.673E-03	2.502E-01	1.673E-03	2.502E-01	3	Glyoxalase II, PNKD, GCL reg
27	extracellular regulation of signal transduction	15	1.961E-03	2.630E-01	1.961E-03	2.630E-01	4	IL1RN, ESR2 (mitochondrial), ESR2 (membrane), ESR2

28	nuclear-transcribed mRNA catabolic process, deadenylation-dependent decay	15	1.961E-03	2.630E-01	1.961E-03	2.630E-01	4	Nocturnin, CNOT6, eIF4A1, AUMH
29	extracellular negative regulation of signal transduction	15	1.961E-03	2.630E-01	1.961E-03	2.630E-01	4	IL1RN, ESR2 (mitochondrial), ESR2 (membrane), ESR2
30	female genitalia development	16	2.538E-03	2.976E-01	2.538E-03	2.976E-01	4	WNT5A, ESR2 (mitochondrial), ESR2 (membrane), ESR2
31	Sertoli cell differentiation	16	2.538E-03	2.976E-01	2.538E-03	2.976E-01	4	SOX9, ESR2 (mitochondrial), ESR2 (membrane), ESR2
32	fear response	26	2.545E-03	2.976E-01	2.545E-03	2.976E-01	5	HTR1A, Alpha-2A adrenergic receptor, ESR2 (mitochondrial), ESR2 (membrane), ESR2
33	nonribosomal peptide biosynthetic process	8	2.603E-03	2.976E-01	2.603E-03	2.976E-01	3	Glyoxalase II, PNKD, GCL reg
34	negative regulation of fibroblast growth factor receptor signaling pathway	8	2.603E-03	2.976E-01	2.603E-03	2.976E-01	3	GATA-3, WNT5A, NGFR(TNFRSF16)
35	regulation of receptor activity	80	3.019E-03	3.354E-01	3.019E-03	3.354E-01	9	IL1RN, WNT7B, Gas6, WNT5A, NODAL, Alpha-2A adrenergic receptor, ESR2 (mitochondrial), ESR2 (membrane), ESR2
36	positive regulation of cytokine-mediated signaling pathway	17	3.222E-03	3.386E-01	3.222E-03	3.386E-01	4	Gas6, RIPK2, WNT5A, FADD
37	positive regulation of response to cytokine stimulus	17	3.222E-03	3.386E-01	3.222E-03	3.386E-01	4	Gas6, RIPK2, WNT5A, FADD
38	prostate gland epithelium morphogenesis	28	3.575E-03	3.620E-01	3.575E-03	3.620E-01	5	SOX9, FRS2, ESR2 (mitochondrial), ESR2 (membrane), ESR2
39	intracellular estrogen receptor signaling pathway	9	3.796E-03	3.620E-01	3.796E-03	3.620E-01	3	ESR2 (mitochondrial), ESR2 (membrane), ESR2
40	lipoprotein biosynthetic process	41	4.065E-03	3.620E-01	4.065E-03	3.620E-01	6	APG10, CD4, PIGG, LCAT, PIGV, APOA1
41	regulation of interleukin-13 secretion	3	4.175E-03	3.620E-01	4.175E-03	3.620E-01	2	IFNA2, GATA-3
42	ventricular zone neuroblast division	3	4.175E-03	3.620E-01	4.175E-03	3.620E-01	2	TYRP2, FGFR1
43	negative regulation of interleukin-1 secretion	3	4.175E-03	3.620E-01	4.175E-03	3.620E-01	2	Gas6, APOA1
44	regulation of interleukin-5 secretion	3	4.175E-03	3.620E-01	4.175E-03	3.620E-01	2	IFNA2, GATA-3
45	prostate gland morphogenesis	29	4.190E-03	3.620E-01	4.190E-03	3.620E-01	5	SOX9, FRS2, ESR2 (mitochondrial), ESR2 (membrane), ESR2
46	prostate gland development	42	4.597E-03	3.824E-01	4.597E-03	3.824E-01	6	SOX9, FRS2, WNT5A, ESR2 (mitochondrial), ESR2 (membrane), ESR2
47	peptide metabolic process	70	4.622E-03	3.824E-01	4.622E-03	3.824E-01	8	Glyoxalase II, PNKD, GSTM5, GSTA5, GSTM1, GCL reg, GSTA3, GSTK1
48	tetrapyrrole biosynthetic process	19	4.945E-03	4.006E-01	4.945E-03	4.006E-01	4	COX10, HEM2, MMAB, NFE2L1
49	regulation of establishment of planar polarity involved in neural tube closure	10	5.272E-03	4.019E-01	5.272E-03	4.019E-01	3	DVL-3, FZD2, WNT5A
50	planar cell polarity pathway involved in neural tube closure	10	5.272E-03	4.019E-01	5.272E-03	4.019E-01	3	DVL-3, FZD2, WNT5A

Supplemental Table 4: DR shRNA screen-enriched GO biological processes

FAS and TNF GO Processes								
#	Processes	Total	pValue	Min FDR	p-value	FDR	In Data	Network Objects from Active Data
1	response to genistein	5	3.443E-06	1.126E-02	3.443E-06	1.126E-02	4	GSTM1, ESR2 (mitochondrial), ESR2 (membrane), ESR2
2	negative regulation of androgen receptor signaling pathway	11	8.045E-06	1.126E-02	8.045E-06	1.126E-02	5	DAB2, NODAL, ESR2 (mitochondrial), ESR2 (membrane), ESR2
3	regulation of response to cytokine stimulus	75	9.168E-06	1.126E-02	9.168E-06	1.126E-02	11	IFNA1, IFNA2, IFNA16, IL1RN, Gas6, RIPK2, ERK5 (MAPK7), WNT5A, FADD, IFNA21, APOA1
4	response to hydroxyisoflavone	7	2.301E-05	1.810E-02	2.301E-05	1.810E-02	4	GSTM1, ESR2 (mitochondrial), ESR2 (membrane), ESR2
5	cellular response to magnetism	3	2.455E-05	1.810E-02	2.455E-05	1.810E-02	3	ESR2 (mitochondrial), ESR2 (membrane), ESR2
6	regulation of cytokine-mediated signaling pathway	72	3.866E-05	2.375E-02	3.866E-05	2.375E-02	10	IFNA1, IFNA2, IFNA16, IL1RN, Gas6, RIPK2, WNT5A, FADD, IFNA21, APOA1
7	regulation of androgen receptor signaling pathway	16	6.753E-05	2.952E-02	6.753E-05	2.952E-02	5	DAB2, NODAL, ESR2 (mitochondrial), ESR2 (membrane), ESR2
8	regulation of embryonic development	63	7.609E-05	2.952E-02	7.609E-05	2.952E-02	9	IL1RN, GATA-3, DVL-3, ERK5 (MAPK7), FZD2, WNT5A, FGFR1, NODAL, APOA1
9	negative regulation of intracellular steroid hormone receptor signaling pathway	17	9.342E-05	2.952E-02	9.342E-05	2.952E-02	5	DAB2, NODAL, ESR2 (mitochondrial), ESR2 (membrane), ESR2
10	non-canonical Wnt signaling pathway via JNK cascade	4	9.609E-05	2.952E-02	9.609E-05	2.952E-02	3	FZD10, DVL-3, WNT5A
11	epithelial cell maturation involved in prostate gland development	4	9.609E-05	2.952E-02	9.609E-05	2.952E-02	3	ESR2 (mitochondrial), ESR2 (membrane), ESR2
12	response to magnetism	4	9.609E-05	2.952E-02	9.609E-05	2.952E-02	3	ESR2 (mitochondrial), ESR2 (membrane), ESR2
13	uterus development	19	1.674E-04	4.747E-02	1.674E-04	4.747E-02	5	GATA-3, WNT5A, ESR2 (mitochondrial), ESR2 (membrane), ESR2
14	non-canonical Wnt signaling pathway via MAPK cascade	5	2.351E-04	5.629E-02	2.351E-04	5.629E-02	3	FZD10, DVL-3, WNT5A
15	response to organic cyclic compound	793	2.523E-04	5.629E-02	2.523E-04	5.629E-02	40	IFNA1, Cathepsin B, p21, IP3R2, C3, DAB2, IFNA16, IL1RN, WNT7B, IP3R3, PDE4B, CYP2C9, LCAT, RIPK2, GATA-3, ACTA1, Bid, Alpha 1-antitrypsin, FZD2, ACADM, SSTR5, HSD11B1, GSTM1, WNT5A, Caspase-8, NRF1, FosB, PKR, MT-TRX, HEM2, nAChR alpha-3, GPR81, DHFR, A-FABP, CaSR, IFNA21, APOA1, ESR2 (mitochondrial), ESR2 (membrane), ESR2
16	regulation of type I interferon-mediated signaling pathway	32	2.742E-04	5.629E-02	2.742E-04	5.629E-02	6	IFNA1, IFNA2, IFNA16, WNT5A, FADD, IFNA21
17	positive regulation of MAPK cascade	291	2.748E-04	5.629E-02	2.748E-04	5.629E-02	20	C3, DAB2, IL1RN, WNT7B, Gas6, FZD10, PAK3, FRS2, RIPK2, DVL-3, WNT5A, FGFR1, PKR, NODAL, NGFR(TNFRSF16), Alpha-2A adrenergic receptor, Beta-3 adrenergic receptor, ESR2 (mitochondrial), ESR2 (membrane), ESR2
18	Sertoli cell development	12	2.901E-04	5.629E-02	2.901E-04	5.629E-02	4	SOX9, ESR2 (mitochondrial), ESR2 (membrane), ESR2
19	vagina development	12	2.901E-04	5.629E-02	2.901E-04	5.629E-02	4	WNT5A, ESR2 (mitochondrial), ESR2 (membrane), ESR2
20	regulation of gastrulation	23	4.407E-04	7.707E-02	4.407E-04	7.707E-02	5	IL1RN, ERK5 (MAPK7), FGFR1, NODAL, APOA1
21	regulation of cell-cell adhesion involved in gastrulation	6	4.600E-04	7.707E-02	4.600E-04	7.707E-02	3	IL1RN, ERK5 (MAPK7), APOA1
22	negative regulation of heterotypic cell-cell adhesion	6	4.600E-04	7.707E-02	4.600E-04	7.707E-02	3	IL1RN, ERK5 (MAPK7), APOA1
23	regulation of receptor activity	80	4.878E-04	7.817E-02	4.878E-04	7.817E-02	9	IL1RN, WNT7B, Gas6, WNT5A, NODAL, Alpha-2A adrenergic receptor, ESR2 (mitochondrial), ESR2 (membrane), ESR2

Supplemental Table 4: DR shRNA screen-enriched GO biological processes

24	regulation of phosphate metabolic process	934	5.317E-04	8.166E-02	5.317E-04	8.166E-02	44	ARFGAP1, IFNA1, p21, IFNA2, C3, DAB2, IFNA16, IL1RN, CD4, WNT7B, Gas6, FZD10, p18, PAK3, SOX9, eIF4G3, FRS2, RIPK2, DVL-3, ARFGAP3, ERK5 (MAPK7), FZD2, p130, HSD11B1, 14-3-3 gamma, WNT5A, GDI2, FGFR1, PKR, NODAL, eIF2AK1, FAF1, Adenylate cyclase type II, nAChR alpha-3, Alpha-2A adrenergic receptor, CNK3B (IPCEF1), DNMBP(TUBA), A-FABP, CaSR, DDEF2(PAG3), IFNA21, CalDAG-GEFIII, APOA1, Beta-3 adrenergic receptor
25	cochlea morphogenesis	14	5.604E-04	8.263E-02	5.604E-04	8.263E-02	4	SOX9, DVL-3, FZD2, WNT5A
26	regulation of phosphorus metabolic process	941	6.269E-04	8.888E-02	6.269E-04	8.888E-02	44	ARFGAP1, IFNA1, p21, IFNA2, C3, DAB2, IFNA16, IL1RN, CD4, WNT7B, Gas6, FZD10, p18, PAK3, SOX9, eIF4G3, FRS2, RIPK2, DVL-3, ARFGAP3, ERK5 (MAPK7), FZD2, p130, HSD11B1, 14-3-3 gamma, WNT5A, GDI2, FGFR1, PKR, NODAL, eIF2AK1, FAF1, Adenylate cyclase type II, nAChR alpha-3, Alpha-2A adrenergic receptor, CNK3B (IPCEF1), DNMBP(TUBA), A-FABP, CaSR, DDEF2(PAG3), IFNA21, CalDAG-GEFIII, APOA1, Beta-3 adrenergic receptor
27	response to endogenous stimulus	1148	6.798E-04	9.280E-02	6.798E-04	9.280E-02	51	Cathepsin B, p21, IP3R2, CacyBP(SIP), C3, DAB2, IL1RN, WNT7B, IP3R3, PDE4B, COL4A6, PLD1, CYP2C9, LCAT, ATP6V1F (VATF), GSTM5, SOX9, FRS2, RIPK2, GATA-3, ACTA1, Bid, Alpha 1-antitrypsin, ERK5 (MAPK7), FZD2, ACADM, SSTR5, HSD11B1, GSTM1, 14-3-3 gamma, WNT5A, FGFR1, Caspase-8, NRF1, FosB, NODAL, MT-TRX, HEM2, Adenylate cyclase type II, nAChR alpha-3, ATP6M, Alpha-2A adrenergic receptor, CDK9, GPR81, DHFR, A-FABP, CaSR, APOA1, ESR2 (mitochondrial), ESR2 (membrane), ESR2
28	extracellular regulation of signal transduction	15	7.469E-04	9.494E-02	7.469E-04	9.494E-02	4	IL1RN, ESR2 (mitochondrial), ESR2 (membrane), ESR2
29	extracellular negative regulation of signal transduction	15	7.469E-04	9.494E-02	7.469E-04	9.494E-02	4	IL1RN, ESR2 (mitochondrial), ESR2 (membrane), ESR2
30	hormone-mediated apoptotic signaling pathway	7	7.878E-04	9.542E-02	7.878E-04	9.542E-02	3	ESR2 (mitochondrial), ESR2 (membrane), ESR2
31	fear response	26	8.025E-04	9.542E-02	8.025E-04	9.542E-02	5	HTR1A, Alpha-2A adrenergic receptor, ESR2 (mitochondrial), ESR2 (membrane), ESR2
32	regulation of molecular function	1345	9.582E-04	1.055E-01	9.582E-04	1.055E-01	57	ARFGAP1, Cathepsin B, p21, IP3R2, Securin, C3, DAB2, ATR, IL1RN, CD4, WNT7B, IP3R3, PDE4B, Gas6, FZD10, p18, PAK3, FRS2, RIPK2, CNOT6, GATA-3, DVL-3, ARFGAP3, Bid, Alpha 1-antitrypsin, ERK5 (MAPK7), FZD2, p130, 14-3-3 gamma, WNT5A, GDI2, FGFR1, Caspase-8, PKR, NODAL, FAF1, Fc epsilon RI beta, Adenylate cyclase type II, nAChR alpha-3, NGFR(TNFRSF16), PIST (CAL), Collagen VII, SERPINA2, Alpha-2A adrenergic receptor, FADD, DHFR, DNMBP(TUBA), A-FABP, CaSR, DDEF2(PAG3), CalDAG-GEFIII, APOA1, Beta-3 adrenergic receptor, ESR2 (mitochondrial), ESR2 (membrane), ESR2, PPP2R2C
33	female genitalia development	16	9.734E-04	1.055E-01	9.734E-04	1.055E-01	4	WNT5A, ESR2 (mitochondrial), ESR2 (membrane), ESR2
34	Sertoli cell differentiation	16	9.734E-04	1.055E-01	9.734E-04	1.055E-01	4	SOX9, ESR2 (mitochondrial), ESR2 (membrane), ESR2
35	regulation of catalytic activity	1112	1.103E-03	1.094E-01	1.103E-03	1.094E-01	49	ARFGAP1, Cathepsin B, p21, IP3R2, Securin, C3, DAB2, IL1RN, CD4, WNT7B, IP3R3, Gas6, FZD10, p18, PAK3, FRS2, RIPK2, DVL-3, ARFGAP3, Bid, Alpha 1-antitrypsin, ERK5 (MAPK7), p130, 14-3-3 gamma, WNT5A, GDI2, FGFR1, Caspase-8, PKR, NODAL, FAF1, Fc epsilon RI beta, Adenylate cyclase type II, nAChR alpha-3, NGFR(TNFRSF16), PIST (CAL), Collagen VII, SERPINA2, Alpha-2A adrenergic receptor, FADD, DHFR, DNMBP(TUBA), A-FABP, CaSR, DDEF2(PAG3), CalDAG-GEFIII, APOA1, Beta-3 adrenergic receptor, PPP2R2C
36	prostate gland epithelium morphogenesis	28	1.144E-03	1.094E-01	1.144E-03	1.094E-01	5	SOX9, FRS2, ESR2 (mitochondrial), ESR2 (membrane), ESR2

Supplemental Table 4: DR shRNA screen-enriched GO biological processes

37	response to steroid hormone	449	1.159E-03	1.094E-01	1.159E-03	1.094E-01	25	p21, C3, DAB2, IL1RN, WNT7B, LCAT, GATA-3, ACTA1, Bid, Alpha 1-antitrypsin, ACADM, SSTR5, HSD11B1, GSTM1, WNT5A, Caspase-8, NRF1, FosB, HEM2, GPR81, A-FABP, APOA1, ESR2 (mitochondrial), ESR2 (membrane), ESR2
38	negative regulation of fibroblast growth factor receptor signaling pathway	8	1.233E-03	1.094E-01	1.233E-03	1.094E-01	3	GATA-3, WNT5A, NGFR(TNFRSF16)
39	positive regulation of cytokine-mediated signaling pathway	17	1.244E-03	1.094E-01	1.244E-03	1.094E-01	4	Gas6, RIPK2, WNT5A, FADD
40	response to phenylpropanoid	17	1.244E-03	1.094E-01	1.244E-03	1.094E-01	4	GSTM1, ESR2 (mitochondrial), ESR2 (membrane), ESR2
41	positive regulation of response to cytokine stimulus	17	1.244E-03	1.094E-01	1.244E-03	1.094E-01	4	Gas6, RIPK2, WNT5A, FADD
42	prostate gland development	42	1.247E-03	1.094E-01	1.247E-03	1.094E-01	6	SOX9, FRS2, WNT5A, ESR2 (mitochondrial), ESR2 (membrane), ESR2
43	prostate gland morphogenesis	29	1.350E-03	1.151E-01	1.350E-03	1.151E-01	5	SOX9, FRS2, ESR2 (mitochondrial), ESR2 (membrane), ESR2
44	neuron migration	58	1.374E-03	1.151E-01	1.374E-03	1.151E-01	7	Gas6, GATA-3, CCR4, FGFR1, ESR2 (mitochondrial), ESR2 (membrane), ESR2
45	single-organism process	4487	1.673E-03	1.370E-01	1.673E-03	1.370E-01	144	ARFGAP1, IFNA1, Coagulation factor IX, Nocturnin, LAMG1, beta-ENaC, FEN1, PRKCSH, TRIP12, Cathepsin B, p21, AP3M1, IP3R2, MutYH, ATR/TEM8, CacyBP(SIP), KLRC3, Securin, IFNA2, APG10, C3, APRIL(TNFSF13), DAB2, IFNA16, ATR, IL1RN, CD4, WNT7B, XAB2, CD20, PU.1, IP3R3, PDE4B, AZGP1, HTR1A, IL8RB, Gas6, FZD10, p18, ATP5I, COL4A6, GRK5, PLD1, CYP2C9, PAK3, LCAT, ATP6V1F (VATF), TGM1, GSTM5, SOX9, eIF4G3, FRS2, RIPK2, G3P2, LIPG, TYRP2, Carbonic anhydrase XIV, CNOT6, Casein kinase I gamma 2, GATA-3, DVL-3, ACTA1, eIF4A1, Glutaredoxin 1, ARFGAP3, 5'-NTD, GITR, Aquaporin 7, GPR50, CDS2, Bid, Alpha 1-antitrypsin, POLA2, ERK5 (MAPK7), FZD2, iASPP, p130, FOXP1, CNTN1 (F3), ACADM, SSTR5, HSD11B1, GSTM1, CysLT1 receptor, CD1c, CCR4, 14-3-3 gamma, GAMT, WNT5A, GDI2, FGFR1, FXYD2, MLH3, GAD2, NR2D, Caspase-8, NRF1, TANK, Bone sialoprotein, FosB, PKR, CD8 alpha, CLCN2, NODAL, MT-TRX, FAF1, HEM2, Fc epsilon RI beta, Epo receptor, Adenylate cyclase type II, nAChR alpha-3, SNAP-29, NGFR(TNFRSF16), PIST (CAL), PC2 (SPC2), AP2A1, Collagen VII, ATP6M, TLR8, WASF1(WAVE1), Alpha-2A adrenergic receptor, FADD, CDK9, CNK3B (IPCEF1), GPR81, Autophagin-1, DHFR, DNMBP(TUBA), A-FABP, CaSR, TRUNDD(TNFRSF10D), Delta-type opioid receptor, IFNA21, Bcl-G, CCR6, CalDAG-GEFIII, APOA1, Beta- [illegible]
46	intracellular estrogen receptor signaling pathway	9	1.811E-03	1.390E-01	1.811E-03	1.390E-01	3	ESR2 (mitochondrial), ESR2 (membrane), ESR2
47	defense response	903	1.843E-03	1.390E-01	1.843E-03	1.390E-01	41	IFNA1, PRKCSH, Cathepsin B, p21, IP3R2, KLRC3, IFNA2, C3, IFNA16, IL1RN, CD4, IP3R3, HTR1A, IL8RB, PLD1, FRS2, RIPK2, G3P2, GATA-3, Alpha 1-antitrypsin, ERK5 (MAPK7), PLA2G2D, CysLT1 receptor, CCR4, WNT5A, FGFR1, Caspase-8, TANK, PKR, CD8 alpha, Fc epsilon RI beta, Adenylate cyclase type II, TLR8, Alpha-2A adrenergic receptor, FADD, IFNA21, CCR6, ESR2 (mitochondrial), ESR2 (membrane), ESR2, NFE2L1

Supplemental Table 4: DR shRNA screen-enriched GO biological processes

48	response to organic substance	1821	1.847E-03	1.390E-01	1.847E-03	1.390E-01	71	ARFGAP1, IFNA1, Cathepsin B, p21, IP3R2, CacyBP(SIP), IFNA2, C3, DAB2, IFNA16, IL1RN, WNT7B, IP3R3, PDE4B, IL8RB, Gas6, FZD10, COL4A6, PLD1, CYP2C9, LCAT, ATP6V1F (VATF), GSTM5, SOX9, eIF4G3, FRS2, RIPK2, G3P2, GATA-3, ACTA1, eIF4A1, GITR, Bid, Alpha 1-antitrypsin, ERK5 (MAPK7), FZD2, ACADM, SSTR5, HSD11B1, GSTM1, CCR4, 14-3-3 gamma, WNT5A, FGFR1, Caspase-8, NRF1, Bone sialoprotein, FosB, PKR, NODAL, MT-TRX, HEM2, Epo receptor, Adenylate cyclase type II, nAChR alpha-3, NGFR(TNFRSF16), AP2A1, ATP6M, Alpha-2A adrenergic receptor, CDK9, GPR81, DHFR, A-FABP, CaSR, Delta-type opioid receptor, IFNA21, CCR6, APOA1, ESR2 (mitochondrial), ESR2 (membrane), ESR2
49	response to stimulus	3675	1.848E-03	1.390E-01	1.848E-03	1.390E-01	124	ARFGAP1, IFNA1, Coagulation factor IX, IDH2, LAMG1, beta-ENaC, FEN1, PRKCSH, TRIP12, Cathepsin B, p21, IP3R2, MutYH, ATR/TEM8, CacyBP(SIP), KLRC3, Securin, IFNA2, APG10, C3, APRIL(TNFSF13), DAB2, IFNA16, ATR, IL1RN, CD4, WNT7B, XAB2, CD20, IP3R3, PDE4B, AZGP1, AIRE, HTR1A, IL8RB, Gas6, FZD10, COL4A6, GRK5, PLD1, CYP2C9, PAK3, LCAT, ATP6V1F (VATF), GSTM5, SOX9, eIF4G3, FRS2, RIPK2, G3P2, Casein kinase I gamma 2, GATA-3, DVL-3, ACTA1, eIF4A1, 5'-NTD, GITR, Aquaporin 7, GPR50, CDS2, Bid, Alpha 1-antitrypsin, ERK5 (MAPK7), FZD2, PLA2G2D, FOXP1, CNTN1 (F3), ACADM, SSTR5, HSD11B1, GSTM1, CysLT1 receptor, CD1c, CCR4, 14-3-3 gamma, WNT5A, GDI2, FGFR1, MLH3, GAD2, NR2D, Caspase-8, NRF1, TANK, Bone sialoprotein, FosB, PKR, CD8 alpha, NODAL, eIF2AK1, MT-TRX, HEM2, Fc epsilon RI beta, Epo receptor, Adenylate cyclase type II, nAChR alpha-3, SNAP-29, NGFR(TNFRSF16), AP2A1, ATP6M, TLR8, Alpha-2A adrenergic receptor, FADD, CDK9, CNK3B (IPCEF1), GPR81, Autophagin-1, DHFR, DNMBP(TUBA), A-FABP, CaSR, TRUNDD(TNFRSF10D), Delta-type opioid receptor, IFNA21, CCR6, CalDAG-GEFIII, APOA1, Beta-3 adrenergic receptor, ESR2 (mitochondrial), ESR2 (membrane), ESR2, PPP2R2C, NFE2L1, NF-E2 (45 kDa)
50	response to alcohol	366	2.057E-03	1.435E-01	2.057E-03	1.435E-01	21	Cathepsin B, p21, IP3R2, C3, CYP2C9, GSTM5, GATA-3, Bid, Alpha 1-antitrypsin, FZD2, HSD11B1, WNT5A, Caspase-8, NRF1, FosB, HEM2, GPR81, CaSR, ESR2 (mitochondrial), ESR2 (membrane), ESR2

Komarov et al. Functional genetics-directed identification of novel pharmacological inhibitors of FAS- and TNF-dependent apoptosis that protect mice from acute liver failure

Supplemental Table 5:

DR shRNA screen-enriched target networks

Supplemental Table 5: DR shRNA screen-enriched target networks

FAS NETWORKS								
#	Networks	Total	pValue	Min FDR	p-value	FDR	In Data	Network Objects from Active Data
1	Response to hypoxia and oxidative stress	95	8.634E-03	7.743E-01	8.634E-03	7.743E-01	12	p18, GSTM5, Glutaredoxin 1, GSTA5, p21, ATR, G3P2, GSTM1, GCL reg, GSTA3, NRF1, GSTK1
2	Apoptosis_Death Domain receptors & caspases in apoptosis	86	1.072E-02	7.743E-01	1.072E-02	7.743E-01	11	PARP-1, GITR, Caspase-8, NGFR(TNFRSF16), FasR(CD95), CD137 ligand(TNFSF9), TRADD, FAF1, DEDD2, FADD, TRUNDD(TNFRSF10D)
3	Inflammation_Complement system	48	2.024E-02	7.743E-01	2.024E-02	7.743E-01	7	C4BP beta, C3a, C3dg, iC3b, C3, C3b, C1qRp
4	Apoptosis_Apoptotic mitochondria	48	2.024E-02	7.743E-01	2.024E-02	7.743E-01	7	RIPK2, Glutaredoxin 1, NIP1, Bid, tBid, Bcl-G, 14-3-3 eta
5	DNA damage_BER-NER repair	77	3.425E-02	9.960E-01	3.425E-02	9.960E-01	9	FEN1, VPARP, HUS1, PARP-1, Histone H2AX, MutYH, MPG, ATR, XAB2
6	Inflammation_IL-4 signaling	86	6.232E-02	9.960E-01	6.232E-02	9.960E-01	9	CD20, Fc epsilon RI beta, JAK3, IL13RA2, JAK2, jBid, Bid, tBid, HLA-DQA1
7	Apoptosis_Apoptosis stimulation by external signals	103	7.702E-02	9.960E-01	7.702E-02	9.960E-01	10	GITR, Caspase-8, NGFR(TNFRSF16), JAK2, FasR(CD95), jBid, Bid, tBid, TRADD, FADD
8	Apoptosis_Anti-Apoptosis mediated by external signals by Estrogen	52	8.107E-02	9.960E-01	8.107E-02	9.960E-01	6	ESR1 (mitochondrial), ESR1 (membrane), ESR1 (nuclear), PKC-epsilon, PKC-gamma, ESR2
9	Inflammation_IL-2 signaling	65	8.355E-02	9.960E-01	8.355E-02	9.960E-01	7	ESR1 (nuclear), PKC-epsilon, PKC-gamma, CCR2, JAK3, CCR6, ESR2
10	Inflammation_Inflammasome	78	8.388E-02	9.960E-01	8.388E-02	9.960E-01	8	RIPK2, eIF2AK1, IFNA2, PKR, TRADD, TLR8, FADD, APOA1
11	DNA damage_MMR repair	44	1.122E-01	9.960E-01	1.122E-01	9.960E-01	5	Histone H2AX, PMS1, MutYH, ATR, MLH3
12	Apoptosis_Anti-Apoptosis mediated by external signals via MAPK and JAK/STAT	125	1.126E-01	9.960E-01	1.126E-01	9.960E-01	11	IL-15RA, BETA-PIX, CCKBR, PKC-epsilon, NGFR(TNFRSF16), JAK3, HTR1A, JAK2, G-protein alpha-s, PKR, Epo receptor
13	Cardiac development_FGF_ErbB signaling	84	1.164E-01	9.960E-01	1.164E-01	9.960E-01	8	ERK5 (MAPK7), ESR1 (nuclear), PKC-epsilon, FRS2, FOXP1, FGFR1, Epo receptor, G-protein alpha-i2
14	Immune response_Innate immune response to RNA viral infection	58	1.214E-01	9.960E-01	1.214E-01	9.960E-01	6	Caspase-8, TANK, PKR, TRADD, TLR8, FADD
15	Inflammation_Innate inflammatory response	114	1.285E-01	9.960E-01	1.285E-01	9.960E-01	10	C3a, C3, C3b, IP3R3, RIPK2, IP3R2, TANK, G-protein alpha-i2, TLR10, CCR6
16	Cell cycle_G0-G1	46	1.292E-01	9.960E-01	1.292E-01	9.960E-01	5	p18, p130, p21, RBBP4 (RbAp48), p107
17	Muscle contraction_Nitric oxide signaling in the cardiovascular system	62	1.529E-01	9.960E-01	1.529E-01	9.960E-01	6	PKC-epsilon, Beta-3 adrenergic receptor, Beta-1 adrenergic receptor, G-protein alpha-s, FosB, ESR2
18	DNA damage_Core	24	1.631E-01	9.960E-01	1.631E-01	9.960E-01	3	HUS1, Histone H2AX, ATR
19	Reproduction_GnRH signaling pathway	93	1.760E-01	9.960E-01	1.760E-01	9.960E-01	8	ESR1 (nuclear), PKC-epsilon, PKC-gamma, Protein kinase G 2, GABA-A receptor alpha-2 subunit, JAK2, G-protein alpha-s, GABA-A receptor epsilon subunit
20	Development_Keratinocyte differentiation	38	1.804E-01	9.960E-01	1.804E-01	9.960E-01	4	GATA-3, p21, TGM1, CaSR
21	Inflammation_IL-13 signaling pathway	53	1.969E-01	9.960E-01	1.969E-01	9.960E-01	5	PKC-epsilon, Adenylate cyclase type II, IL1RN, IL13RA2, JAK2
22	Cell cycle_G1-S	113	2.151E-01	9.960E-01	2.151E-01	9.960E-01	9	HUS1, p130, CDC14a, p21, ATR, FosB, TAF1, p107, 14-3-3 eta
23	Cell adhesion_Amyloid proteins	128	2.151E-01	9.960E-01	2.151E-01	9.960E-01	10	PAK3, BACE2, FZD2, Caspase-8, NGFR (CTF), NGFR(TNFRSF16), NGFR (ICD), DAB2, WNT7B, WNT5A
24	Inflammation_IL-6 signaling	85	2.286E-01	9.960E-01	2.286E-01	9.960E-01	7	C3, Alpha 1-antitrypsin, p21, JAK3, JAK2, IL-22, 14-3-3 eta
25	Signal transduction_ESR2 pathway	56	2.290E-01	9.960E-01	2.290E-01	9.960E-01	5	CHIP, G-protein alpha-s, 14-3-3 eta, ESR2 (membrane), ESR2
26	Reproduction_Gonadotropin regulation	116	2.376E-01	9.960E-01	2.376E-01	9.960E-01	9	ESR1 (nuclear), PKC-epsilon, PKC-gamma, Protein kinase G 2, GABA-A receptor alpha-2 subunit, JAK2, G-protein alpha-s, FosB, GABA-A receptor epsilon subunit
27	Inflammation_Interferon signaling	87	2.465E-01	9.960E-01	2.465E-01	9.960E-01	7	ESR1 (nuclear), Caspase-8, TAP1 (PSF1), IFNA2, JAK2, FasR(CD95), PKR
28	Inflammation_NK cell cytotoxicity	103	2.548E-01	9.960E-01	2.548E-01	9.960E-01	8	KLRC3, IL-15RA, PKC-epsilon, Caspase-8, IFNA2, FasR(CD95), Bid, FADD
29	Inflammation_Neutrophil activation	135	2.656E-01	9.960E-01	2.656E-01	9.960E-01	10	IL8RB, PLD1, Caspase-8, Adenylate cyclase type II, Bid, tBid, PLA2G2D, TRADD, G-protein alpha-i2, FADD
30	Development_Hedgehog signaling	182	2.670E-01	9.960E-01	2.670E-01	9.960E-01	13	MLL1 (HRX), FZD2, FASN, ALK-2, p21, DAB2, Parathyroid hormone, SOX9, FRS2, PTHR1, WNT5A, FGFR1, G-protein alpha-i2
31	Cell cycle_Core	76	2.853E-01	9.960E-01	2.853E-01	9.960E-01	6	FEN1, p18, p130, CDC14a, p21, p107
32	Signal transduction_Nitric oxide signaling	47	2.959E-01	9.960E-01	2.959E-01	9.960E-01	4	Caspase-8, JAK3, JAK2, G3P2

Supplemental Table 5: DR shRNA screen-enriched target networks

33	Signal transduction_ESR1-membrane pathway	62	2.972E-01	9.960E-01	2.972E-01	9.960E-01	5	ESR1 (membrane), ESR1 (nuclear), PKC-gamma, Adenylate cyclase type II, G-protein alpha-s
34	Neurophysiological process_Taste signaling	6	3.043E-01	9.960E-01	3.043E-01	9.960E-01	1	IP3R3
35	Development_Regulation of telomere length	33	3.048E-01	9.960E-01	3.048E-01	9.960E-01	3	p130, p21, p107
36	Signal Transduction_Cholecystokinin signaling	64	3.207E-01	9.960E-01	3.207E-01	9.960E-01	5	CCKBR, ESR1 (nuclear), PKC-epsilon, FRS2, PC2 (SPC2)
37	Transcription_Nuclear receptors transcriptional regulation	111	3.246E-01	9.960E-01	3.246E-01	9.960E-01	8	LXR-beta, ESR1 (nuclear), G-protein alpha-s, NOR1, FasR(CD95), CAR, TAF1, ESR2
38	Apoptosis_Apoptotic nucleus	112	3.336E-01	9.960E-01	3.336E-01	9.960E-01	8	PARP-1, iASPP, Caspase-8, p21, ATR, Bid, tBid, PKR
39	Neurophysiological process_Circadian rhythm	35	3.377E-01	9.960E-01	3.377E-01	9.960E-01	3	LDHA, Protein kinase G 2, G-protein alpha-s
40	Signal transduction_WNT signaling	132	3.689E-01	9.960E-01	3.689E-01	9.960E-01	9	ESR1 (nuclear), FZD2, p130, PKC-gamma, WNT7B, FZD10, G-protein alpha-s, Casein kinase I gamma 2, WNT5A
41	Immune response_T helper cell differentiation	100	3.704E-01	9.960E-01	3.704E-01	9.960E-01	7	GATA-3, JAK3, CD4, JAK2, CD137 ligand(TNFSF9), IL-22, TLR8
42	Cell cycle_G1-S Growth factor regulation	133	3.774E-01	9.960E-01	3.774E-01	9.960E-01	9	CCKBR, p130, PKC-epsilon, PKC-gamma, p21, JAK2, FRS2, FGFR1, p107
43	Inflammation_Jak-STAT Pathway	133	3.774E-01	9.960E-01	3.774E-01	9.960E-01	9	IL-15RA, CCR2, IL-9, JAK3, IFNA2, IL13RA2, JAK2, IL-22, Epo receptor
44	Apoptosis_Endoplasmic reticulum stress pathway	53	3.775E-01	9.960E-01	3.775E-01	9.960E-01	4	Caspase-8, FasR(CD95), Bid, tBid
45	Autophagy_Autophagy	39	4.030E-01	9.960E-01	4.030E-01	9.960E-01	3	Autophagin-1, APG10, TLR8
46	Transport_Synaptic vesicle exocytosis	55	4.047E-01	9.960E-01	4.047E-01	9.960E-01	4	HNMT, GAD2, SNAP-29, Rab-3A
47	Development_EMT_Regulation of epithelial-to-mesenchymal transition	153	4.081E-01	9.960E-01	4.081E-01	9.960E-01	10	TGIF, ESR1 (nuclear), ALK-2, DAB2, JAK2, SOX9, FRS2, FGFR1, Sno-N, TRADD
48	Apoptosis_Anti-apoptosis mediated by external signals via NF-kB	73	4.277E-01	9.960E-01	4.277E-01	9.960E-01	5	IL-15RA, Caspase-8, NGFR(TNFRSF16), HTR1A, TRADD
49	Transcription_Transcription by RNA polymerase II	57	4.316E-01	9.960E-01	4.316E-01	9.960E-01	4	TFIIF, beta subunit, PARP-1, TAF1, CDK9
50	Development_Melanocyte development and pigmentation	25	4.359E-01	9.960E-01	4.359E-01	9.960E-01	2	G-protein alpha-s, TYRP2

Supplemental Table 5: DR shRNA screen-enriched target networks

TNF NETWORKS

#	Networks	Total	pValue	Min FDR	p-value	FDR	In Data	Network Objects from Active Data
1	Response to hypoxia and oxidative stress	95	1.862E-04	2.440E-02	1.862E-04	2.440E-02	12	p21, ATR, p18, GSTM5, G3P2, Glutaredoxin 1, GSTA5, GSTM1, GCL reg, GSTA3, NRF1, GSTK1
2	Apoptosis_Apoptotic mitochondria	48	8.570E-03	5.614E-01	8.570E-03	5.614E-01	6	RIPK2, Glutaredoxin 1, Bid, tBid, 14-3-3 gamma, Bcl-G
3	Inflammation_Inflammasome	78	2.638E-02	9.910E-01	2.638E-02	9.910E-01	7	IFNA2, RIPK2, PKR, eIF2AK1, TLR8, FADD, APOA1
4	Inflammation_Complement system	48	3.308E-02	9.910E-01	3.308E-02	9.910E-01	5	C3a, C3dg, iC3b, C3, C3b
5	Cell adhesion_Amyloid proteins	128	5.052E-02	9.910E-01	5.052E-02	9.910E-01	9	DAB2, WNT7B, PAK3, FZD2, WNT5A, Caspase-8, NGFR (CTF), NGFR(TNFRSF16), NGFR (ICD)
6	Development_Keratinocyte differentiation	38	5.310E-02	9.910E-01	5.310E-02	9.910E-01	4	p21, TGM1, GATA-3, CaSR
7	Inflammation_Innate inflammatory response	114	6.404E-02	9.910E-01	6.404E-02	9.910E-01	8	IP3R2, C3a, C3, C3b, IP3R3, RIPK2, TANK, CCR6
8	Immune response_Innate immune response to RNA viral infection	58	6.590E-02	9.910E-01	6.590E-02	9.910E-01	5	Caspase-8, TANK, PKR, TLR8, FADD
9	Translation_Translation initiation	60	7.407E-02	9.910E-01	7.407E-02	9.910E-01	5	eIF4G3, Casein kinase I gamma 2, eIF4A1, PKR, eIF2AK1
10	Apoptosis_Apoptosis stimulation by external signals	103	9.202E-02	9.910E-01	9.202E-02	9.910E-01	7	GITR, jBid, Bid, tBid, Caspase-8, NGFR(TNFRSF16), FADD
11	Cardiac development_FGF_ErbB signaling	84	9.548E-02	9.910E-01	9.548E-02	9.910E-01	6	FRS2, ERK5 (MAPK7), FOXP1, FGFR1, Epo receptor, ID1
12	Apoptosis_Death Domain receptors & caspases in apoptosis	86	1.040E-01	9.910E-01	1.040E-01	9.910E-01	6	GITR, Caspase-8, FAF1, NGFR(TNFRSF16), FADD, TRUNDD(TNFRSF10D)
13	Apoptosis_Apoptotic nucleus	112	1.281E-01	9.910E-01	1.281E-01	9.910E-01	7	p21, ATR, Bid, tBid, iASPP, Caspase-8, PKR
14	Inflammation_Neutrophil activation	135	1.352E-01	9.910E-01	1.352E-01	9.910E-01	8	IL8RB, PLD1, Bid, tBid, PLA2G2D, Caspase-8, Adenylate cyclase type II, FADD
15	Development_Hedgehog signaling	182	1.465E-01	9.910E-01	1.465E-01	9.910E-01	10	p21, DAB2, Parathyroid hormone, SOX9, FRS2, PTHR1, FZD2, WNT5A, FGFR1, ID1
16	Signal Transduction_BMP and GDF signaling	75	1.514E-01	9.910E-01	1.514E-01	9.910E-01	5	p21, SOX9, Bone sialoprotein, NODAL, ID1
17	Cell cycle_Core	76	1.575E-01	9.910E-01	1.575E-01	9.910E-01	5	FEN1, p21, Securin, p18, p130
18	Proteolysis_Proteolysis in cell cycle and apoptosis	76	1.575E-01	9.910E-01	1.575E-01	9.910E-01	5	Cathepsin B, Securin, Bid, tBid, Caspase-8
19	Autophagy_Autophagy	39	1.808E-01	9.910E-01	1.808E-01	9.910E-01	3	APG10, TLR8, Autophagin-1
20	Neurophysiological process_Taste signaling	6	2.063E-01	9.910E-01	2.063E-01	9.910E-01	1	IP3R3
21	Cell cycle_Meiosis	63	2.129E-01	9.910E-01	2.129E-01	9.910E-01	4	Securin, ATR, 14-3-3 gamma, MLH3
22	Inflammation_IL-4 signaling	86	2.232E-01	9.910E-01	2.232E-01	9.910E-01	5	CD20, jBid, Bid, tBid, Fc epsilon RI beta
23	DNA damage_MMR repair	44	2.301E-01	9.910E-01	2.301E-01	9.910E-01	3	MutYH, ATR, MLH3
24	Cell cycle_S phase	87	2.301E-01	9.910E-01	2.301E-01	9.910E-01	5	FEN1, p21, Securin, ATR, POLA2
25	Signal transduction_NOTCH signaling	182	2.470E-01	9.910E-01	2.470E-01	9.910E-01	9	p21, DAB2, CD4, WNT7B, FZD10, GATA-3, DVL-3, FZD2, WNT5A
26	Cell cycle_G0-G1	46	2.505E-01	9.910E-01	2.505E-01	9.910E-01	3	p21, p18, p130
27	Transport_Sodium transport	70	2.708E-01	9.910E-01	2.708E-01	9.910E-01	4	beta-ENaC, FXYD2, ASCT1 (SLC1A4), ATP12A
28	Chemotaxis	95	2.881E-01	9.910E-01	2.881E-01	9.910E-01	5	CD4, IL8RB, PLD1, CCR4, CCR6
29	Apoptosis_Anti-apoptosis mediated by external signals via NF-kB	73	2.965E-01	9.910E-01	2.965E-01	9.910E-01	4	APRIL(TNFSF13), HTR1A, Caspase-8, NGFR(TNFRSF16)
30	Apoptosis_Endoplasmic reticulum stress pathway	53	3.235E-01	9.910E-01	3.235E-01	9.910E-01	3	Bid, tBid, Caspase-8
31	DNA damage_BER-NER repair	77	3.311E-01	9.910E-01	3.311E-01	9.910E-01	4	FEN1, MutYH, ATR, XAB2
32	Transport_Synaptic vesicle exocytosis	55	3.445E-01	9.910E-01	3.445E-01	9.910E-01	3	HNMT, GAD2, SNAP-29
33	Inflammation_NK cell cytotoxicity	103	3.485E-01	9.910E-01	3.485E-01	9.910E-01	5	KLRC3, IFNA2, Bid, Caspase-8, FADD
34	Development_Regulation of telomere length	33	3.559E-01	9.910E-01	3.559E-01	9.910E-01	2	p21, p130
35	Signal transduction_WNT signaling	132	3.808E-01	9.910E-01	3.808E-01	9.910E-01	6	WNT7B, FZD10, Casein kinase I gamma 2, FZD2, p130, WNT5A
36	DNA damage_Checkpoint	83	3.835E-01	9.910E-01	3.835E-01	9.910E-01	4	p21, Securin, ATR, 14-3-3 gamma
37	Inflammation_IL-6 signaling	85	4.009E-01	9.910E-01	4.009E-01	9.910E-01	4	p21, C3, Alpha 1-antitrypsin, 14-3-3 gamma
38	Development_Neuromuscular junction	86	4.095E-01	9.910E-01	4.095E-01	9.910E-01	4	LAMG1, DVL-3, 14-3-3 gamma, nAChR alpha-3
39	Muscle contraction_Nitric oxide signaling in the cardiovascular system	62	4.172E-01	9.910E-01	4.172E-01	9.910E-01	3	FosB, Beta-3 adrenergic receptor, ESR2
40	Cell cycle_G1-S	113	4.248E-01	9.910E-01	4.248E-01	9.910E-01	5	p21, ATR, p130, 14-3-3 gamma, FosB

Supplemental Table 5: DR shRNA screen-enriched target networks

41	Protein folding_ER and cytoplasm	17	4.810E-01	9.910E-01	4.810E-01	9.910E-01	1	FAF1
42	Proliferation_Negative regulation of cell proliferation	122	4.921E-01	9.910E-01	4.921E-01	9.910E-01	5	p21, Securin, PTHR1, SSTR5, PKR
43	Cell cycle_Mitosis	70	4.970E-01	9.910E-01	4.970E-01	9.910E-01	3	Securin, 14-3-3 gamma, PKR
44	Transport_Manganese transport	44	5.000E-01	9.910E-01	5.000E-01	9.910E-01	2	FEN1, 5'-NTD
45	Transport_Iron transport	46	5.239E-01	9.910E-01	5.239E-01	9.910E-01	2	TPH2, 5'-NTD
46	Immune response_Phagocytosis	127	5.284E-01	9.910E-01	5.284E-01	9.910E-01	5	C3dg, iC3b, C3, C3b, Fc epsilon RI beta
47	Inflammation_IFN-gamma signaling	74	5.348E-01	9.910E-01	5.348E-01	9.910E-01	3	p21, Caspase-8, PKR
48	Immune response_Antigen presentation	128	5.355E-01	9.910E-01	5.355E-01	9.910E-01	5	AP3M1, KLRC3, CD4, AZGP1, CD8 alpha
49	Signal transduction_Nitric oxide signaling	47	5.356E-01	9.910E-01	5.356E-01	9.910E-01	2	G3P2, Caspase-8
50	Cell adhesion_Leucocyte chemotaxis	129	5.425E-01	9.910E-01	5.425E-01	9.910E-01	5	CD4, IL8RB, PLD1, CCR4, CCR6

Supplemental Table 5: DR shRNA screen-enriched target networks

FAS and TNF NETWORKS								
#	Networks	Total	pValue	Min FDR	p-value	FDR	In Data	Network Objects from Active Data
1	Apoptosis_Apoptotic mitochondria	48	5.453E-03	7.034E-01	5.453E-03	7.034E-01	6	RIPK2, Glutaredoxin 1, Bid, tBid, 14-3-3 gamma, Bcl-G
2	Response to hypoxia and oxidative stress	95	1.530E-02	7.133E-01	1.530E-02	7.133E-01	8	p21, ATR, p18, GSTM5, G3P2, Glutaredoxin 1, GSTM1, NRF1
3	Inflammation_Inflammasome	78	1.659E-02	7.133E-01	1.659E-02	7.133E-01	7	IFNA2, RIPK2, PKR, eIF2AK1, TLR8, FADD, APOA1
4	Inflammation_Complement system	48	2.316E-02	7.465E-01	2.316E-02	7.465E-01	5	C3a, C3dg, iC3b, C3, C3b
5	Cell adhesion_Amyloid proteins	128	3.024E-02	7.465E-01	3.024E-02	7.465E-01	9	DAB2, WNT7B, PAK3, FZD2, WNT5A, Caspase-8, NGFR (CTF), NGFR(TNFRSF16), NGFR (ICD)
6	Development_Keratinocyte differentiation	38	3.977E-02	7.465E-01	3.977E-02	7.465E-01	4	p21, TGM1, GATA-3, CaSR
7	Inflammation_Innate inflammatory response	114	4.051E-02	7.465E-01	4.051E-02	7.465E-01	8	IP3R2, C3a, C3, C3b, IP3R3, RIPK2, TANK, CCR6
8	Immune response_Innate immune response to RNA viral infection	58	4.737E-02	7.639E-01	4.737E-02	7.639E-01	5	Caspase-8, TANK, PKR, TLR8, FADD
9	Translation_Translation initiation	60	5.353E-02	7.672E-01	5.353E-02	7.672E-01	5	eIF4G3, Casein kinase I gamma 2, eIF4A1, PKR, eIF2AK1
10	Apoptosis_Apoptosis stimulation by external signals	103	6.198E-02	7.995E-01	6.198E-02	7.995E-01	7	GITR, jBid, Bid, tBid, Caspase-8, NGFR(TNFRSF16), FADD
11	Apoptosis_Death Domain receptors & caspases in apoptosis	86	7.341E-02	8.609E-01	7.341E-02	8.609E-01	6	GITR, Caspase-8, FAF1, NGFR(TNFRSF16), FADD, TRUNDD(TNFRSF10D)
12	Apoptosis_Apoptotic nucleus	112	8.839E-02	8.982E-01	8.839E-02	8.982E-01	7	p21, ATR, Bid, tBid, iASPP, Caspase-8, PKR
13	Inflammation_Neutrophil activation	135	9.052E-02	8.982E-01	9.052E-02	8.982E-01	8	IL8RB, PLD1, Bid, tBid, PLA2G2D, Caspase-8, Adenylate cyclase type II, FADD
14	Cell cycle_Core	76	1.185E-01	9.861E-01	1.185E-01	9.861E-01	5	FEN1, p21, Securin, p18, p130
15	Proteolysis_Proteolysis in cell cycle and apoptosis	76	1.185E-01	9.861E-01	1.185E-01	9.861E-01	5	Cathepsin B, Securin, Bid, tBid, Caspase-8
16	Autophagy_Autophagy	39	1.486E-01	9.861E-01	1.486E-01	9.861E-01	3	APG10, TLR8, Autophagin-1
17	Cardiac development_FGF_ErbB signaling	84	1.606E-01	9.861E-01	1.606E-01	9.861E-01	5	FRS2, ERK5 (MAPK7), FOXP1, FGFR1, Epo receptor
18	Cell cycle_Meiosis	63	1.694E-01	9.861E-01	1.694E-01	9.861E-01	4	Securin, ATR, 14-3-3 gamma, MLH3
19	Signal transduction_NOTCH signaling	182	1.711E-01	9.861E-01	1.711E-01	9.861E-01	9	p21, DAB2, CD4, WNT7B, FZD10, GATA-3, DVL-3, FZD2, WNT5A
20	Inflammation_IL-4 signaling	86	1.720E-01	9.861E-01	1.720E-01	9.861E-01	5	CD20, jBid, Bid, tBid, Fc epsilon RI beta
21	Cell cycle_S phase	87	1.778E-01	9.861E-01	1.778E-01	9.861E-01	5	FEN1, p21, Securin, ATR, POLA2
22	Neurophysiological process_Taste signaling	6	1.895E-01	9.861E-01	1.895E-01	9.861E-01	1	IP3R3
23	DNA damage_MMR repair	44	1.912E-01	9.861E-01	1.912E-01	9.861E-01	3	MutYH, ATR, MLH3
24	Cell cycle_G0-G1	46	2.091E-01	9.861E-01	2.091E-01	9.861E-01	3	p21, p18, p130
25	Chemotaxis	95	2.267E-01	9.861E-01	2.267E-01	9.861E-01	5	CD4, IL8RB, PLD1, CCR4, CCR6
26	Apoptosis_Anti-apoptosis mediated by external signals via NF-kB	73	2.413E-01	9.861E-01	2.413E-01	9.861E-01	4	APRIL(TNFSF13), HTR1A, Caspase-8, NGFR(TNFRSF16)
27	Signal Transduction_BMP and GDF signaling	75	2.565E-01	9.861E-01	2.565E-01	9.861E-01	4	p21, SOX9, Bone sialoprotein, NODAL
28	DNA damage_BER-NER repair	77	2.718E-01	9.861E-01	2.718E-01	9.861E-01	4	FEN1, MutYH, ATR, XAB2
29	Apoptosis_Endoplasmic reticulum stress pathway	53	2.739E-01	9.861E-01	2.739E-01	9.861E-01	3	Bid, tBid, Caspase-8
30	Inflammation_NK cell cytotoxicity	103	2.792E-01	9.861E-01	2.792E-01	9.861E-01	5	KLRC3, IFNA2, Bid, Caspase-8, FADD
31	Signal transduction_WNT signaling	132	3.003E-01	9.861E-01	3.003E-01	9.861E-01	6	WNT7B, FZD10, Casein kinase I gamma 2, FZD2, p130, WNT5A
32	Development_Regulation of telomere length	33	3.146E-01	9.861E-01	3.146E-01	9.861E-01	2	p21, p130
33	DNA damage_Checkpoint	83	3.188E-01	9.861E-01	3.188E-01	9.861E-01	4	p21, Securin, ATR, 14-3-3 gamma
34	Inflammation_IL-6 signaling	85	3.346E-01	9.861E-01	3.346E-01	9.861E-01	4	p21, C3, Alpha 1-antitrypsin, 14-3-3 gamma
35	Development_Neuromuscular junction	86	3.426E-01	9.861E-01	3.426E-01	9.861E-01	4	LAMG1, DVL-3, 14-3-3 gamma, nAChR alpha-3
36	Cell cycle_G1-S	113	3.477E-01	9.861E-01	3.477E-01	9.861E-01	5	p21, ATR, p130, 14-3-3 gamma, FosB
37	Muscle contraction_Nitric oxide signaling in the cardiovascular system	62	3.597E-01	9.861E-01	3.597E-01	9.861E-01	3	FosB, Beta-3 adrenergic receptor, ESR2
38	Cell cycle_Mitosis	70	4.349E-01	9.861E-01	4.349E-01	9.861E-01	3	Securin, 14-3-3 gamma, PKR
39	Development_Hedgehog signaling	182	4.366E-01	9.861E-01	4.366E-01	9.861E-01	7	p21, DAB2, SOX9, FRS2, FZD2, WNT5A, FGFR1
40	Immune response_Phagocytosis	127	4.448E-01	9.861E-01	4.448E-01	9.861E-01	5	C3dg, iC3b, C3, C3b, Fc epsilon RI beta
41	Protein folding_ER and cytoplasm	17	4.491E-01	9.861E-01	4.491E-01	9.861E-01	1	FAF1

Supplemental Table 5: DR shRNA screen-enriched target networks

42	Transport_Manganese transport	44	4.505E-01	9.861E-01	4.505E-01	9.861E-01	2	FEN1, 5'-NTD
43	Immune response_Antigen presentation	128	4.516E-01	9.861E-01	4.516E-01	9.861E-01	5	AP3M1, KLRC3, CD4, AZGP1, CD8 alpha
44	Cell adhesion_Leucocyte chemotaxis	129	4.585E-01	9.861E-01	4.585E-01	9.861E-01	5	CD4, IL8RB, PLD1, CCR4, CCR6
45	Proliferation_Positive regulation cell proliferation	158	4.623E-01	9.861E-01	4.623E-01	9.861E-01	6	p21, HTR1A, IL8RB, FRS2, FGFR1, Alpha-2A adrenergic receptor
46	Inflammation_IFN-gamma signaling	74	4.713E-01	9.861E-01	4.713E-01	9.861E-01	3	p21, Caspase-8, PKR
47	Inflammation_TREM1 signaling	103	4.760E-01	9.861E-01	4.760E-01	9.861E-01	4	jBid, Bid, tBid, 14-3-3 gamma
48	Transport_Calcium transport	75	4.803E-01	9.861E-01	4.803E-01	9.861E-01	3	PRKCSH, nAChR alpha-3, CaSR
49	Signal transduction_Nitric oxide signaling	47	4.850E-01	9.861E-01	4.850E-01	9.861E-01	2	G3P2, Caspase-8
50	Inflammation_Kallikrein-kinin system	105	4.911E-01	9.861E-01	4.911E-01	9.861E-01	4	Coagulation factor IX, C3a, C3, Alpha 1-antitrypsin

Komarov et al. Functional genetics-directed identification of novel pharmacological inhibitors of FAS- and TNF-dependent apoptosis that protect mice from acute liver failure

Supplemental Table 6:

DR shRNA screen-enriched diseases (biomarkers)

Supplemental Table 6. DR shRNA screen-enriched diseases (biomarkers)

FAS Diseases (by Biomarkers)								
#	Diseases	Total	pValue	Min FDR	p-value	FDR	In Data	Network Objects from Active Data
1	Hip Injuries	8	1.319E-08	1.906E-05	1.319E-08	1.906E-05	7	Osteoprotegerin, ESR1 (mitochondrial), ESR1 (membrane), ESR1 (nuclear), ESR2 (mitochondrial), ESR2 (membrane), ESR2
2	Leg Injuries	9	5.648E-08	2.040E-05	5.648E-08	2.040E-05	7	Osteoprotegerin, ESR1 (mitochondrial), ESR1 (membrane), ESR1 (nuclear), ESR2 (mitochondrial), ESR2 (membrane), ESR2
3	Hip Fractures	9	5.648E-08	2.040E-05	5.648E-08	2.040E-05	7	Osteoprotegerin, ESR1 (mitochondrial), ESR1 (membrane), ESR1 (nuclear), ESR2 (mitochondrial), ESR2 (membrane), ESR2
4	Femoral Fractures	9	5.648E-08	2.040E-05	5.648E-08	2.040E-05	7	Osteoprotegerin, ESR1 (mitochondrial), ESR1 (membrane), ESR1 (nuclear), ESR2 (mitochondrial), ESR2 (membrane), ESR2
5	Pneumonia, Pneumocystis	8	7.929E-07	1.909E-04	7.929E-07	1.909E-04	6	C3a, C3dg, iC3b, C3, C3b, C3c
6	Pneumocystis Infections	8	7.929E-07	1.909E-04	7.929E-07	1.909E-04	6	C3a, C3dg, iC3b, C3, C3b, C3c
7	Encephalomyelitis, Autoimmune, Experimental	12	1.071E-06	1.935E-04	1.071E-06	1.935E-04	7	C3a, C3dg, iC3b, C3, C3b, C3c, FADD
8	Nervous System Autoimmune Disease, Experimental	12	1.071E-06	1.935E-04	1.071E-06	1.935E-04	7	C3a, C3dg, iC3b, C3, C3b, C3c, FADD
9	Hot Flashes	10	5.395E-06	7.796E-04	5.395E-06	7.796E-04	6	ESR1 (mitochondrial), ESR1 (membrane), ESR1 (nuclear), ESR2 (mitochondrial), ESR2 (membrane), ESR2
10	Complement Factor I Deficiency	10	5.395E-06	7.796E-04	5.395E-06	7.796E-04	6	C3a, C3dg, iC3b, C3, C3b, C3c
11	Premenstrual Syndrome	11	1.131E-05	1.485E-03	1.131E-05	1.485E-03	6	ESR1 (mitochondrial), ESR1 (membrane), ESR1 (nuclear), ESR2 (mitochondrial), ESR2 (membrane), ESR2
12	Purpura, Thrombotic Thrombocytopenic	12	2.154E-05	2.594E-03	2.154E-05	2.594E-03	6	C3a, C3dg, iC3b, C3, C3b, C3c
13	Dyslipidemias	151	3.290E-05	3.657E-03	3.290E-05	3.657E-03	22	C3a, C3dg, iC3b, C3, C3b, C3c, CYP2C9, GPR50, ESR1 (mitochondrial), ESR1 (membrane), ESR1 (nuclear), L-FABP, Autophagin-1, A-FABP, CYP7A1, Beta-3 adrenergic receptor, DAB2, HTR1A, LCAT, JAK2, FGFR1, APOA1
14	Lung Diseases, Fungal	13	3.812E-05	3.663E-03	3.812E-05	3.663E-03	6	C3a, C3dg, iC3b, C3, C3b, C3c
15	Poisoning	82	4.325E-05	3.663E-03	4.325E-05	3.663E-03	15	C3a, C3dg, iC3b, C3, C3b, C3c, ESR1 (mitochondrial), ESR1 (membrane), ESR1 (nuclear), Delta-type opioid receptor, MutYH, HTR1A, GSTM1, HEM2, Activin beta B
16	Spinal Fractures	5	4.795E-05	3.663E-03	4.795E-05	3.663E-03	4	Osteoprotegerin, ESR1 (mitochondrial), ESR1 (membrane), ESR1 (nuclear)
17	Spinal Injuries	5	4.795E-05	3.663E-03	4.795E-05	3.663E-03	4	Osteoprotegerin, ESR1 (mitochondrial), ESR1 (membrane), ESR1 (nuclear)
18	Back Injuries	5	4.795E-05	3.663E-03	4.795E-05	3.663E-03	4	Osteoprotegerin, ESR1 (mitochondrial), ESR1 (membrane), ESR1 (nuclear)
19	Varicose Ulcer	19	4.824E-05	3.663E-03	4.824E-05	3.663E-03	7	ESR1 (mitochondrial), ESR1 (membrane), ESR1 (nuclear), FasR(CD95), ESR2 (mitochondrial), ESR2 (membrane), ESR2
20	Glaucoma, Open-Angle	56	5.070E-05	3.663E-03	5.070E-05	3.663E-03	12	COL4A6, ESR1 (mitochondrial), ESR1 (membrane), ESR1 (nuclear), TAP1 (PSF1), p21, Beta-1 adrenergic receptor, FasR(CD95), GSTM1, ESR2 (mitochondrial), ESR2 (membrane), ESR2
21	Varicose Veins	27	8.226E-05	5.403E-03	8.226E-05	5.403E-03	8	Alpha 1-antitrypsin, ESR1 (mitochondrial), ESR1 (membrane), ESR1 (nuclear), FasR(CD95), ESR2 (mitochondrial), ESR2 (membrane), ESR2
22	Hypospadias	27	8.226E-05	5.403E-03	8.226E-05	5.403E-03	8	ESR1 (mitochondrial), ESR1 (membrane), ESR1 (nuclear), HSD17B3, SOX9, ESR2 (mitochondrial), ESR2 (membrane), ESR2
23	Lupus Erythematosus, Systemic	556	1.387E-04	8.692E-03	1.387E-04	8.692E-03	52	C3a, C3dg, iC3b, C3, C3b, C3c, IP3R3, IL8RB, CYP2C9, RIPK2, GATA-3, PARP-1, GITR, Osteoprotegerin, ESR1 (mitochondrial), ESR1 (membrane), ESR1 (nuclear), CD8 alpha, Fc epsilon RI beta, CCR2, TAP1 (PSF1), IFNA1, p21, IFNA2, IFNA16, IL1RN, CD4, HTR1A, Gas6, JAK2, Protein C, FasR(CD95), jBid, Bid, tBid, GSTM1, PKR, IL-22, TRADD, FAF1, DEDD2, TLR8, HLA-DQA1, FADD, CNK3B (IPCEF1), 70Z-PEP, IFNA21, Bcl-G, APOA1, ESR2 (mitochondrial), ESR2 (membrane), ESR2
24	Hypertriglyceridemia	62	1.444E-04	8.692E-03	1.444E-04	8.692E-03	12	C3a, C3dg, iC3b, C3, C3b, C3c, GPR50, Autophagin-1, CYP7A1, Beta-3 adrenergic receptor, JAK2, APOA1
25	Hypoparathyroidism	11	1.949E-04	1.109E-02	1.949E-04	1.109E-02	5	GATA-3, Parathyroid hormone, AIRE, CaSR, 70Z-PEP
26	Parathyroid Diseases	38	1.995E-04	1.109E-02	1.995E-04	1.109E-02	9	p18, GATA-3, p21, Parathyroid hormone, AIRE, GNAS1, G-protein alpha-s, CaSR, 70Z-PEP

Supplemental Table 6. DR shRNA screen-enriched diseases (biomarkers)

27	Puberty, Precocious	17	2.266E-04	1.169E-02	2.266E-04	1.169E-02	6	ESR1 (mitochondrial), ESR1 (membrane), ESR1 (nuclear), Beta-3 adrenergic receptor, GNAS1, G-protein alpha-s
28	Cicatrix, Hypertrophic	17	2.266E-04	1.169E-02	2.266E-04	1.169E-02	6	C3a, C3dg, iC3b, C3, C3b, C3c
29	Uremia	39	2.467E-04	1.208E-02	2.467E-04	1.208E-02	9	C3a, C3dg, iC3b, C3, C3b, C3c, Protein C, PTHR1, TAF1
30	Drug-Induced Liver Injury, Chronic	24	2.592E-04	1.208E-02	2.592E-04	1.208E-02	7	C3a, C3dg, iC3b, C3, C3b, C3c, GSTM1
31	Drug-Induced Liver Injury	24	2.592E-04	1.208E-02	2.592E-04	1.208E-02	7	C3a, C3dg, iC3b, C3, C3b, C3c, GSTM1
32	Signs and Symptoms	1206	2.683E-04	1.212E-02	2.683E-04	1.212E-02	94	Transducin, rod-specific, ATR/TEM8, MLL1 (HRX), sIL-15RA, IL-15RA, C3a, C3dg, iC3b, C3, C3b, C3c, CD20, IP3R3, AZGP1, p18, CYP2C9, PAK3, GATA-3, LXR-beta, CCKBR, Osteoprotegerin, CHIP, ERK5 (MAPK7), ESR1 (mitochondrial), ESR1 (membrane), ESR1 (nuclear), PKC-epsilon, FASN, GAD2, Caspase-8, p18 Casp-8, PKC-gamma, MT-TRX, Adenylate cyclase type II, Fc epsilon RI beta, L-FABP, BChE, Alpha-2A adrenergic receptor, CCR2, PMS1, Rab-3A, TAP1 (PSF1), A-FABP, Delta-type opioid receptor, Beta-3 adrenergic receptor, IFNA1, COX10, p21, MR1, ATR, GALE, IL1RN, PNKD, CD4, PMP22, Beta-1 adrenergic receptor, HTR3C, PU.1, HTR1A, Gas6, JAK2, GNAS1, G-protein alpha-s, SOX9, LIPG, Casein kinase I gamma 2, FasR(CD95), Aquaporin 7, ALPP, jBid, Bid, tBid, PLA2G2D, ACADM, HSD11B1, GSTM1, CNGA3, GAMT, FGFR1, AUMH, FosB, TAF1, PKA-cat gamma, Epo receptor, GSTK1, Activin beta B, HLA-DQA1, FADD, CaSR, 70Z-PEP, APOA1, ESR2 (mitochondrial), ESR2 (membrane), ESR2
33	Lipid Metabolism Disorders	211	3.051E-04	1.336E-02	3.051E-04	1.336E-02	25	C3a, C3dg, iC3b, C3, C3b, C3c, CYP2C9, GPR50, ESR1 (mitochondrial), ESR1 (membrane), ESR1 (nuclear), L-FABP, Autophagin-1, A-FABP, CYP7A1, Beta-3 adrenergic receptor, DAB2, HTR1A, LCAT, JAK2, FasR(CD95), ACADM, HSD11B1, FGFR1, APOA1
34	Hemolytic-Uremic Syndrome	25	3.427E-04	1.457E-02	3.427E-04	1.457E-02	7	C3a, C3dg, iC3b, C3, C3b, C3c, TAF1
35	Body Constitution	506	3.568E-04	1.473E-02	3.568E-04	1.473E-02	47	MLL1 (HRX), sIL-15RA, IL-15RA, C3a, C3dg, iC3b, C3, C3b, C3c, IP3R3, AZGP1, p18, CYP2C9, LXR-beta, PARP-1, Osteoprotegerin, ESR1 (mitochondrial), ESR1 (membrane), ESR1 (nuclear), FASN, GAD2, Adenylate cyclase type II, Alpha-2A adrenergic receptor, A-FABP, Beta-3 adrenergic receptor, IL1RN, CD4, Beta-1 adrenergic receptor, PU.1, Gas6, GNAS1, G-protein alpha-s, LIPG, FasR(CD95), Aquaporin 7, ALPP, PLA2G2D, HSD11B1, FGFR1, GSTK1, HLA-DQA1, CaSR, 70Z-PEP, APOA1, ESR2 (mitochondrial), ESR2 (membrane), ESR2
36	Skin Diseases	3381	3.820E-04	1.533E-02	3.820E-04	1.533E-02	219	TFIIF, beta subunit, Coagulation factor IX, PIWIL3, FEN1, Glyoxalase II, SHC4, CACNA2D4, ATR/TEM8, MLL1 (HRX), C4BP beta, VPARP, C3a, C3dg, iC3b, C3, C3b, C3c, TPH2, Claudin-16, CD20, IP3R3, AZGP1, IL8RB, MTIF2, p18, SPF, COL4A6, GRK5, PIGG, PLD1, CYP2C9, PAK3, Collagen XXIII, AMYS, HUS1, SIA7C, WWP2, GSTM5, RIPK2, BACE2, CNOT6, ABCB9, GATA-3, eIF4A1, FUT4, BETA-PIX, PARP-1, 5'-NTD, CCKBR, GSTA5, Osteoprotegerin, GPRC5B, Alpha 1-antitrypsin, ERK5 (MAPK7), ESR1 (mitochondrial), ESR1 (membrane), ESR1 (nuclear), iASPP, p130, CNTN1 (F3), PKC-epsilon, Histone H2AX, CSTF3, CysLT1 receptor, CD1c, KCRS, PIGV, FASN, HNMT, GAD2, NR2D, Caspase-8, p18 Casp-8, ASCT1 (SLC1A4), Bone sialoprotein, C1qRp, PKC-gamma, CD8 alpha, PLAP-like, MT-TRX, Adenylate cyclase type II, Fc epsilon RI beta, SNAP-29, L-FABP, NGFR (CTF), NGFR(TNFRSF16), NGFR (ICD), BChE, WASF1(WAVE1), COL5A3, CCR2, PMS1, TAP1 (PSF1), CD34, A-FABP, DDEF2(PAG3), Delta-type opioid receptor, GA6S, LDHA, CYP7A1, Beta-3 adrenergic receptor, TDO2, NFE2L1, M-cadherin, IFNA1, Protein kinase G 2, GPT, LAMG1, Keratin 17, COX10, Cathepsin B, CDC14a, p21, JAK3, AP3M1, IP3R2, MutYH, MR1, MPG, HSD17B3, DAB2, NIP1, CHKA, ATR, IL1RN, CD4, PMP22, B4GT7, HTR3C, PYC, WNT7B, PDE4B, AIRE, RBBP4 (RbAp48), DBC1, HTR1A, Gas6, FZD10, ATP5I, SLC16A3, DBCCR1, BHMT, LCAT, IL13RA2, ATP6V1F (VATF), JAK2, TGM1, GNAS1, G-protein alpha-s, SOX9, UBE2G2, G3P2, TYRP2, DVL-3, FasR(CD95), Dysferlin, ARFGAP3, CAR, B4G6, jBid, Bid, tBid, PTHR1, FOXP1, SSTR5, HSD11B1, GSTM1, M33, GCL reg, GSTA3, CNGA3, CCR4, CD137 ligand(TNFSF9), WNT5A, GDI2, FGFR1, FXYD2, MLH3
37	Neoplasms, Fibroepithelial	19	4.510E-04	1.715E-02	4.510E-04	1.715E-02	6	ESR1 (mitochondrial), ESR1 (membrane), ESR1 (nuclear), ESR2 (mitochondrial), ESR2 (membrane), ESR2
38	Fibroadenoma	19	4.510E-04	1.715E-02	4.510E-04	1.715E-02	6	ESR1 (mitochondrial), ESR1 (membrane), ESR1 (nuclear), ESR2 (mitochondrial), ESR2 (membrane), ESR2

39	Panic Disorder	51	4.633E-04	1.716E-02	4.633E-04	1.716E-02	10	TPH2, CCKBR, ESR1 (mitochondrial), ESR1 (membrane), ESR1 (nuclear), NGFR (CTF), NGFR(TNFRSF16), NGFR (ICD), LDHA, HTR1A
40	Dermatitis, Atopic	205	4.814E-04	1.722E-02	4.814E-04	1.722E-02	24	C3a, C3dg, iC3b, C3, C3b, C3c, CD20, GATA-3, CysLT1 receptor, HNMT, Fc epsilon RI beta, NGFR (CTF), NGFR(TNFRSF16), NGFR (ICD), TAP1 (PSF1), p21, IL1RN, PDE4B, HTR1A, IL13RA2, GSTM1, CCR4, IL-22, TLR8
41	Body Weight	513	4.885E-04	1.722E-02	4.885E-04	1.722E-02	47	MLL1 (HRX), sIL-15RA, IL-15RA, C3a, C3dg, iC3b, C3, C3b, C3c, IP3R3, AZGP1, p18, CYP2C9, LXR-beta, Osteoprotegerin, ESR1 (mitochondrial), ESR1 (membrane), ESR1 (nuclear), FASN, GAD2, Adenylate cyclase type II, Alpha-2A adrenergic receptor, A-FABP, Beta-3 adrenergic receptor, IL1RN, CD4, Beta-1 adrenergic receptor, PU.1, Gas6, GNAS1, G-protein alpha-s, LIPG, FasR(CD95), Aquaporin 7, ALPP, PLA2G2D, HSD11B1, FGFR1, FosB, GSTK1, Activin beta B, HLA-DQA1, CaSR, APOA1, ESR2 (mitochondrial), ESR2 (membrane), ESR2
42	Fractures, Bone	52	5.454E-04	1.876E-02	5.454E-04	1.876E-02	10	Osteoprotegerin, ESR1 (mitochondrial), ESR1 (membrane), ESR1 (nuclear), IL1RN, Parathyroid hormone, CaSR, ESR2 (mitochondrial), ESR2 (membrane), ESR2
43	Spinal Osteophytosis	8	5.855E-04	1.888E-02	5.855E-04	1.888E-02	4	ESR1 (mitochondrial), ESR1 (membrane), ESR1 (nuclear), Parathyroid hormone
44	Dermatitis	351	6.032E-04	1.888E-02	6.032E-04	1.888E-02	35	C3a, C3dg, iC3b, C3, C3b, C3c, CD20, CYP2C9, GATA-3, CysLT1 receptor, HNMT, Caspase-8, p18 Casp-8, PKC-gamma, Fc epsilon RI beta, NGFR (CTF), NGFR(TNFRSF16), NGFR (ICD), CCR2, TAP1 (PSF1), p21, IL1RN, CD4, PDE4B, HTR1A, IL13RA2, ATP6V1F (VATF), TGM1, FasR(CD95), GSTM1, GCL reg, CCR4, IL-22, TLR8, CCR6
45	Skin and Connective Tissue Diseases	3570	6.058E-04	1.888E-02	6.058E-04	1.888E-02	228	TFIIF, beta subunit, Coagulation factor IX, PIWIL3, FEN1, Glyoxalase II, SHC4, CACNA2D4, ATR/TEM8, MLL1 (HRX), C4BP beta, VPARP, C3a, C3dg, iC3b, C3, C3b, C3c, TPH2, Claudin-16, CD20, IP3R3, AZGP1, IL8RB, MTIF2, p18, SPF, COL4A6, GRK5, PIGG, PLD1, CYP2C9, PAK3, Collagen XXIII, AMYS, HUS1, SIA7C, WWP2, GSTM5, RIPK2, BACE2, CNOT6, ABCB9, GATA-3, eIF4A1, FUT4, BETA-PIX, PARP-1, GITR, 5'-NTD, CCKBR, GSTA5, Osteoprotegerin, GPRC5B, Alpha 1-antitrypsin, ERK5 (MAPK7), ESR1 (mitochondrial), ESR1 (membrane), ESR1 (nuclear), iASPP, p130, CNTN1 (F3), PKC-epsilon, Histone H2AX, CSTF3, CysLT1 receptor, CD1c, KCRS, PIGV, FASN, HNMT, GAD2, NR2D, Caspase-8, p18 Casp-8, ASCT1 (SLC1A4), Bone sialoprotein, C1qRp, PKC-gamma, CD8 alpha, PLAP-like, MT-TRX, Adenylate cyclase type II, Fc epsilon RI beta, SNAP-29, L-FABP, NGFR (CTF), NGFR(TNFRSF16), NGFR (ICD), BChE, WASF1(WAVE1), COL5A3, CCR2, PMS1, TAP1 (PSF1), CD34, A-FABP, DDEF2(PAG3), Delta-type opioid receptor, GA6S, LDHA, CYP7A1, IL-9, Beta-3 adrenergic receptor, TDO2, NFE2L1, Podocalyxin-like 1, M-cadherin, IFNA1, Protein kinase G 2, GPT, LAMG1, Keratin 17, COX10, Cathepsin B, CDC14a, p21, JAK3, AP3M1, IP3R2, MutYH, MR1, MPG, IFNA2, HSD17B3, DAB2, NIP1, CHKA, ATR, IFNA16, IL1RN, CD4, PMP22, B4GT7, HTR3C, PYC, WNT7B, PDE4B, AIRE, RBBP4 (RbAp48), DBC1, HTR1A, Gas6, FZD10, ATP5I, SLC16A3, DBCCR1, BHMT, LCAT, IL13RA2, ATP6V1F (VATF), JAK2, TGM1, GNAS1, G-protein alpha-s, SOX9, Protein C, UBE2G2, G3P2, TYRP2, DVL-3, FasR(CD95), Dysferlin, ARFGAP3, CAR, B4G6, jBid, Bid, tBid, PTHR1, PLA2G2D, FOXP1, SSTR5, HSD11B1, GSTM1, M33, GCL reg
46	Infertility, Female	20	6.140E-04	1.888E-02	6.140E-04	1.888E-02	6	ESR1 (mitochondrial), ESR1 (membrane), ESR1 (nuclear), ESR2 (mitochondrial), ESR2 (membrane), ESR2
47	Adrenocortical Adenoma	20	6.140E-04	1.888E-02	6.140E-04	1.888E-02	6	ESR1 (mitochondrial), ESR1 (membrane), ESR1 (nuclear), ESR2 (mitochondrial), ESR2 (membrane), ESR2
48	Body Weights and Measures	492	6.766E-04	2.004E-02	6.766E-04	2.004E-02	45	MLL1 (HRX), sIL-15RA, IL-15RA, C3a, C3dg, iC3b, C3, C3b, C3c, IP3R3, AZGP1, p18, CYP2C9, LXR-beta, Osteoprotegerin, ESR1 (mitochondrial), ESR1 (membrane), ESR1 (nuclear), FASN, GAD2, Adenylate cyclase type II, Alpha-2A adrenergic receptor, A-FABP, Beta-3 adrenergic receptor, IL1RN, CD4, Beta-1 adrenergic receptor, PU.1, Gas6, GNAS1, G-protein alpha-s, LIPG, FasR(CD95), Aquaporin 7, ALPP, PLA2G2D, HSD11B1, FGFR1, GSTK1, HLA-DQA1, CaSR, APOA1, ESR2 (mitochondrial), ESR2 (membrane), ESR2
49	Cadmium Poisoning	4	6.865E-04	2.004E-02	6.865E-04	2.004E-02	3	ESR1 (mitochondrial), ESR1 (membrane), ESR1 (nuclear)

Supplemental Table 6. DR shRNA screen-enriched diseases (biomarkers)

50	Eye Diseases	847	6.935E-04	2.004E-02	6.935E-04	2.004E-02	69	Transducin, rod-specific, Csk, C3a, C3dg, iC3b, C3, C3b, C3c, TGIF, IP3R3, IL8RB, DES1, COL4A6, CYP2C9, RIPK2, GATA-3, PARP-1, Alpha 1-antitrypsin, ESR1 (mitochondrial), ESR1 (membrane), ESR1 (nuclear), p130, CD1c, Caspase-8, p18 Casp-8, PKC-gamma, CD8 alpha, NGFR (CTF), NGFR(TNFRSF16), NGFR (ICD), TAP1 (PSF1), CD34, LDHA, Beta-3 adrenergic receptor, IFNA1, LAMG1, Keratin 17, Cathepsin B, p21, AP3M1, IFNA2, IFNA16, GALE, IL1RN, CD4, Beta-1 adrenergic receptor, RBBP4 (RbAp48), HTR1A, Gas6, LCAT, JAK2, TYRP2, FasR(CD95), SSTR5, GSTM1, CNGA3, CCR4, WNT5A, FGFR1, IL-22, Epo receptor, HLA-DQA1, p107, 70Z-PEP, IFNA21, APOA1, ESR2 (mitochondrial), ESR2 (membrane), ESR2

Supplemental Table 6. DR shRNA screen-enriched diseases (biomarkers)

TNF Diseases (by Biomarkers)								
#	Diseases	Total	pValue	Min FDR	p-value	FDR	In Data	Network Objects from Active Data
1	Encephalomyelitis, Autoimmune, Experimental	12	7.628E-08	2.375E-05	7.628E-08	2.375E-05	7	C3a, C3dg, iC3b, C3, C3b, C3c, FADD
2	Nervous System Autoimmune Disease, Experimental	12	7.628E-08	2.375E-05	7.628E-08	2.375E-05	7	C3a, C3dg, iC3b, C3, C3b, C3c, FADD
3	Pneumonia, Pneumocystis	8	7.964E-08	2.375E-05	7.964E-08	2.375E-05	6	C3a, C3dg, iC3b, C3, C3b, C3c
4	Pneumocystis Infections	8	7.964E-08	2.375E-05	7.964E-08	2.375E-05	6	C3a, C3dg, iC3b, C3, C3b, C3c
5	Complement Factor I Deficiency	10	5.597E-07	1.335E-04	5.597E-07	1.335E-04	6	C3a, C3dg, iC3b, C3, C3b, C3c
6	Purpura, Thrombotic Thrombocytopenic	12	2.307E-06	4.588E-04	2.307E-06	4.588E-04	6	C3a, C3dg, iC3b, C3, C3b, C3c
7	Lung Diseases, Fungal	13	4.148E-06	7.070E-04	4.148E-06	7.070E-04	6	C3a, C3dg, iC3b, C3, C3b, C3c
8	Dermatitis, Atopic	205	1.007E-05	1.501E-03	1.007E-05	1.501E-03	22	p21, C3a, C3dg, iC3b, C3, C3b, C3c, APRIL(TNFSF13), IL1RN, CD20, PDE4B, HTR1A, GATA-3, GSTM1, CysLT1 receptor, CCR4, HNMT, Fc epsilon RI beta, NGFR (CTF), NGFR(TNFRSF16), NGFR (ICD), TLR8
9	Skin Diseases, Genetic	314	2.133E-05	2.397E-03	2.133E-05	2.397E-03	28	p21, AP3M1, C3a, C3dg, iC3b, C3, C3b, C3c, APRIL(TNFSF13), IL1RN, B4GT7, CD20, PDE4B, HTR1A, TGM1, GATA-3, GSTM1, CysLT1 receptor, CCR4, HNMT, HEM2, Fc epsilon RI beta, SNAP-29, NGFR (CTF), NGFR(TNFRSF16), NGFR (ICD), Collagen VII, TLR8
10	Drug-Induced Liver Injury, Chronic	24	2.243E-05	2.397E-03	2.243E-05	2.397E-03	7	C3a, C3dg, iC3b, C3, C3b, C3c, GSTM1
11	Drug-Induced Liver Injury	24	2.243E-05	2.397E-03	2.243E-05	2.397E-03	7	C3a, C3dg, iC3b, C3, C3b, C3c, GSTM1
12	Dermatitis	351	2.411E-05	2.397E-03	2.411E-05	2.397E-03	30	p21, C3a, C3dg, iC3b, C3, C3b, C3c, APRIL(TNFSF13), IL1RN, CD4, CD20, PDE4B, HTR1A, CYP2C9, ATP6V1F (VATF), TGM1, GATA-3, GSTM1, CysLT1 receptor, GCL reg, CCR4, HNMT, Caspase-8, p18 Casp-8, Fc epsilon RI beta, NGFR (CTF), NGFR(TNFRSF16), NGFR (ICD), TLR8, CCR6
13	Cicatrix, Hypertrophic	17	2.627E-05	2.411E-03	2.627E-05	2.411E-03	6	C3a, C3dg, iC3b, C3, C3b, C3c
14	Skin Diseases, Eczematous	325	4.014E-05	3.421E-03	4.014E-05	3.421E-03	28	p21, C3a, C3dg, iC3b, C3, C3b, C3c, APRIL(TNFSF13), IL1RN, CD20, PDE4B, HTR1A, ATP6V1F (VATF), TGM1, GATA-3, GSTM1, CysLT1 receptor, GCL reg, CCR4, HNMT, Caspase-8, p18 Casp-8, Fc epsilon RI beta, NGFR (CTF), NGFR(TNFRSF16), NGFR (ICD), TLR8, CCR6
15	Hypertriglyceridemia	62	1.073E-04	8.533E-03	1.073E-04	8.533E-03	10	C3a, C3dg, iC3b, C3, C3b, C3c, GPR50, Autophagin-1, APOA1, Beta-3 adrenergic receptor
16	Purpura, Thrombocytopenic	41	1.346E-04	1.003E-02	1.346E-04	1.003E-02	8	C3a, C3dg, iC3b, C3, C3b, C3c, IL1RN, CD20
17	Hyperlipidemia, Familial Combined	32	1.677E-04	1.177E-02	1.677E-04	1.177E-02	7	C3a, C3dg, iC3b, C3, C3b, C3c, APOA1
18	Dyslipidemias	151	2.000E-04	1.326E-02	2.000E-04	1.326E-02	16	C3a, C3dg, iC3b, C3, C3b, C3c, DAB2, HTR1A, CYP2C9, LCAT, GPR50, FGFR1, Autophagin-1, A-FABP, APOA1, Beta-3 adrenergic receptor
19	Encephalitis	80	2.142E-04	1.345E-02	2.142E-04	1.345E-02	11	IFNA1, C3a, C3dg, iC3b, C3, C3b, C3c, IL1RN, PKR, CD8 alpha, FADD
20	Pathological Conditions, Signs and Symptoms	2107	2.422E-04	1.445E-02	2.422E-04	1.445E-02	106	IFNA1, LAMG1, beta-ENaC, FEN1, PRKCSH, COX10, Cathepsin B, p21, MutYH, MR1, ATR/TEM8, Securin, C3a, C3dg, iC3b, C3, C3b, C3c, APRIL(TNFSF13), TPH2, ATR, GALE, IL1RN, PNKD, CD4, HTR3C, CD20, PU.1, Parathyroid hormone, IP3R3, AZGP1, AIRE, HTR1A, IL8RB, Gas6, p18, COL4A6, CYP2C9, PAK3, LCAT, SOX9, RIPK2, G3P2, LIPG, Casein kinase I gamma 2, GATA-3, ACTA1, Glutaredoxin 1, Dysferlin, GITR, Aquaporin 7, ALPP, jBid, Bid, tBid, PTHR1, Alpha 1-antitrypsin, ERK5 (MAPK7), PLA2G2D, FOXP1, ACADM, HSD11B1, GSTM1, GCL reg, 14-3-3 gamma, GAMT, WNT5A, GDI2, FGFR1, HNMT, GAD2, Caspase-8, p18 Casp-8, AUMH, TANK, Bone sialoprotein, FosB, PKR, CD8 alpha, CLCN2, PLAP-like, MT-TRX, Adenylate cyclase type II, Fc epsilon RI beta, Epo receptor, NGFR (CTF), NGFR(TNFRSF16), NGFR (ICD), PC2 (SPC2), WASF1(WAVE1), GSTK1, Alpha-2A adrenergic receptor, FADD, DHFR, CaSR, A-FABP, TRUNDD(TNFRSF10D), Delta-type opioid receptor, CPGL2, CCR6, ID1, APOA1, Beta-3 adrenergic receptor, ESR2 (mitochondrial), ESR2 (membrane), ESR2

Supplemental Table 6. DR shRNA screen-enriched diseases (biomarkers)

21	Lupus Erythematosus, Systemic	556	2.656E-04	1.451E-02	2.656E-04	1.451E-02	38	IFNA1, p21, IFNA2, C3a, C3dg, iC3b, C3, C3b, C3c, APRIL(TNFSF13), IFNA16, IL1RN, CD4, IP3R3, HTR1A, IL8RB, Gas6, CYP2C9, RIPK2, GATA-3, GITR, jBid, Bid, tBid, GSTM1, PKR, CD8 alpha, FAF1, Fc epsilon RI beta, TLR8, FADD, CNK3B (IPCEF1), IFNA21, Bcl-G, APOA1, ESR2 (mitochondrial), ESR2 (membrane), ESR2
22	Poisoning	82	2.675E-04	1.451E-02	2.675E-04	1.451E-02	11	MutYH, C3a, C3dg, iC3b, C3, C3b, C3c, HTR1A, GSTM1, HEM2, Delta-type opioid receptor
23	Hemolytic-Uremic Syndrome	25	2.903E-04	1.506E-02	2.903E-04	1.506E-02	6	C3a, C3dg, iC3b, C3, C3b, C3c
24	Thyroid Carcinoma, Follicular	58	3.223E-04	1.602E-02	3.223E-04	1.602E-02	9	C3a, C3dg, iC3b, C3, C3b, C3c, IL1RN, Collagen XXIII, FosB
25	Foot Deformities	18	4.581E-04	1.952E-02	4.581E-04	1.952E-02	5	jBid, Bid, tBid, Caspase-8, p18 Casp-8
26	Clubfoot	18	4.581E-04	1.952E-02	4.581E-04	1.952E-02	5	jBid, Bid, tBid, Caspase-8, p18 Casp-8
27	Foot Deformities, Congenital	18	4.581E-04	1.952E-02	4.581E-04	1.952E-02	5	jBid, Bid, tBid, Caspase-8, p18 Casp-8
28	Lower Extremity Deformities, Congenital	18	4.581E-04	1.952E-02	4.581E-04	1.952E-02	5	jBid, Bid, tBid, Caspase-8, p18 Casp-8
29	Hypoparathyroidism	11	5.691E-04	2.341E-02	5.691E-04	2.341E-02	4	Parathyroid hormone, AIRE, GATA-3, CaSR
30	Uremia	39	6.094E-04	2.424E-02	6.094E-04	2.424E-02	7	C3a, C3dg, iC3b, C3, C3b, C3c, PTHR1
31	Encephalomyelitis	53	8.391E-04	3.229E-02	8.391E-04	3.229E-02	8	C3a, C3dg, iC3b, C3, C3b, C3c, TPH2, FADD
32	Molluscum Contagiosum	6	1.032E-03	3.621E-02	1.032E-03	3.621E-02	3	Caspase-8, p18 Casp-8, FADD
33	Pigmented Nodular Adrenocortical Disease, Primary	6	1.032E-03	3.621E-02	1.032E-03	3.621E-02	3	ESR2 (mitochondrial), ESR2 (membrane), ESR2
34	Hyperparathyroidism, Secondary	6	1.032E-03	3.621E-02	1.032E-03	3.621E-02	3	p21, Parathyroid hormone, p18
35	Lipid Metabolism Disorders	211	1.160E-03	3.954E-02	1.160E-03	3.954E-02	18	C3a, C3dg, iC3b, C3, C3b, C3c, DAB2, HTR1A, CYP2C9, LCAT, GPR50, ACADM, HSD11B1, FGFR1, Autophagin-1, A-FABP, APOA1, Beta-3 adrenergic receptor
36	Pancreatic Diseases	1268	1.278E-03	4.234E-02	1.278E-03	4.234E-02	68	beta-ENaC, Cathepsin B, p21, IP3R2, CacyBP(SIP), Securin, C3a, C3dg, iC3b, C3, C3b, C3c, DAB2, ATR, IL1RN, CD4, WNT7B, PU.1, IL8RB, COL4A6, GRK5, CYP2C9, PAK3, Collagen XXIII, SOX9, G3P2, GATA-3, FUT4, B4G6, jBid, Bid, tBid, Alpha 1-antitrypsin, FZD2, SSTR5, GSTM1, CD1c, WNT5A, GDI2, FGFR1, MLH3, GAD2, NR2D, Caspase-8, p18 Casp-8, TANK, ASCT1 (SLC1A4), Bone sialoprotein, FosB, PANK3, CLCN2, ATP12A, FAF1, Adenylate cyclase type II, NGFR (CTF), NGFR(TNFRSF16), NGFR (ICD), PC2 (SPC2), FADD, GPR81, DHFR, ABCC5, CaSR, TRUNDD(TNFRSF10D), CCR6, ID1, APOA1, Beta-3 adrenergic receptor
37	Macular Degeneration	70	1.319E-03	4.254E-02	1.319E-03	4.254E-02	9	LAMG1, C3a, C3dg, iC3b, C3, C3b, C3c, HTR1A, GSTM1
38	Nutrition Disorders	526	1.572E-03	4.794E-02	1.572E-03	4.794E-02	34	C3a, C3dg, iC3b, C3, C3b, C3c, IL1RN, CD4, PU.1, Parathyroid hormone, IP3R3, AZGP1, Gas6, p18, CYP2C9, BHMT, LIPG, Aquaporin 7, ACADM, HSD11B1, FGFR1, GAD2, Caspase-8, p18 Casp-8, Adenylate cyclase type II, GSTK1, Alpha-2A adrenergic receptor, CaSR, A-FABP, APOA1, Beta-3 adrenergic receptor, ESR2 (mitochondrial), ESR2 (membrane), ESR2
39	Hyperlipidemias	132	1.577E-03	4.794E-02	1.577E-03	4.794E-02	13	C3a, C3dg, iC3b, C3, C3b, C3c, DAB2, CYP2C9, GPR50, FGFR1, Autophagin-1, APOA1, Beta-3 adrenergic receptor
40	Eye Diseases	847	1.608E-03	4.794E-02	1.608E-03	4.794E-02	49	IFNA1, LAMG1, Cathepsin B, p21, AP3M1, IFNA2, C3a, C3dg, iC3b, C3, C3b, C3c, APRIL(TNFSF13), IFNA16, GALE, IL1RN, CD4, IP3R3, HTR1A, IL8RB, Gas6, COL4A6, CYP2C9, LCAT, RIPK2, TYRP2, GATA-3, Alpha 1-antitrypsin, p130, SSTR5, GSTM1, CD1c, CCR4, WNT5A, FGFR1, Caspase-8, p18 Casp-8, CD8 alpha, Epo receptor, NGFR (CTF), NGFR(TNFRSF16), NGFR (ICD), IFNA21, ID1, APOA1, Beta-3 adrenergic receptor, ESR2 (mitochondrial), ESR2 (membrane), ESR2
41	Thrombotic Microangiopathies	61	2.142E-03	6.231E-02	2.142E-03	6.231E-02	8	C3a, C3dg, iC3b, C3, C3b, C3c, IL1RN, CD20
42	Mycoses	49	2.457E-03	6.957E-02	2.457E-03	6.957E-02	7	C3a, C3dg, iC3b, C3, C3b, C3c, AIRE
43	Thyroid Diseases	520	2.508E-03	6.957E-02	2.508E-03	6.957E-02	33	p21, Securin, C3a, C3dg, iC3b, C3, C3b, C3c, IL1RN, AIRE, p18, Collagen XXIII, G3P2, PTHR1, SSTR5, GSTM1, CD1c, WNT5A, FGFR1, Caspase-8, p18 Casp-8, Bone sialoprotein, FosB, HEM2, Epo receptor, NGFR (CTF), NGFR(TNFRSF16), NGFR (ICD), PC2 (SPC2), ID1, ESR2 (mitochondrial), ESR2 (membrane), ESR2
44	Rhabdoviridae Infections	8	2.729E-03	7.077E-02	2.729E-03	7.077E-02	3	NGFR (CTF), NGFR(TNFRSF16), NGFR (ICD)
45	Hip Injuries	8	2.729E-03	7.077E-02	2.729E-03	7.077E-02	3	ESR2 (mitochondrial), ESR2 (membrane), ESR2
46	Rabies	8	2.729E-03	7.077E-02	2.729E-03	7.077E-02	3	NGFR (CTF), NGFR(TNFRSF16), NGFR (ICD)

Supplemental Table 6. DR shRNA screen-enriched diseases (biomarkers)

47	Parathyroid Diseases	38	2.980E-03	7.554E-02	2.980E-03	7.554E-02	6	p21, Parathyroid hormone, AIRE, p18, GATA-3, CaSR
48	Movement Disorders	444	3.061E-03	7.554E-02	3.061E-03	7.554E-02	29	FEN1, MR1, TPH2, PNKD, CD4, HTR1A, GRK5, CYP2C9, TGM1, G3P2, ACTA1, FOXP1, GSTM1, GDI2, HNMT, GAD2, NR2D, Caspase-8, p18 Casp-8, HEM2, NGFR (CTF), NGFR(TNFRSF16), NGFR (ICD), Alpha-2A adrenergic receptor, FADD, ESR2 (mitochondrial), ESR2 (membrane), ESR2, TDO2
49	Limb Deformities, Congenital	51	3.103E-03	7.554E-02	3.103E-03	7.554E-02	7	jBid, Bid, tBid, GSTM1, FGFR1, Caspase-8, p18 Casp-8
50	Thyroid Neoplasms	405	3.216E-03	7.674E-02	3.216E-03	7.674E-02	27	p21, Securin, C3a, C3dg, iC3b, C3, C3b, C3c, IL1RN, p18, Collagen XXIII, G3P2, PTHR1, SSTR5, GSTM1, WNT5A, Bone sialoprotein, FosB, Epo receptor, NGFR (CTF), NGFR(TNFRSF16), NGFR (ICD), PC2 (SPC2), ID1, ESR2 (mitochondrial), ESR2 (membrane), ESR2

Supplemental Table 6. DR shRNA screen-enriched diseases (biomarkers)

FAS and TNF Diseases (by Biomarkers)								
#	Diseases	Total	pValue	Min FDR	p-value	FDR	In Data	Network Objects from Active Data
1	Encephalomyelitis, Autoimmune, Experimental	12	1.351E-08	5.150E-06	1.351E-08	5.150E-06	7	C3a, C3dg, iC3b, C3, C3b, C3c, FADD
2	Nervous System Autoimmune Disease, Experimental	12	1.351E-08	5.150E-06	1.351E-08	5.150E-06	7	C3a, C3dg, iC3b, C3, C3b, C3c, FADD
3	Pneumonia, Pneumocystis	8	1.782E-08	5.150E-06	1.782E-08	5.150E-06	6	C3a, C3dg, iC3b, C3, C3b, C3c
4	Pneumocystis Infections	8	1.782E-08	5.150E-06	1.782E-08	5.150E-06	6	C3a, C3dg, iC3b, C3, C3b, C3c
5	Complement Factor I Deficiency	10	1.271E-07	2.939E-05	1.271E-07	2.939E-05	6	C3a, C3dg, iC3b, C3, C3b, C3c
6	Purpura, Thrombotic Thrombocytopenic	12	5.319E-07	9.721E-05	5.319E-07	9.721E-05	6	C3a, C3dg, iC3b, C3, C3b, C3c
7	Dermatitis, Atopic	205	6.123E-07	9.721E-05	6.123E-07	9.721E-05	21	p21, C3a, C3dg, iC3b, C3, C3b, C3c, APRIL(TNFSF13), IL1RN, CD20, PDE4B, HTR1A, GATA-3, GSTM1, CysLT1 receptor, CCR4, Fc epsilon RI beta, NGFR (CTF), NGFR(TNFRSF16), NGFR (ICD), TLR8
8	Lupus Erythematosus, Systemic	556	6.727E-07	9.721E-05	6.727E-07	9.721E-05	38	IFNA1, p21, IFNA2, C3a, C3dg, iC3b, C3, C3b, C3c, APRIL(TNFSF13), IFNA16, IL1RN, CD4, IP3R3, HTR1A, IL8RB, Gas6, CYP2C9, RIPK2, GATA-3, GITR, jBid, Bid, tBid, GSTM1, PKR, CD8 alpha, FAF1, Fc epsilon RI beta, TLR8, FADD, CNK3B (IPCEF1), IFNA21, Bcl-G, APOA1, ESR2 (mitochondrial), ESR2 (membrane), ESR2
9	Lung Diseases, Fungal	13	9.634E-07	1.237E-04	9.634E-07	1.237E-04	6	C3a, C3dg, iC3b, C3, C3b, C3c
10	Dermatitis	351	1.336E-06	1.545E-04	1.336E-06	1.545E-04	28	p21, C3a, C3dg, iC3b, C3, C3b, C3c, APRIL(TNFSF13), IL1RN, CD4, CD20, PDE4B, HTR1A, CYP2C9, ATP6V1F (VATF), TGM1, GATA-3, GSTM1, CysLT1 receptor, CCR4, Caspase-8, p18 Casp-8, Fc epsilon RI beta, NGFR (CTF), NGFR(TNFRSF16), NGFR (ICD), TLR8, CCR6
11	Skin Diseases, Genetic	314	1.672E-06	1.757E-04	1.672E-06	1.757E-04	26	p21, AP3M1, C3a, C3dg, iC3b, C3, C3b, C3c, APRIL(TNFSF13), IL1RN, CD20, PDE4B, HTR1A, TGM1, GATA-3, GSTM1, CysLT1 receptor, CCR4, HEM2, Fc epsilon RI beta, SNAP-29, NGFR (CTF), NGFR(TNFRSF16), NGFR
12	Skin Diseases, Eczematous	325	3.201E-06	3.083E-04	3.201E-06	3.083E-04	26	p21, C3a, C3dg, iC3b, C3, C3b, C3c, APRIL(TNFSF13), IL1RN, CD20, PDE4B, HTR1A, ATP6V1F (VATF), TGM1, GATA-3, GSTM1, CysLT1 receptor, CCR4, Caspase-8, p18 Casp-8, Fc epsilon RI beta, NGFR (CTF), NGFR(TNFRSF16), NGFR (ICD), TLR8, CCR6
13	Drug-Induced Liver Injury, Chronic	24	4.351E-06	3.593E-04	4.351E-06	3.593E-04	7	C3a, C3dg, iC3b, C3, C3b, C3c, GSTM1
14	Drug-Induced Liver Injury	24	4.351E-06	3.593E-04	4.351E-06	3.593E-04	7	C3a, C3dg, iC3b, C3, C3b, C3c, GSTM1
15	Cicatrix, Hypertrophic	17	6.286E-06	4.844E-04	6.286E-06	4.844E-04	6	C3a, C3dg, iC3b, C3, C3b, C3c
16	Dyslipidemias	151	9.528E-06	6.742E-04	9.528E-06	6.742E-04	16	C3a, C3dg, iC3b, C3, C3b, C3c, DAB2, HTR1A, CYP2C9, LCAT, GPR50, FGFR1, Autophagin-1, A-FABP, APOA1, Beta-3 adrenergic receptor
17	Eye Diseases	847	9.914E-06	6.742E-04	9.914E-06	6.742E-04	47	IFNA1, LAMG1, Cathepsin B, p21, AP3M1, IFNA2, C3a, C3dg, iC3b, C3, C3b, C3c, APRIL(TNFSF13), IFNA16, IL1RN, CD4, IP3R3, HTR1A, IL8RB, Gas6, COL4A6, CYP2C9, LCAT, RIPK2, TYRP2, GATA-3, Alpha 1-antitrypsin, p130, SSTR5, GSTM1, CD1c, CCR4, WNT5A, FGFR1, Caspase-8, p18 Casp-8, CD8 alpha, Epo receptor, NGFR (CTF), NGFR(TNFRSF16), NGFR (ICD), IFNA21, APOA1, Beta-3 adrenergic receptor, ESR2 (mitochondrial), ESR2
18	Hypertriglyceridemia	62	1.263E-05	8.109E-04	1.263E-05	8.109E-04	10	C3a, C3dg, iC3b, C3, C3b, C3c, GPR50, Autophagin-1, APOA1, Beta-3 adrenergic receptor
19	Pancreatic Diseases	1268	1.420E-05	8.637E-04	1.420E-05	8.637E-04	62	beta-ENaC, Cathepsin B, p21, IP3R2, CacyBP(SIP), Securin, C3a, C3dg, iC3b, C3, C3b, C3c, DAB2, ATR, IL1RN, CD4, WNT7B, PU.1, IL8RB, COL4A6, GRK5, CYP2C9, PAK3, SOX9, G3P2, GATA-3, FUT4, B4G6, jBid, Bid, tBid, Alpha 1-antitrypsin, FZD2, SSTR5, GSTM1, CD1c, WNT5A, GDI2, FGFR1, MLH3, GAD2, NR2D, Caspase-8, p18 Casp-8, TANK, Bone sialoprotein, FosB, CLCN2, FAF1, Adenylate cyclase type II, NGFR (CTF), NGFR(TNFRSF16), NGFR (ICD), PC2 (SPC2), FADD, GPR81, DHFR, CaSR, TRUNDD(TNFRSF10D), CCR6, APOA1, Beta-3 adrenergic
20	Encephalitis	80	2.224E-05	1.257E-03	2.224E-05	1.257E-03	11	IFNA1, C3a, C3dg, iC3b, C3, C3b, C3c, IL1RN, PKR, CD8 alpha, FADD

Supplemental Table 6. DR shRNA screen-enriched diseases (biomarkers)

21	Purpura, Thrombocytopenic	41	2.283E-05	1.257E-03	2.283E-05	1.257E-03	8	C3a, C3dg, iC3b, C3, C3b, C3c, IL1RN, CD20
22	Pathological Conditions, Signs and Symptoms	2107	2.440E-05	1.282E-03	2.440E-05	1.282E-03	89	IFNA1, LAMG1, beta-ENaC, FEN1, PRKCSH, Cathepsin B, p21, MutYH, ATR/TEM8, Securin, C3a, C3dg, iC3b, C3, C3b, C3c, APRIL(TNFSF13), ATR, IL1RN, CD4, CD20, PU.1, IP3R3, AZGP1, AIRE, HTR1A, IL8RB, Gas6, p18, COL4A6, CYP2C9, PAK3, LCAT, SOX9, RIPK2, G3P2, LIPG, Casein kinase I gamma 2, GATA-3, ACTA1, Glutaredoxin 1, GITR, Aquaporin 7, jBid, Bid, tBid, Alpha 1-antitrypsin, ERK5 (MAPK7), PLA2G2D, FOXP1, ACADM, HSD11B1, GSTM1, 14-3-3 gamma, GAMT, WNT5A, GDI2, FGFR1, GAD2, Caspase-8, p18 Casp-8, TANK, Bone sialoprotein, FosB, PKR, CD8 alpha, CLCN2, MT-TRX, Fc epsilon RI beta, Epo receptor, Adenylate cyclase type II, NGFR (CTF), NGFR(TNFRSF16), NGFR (ICD), PC2 (SPC2), WASF1(WAVE1), Alpha-2A adrenergic receptor, FADD, DHFR, A-FABP, CaSR, TRUNDD(TNFRSF10D), Delta-type opioid receptor, CCR6, APOA1, Beta-3 adrenergic receptor,
23	Poisoning	82	2.822E-05	1.418E-03	2.822E-05	1.418E-03	11	MutYH, C3a, C3dg, iC3b, C3, C3b, C3c, HTR1A, GSTM1, HEM2, Delta-type opioid receptor
24	Hyperlipidemia, Familial Combined	32	3.455E-05	1.664E-03	3.455E-05	1.664E-03	7	C3a, C3dg, iC3b, C3, C3b, C3c, APOA1
25	Autoimmune Diseases	1248	3.686E-05	1.704E-03	3.686E-05	1.704E-03	60	IFNA1, Cathepsin B, p21, IP3R2, IFNA2, C3a, C3dg, iC3b, C3, C3b, C3c, APRIL(TNFSF13), IFNA16, IL1RN, CD4, XAB2, CD20, IP3R3, PDE4B, AIRE, HTR1A, IL8RB, Gas6, CYP2C9, LCAT, RIPK2, TYRP2, GATA-3, GITR, jBid, Bid, tBid, Alpha 1-antitrypsin, PLA2G2D, SSTR5, GSTM1, CD1c, CCR4, WNT5A, FXYD2, GAD2, Caspase-8, p18 Casp-8, PKR, CD8 alpha, FAF1, Fc epsilon RI beta, TLR8, FADD, CNK3B (IPCEF1), TRUNDD(TNFRSF10D), Delta-type opioid receptor, IFNA21, Bcl-G, CCR6, APOA1, Beta-3 adrenergic receptor, ESR2 (mitochondrial), ESR2 (membrane), ESR2
26	Lipid Metabolism Disorders	211	5.206E-05	2.315E-03	5.206E-05	2.315E-03	18	C3a, C3dg, iC3b, C3, C3b, C3c, DAB2, HTR1A, CYP2C9, LCAT, GPR50, ACADM, HSD11B1, FGFR1, Autophagin-1, A-FABP, APOA1, Beta-3 adrenergic receptor
27	Connective Tissue Diseases	1073	6.474E-05	2.772E-03	6.474E-05	2.772E-03	53	IFNA1, Cathepsin B, p21, IP3R2, IFNA2, C3a, C3dg, iC3b, C3, C3b, C3c, APRIL(TNFSF13), IFNA16, IL1RN, CD4, CD20, IP3R3, AIRE, HTR1A, IL8RB, Gas6, COL4A6, CYP2C9, LCAT, RIPK2, GATA-3, GITR, jBid, Bid, tBid, Alpha 1-antitrypsin, PLA2G2D, GSTM1, CCR4, WNT5A, Caspase-8, p18 Casp-8, PKR, CD8 alpha, FAF1, Fc epsilon RI beta, Collagen VII, TLR8, FADD, CNK3B (IPCEF1), IFNA21, Bcl-G, CCR6, APOA1, Beta-3 adrenergic receptor, ESR2 (mitochondrial), ESR2 (membrane), ESR2
28	Hemolytic-Uremic Syndrome	25	7.365E-05	3.041E-03	7.365E-05	3.041E-03	6	C3a, C3dg, iC3b, C3, C3b, C3c
29	Immune System Diseases	2398	8.863E-05	3.533E-03	8.863E-05	3.533E-03	96	IFNA1, Nocturnin, LAMG1, FEN1, Cathepsin B, p21, IP3R2, ATR/TEM8, Securin, IFNA2, C3a, C3dg, iC3b, C3, C3b, C3c, APRIL(TNFSF13), IFNA16, ATR, IL1RN, CD4, XAB2, CD20, PU.1, IP3R3, PDE4B, AZGP1, AIRE, HTR1A, IL8RB, Gas6, FZD10, p18, COL4A6, PLD1, CYP2C9, LCAT, ATP6V1F (VATF), RIPK2, TYRP2, GATA-3, DVL-3, ACTA1, eIF4A1, FUT4, 5'-NTD, GITR, jBid, Bid, tBid, Alpha 1-antitrypsin, ERK5 (MAPK7), FZD2, iASPP, p130, PLA2G2D, FOXP1, SSTR5, HSD11B1, GSTM1, CysLT1 receptor, CD1c, CCR4, WNT5A, FGFR1, FXYD2, MLH3, GAD2, Caspase-8, p18 Casp-8, Bone sialoprotein, PKR, CD8 alpha, FAF1, Fc epsilon RI beta, Epo receptor, Adenylate cyclase type II, NGFR (CTF), NGFR(TNFRSF16), NGFR (ICD), PC2 (SPC2), TLR8, FADD, CDK9, CNK3B (IPCEF1), DHFR, TRUNDD(TNFRSF10D), Delta-type opioid receptor, IFNA21, Bcl-G, CCR6, APOA1, Beta-3 adrenergic receptor, ESR2
30	Foot Deformities	18	1.432E-04	4.919E-03	1.432E-04	4.919E-03	5	jBid, Bid, tBid, Caspase-8, p18 Casp-8
31	Clubfoot	18	1.432E-04	4.919E-03	1.432E-04	4.919E-03	5	jBid, Bid, tBid, Caspase-8, p18 Casp-8
32	Foot Deformities, Congenital	18	1.432E-04	4.919E-03	1.432E-04	4.919E-03	5	jBid, Bid, tBid, Caspase-8, p18 Casp-8
33	Lower Extremity Deformities, Congenital	18	1.432E-04	4.919E-03	1.432E-04	4.919E-03	5	jBid, Bid, tBid, Caspase-8, p18 Casp-8
34	Hyperlipidemias	132	1.447E-04	4.919E-03	1.447E-04	4.919E-03	13	C3a, C3dg, iC3b, C3, C3b, C3c, DAB2, CYP2C9, GPR50, FGFR1, Autophagin-1, APOA1, Beta-3 adrenergic receptor
35	Nutrition Disorders	526	1.611E-04	5.274E-03	1.611E-04	5.274E-03	31	C3a, C3dg, iC3b, C3, C3b, C3c, IL1RN, CD4, PU.1, IP3R3, AZGP1, Gas6, p18, CYP2C9, LIPG, Aquaporin 7, ACADM, HSD11B1, FGFR1, GAD2, Caspase-8, p18 Casp-8, Adenylate cyclase type II, Alpha-2A adrenergic receptor, A-FABP, CaSR, APOA1, Beta-3 adrenergic receptor, ESR2 (mitochondrial), ESR2 (membrane), ESR2

36	Lung Diseases, Obstructive	835	1.642E-04	5.274E-03	1.642E-04	5.274E-03	43	IFNA1, LAMG1, Cathepsin B, p21, ATR/TEM8, C3a, C3dg, iC3b, C3, C3b, C3c, IL1RN, CD20, PDE4B, AZGP1, IL8RB, Gas6, COL4A6, SOX9, RIPK2, GATA-3, DVL-3, ACTA1, FUT4, 5'-NTD, Alpha 1-antitrypsin, PLA2G2D, GSTM1, CysLT1 receptor, CCR4, FGFR1, Caspase-8, p18 Casp-8, CD8 alpha, Fc epsilon RI beta, Adenylate cyclase type II, nAChR alpha-3, TLR8, CNK3B (IPCEF1), A-FABP, Bcl-G,
37	Macular Degeneration	70	2.147E-04	6.709E-03	2.147E-04	6.709E-03	9	LAMG1, C3a, C3dg, iC3b, C3, C3b, C3c, HTR1A, GSTM1
38	Body Weight	513	2.404E-04	7.312E-03	2.404E-04	7.312E-03	30	C3a, C3dg, iC3b, C3, C3b, C3c, IL1RN, CD4, PU.1, IP3R3, AZGP1, Gas6, p18, CYP2C9, LIPG, Aquaporin 7, PLA2G2D, HSD11B1, FGFR1, GAD2, FosB, Adenylate cyclase type II, Alpha-2A adrenergic receptor, A-FABP, CaSR, APOA1, Beta-3 adrenergic receptor, ESR2 (mitochondrial), ESR2 (membrane), ESR2
39	Body Weights and Measures	492	2.713E-04	7.974E-03	2.713E-04	7.974E-03	29	C3a, C3dg, iC3b, C3, C3b, C3c, IL1RN, CD4, PU.1, IP3R3, AZGP1, Gas6, p18, CYP2C9, LIPG, Aquaporin 7, PLA2G2D, HSD11B1, FGFR1, GAD2, Adenylate cyclase type II, Alpha-2A adrenergic receptor, A-FABP, CaSR, APOA1, Beta-3 adrenergic receptor, ESR2 (mitochondrial), ESR2
40	Congenital, Hereditary, and Neonatal Diseases and Abnormalities	1786	2.759E-04	7.974E-03	2.759E-04	7.974E-03	75	Coagulation factor IX, Nocturnin, beta-ENaC, FEN1, Cathepsin B, p21, AP3M1, MutYH, C3a, C3dg, iC3b, C3, C3b, C3c, APRIL(TNFSF13), ATR, IL1RN, CD4, CD20, PU.1, PDE4B, AIRE, HTR1A, IL8RB, COL4A6, GRK5, CYP2C9, LCAT, TGM1, SOX9, G3P2, GATA-3, FUT4, jBid, Bid, tBid, Alpha 1-antitrypsin, FOXP1, ACADM, HSD11B1, GSTM1, CysLT1 receptor, CD1c, CCR4, 14-3-3 gamma, GAMT, WNT5A, GDI2, FGFR1, MLH3, NR2D, Caspase-8, p18 Casp-8, Bone sialoprotein, PKR, CLCN2, NODAL, HEM2, Fc epsilon RI beta, Adenylate cyclase type II, nAChR alpha-3, SNAP-29, NGFR (CTF), NGFR(TNFRSF16), NGFR (ICD), Collagen VII, TLR8, Alpha-2A adrenergic receptor, MMAB, DHFR, APOA1, Beta-3 adrenergic receptor, ESR2
41	Thyroid Carcinoma, Follicular	58	2.967E-04	8.293E-03	2.967E-04	8.293E-03	8	C3a, C3dg, iC3b, C3, C3b, C3c, IL1RN, FosB
42	Thyroid Diseases	520	3.048E-04	8.293E-03	3.048E-04	8.293E-03	30	p21, Securin, C3a, C3dg, iC3b, C3, C3b, C3c, IL1RN, AIRE, p18, G3P2, SSTR5, GSTM1, CD1c, WNT5A, FGFR1, Caspase-8, p18 Casp-8, Bone sialoprotein, FosB, HEM2, Epo receptor, NGFR (CTF), NGFR(TNFRSF16), NGFR (ICD), PC2 (SPC2), ESR2 (mitochondrial), ESR2
43	Pancreatic Neoplasms	1080	3.085E-04	8.293E-03	3.085E-04	8.293E-03	51	Cathepsin B, p21, IP3R2, CacyBP(SIP), Securin, C3a, C3dg, iC3b, C3, C3b, C3c, DAB2, ATR, WNT7B, IL8RB, COL4A6, PAK3, SOX9, G3P2, GATA-3, FUT4, B4G6, jBid, Bid, tBid, Alpha 1-antitrypsin, FZD2, SSTR5, CD1c, WNT5A, GDI2, FGFR1, MLH3, GAD2, NR2D, Caspase-8, p18 Casp-8, TANK, Bone sialoprotein, FosB, FAF1, NGFR (CTF), NGFR(TNFRSF16), NGFR (ICD), PC2 (SPC2), FADD, GPR81, DHFR, TRUNDD(TNFRSF10D), CCR6, APOA1
44	Lupus Nephritis	146	3.956E-04	1.019E-02	3.956E-04	1.019E-02	13	p21, C3a, C3dg, iC3b, C3, C3b, C3c, HTR1A, Gas6, CYP2C9, GATA-3, TLR8, FADD
45	Signs and Symptoms	1206	4.029E-04	1.019E-02	4.029E-04	1.019E-02	55	IFNA1, beta-ENaC, p21, ATR/TEM8, C3a, C3dg, iC3b, C3, C3b, C3c, ATR, IL1RN, CD4, CD20, PU.1, IP3R3, AZGP1, HTR1A, Gas6, p18, CYP2C9, PAK3, SOX9, LIPG, Casein kinase I gamma 2, GATA-3, Aquaporin 7, jBid, Bid, tBid, ERK5 (MAPK7), PLA2G2D, ACADM, HSD11B1, GSTM1, GAMT, FGFR1, GAD2, Caspase-8, p18 Casp-8, FosB, MT-TRX, Fc epsilon RI beta, Epo receptor, Adenylate cyclase type II, Alpha-2A adrenergic receptor, FADD, A-FABP, CaSR, Delta-type opioid receptor, APOA1, Beta-3 adrenergic receptor, ESR2 (mitochondrial), ESR2 (membrane), ESR2
46	Thrombotic Microangiopathies	61	4.223E-04	1.019E-02	4.223E-04	1.019E-02	8	C3a, C3dg, iC3b, C3, C3b, C3c, IL1RN, CD20
47	Obesity	456	4.232E-04	1.019E-02	4.232E-04	1.019E-02	27	C3a, C3dg, iC3b, C3, C3b, C3c, IL1RN, CD4, PU.1, IP3R3, AZGP1, Gas6, p18, CYP2C9, LIPG, Aquaporin 7, HSD11B1, FGFR1, GAD2, Adenylate cyclase type II, Alpha-2A adrenergic receptor, A-FABP, APOA1, Beta-3 adrenergic receptor, ESR2 (mitochondrial), ESR2 (membrane), ESR2
48	Overnutrition	456	4.232E-04	1.019E-02	4.232E-04	1.019E-02	27	C3a, C3dg, iC3b, C3, C3b, C3c, IL1RN, CD4, PU.1, IP3R3, AZGP1, Gas6, p18, CYP2C9, LIPG, Aquaporin 7, HSD11B1, FGFR1, GAD2, Adenylate cyclase type II, Alpha-2A adrenergic receptor, A-FABP, APOA1, Beta-3 adrenergic receptor, ESR2 (mitochondrial), ESR2 (membrane), ESR2
49	Body Constitution	506	4.362E-04	1.029E-02	4.362E-04	1.029E-02	29	C3a, C3dg, iC3b, C3, C3b, C3c, IL1RN, CD4, PU.1, IP3R3, AZGP1, Gas6, p18, CYP2C9, LIPG, Aquaporin 7, PLA2G2D, HSD11B1, FGFR1, GAD2, Adenylate cyclase type II, Alpha-2A adrenergic receptor, A-FABP, CaSR, APOA1, Beta-3 adrenergic receptor, ESR2 (mitochondrial), ESR2

Supplemental Table 6. DR shRNA screen-enriched diseases (biomarkers)

50	Cystic Fibrosis	296	4.917E-04	1.085E-02	4.917E-04	1.085E-02	20	beta-ENaC, Cathepsin B, C3a, C3dg, iC3b, C3, C3b, C3c, IL1RN, CD4, PU.1, IL8RB, COL4A6, GRK5, Alpha 1-antitrypsin, GSTM1, NR2D, CLCN2, Adenylate cyclase type II, Beta-3 adrenergic receptor

Komarov et al. Functional genetics-directed identification of novel pharmacological inhibitors of FAS- and TNF-dependent apoptosis that protect mice from acute liver failure

Supplemental Table 7:

DR shRNA screen functional interaction modules

Supplemental Table 7. DR shRNA screen functional interaction modules

Sub Cluster Pathways						
**Cluster**	**Enriched Pathways**	**Number of Genes in Module**	**# Hits**	**P-value**	**FDR**	**Gene Hits**
0	Regulation of cytoplasmic and nuclear SMAD2/3 signaling(N)	289	22	0	<3.33e-04	CTNNB1,GATA3,PPP1CA,JUN,MAPK3,TGIF1,STMN1,STRAP,CREBBP,SMAD2,CDKN1A,PPARG,EGR1,AR,CTBP1,RBBP4,TP53,ESR1,BCL2,NFATC2,HSPA8,SP1
0	Regulation of nuclear SMAD2/3 signaling(N)	289	22	0	<3.33e-04	CTNNB1,GATA3,PPP1CA,JUN,MAPK3,TGIF1,STMN1,STRAP,CREBBP,SMAD2,CDKN1A,PPARG,EGR1,AR,CTBP1,RBBP4,TP53,ESR1,BCL2,NFATC2,HSPA8,SP1
0	TGF-beta receptor signaling(N)	289	22	0	<3.33e-04	CTNNB1,GATA3,PPP1CA,JUN,MAPK3,TGIF1,STMN1,STRAP,CREBBP,SMAD2,CDKN1A,PPARG,EGR1,AR,CTBP1,RBBP4,TP53,ESR1,BCL2,NFATC2,HSPA8,SP1
0	IFN-gamma pathway(N)	373	24	0	<2.50e-04	CTNNB1,GATA3,PPP1CA,JUN,MAPK3,TGIF1,STMN1,STRAP,CREBBP,SMAD2,CDKN1A,GTF3A,PPARG,SPI1,EGR1,AR,CTBP1,RBBP4,TP53,ESR1,BCL2,NFATC2,HSPA8,SP1
0	Retinoic acid receptors-mediated signaling(N)	47	10	0	<2.00e-04	RARA,RXRA,MAPK3,NR1H2,CREBBP,PPARA,PPARD,PPARG,CDK7,BCL2
0	Glypican 1 network(N)	436	24	0	<1.67e-04	CTNNB1,GATA3,PPP1CA,JUN,MAPK3,TGIF1,STMN1,ARFGAP1,STRAP,ELK1,CREBBP,SMAD2,CDKN1A,PPARG,EGR1,AR,CTBP1,RBBP4,TP53,ESR1,BCL2,NFATC2,HSPA8,SP1
0	Cell cycle(K)	128	13	0	<1.25e-04	PTTG1,CDKN2C,RBL2,CREBBP,SMAD2,ATR,CDKN1A,PLK1,PCNA,CDC14A,TP53,MCM2,CDK7
0	TGFBR(C)	128	13	0	<1.25e-04	CTNNB1,RBL2,JUN,TGIF1,STRAP,CREBBP,SMAD2,CDKN1A,AR,TP53,ESR1,HSPA8,SP1
0	Glypican pathway(N)	472	24	0	<1.11e-04	CTNNB1,GATA3,PPP1CA,JUN,MAPK3,TGIF1,STMN1,ARFGAP1,STRAP,ELK1,CREBBP,SMAD2,CDKN1A,PPARG,EGR1,AR,CTBP1,RBBP4,TP53,ESR1,BCL2,NFATC2,HSPA8,SP1
0	RXR and RAR heterodimerization with other nuclear receptor(N)	26	7	0	<1.00e-04	RARA,RXRA,NR1H2,PPARA,PPARD,PPARG,BCL2
0	AndrogenReceptor(C)	72	9	0	9.09E-05	CTNNB1,JUN,TGIF1,CREBBP,GTF2F2,AR,ESR1,CDK9,SP1
0	Pathways in cancer(K)	327	18	0	8.33E-05	CTNNB1,RARA,RXRA,HIF1A,JUN,MAPK3,CREBBP,SMAD2,CDKN1A,PPARD,PPARG,SPI1,MLH1,PAX8,AR,CTBP1,TP53,BCL2
0	mechanism of gene regulation by peroxisome proliferators via ppara(B)	40	7	0	7.69E-05	RXRA,JUN,MAPK3,CREBBP,FABP1,PPARA,SP1
0	Hypoxic and oxygen homeostasis regulation of HIF-1-alpha(N)	80	9	0	7.14E-05	LDHA,BHLHB3,HIF1A,JUN,CREBBP,NT5E,TP53,SP1,SP3
0	Regulation of retinoblastoma protein(N)	61	8	0	6.67E-05	JUN,TAF1,CREBBP,CDKN1A,PPARG,SPI1,CTBP1,RBBP4
0	Presenilin action in Notch and Wnt signaling(N)	43	7	0	6.25E-05	CTNNB1,JUN,MAPK3,CREBBP,NOTCH1,PPARD,CTBP1
0	Thyroid cancer(K)	29	6	0	1.18E-04	CTNNB1,RXRA,MAPK3,PPARG,PAX8,TP53
0	HIF-1-alpha transcription factor network(N)	66	8	0	1.11E-04	LDHA,BHLHB3,HIF1A,JUN,CREBBP,NT5E,SP1,SP3
0	PPAR signaling pathway(K)	69	8	0	1.58E-04	AQP7,CYP7A1,RXRA,FABP1,PPARA,PPARD,PPARG,ACADM
0	TGF-beta signaling pathway(P)	94	9	0	2.00E-04	NODAL,JUN,MAPK3,BMP1,CREBBP,SMAD2,ACVR1B,INHBB,ACVR1
0	FOXA transcription factor networks(N)	75	8	0	4.76E-04	JUN,CREBBP,AR,ACADM,ESR1,SP1,SP3,NFIC
0	hypoxia and p53 in the cardiovascular system(B)	22	5	0	5.00E-04	HIF1A,MAPK3,TAF1,CDKN1A,TP53
0	p53 signaling pathway(B)	12	4	0	6.09E-04	CDKN1A,PCNA,TP53,BCL2
0	Transcription(R)	136	10	0	6.67E-04	MAPK3,TAF1,CSTF3,GTF2F2,GTF3A,CDK9,CDK7,GTF3C3,POLR3A,NFIC
0	DNA Repair(R)	85	8	0	6.92E-04	MUTYH,H2AFX,XAB2,PCNA,CDK7,RPA2,FEN1,MPG
0	TGF-beta signaling pathway(K)	85	8	0	6.92E-04	RBL2,NODAL,MAPK3,CREBBP,SMAD2,INHBB,ACVR1,SP1
0	Colorectal cancer(K)	62	7	0	6.67E-04	CTNNB1,JUN,MAPK3,SMAD2,MLH1,TP53,BCL2
0	FOXA1 transcription factor network(N)	42	6	0	6.43E-04	JUN,CREBBP,AR,ESR1,SP1,NFIC
0	Role of Calcineurin-dependent NFAT signaling in lymphocytes(N)	88	8	0	7.24E-04	GATA3,JUN,MAPK3,CREBBP,PPARG,EGR1,BCL2,NFATC2
0	Regulation of Telomerase(N)	67	7	0.0001	9.33E-04	JUN,MAPK3,HUS1,RBBP4,ESR1,SP1,SP3
1	Jak-STAT signaling pathway(K)	155	19	0	<1.00e-03	STAT5A,AKT1,IL15RA,CSF2RB,IL13RA2,IL22,IFNAR1,GRB2,STAT6,IFNA2,IL10RA,IL2RG,PTPN6,IL9,CBL,STAT3,JAK2,EPOR,JAK3
1	ErbB signaling pathway(K)	87	15	0	<5.00e-04	HRAS,STAT5A,AKT1,PAK3,SHC2,SHC4,EGFR,PRKCG,NCK2,GRB2,MAP2K1,MAP2K2,CBL,RAF1,ABL2

Supplemental Table 7. DR shRNA screen functional interaction modules

1	TCR signaling in naïve CD8+ T cells(N)	127	17	0	<3.33e-04	HRAS,CD8A,AKT1,CSK,HLA-A,PRKCE,IFNAR1,GRB2,CD247,IFNA2,IL2RG,PTPN6,MAP2K1,MAP2K2,CBL,RAF1,CALM1
1	EGF receptor signaling pathway(P)	91	15	0	<2.50e-04	HRAS,STAT5A,AKT1,EGFR,PRKCG,PRKCE,GRB2,STAT6,RAC2,MAP2K1,MAP2K2,CBL,RAF1,STAT3,YWHAH
1	Signaling events mediated by Stem cell factor receptor (c-Kit)(N)	52	12	0	<2.00e-04	HRAS,STAT5A,AKT1,GRB2,PTPN6,MAP2K1,MAP2K2,CBL,RAF1,STAT3,JAK2,EPOR
1	role of erbb2 in signal transduction and oncology(B)	29	10	0	<1.67e-04	HRAS,CSK,EGFR,GRB2,MAP2K1,MAP2K2,RAF1,STAT3,JAK2,JAK3
1	Gap junction(K)	90	14	0	<1.43e-04	HRAS,GJA1,EGFR,PRKCG,PDGFRB,MAPK7,GRB2,MAP2K1,MAP2K2,RAF1,ITPR3,ITPR2,TUBA8,TJP1
1	Natural killer cell mediated cytotoxicity(K)	137	16	0	<1.25e-04	HRAS,KLRC3,SHC2,SHC4,HLA-A,PRKCG,NCR1,IFNAR1,GRB2,CD247,IFNA2,RAC2,PTPN6,MAP2K1,MAP2K2,RAF1
1	FGF signaling pathway(P)	102	14	0	<1.11e-04	AKT1,FRS2,PRKCG,PRKCE,FGFR1,FGFR4,GRB2,RAC2,PPP2R2C,PTPN6,MAP2K1,MAP2K2,RAF1,YWHAH
1	Glioma(K)	65	12	0	<9.09e-05	HRAS,AKT1,SHC2,SHC4,EGFR,PRKCG,PDGFRB,GRB2,MAP2K1,MAP2K2,RAF1,CALM1
1	Downstream signaling in naïve CD8+ T cells(N)	65	12	0	<9.09e-05	HRAS,CD8A,HLA-A,PRKCE,IFNAR1,CD247,IFNA2,IL2RG,MAP2K1,MAP2K2,RAF1,CALM1
1	Neurotrophin signaling pathway(K)	126	15	0	<8.33e-05	HRAS,AKT1,CSK,SHC2,FRS2,SHC4,MAPK7,GRB2,MAP2K1,MAP2K2,RAF1,YWHAG,YWHAH,RAP1B,CALM1
1	Neurotrophic factor-mediated Trk receptor signaling(N)	98	13	0	<7.69e-05	HRAS,STAT5A,AKT1,SHC2,FRS2,MAPK7,GRB2,MAP2K1,RAF1,STAT3,YWHAG,YWHAH,RAP1B
1	T cell activation(P)	100	13	0	<7.14e-05	HRAS,AKT1,PAK3,CSK,HLA-DQA1,NCK2,GRB2,CD247,RAC2,MAP2K1,MAP2K2,RAF1,CALM1
1	EGFR1(C)	149	15	0	<6.67e-05	HRAS,STAT5A,GJA1,AKT1,CSK,EGFR,PRKCG,NCK2,MAPK7,MAP2K1,MAP2K2,CBL,RAF1,STAT3,JAK2
1	Sphingosine 1-phosphate (S1P) pathway(N)	130	14	0	<6.25e-05	HRAS,STAT5A,AKT1,CSK,NCK2,PDGFRB,FGR,GRB2,MAP2K1,MAP2K2,CBL,RAF1,STAT3,JAK2
1	T cell receptor signaling pathway(K)	108	13	0	<5.88e-05	HRAS,CD8A,AKT1,PAK3,NCK2,GRB2,CD247,CD4,PTPN6,MAP2K1,MAP2K2,CBL,RAF1
1	Insulin signaling pathway(K)	137	14	0	<5.56e-05	HRAS,PRKAG2,AKT1,SHC2,SHC4,GRB2,PRKAA1,MAP2K1,MAP2K2,CBL,ACACA,RAF1,ACACB,CALM1
1	PDGFR-beta signaling pathway(N)	53	10	0	<5.26e-05	HRAS,STAT5A,AKT1,CSK,NCK2,PDGFRB,FGR,GRB2,CBL,STAT3
1	IFN-gamma pathway(N)	373	21	0	<5.00e-05	STAT5A,AKT1,DAB2,PAK3,IL13RA2,PRKCG,PRKCE,FGR,GRB2,STAT6,IL2RG,PTPN6,MAP2K1,CBL,RAF1,YWHAG,YWHAH,RAP1B,JAK2,JAK3,CALM1
1	Trk receptor signaling mediated by PI3K and PLC-gamma(N)	60	10	0	<4.76e-05	HRAS,STAT5A,AKT1,MAPK7,GRB2,MAP2K1,RAF1,YWHAG,YWHAH,RAP1B
1	Nectin adhesion pathway(N)	105	12	0	<4.55e-05	HRAS,STAT5A,AKT1,CSK,NCK2,PDGFRB,FGR,GRB2,CBL,STAT3,TJP1,RAP1B
1	Signaling by PDGF(R)	64	10	0	<4.35e-05	HRAS,STAT5A,NCK2,PDGFRB,GRB2,STAT6,MAP2K1,MAP2K2,RAF1,STAT3
1	S1P1 pathway(N)	65	10	0	<4.17e-05	HRAS,STAT5A,AKT1,CSK,NCK2,PDGFRB,FGR,GRB2,CBL,STAT3
1	IL2-mediated signaling events(N)	111	12	0	<4.00e-05	HRAS,STAT5A,AKT1,PRKCE,GRB2,IL2RG,MAP2K1,MAP2K2,RAF1,STAT3,JAK3,CALM1
1	Signaling events mediated by PTP1B(N)	51	9	0	<3.85e-05	STAT5A,AKT1,CSK,EGFR,PDGFRB,FGR,GRB2,STAT3,JAK2
1	Signaling events activated by Hepatocyte Growth Factor Receptor (c-Met)(N)	169	14	0	<3.70e-05	HRAS,AKT1,EGFR,NCK2,GRB2,CD4,MAP2K1,MAP2K2,CBL,RAF1,STAT3,YWHAG,YWHAH,RAP1B
1	Long-term potentiation(K)	70	10	0	<3.57e-05	HRAS,GRIN2D,PRKCG,MAP2K1,MAP2K2,RAF1,ITPR3,ITPR2,RAP1B,CALM1
1	MAPK signaling pathway(K)	267	17	0	<3.33e-05	HRAS,AKT1,EGFR,PRKCG,RASGRF2,PDGFRB,MAPK7,FGFR1,FGFR4,GRB2,RASGRP3,RAC2,MAP2K1,MAP2K2,RAF1,CACNA2D4,RAP1B
1	Proteogylcan syndecan-mediated signaling events(N)	267	17	0	<3.33e-05	HRAS,AKT1,FRS2,EGFR,NCK2,FGFR1,FGFR4,GRB2,CD4,MAP2K1,MAP2K2,CBL,RAF1,STAT3,YWHAG,YWHAH,RAP1B
1	B cell activation(P)	71	10	0	<3.23e-05	HRAS,GRB2,RAC2,PTPN6,MAP2K1,MAP2K2,RAF1,ITPR3,ITPR2,CALM1
1	KitReceptor(C)	53	9	0	<3.13e-05	HRAS,STAT5A,AKT1,CSF2RB,FGR,CBL,RAF1,JAK2,EPOR
1	Signalling by NGF(R)	173	14	0	<3.03e-05	HRAS,AKT1,SHC2,FRS2,PRKCE,MAPK7,GRB2,MAP2K1,MAP2K2,RAF1,ITPR3,STAT3,ITPR2,CALM1
1	Chronic myeloid leukemia(K)	73	10	0	<2.94e-05	HRAS,STAT5A,AKT1,SHC2,SHC4,GRB2,MAP2K1,MAP2K2,CBL,RAF1
1	Syndecan-1-mediated signaling events(N)	177	14	0	<2.86e-05	HRAS,AKT1,EGFR,NCK2,GRB2,CD4,MAP2K1,MAP2K2,CBL,RAF1,STAT3,YWHAG,YWHAH,RAP1B

Supplemental Table 7. DR shRNA screen functional interaction modules

1	Interleukin signaling pathway(P)	77	10	0	<2.78e-05	STAT5A,AKT1,IL13RA2,MAPK7,GRB2,STAT6,IL10RA,RAF1,STAT3,JAK3
1	Chemokine signaling pathway(K)	189	14	0	<2.70e-05	HRAS,AKT1,CSK,SHC2,SHC4,FGR,GRB2,RAC2,MAP2K1,RAF1,STAT3,RAP1B,JAK2,JAK3
1	IL4-mediated signaling events(N)	61	9	0	<2.63e-05	STAT5A,IL13RA2,GRB2,STAT6,IL2RG,PTPN6,CBL,JAK2,JAK3
1	TCR signaling in naïve CD4+ T cells(N)	133	12	0	<2.56e-05	HRAS,AKT1,CSK,PRKCE,GRB2,CD247,CD4,PTPN6,MAP2K1,CBL,RAF1,CALM1
1	Signaling by EGFR(R)	45	8	0	<2.44e-05	HRAS,CSK,EGFR,GRB2,MAP2K1,MAP2K2,CBL,RAF1
1	il-2 receptor beta chain in t cell activation(B)	45	8	0	<2.44e-05	HRAS,STAT5A,GRB2,IL2RG,MAP2K1,MAP2K2,CBL,RAF1
1	sprouty regulation of tyrosine kinase signals(B)	18	6	0	<2.38e-05	HRAS,GRB2,MAP2K1,MAP2K2,CBL,RAF1
1	Endothelins(N)	168	13	0	<2.33e-05	HRAS,AKT1,EGFR,PRKCG,PRKCE,GRB2,MAP2K1,MAP2K2,RAF1,YWHAG,YWHAH,JAK2,CALM1
1	Focal adhesion(K)	200	14	0	<2.27e-05	HRAS,AKT1,PAK3,SHC2,SHC4,EGFR,MYLK2,PRKCG,PDGFRB,GRB2,RAC2,MAP2K1,RAF1,RAP1B
1	p75(NTR)-mediated signaling(N)	174	13	0	<2.22e-05	HRAS,STAT5A,AKT1,SHC2,FRS2,MAPK7,GRB2,MAP2K1,RAF1,STAT3,YWHAG,YWHAH,RAP1B
1	EPO signaling pathway(N)	33	7	0	<2.17e-05	HRAS,STAT5A,GRB2,PTPN6,CBL,JAK2,EPOR
1	Non-small cell lung cancer(K)	54	8	0	<2.13e-05	HRAS,AKT1,EGFR,PRKCG,GRB2,MAP2K1,MAP2K2,RAF1
1	B cell receptor signaling pathway(K)	75	9	0	<2.08e-05	HRAS,AKT1,GRB2,RASGRP3,RAC2,PTPN6,MAP2K1,MAP2K2,RAF1
1	GnRH signaling pathway(K)	101	10	0	<2.04e-05	HRAS,EGFR,MAPK7,GRB2,MAP2K1,MAP2K2,RAF1,ITPR3,ITPR2,CALM1
1	erk1/erk2 mapk signaling pathway(B)	23	6	0	<2.00e-05	HRAS,GRB2,MAP2K1,MAP2K2,RAF1,STAT3
1	Endothelin signaling pathway(P)	59	8	0	<1.92e-05	AKT1,PRKCG,PRKCE,MAP2K1,MAP2K2,RAF1,ITPR3,ITPR2
1	Acute myeloid leukemia(K)	59	8	0	<1.92e-05	HRAS,STAT5A,AKT1,GRB2,MAP2K1,MAP2K2,RAF1,STAT3
1	phosphorylation of mek1 by cdk5/p35 down regulates the map kinase pathway(B)	13	5	0	<1.82e-05	HRAS,GRB2,MAP2K1,MAP2K2,RAF1
1	il 6 signaling pathway(B)	13	5	0	<1.82e-05	HRAS,GRB2,STAT3,JAK2,JAK3
1	multiple antiapoptotic pathways from igf-1r signaling lead to bad phosphorylation(B)	13	5	0	<1.82e-05	HRAS,EGFR,GRB2,RAF1,YWHAH
1	tpo signaling pathway(B)	26	6	0	<1.79e-05	HRAS,STAT5A,GRB2,MAP2K1,RAF1,STAT3
1	BCR signaling pathway(N)	65	8	0	<1.75e-05	HRAS,AKT1,CSK,GRB2,PTPN6,MAP2K1,RAF1,CALM1
1	Prostate cancer(K)	89	9	0	<1.72e-05	HRAS,AKT1,EGFR,PDGFRB,FGFR1,GRB2,MAP2K1,MAP2K2,RAF1
1	Signaling events mediated by VEGFR1 and VEGFR2(N)	66	8	0	<1.69e-05	HRAS,AKT1,SHC2,FRS2,GRB2,PTPN6,CBL,CALM1
1	Class I PI3K signaling events(N)	251	14	0	<1.67e-05	HRAS,AKT1,CSK,PRKCG,PRKCE,FGR,GRB2,CD4,PTPN6,MAP2K1,RAF1,YWHAG,YWHAH,CALM1
1	Regulation of actin cytoskeleton(K)	215	13	0	<1.64e-05	HRAS,WASF1,PAK3,CSK,EGFR,MYLK2,PDGFRB,FGFR1,FGFR4,RAC2,MAP2K1,MAP2K2,RAF1
1	Angiogenesis(P)	92	9	0	<1.61e-05	AKT1,SHC2,FRS2,PRKCG,PDGFRB,FGFR1,GRB2,RAF1,STAT3
1	Renal cell carcinoma(K)	70	8	0	<1.56e-05	HRAS,AKT1,PAK3,GRB2,MAP2K1,MAP2K2,RAF1,RAP1B
1	Ras Pathway(P)	70	8	0	<1.56e-05	AKT1,PAK3,GRB2,RAC2,MAP2K1,MAP2K2,RAF1,STAT3
1	keratinocyte differentiation(B)	49	7	0	<1.54e-05	HRAS,PRKCG,PRKCE,GRB2,MAP2K1,MAP2K2,RAF1
1	Melanoma(K)	71	8	0	<1.52e-05	HRAS,AKT1,EGFR,PDGFRB,FGFR1,MAP2K1,MAP2K2,RAF1
1	Endometrial cancer(K)	52	7	0	<1.49e-05	HRAS,AKT1,EGFR,GRB2,MAP2K1,MAP2K2,RAF1
1	Plasma membrane estrogen receptor signaling(N)	310	15	0	<1.47e-05	HRAS,AKT1,PAK3,EGFR,PRKCG,PRKCE,NCK2,FGR,GRB2,CD4,CBL,RAF1,YWHAG,YWHAH,CALM1
1	angiotensin ii mediated activation of jnk pathway via pyk2 dependent signaling(B)	34	6	0	<1.45e-05	HRAS,CSK,GRB2,MAP2K1,MAP2K2,RAF1
1	VEGF signaling pathway(K)	76	8	0	<1.43e-05	HRAS,AKT1,SHC2,PRKCG,RAC2,MAP2K1,MAP2K2,RAF1
1	Fc epsilon RI signaling pathway(K)	79	8	0	<1.41e-05	HRAS,AKT1,PRKCE,GRB2,RAC2,MAP2K1,MAP2K2,RAF1
1	TRAIL signaling pathway(N)	323	15	0	<1.39e-05	HRAS,AKT1,CSK,PRKCG,PRKCE,FGR,GRB2,CD4,PTPN6,MAP2K1,MAP2K2,RAF1,YWHAG,YWHAH,CALM1
1	regulation of splicing through sam68(B)	10	4	0	<1.35e-05	HRAS,MAP2K1,MAP2K2,RAF1
1	il22 soluble receptor signaling pathway(B)	10	4	0	<1.35e-05	STAT5A,IL22,IL10RA,STAT3
1	egf signaling pathway(B)	22	5	0	<1.32e-05	HRAS,EGFR,GRB2,MAP2K1,STAT3
1	igf-1 signaling pathway(B)	22	5	0	<1.32e-05	HRAS,EGFR,GRB2,MAP2K1,RAF1
1	IL12-mediated signaling events(N)	112	9	0	<1.30e-05	CD8A,STAT5A,HLA-A,CD247,STAT6,IL2RG,CD4,STAT3,JAK2
1	Fc-epsilon receptor I signaling in mast cells(N)	61	7	0	<1.28e-05	HRAS,AKT1,GRB2,MAP2K1,MAP2K2,CBL,RAF1
1	Vascular smooth muscle contraction(K)	116	9	0	<1.27e-05	MYLK2,PRKCG,PRKCE,MAP2K1,MAP2K2,RAF1,ITPR3,ITPR2,CALM1
1	il 2 signaling pathway(B)	11	4	0	1.25E-05	HRAS,STAT5A,GRB2,IL2RG
1	inhibition of cellular proliferation by gleevec(B)	24	5	0	1.23E-05	HRAS,STAT5A,GRB2,MAP2K1,RAF1

Supplemental Table 7. DR shRNA screen functional interaction modules

1	IGF1 pathway(N)	150	10	0	1.22E-05	HRAS,AKT1,EGFR,NCK2,GRB2,CD4,CBL,RAF1,YWHAG,YWHAH
1	Glypican 1 network(N)	436	17	0	1.20E-05	HRAS,AKT1,DAB2,PAK3,CSK,PRKCG,PRKCE,FGFR1,FGR,GRB2,CD4,PTPN6,MAP2K1,RAF1,YWHAG,YWHAH,CALM1
1	links between pyk2 and map kinases(B)	25	5	0	1.18E-05	HRAS,GRB2,MAP2K1,MAP2K2,RAF1
1	role of erk5 in neuronal survival pathway(B)	25	5	0	1.18E-05	HRAS,GRB2,MAP2K1,MAP2K2,RAF1
1	Adipocytokine signaling pathway(K)	67	7	0	1.16E-05	PRKAG2,CAMKK1,AKT1,PRKAA1,ACACB,STAT3,JAK2
1	fmlp induced chemokine gene expression in hmc-1 cells(B)	26	5	0	1.15E-05	HRAS,CAMKK1,MAP2K1,MAP2K2,RAF1
1	Fc gamma R-mediated phagocytosis(K)	95	8	0	2.27E-05	WASF1,AKT1,PRKCG,PRKCE,RAC2,PPAP2A,MAP2K1,RAF1
1	Signaling by FGFR(R)	27	5	0	2.25E-05	FRS2,FGFR1,FGFR4,GRB2,CBL
1	il 3 signaling pathway(B)	13	4	0	3.30E-05	HRAS,STAT5A,CSF2RB,GRB2
1	epo signaling pathway(B)	13	4	0	3.30E-05	HRAS,STAT5A,GRB2,EPOR
1	Inflammation mediated by chemokine and cytokine signaling pathway(P)	127	9	0	3.26E-05	AKT1,MYLK2,PRKCE,IFNAR1,GRB2,RAC2,RAF1,STAT3,JAK2
1	Long-term depression(K)	70	7	0	3.23E-05	HRAS,PRKCG,MAP2K1,MAP2K2,RAF1,ITPR3,ITPR2
1	a6b1 and a6b4 Integrin signaling(N)	47	6	0	3.19E-05	HRAS,AKT1,EGFR,GRB2,YWHAG,YWHAH
1	pdgf signaling pathway(B)	28	5	0	4.21E-05	HRAS,GRB2,MAP2K1,RAF1,STAT3
1	calcium signaling by hbx of hepatitis b virus(B)	14	4	0	5.21E-05	HRAS,GRB2,MAP2K1,RAF1
1	fc epsilon receptor i signaling in mast cells(B)	29	5	0	5.15E-05	HRAS,GRB2,MAP2K1,MAP2K2,RAF1
1	Pathways in cancer(K)	327	14	0	5.10E-05	HRAS,STAT5A,AKT1,EGFR,PRKCG,PDGFRB,FGFR1,GRB2,RAC2,MAP2K1,MAP2K2,CBL,RAF1,STAT3
1	Glypican pathway(N)	472	17	0	5.05E-05	HRAS,AKT1,DAB2,PAK3,CSK,PRKCG,PRKCE,FGFR1,FGR,GRB2,CD4,PTPN6,MAP2K1,RAF1,YWHAG,YWHAH,CALM1
1	cadmium induces dna synthesis and proliferation in macrophages(B)	15	4	0	7.92E-05	HRAS,MAP2K1,MAP2K2,RAF1
1	melanocyte development and pigmentation pathway(B)	15	4	0	7.92E-05	HRAS,MAP2K1,MAP2K2,RAF1
1	t cell receptor signaling pathway(B)	51	6	0	7.84E-05	HRAS,GRB2,CD247,CD4,MAP2K1,RAF1
1	Arf6 trafficking events(N)	139	9	0	8.57E-05	HRAS,AKT1,EGFR,NCK2,GRB2,CD4,CBL,YWHAG,YWHAH
1	Arf6 signaling events(N)	139	9	0	8.57E-05	HRAS,AKT1,EGFR,NCK2,GRB2,CD4,CBL,YWHAG,YWHAH
1	Insulin Pathway(N)	139	9	0	8.57E-05	HRAS,AKT1,EGFR,NCK2,GRB2,CD4,CBL,YWHAG,YWHAH
1	EphrinB-EPHB pathway(N)	53	6	0	8.49E-05	HRAS,NCK2,FGR,GRB2,MAP2K1,RAP1B
1	Trk receptor signaling mediated by the MAPK pathway(N)	32	5	0	8.18E-05	HRAS,MAPK7,MAP2K1,RAF1,RAP1B
1	phospholipids as signalling intermediaries(B)	32	5	0	8.18E-05	HRAS,GRB2,MAP2K1,MAP2K2,RAF1
1	roles of ß arrestin dependent recruitment of src kinases in gpcr signaling(B)	32	5	0	8.18E-05	HRAS,FGR,MAP2K1,MAP2K2,RAF1
1	role of egf receptor transactivation by gpcrs in cardiac hypertrophy(B)	32	5	0	8.18E-05	HRAS,GRB2,MAP2K1,MAP2K2,RAF1
1	role of mal in rho-mediated activation of srf(B)	16	4	0	8.11E-05	HRAS,MAP2K1,MAP2K2,RAF1
1	trefoil factors initiate mucosal healing(B)	33	5	0	1.07E-04	HRAS,GRB2,MAP2K1,MAP2K2,RAF1
1	nerve growth factor pathway (ngf)(B)	18	4	0.0001	1.86E-04	HRAS,GRB2,MAP2K1,RAF1
1	Hematopoietic cell lineage(K)	89	7	0.0001	2.63E-04	CD8A,MS4A1,CD37,CD34,CD4,CD1C,EPOR
1	Signaling by Insulin receptor(R)	39	5	0.0001	3.04E-04	HRAS,GRB2,MAP2K1,MAP2K2,RAF1
1	Bladder cancer(K)	42	5	0.0001	4.19E-04	HRAS,EGFR,MAP2K1,MAP2K2,RAF1
1	FGF signaling pathway(N)	42	5	0.0001	4.19E-04	FRS2,FGFR1,FGFR4,GRB2,CBL
1	influence of ras and rho proteins on g1 to s transition(B)	23	4	0.0002	4.66E-04	HRAS,MAP2K1,MAP2K2,RAF1
1	Syndecan-2-mediated signaling events(N)	70	6	0.0002	5.08E-04	HRAS,FRS2,FGFR1,FGFR4,GRB2,CBL
1	Pancreatic cancer(K)	70	6	0.0002	5.08E-04	AKT1,EGFR,RAC2,MAP2K1,RAF1,STAT3
1	Nongenotropic Androgen signaling(N)	24	4	0.0002	5.54E-04	HRAS,MAP2K1,MAP2K2,RAF1
1	Dorso-ventral axis formation(K)	25	4	0.0002	6.72E-04	PIWIL3,EGFR,GRB2,MAP2K1
1	Adherens junction(K)	74	6	0.0002	7.24E-04	WASF1,EGFR,FGFR1,RAC2,PTPN6,TJP1
1	VEGF signaling pathway(P)	26	4	0.0003	7.92E-04	AKT1,SHC2,PRKCG,RAF1
1	mcalpain and friends in cell motility(B)	26	4	0.0003	7.92E-04	HRAS,GRB2,MAP2K1,MAP2K2
1	Cytokine-cytokine receptor interaction(K)	266	11	0.0003	8.02E-04	IL15RA,CSF2RB,EGFR,IL22,IFNAR1,PDGFRB,IFNA2,IL10RA,IL2RG,IL9,EPOR
1	VEGFR1 specific signals(N)	27	4	0.0003	8.58E-04	AKT1,SHC2,CBL,CALM1
1	TNF receptor signaling pathway(N)	272	11	0.0003	9.84E-04	AKT1,PAK3,PRKCG,PRKCE,FGR,MAP2K1,MAP2K2,RAF1,YWHAG,YWHAH,CALM1

Supplemental Table 7. DR shRNA screen functional interaction modules

2	Heterotrimeric G-protein signaling pathway-Gi alpha and Gs alpha mediated pathway(P)	168	23	0	<2.50e-04	ADCY2,ADORA1,PRKACG,PRKAR2B,HTR1A,GRM5,SSTR5,ADRB1,ARRB2,ARRB1,CHRM2,GPR50,GNAS,GNAI2,GNAI1,ADORA2A,DRD2,DRD5,ADRBK1,ADRB3,ADRA2B,GRK5,OPRD1
2	Chemokine signaling pathway(K)	189	21	0	<2.50e-04	ADCY2,CXCL12,PRKACG,PLCB1,CCR7,CCR6,ARRB2,CCR4,ARRB1,GNB1,CCR2,CCL1,GNAI2,GNAI1,ADRBK1,CCL5,CCL26,CCL20,IL8,CCL18,GRK5
2	Class A/1 (Rhodopsin-like receptors)(R)	183	23	0	<2.50e-04	CXCL12,EDNRA,AGTR2,HTR1A,C5AR1,CCKBR,SSTR5,CCR7,ADRB1,CCR6,CCR4,CHRM2,CCR2,DRD2,DRD5,CCL5,ADRB3,CCL20,ADRA2A,ADRA2B,IL8,IL8RB,OPRD1
2	Receptor-ligand complexes bind G proteins(R)	157	28	0	<2.50e-04	GNA14,CXCL12,AGTR2,HTR1A,C5AR1,SSTR5,CCR7,ADRB1,CCR6,CCR4,GNB1,CHRM2,CCR2,GNAS,GNAI2,GNAI1,DRD2,DRD5,CCL5,ADRB3,CCL20,ADRA2A,ADRA2B,GNAT1,IL8,GNAT2,IL8RB,OPRD1
2	Neuroactive ligand-receptor interaction(K)	272	22	0	<2.00e-04	ADORA1,EDNRA,AGTR2,HTR1A,C5AR1,CCKBR,GRM5,SSTR5,ADRB1,CHRM2,GPR50,C3AR1,CYSLTR1,ADORA2A,DRD2,DRD5,ADRB3,ADRA2A,ADRA2B,GABRA2,GRIA1,OPRD1
2	Opioid Signalling(R)	79	12	0	<1.67e-04	GNA14,ADCY2,PRKACG,PRKAR2B,PDE4B,PLCB1,GNB1,GNAI2,GNAI1,ADRBK1,GNAT1,GNAT2
2	Calcium signaling pathway(K)	178	14	0	<1.43e-04	GNA14,ADCY2,PRKACG,EDNRA,PLCB1,CCKBR,GRM5,ADRB1,CHRM2,GNAS,CYSLTR1,ADORA2A,DRD5,ADRB3
2	Inflammation mediated by chemokine and cytokine signaling pathway(P)	127	12	0	<1.25e-04	PLCB1,C5AR1,CCR7,CCR6,ARRB2,CCR4,GNAI2,ADRBK1,CCL26,CCL20,CCL18,IL8RB
2	Melanogenesis(K)	102	11	0	<1.11e-04	ADCY2,PRKACG,PLCB1,GNAS,WNT5A,GNAI2,GNAI1,DCT,DVL3,WNT7B,FZD10
2	ß-arrestins in gpcr desensitization(B)	26	7	0	<1.00e-04	CXCL12,AGTR2,ARRB1,GNB1,GNAS,ADRBK1,DNM1
2	role of ß-arrestins in the activation and targeting of map kinases(B)	28	7	0	<9.09e-05	CXCL12,AGTR2,ARRB1,GNB1,GNAS,ADRBK1,DNM1
2	roles of ß arrestin dependent recruitment of src kinases in gpcr signaling(B)	32	7	0	<8.33e-05	CXCL12,AGTR2,ARRB1,GNB1,GNAS,ADRBK1,DNM1
2	Thromboxane A2 receptor signaling(N)	54	8	0	<7.69e-05	GNA14,GNA12,SLC9A3R1,ARRB2,GNB1,GNAI2,ADRBK1,DNM1
2	regulation of ck1/cdk5 by type 1 glutamate receptors(B)	22	6	0	<7.14e-05	PRKAR2B,PLCB1,GNB1,GNAS,DRD2,ADRBK1
2	Gap junction(K)	90	9	0	<6.67e-05	ADCY2,PRKACG,PLCB1,GRM5,ADRB1,GNAS,GNAI2,GNAI1,DRD2
2	Heterotrimeric G-protein signaling pathway-Gq alpha and Go alpha mediated pathway(P)	126	10	0	<6.25e-05	ADORA1,PLCB1,GRM5,SSTR5,GNB1,CHRM2,ADORA2A,DRD2,DRD5,OPRD1
2	LPA receptor mediated events(N)	97	9	0	<5.88e-05	GNA14,ADCY2,GNA12,ARRB2,GNB1,GNAS,GNAI2,GNAI1,IL8
2	Long-term depression(K)	70	8	0	<5.56e-05	GNA12,PLCB1,GRM5,GNAS,PLA2G2D,GNAI2,GNAI1,GRIA1
2	cystic fibrosis transmembrane conductance regulator (cftr) and beta 2 adrenergic receptor (b2ar) pathway(B)	19	5	0	5.26E-05	CXCL12,PRKAR2B,SLC9A3R1,GNB1,GNAS
2	Signaling events mediated by the Hedgehog family(N)	63	7	0	1.00E-04	CSNK1G1,CSNK1G2,ARRB2,GNB1,GNAI2,GNAI1,ADRBK1
2	signaling pathway from g-protein families(B)	22	5	0	9.52E-05	PRKAR2B,PLCB1,GNB1,GNAS,GNAI1
2	Endothelins(N)	168	10	0	9.09E-05	GNA14,ADCY2,GNA12,EDNRA,PLCB1,GNAS,GNAI2,CYSLTR1,GNAI1,IL8
2	cxcr4 signaling pathway(B)	11	4	0	8.70E-05	CXCL12,PLCB1,GNB1,GNAI1
2	Hedgehog signaling events mediated by Gli proteins(N)	46	6	0	8.33E-05	CSNK1G1,CSNK1G2,ARRB2,GNB1,GNAI2,GNAI1
2	activation of camp-dependent protein kinase pka(B)	26	5	0	8.00E-05	CXCL12,PRKAR2B,AGTR2,GNB1,GNAS
2	Gastric acid secretion(K)	74	7	0	7.69E-05	ADCY2,PRKACG,PLCB1,CCKBR,GNAS,GNAI2,GNAI1
2	attenuation of gpcr signaling(B)	12	4	0	7.41E-05	PRKAR2B,ARRB1,GNB1,GNAS
2	Phototransduction(K)	29	5	0	7.14E-05	ARRB2,ARRB1,GNB1,GNAT1,GNAT2
2	Vascular smooth muscle contraction(K)	116	8	0	1.38E-04	ADCY2,GNA12,PRKACG,EDNRA,PLCB1,GNAS,PLA2G2D,ADORA2A
2	Glucagon signaling in metabolic regulation(R)	34	5	0	2.33E-04	ADCY2,PRKACG,PRKAR2B,GNB1,GNAS
2	Plasma membrane estrogen receptor signaling(N)	310	12	0	2.26E-04	GNA14,PLCB1,PLD1,ESR2,ARRB2,ARRB1,GNB1,CHRM2,GNAS,GNAI2,GNAI1,IL8
2	chrebp regulation by carbohydrates and camp(B)	35	5	0	2.19E-04	CXCL12,PRKAR2B,AGTR2,GNB1,GNAS
2	Cytokine-cytokine receptor interaction(K)	266	11	0	2.73E-04	CXCL12,CCR7,CCR6,CCR4,CCR2,CCL1,CCL5,CCL26,CCL20,IL8,CCL18
2	Proteogylcan syndecan-mediated signaling events(N)	267	11	0	2.65E-04	GNA14,CXCL12,PLD1,ARRB2,ARRB1,GNB1,CHRM2,GNAS,WNT5A,CCL5,IL8
2	activation of csk by camp-dependent protein kinase inhibits signaling through the t cell receptor(B)	42	5	0	6.29E-04	CXCL12,PRKAR2B,AGTR2,GNB1,GNAS
2	Chagas disease(K)	104	7	0	6.11E-04	GNA14,PLCB1,GNAS,GNAI2,GNAI1,CCL5,IL8

Supplemental Table 7. DR shRNA screen functional interaction modules

2	how progesterone initiates the oocyte maturation(B)	22	4	0	7.84E-04	PRKAR2B,GNB1,GNAS,GNAI1
2	Syndecan-4-mediated signaling events(N)	47	5	0.0001	1.00E-03	CXCL12,GNB1,GNAS,WNT5A,CCL5
2	S1P3 pathway(N)	24	4	0.0001	9.74E-04	GNA14,GNA12,GNAI2,GNAI1
3	hiv-1 nef: negative effector of fas and tnf(B)	32	11	0	<1.00e-03	CASP7,CASP8,FAS,RELA,FADD,BID,TRAF2,TNFRSF1A,CFLAR,TRADD,MAPK14
3	HIV-1 Nef: Negative effector of Fas and TNF-alpha(N)	33	11	0	<5.00e-04	NFKB1,CASP7,CASP8,FAS,RELA,FADD,BID,TRAF2,TNFRSF1A,CFLAR,TRADD
3	Apoptosis(K)	88	14	0	<3.33e-04	NFKB1,CASP7,CASP8,FAS,IRAK1,RELA,FADD,TNFRSF10D,BID,TRAF2,IL1R1,TNFRSF1A,CFLAR,TRADD
3	nf-kb signaling pathway(B)	21	9	0	<2.50e-04	NFKB1,IRAK1,RELA,FADD,IL1R1,TNFRSF1A,TRAF6,TRADD,MAPK14
3	TNF alpha/NF-kB(C)	136	14	0	<2.00e-04	NFKB1,CASP7,CASP8,RELA,FADD,TANK,RIPK2,TRAF2,TNFRSF8,TNFRSF1A,TRAF6,CFLAR,TRADD,FAF1
3	TNF receptor signaling pathway(N)	272	17	0	<1.67e-04	NFKB1,APP,CASP7,CASP8,ITCH,RELA,FADD,TXN,RIPK2,MAP3K12,BID,TRAF2,MAP3K7IP2,TNFRSF1A,TRAF6,TRADD,MAPK14
3	TRAIL signaling pathway(N)	323	17	0	<1.43e-04	NFKB1,APP,CASP7,CASP8,ITCH,RELA,FADD,TNFRSF10D,EIF4A1,RIPK2,BID,TRAF2,TNFRSF1A,TRAF6,CFLAR,TRADD,MAPK14
3	fas signaling pathway (cd95)(B)	20	7	0	<1.25e-04	CASP7,CASP8,FAS,FADD,CFLAR,MAPK14,FAF1
3	FAS signaling pathway (CD95)(N)	35	8	0	<1.11e-04	NFKB1,CASP7,CASP8,FAS,FADD,BID,CFLAR,FAF1
3	induction of apoptosis through dr3 and dr4/5 death receptors(B)	21	7	0	<1.00e-04	CASP7,CASP8,FADD,BID,TRAF2,TRADD,MAPK14
3	Chagas disease(K)	104	11	0	<9.09e-05	TLR2,NFKB1,CASP8,FAS,IRAK1,RELA,FADD,TNFRSF1A,TRAF6,CFLAR,MAPK14
3	Ceramide signaling pathway(N)	47	8	0	<8.33e-05	NFKB1,CASP8,RELA,FADD,BID,TRAF2,TNFRSF1A,TRADD
3	IL1-mediated signaling events(N)	204	13	0	<7.69e-05	NFKB1,ITCH,IRAK1,RELA,RIPK2,MAP3K12,IL1R1,UBE2V1,MAP3K7IP2,TNFRSF1A,TRAF6,IL1RN,MAPK14
3	RIG-I-like receptor signaling pathway(K)	71	9	0	<7.14e-05	NFKB1,CASP8,RELA,FADD,TANK,TRAF2,TRAF6,TRADD,MAPK14
3	tnfr1 signaling pathway(B)	17	6	0	<6.67e-05	CASP8,FADD,TRAF2,TNFRSF1A,TRADD,MAPK14
3	ceramide signaling pathway(B)	32	7	0	<6.25e-05	CASP8,RELA,FADD,TRAF2,TNFRSF1A,TRADD,MAPK14
3	Apoptosis signaling pathway(P)	75	9	0	<5.88e-05	CASP7,CASP8,FAS,LTB,RELA,FADD,TRAF2,TNFRSF1A,CFLAR
3	FAS signaling pathway(P)	34	7	0	<5.56e-05	CASP7,CASP8,FAS,FADD,CFLAR,PARP4,FAF1
3	Cytokine-cytokine receptor interaction(K)	266	14	0	<5.26e-05	TNFSF13,TNFRSF11B,FAS,LTB,TNFSF13B,TNFRSF10D,NGFR,IL1R1,TNFRSF8,TNFRSF1A,TNFRSF18,IL18R1,TNFRSF13B,TNFSF9
3	signal transduction through il1r(B)	35	7	0	<5.00e-05	IRAK1,RELA,IL1R1,MAP3K7IP2,TRAF6,IL1RN,MAPK14
3	Apoptosis(R)	128	10	0	<4.76e-05	CASP7,CASP8,FAS,RPS27A,FADD,BID,TRAF2,TNFRSF1A,CFLAR,TRADD
3	Toll-like receptor signaling pathway(K)	102	9	0	<4.55e-05	TLR2,NFKB1,TLR8,CASP8,IRAK1,RELA,FADD,TRAF6,MAPK14
3	Caspase cascade in apoptosis(N)	52	7	0	<4.35e-05	APP,CASP7,CASP8,BID,TRAF2,TNFRSF1A,TRADD
3	toll-like receptor pathway(B)	34	6	0	<4.17e-05	TLR2,IRAK1,RELA,MAP3K7IP2,TRAF6,MAPK14
3	Canonical NF-kappaB pathway(N)	35	6	0	<4.00e-05	NFKB1,RELA,RIPK2,TNFRSF1A,TRAF6,MAPK14
3	TCR signaling(R)	59	7	0	<3.85e-05	NFKB1,RPS27A,RELA,RIPK2,UBE2V1,MAP3K7IP2,TRAF6
3	Sphingosine 1-phosphate (S1P) pathway(N)	130	9	0	<3.70e-05	NFKB1,CASP8,RELA,FADD,BID,TRAF2,TNFRSF1A,TRADD,MAPK14
3	tnf/stress related signaling(B)	21	5	0	<3.57e-05	TANK,TRAF2,TNFRSF1A,TRADD,MAPK14
3	IL23-mediated signaling events(N)	65	7	0	<3.45e-05	NFKB1,RELA,RIPK2,TNFRSF1A,TRAF6,IL18R1,MAPK14
3	Innate Immunity Signaling(R)	104	8	0	<3.33e-05	TLR2,NFKB1,TLR8,IRAK1,RELA,MAP3K7IP2,TRAF6,TLR10
3	IL12-mediated signaling events(N)	112	8	0	<3.23e-05	NFKB1,RELA,RIPK2,IL1R1,TNFRSF1A,TRAF6,IL18R1,MAPK14
3	the 41bb-dependent immune response(B)	14	4	0	<3.13e-05	RELA,TRAF2,TNFSF9,MAPK14
3	p75(NTR)-mediated signaling(N)	174	9	0	<3.03e-05	NFKB1,APP,IRAK1,RELA,RIPK2,NGFR,TNFRSF1A,TRAF6,MAPK14
3	Class I PI3K signaling events mediated by Akt(N)	94	7	0	<2.94e-05	NFKB1,RELA,EIF4A1,RIPK2,TNFRSF1A,TRAF6,MAPK14
3	NOD-like receptor signaling pathway(K)	62	6	0	<2.78e-05	NFKB1,CASP8,RELA,RIPK2,TRAF6,MAPK14
3	Aurora A signaling(N)	62	6	0	<2.78e-05	NFKB1,RELA,RIPK2,TNFRSF1A,TRAF6,MAPK14
3	IL2 signaling events mediated by PI3K(N)	65	6	0	<2.70e-05	NFKB1,RELA,RIPK2,TNFRSF1A,TRAF6,MAPK14
3	internal ribosome entry pathway(B)	18	4	0	5.26E-05	CASP7,CASP8,EIF4G3,EIF4A1
3	Signaling events regulated by Ret tyrosine kinase(N)	69	6	0	7.69E-05	NFKB1,RELA,RIPK2,TNFRSF1A,TRAF6,MAPK14
3	Glypican 1 network(N)	436	13	0	1.50E-04	NFKB1,APP,ITCH,RELA,EIF4A1,TXN,RIPK2,MAP3K12,TRAF2,MAP3K7IP2,TNFRSF1A,TRAF6,MAPK14
3	Leishmaniasis(K)	73	6	0	1.46E-04	TLR2,NFKB1,IRAK1,RELA,TRAF6,MAPK14
3	keratinocyte differentiation(B)	49	5	0	1.90E-04	FAS,RELA,TRAF2,TNFRSF1A,MAPK14
3	Alzheimer's disease(K)	168	8	0	1.86E-04	APP,CASP7,CASP8,FAS,FADD,BID,TNFRSF1A,GAPDH

Supplemental Table 7. DR shRNA screen functional interaction modules

3	Glypican pathway(N)	472	13	0	1.82E-04	NFKB1,APP,ITCH,RELA,EIF4A1,TXN,RIPK2,MAP3K12,TRAF2, MAP3K7IP2,TNFRSF1A,TRAF6,MAPK14
3	Neurotrophin signaling pathway(K)	126	7	0	1.78E-04	NFKB1,IRAK1,RELA,RIPK2,NGFR,TRAF6,MAPK14
3	Signalling by NGF(R)	173	8	0	1.74E-04	NFKB1,RPS27A,IRAK1,RELA,RIPK2,NGFR,TRAF6,MAPK14
3	TCR signaling in naïve CD8+ T cells(N)	127	7	0	1.70E-04	NFKB1,RELA,RIPK2,TNFRSF1A,TNFRSF18,TRAF6,MAPK14
3	Signaling by Aurora kinases(N)	94	6	0.0001	4.79E-04	NFKB1,RELA,RIPK2,TNFRSF1A,TRAF6,MAPK14
3	LPA receptor mediated events(N)	97	6	0.0001	5.71E-04	NFKB1,RELA,RIPK2,TNFRSF1A,TRAF6,MAPK14
3	Adipocytokine signaling pathway(K)	67	5	0.0002	9.80E-04	NFKB1,RELA,TRAF2,TNFRSF1A,TRADD
4	Complement and coagulation cascades(K)	69	9	0	<1.00e-03	F12,F10,F9,PROC,F2,C3,C1R,C4BPB,PLG
4	intrinsic prothrombin activation pathway(B)	23	6	0	<5.00e-04	F12,F10,F9,PROC,F2,COL4A6
4	ECM-receptor interaction(K)	84	8	0	<3.33e-04	SDC4,SDC1,LAMC1,IBSP,ITGB4,FN1,COL5A3,COL4A6
4	Formation of Fibrin Clot (Clotting Cascade)(R)	32	5	0	1.00E-03	F12,F10,F9,PROC,F2
4	Metabolism of lipids and lipoproteins(R)	106	7	0	1.00E-03	APOA1,APOE,LCAT,SDC1,DCI,ALB,SCARB1
5	SNARE interactions in vesicular transport(K)	36	3	0	<1.00e-03	SNAP29,STX1A,STX17
6	Collecting duct acid secretion(K)	27	3	0	1.00E-03	ATP6V0A1,ATP6V1D,ATP6V1F
6	Vibrio cholerae infection(K)	55	3	0	5.00E-04	ATP6V0A1,ATP6V1D,ATP6V1F
6	Epithelial cell signaling in Helicobacter pylori infection(K)	68	3	0	3.33E-04	ATP6V0A1,ATP6V1D,ATP6V1F

Komarov et al. Functional genetics-directed identification of novel pharmacological inhibitors of FAS- and TNF-dependent apoptosis that protect mice from acute liver failure

Supplemental Table 8:

Key node analysis (upstream & downstream)

Supplemental Table 8. Key node analysis (upstream & downstream)

Upstream Regulators			
**Upstream Regulator**	**Molecule Type**	**p-value**	**Target molecules in Dataset**
lysophosphatidic acid	chemical - other	4.71E-11	CCL26,CXCL8,EGFR,EGR1,FABP4,HIF1A,IL13RA2,JUN,NFKB1,PDGFRB,PPARG,PRKCG,SCARB1,TP53
lysophosphatidylcholine	chemical - other	4.33E-06	CCL5,CXCL8,EGR1,GJA1,JUN,PPARG,SCARB1
platelet activating factor	chemical - endogenous mammalian	1.82E-04	CDKN1A,EGR1,FN1,GAS6,SELL,SELP
sphingosylphospho-choline	chemical - endogenous non-mammalian	8.31E-03	JUN,TGM1
tributyrin	chemical drug	6.11E-03	AZGP1,CCL20,CDKN1A,CXCL8,MMP13
octanoic acid	chemical - endogenous mammalian	1.78E-03	FABP4,PPARD,PPARG
8,9-epoxyeicosatrienoic acid	chemical - endogenous mammalian	1.06E-02	EGFR,FABP4
11,12-epoxyeicosatrienoic acid	chemical - endogenous mammalian	3.88E-04	CYP2C9,EGFR,FABP4,PPARG
14,15-epoxyeicosatrienoic acid	chemical - endogenous mammalian	8.40E-04	CYP2C9,EGFR,FABP4
5,6-epoxyeicosatrienoic acid	chemical - endogenous mammalian	3.54E-02	FABP4
15-deoxy-delta-12,14 -PGJ 2	chemical - endogenous non-mammalian	1.19E-12	BCL2,CCL5,CDKN1A,CFLAR,CTSB,CXCL8,EGR1,ESR1,FABP4,FN1,GSTP1,JUN,MAPK14,MMP13,NFE2L2,NFKB1,PARP1,PPARG,SCARB1,SP1,TGIF1,TP53
prostaglandin J2	chemical - endogenous non-mammalian	1.76E-02	CFLAR,CXCL8,PSMA5
prostaglandin D2	chemical - endogenous mammalian	1.21E-02	CCL5,CXCL8,TNFRSF11B
epoprostenol	chemical - endogenous mammalian	2.87E-02	EGR1,HIF1A
dinoprost	chemical - endogenous mammalian	2.09E-03	EGFR,FABP4,GSTM1,GSTP1,JUN,YWHAG
prostaglandin A1	chemical - endogenous non-mammalian	1.84E-06	BCL2,CD14,CDKN1A,CXCL8,PCNA,TP53
U46619	chemical reagent	1.06E-02	CXCL8,SELP
prostaglandin A2	chemical - endogenous non-mammalian	8.73E-05	CDKN1A,NR4A3,PCNA,TP53
20-hydroxyeicosatetraenoic acid	chemical - endogenous mammalian	4.56E-03	APOA1,CYP2C9
leukotriene D4	chemical - endogenous mammalian	4.35E-04	CXCL8,CYSLTR1,EGR1,NR4A3,PDGFRB,RELA
leukotriene B4	chemical - endogenous mammalian	2.87E-02	CXCL8,JUN
leukotriene C4	chemical - endogenous mammalian	1.42E-03	CXCL8,EGR1,SELP
15(S)-HETE	chemical - endogenous mammalian	4.44E-05	FABP4,JUN,MAPK3,PPARG
lipoxin A4	chemical - endogenous mammalian	2.43E-03	CDKN1A,CXCL8,ESR1,FN1
ATP-gamma-S	chemical reagent	1.25E-03	C3,CCR7,CD8A,CXCL8,EPOR,IL15RA
5'-adenylyl (beta,gamma-methylene)diphosphonate	chemical reagent	1.79E-02	SELP
cyclic AMP	chemical - endogenous mammalian	8.15E-13	ADRA2A,APP,BCL2,CDKN1A,CNTF,DCT,EGR1,FN1,GATA3,GJA1,GTF3A,HIF1A,IL1RN,ITPR3,JUN,OPRD1,PDE4B,PRKAR2B,RARA,RXRA,SCARB1,SOX9,SP1,STAT3,TP53,YWHAG
cyclic GMP	chemical - endogenous mammalian	2.87E-02	BCL2,CDKN1A
Npm	group	3.54E-02	CDKN1A
5-N-ethylcarboxamido adenosine	chemical reagent	4.32E-04	ADORA2A,ALB,CSF2RB,CXCL8,EGR1,EIF2B2,HLA-DQA1,NT5E
N(6)-(3-iodobenzyl)-5'-N-methylcarboxamidoadenosine	chemical drug	1.99E-04	AKT1,CTNNB1,ESR1,TP53
adenosine	chemical - endogenous mammalian	3.68E-06	BID,CCL5,CXCL8,GNAS,HIF1A,IL1R1,RELA,TP53
doxifluridine	chemical drug	3.63E-02	CDKN1A,FAS
zidovudine	chemical drug	2.45E-06	AKT1,BID,CASP8,CDKN1A,CXCL8,FOXP1
bromodeoxyuridine	chemical drug	1.58E-02	CXCL8,GSTP1,IL9,NODAL
6-mercaptopurine	chemical drug	1.09E-04	CCR7,NR4A3,PCNA
hypoxanthine	chemical - endogenous mammalian	3.54E-02	CXCL8
O6-benzylguanine	chemical drug	3.37E-06	CDKN1A,CFLAR,FAS,H2AFX
uric acid	chemical - endogenous mammalian	1.41E-04	AGT,BCL2,CCL5,CXCL8,H2AFX,PARP1,PTTG1,TP53
roscovitine	chemical drug	5.20E-06	APP,CDKN1A,FABP4,FN1,OPRD1,PPARG,TP53

Supplemental Table 8. Key node analysis (upstream & downstream)

3-methyladenine	chemical toxicant	1.33E-02	BCL2,CXCL8,TP53
tenofovir	chemical drug	3.54E-02	CXCL8
ganciclovir	chemical drug	3.07E-03	CDKN1A,TP53
adenine	chemical - endogenous mammalian	9.44E-04	CXCL8,FABP1
isobutylmethylxanthine	chemical toxicant	5.28E-12	AGT,AKT1,CD14,CDKN1A,DHFR,EGR1,FABP4,GAPDH,IBSP,IL2RG,JUN,MAPK3,MMP13,NOTCH1,PPARD,PPARG,RBL2,RXRA,SCARB1
pirinixic acid	chemical toxicant	7.33E-13	ACACA,ACACB,ACADM,AHSG,AP3M1,APCS,APOA1,APOE,C3,CCL5,CDKN1A,CTSB,CXCL12,CXCL8,CYP2C9,CYP7A1,ECI1,EGFR,FABP1,FABP4,FAS,FEN1,GAPDH,HIF1A,HLA-DQA1,MMP13,NR1H2,PCNA,PPARA,PPARG,PSMA5,RXRA,TP53,TXN
5-fluorouracil	chemical drug	1.14E-07	ATP5B,ATP5I,BCL2,CANX,CDKN1A,CFLAR,EGR1,EIF4A1,FAS,GAPDH,HSP90AB1,HSPA8,IDH2,LDHA,RAF1,RBMX,RPS27A,TP53
PP1	chemical - kinase inhibitor	1.58E-06	CXCL8,ESR1,GJA1,GRIA1,IL1RN,JUN,TJP1
PP2/AG1879 tyrosine kinase inhibitor	chemical - kinase inhibitor	2.13E-11	BCL2,CBL,CDKN1A,CXCL8,EGFR,FGR,FN1,GRIA1,HIF1A,MMP13,PAX8,RELA,TJP1,TP53
R 59022	chemical - kinase inhibitor	1.06E-02	CXCL8,PPARG

Supplemental Table 8. Key node analysis (upstream & downstream)

Upstream Key Nodes							
**Molecule name**	**Molecule classification**	**#Hits in network**	**Hits list**	**Distance**	**Score**	**Z-Score**	**FDR**
CKII-alpha: CKII-alpha2: (CKII-beta)2		257	5'-nucleotidase, 5-HT-1A, 6-phosphogluconolactonase, A1R, A2A, ABP-280, AC2, ACCalpha, ACCbeta, ActR-I, ActR-IB, AIRE-isoform1, AKT-1, Albumin, ALEX, alpha-actin, alpha2A-AR, alpha2B-AR, angiotensinogen, Apo-AI, ApoE3, ApoE4, APP695, APP751, APP770, APT1, Aquaporin-5, Aquaporin7, AR-isoform1, ARG, AT2, AXL, B56alpha, Bcl-2-alpha, Beta-4A, beta-arrestin2-isoform1, beta-catenin, beta1-AR, beta3-AR, betaARK-1, betaENaC, Bid-isoform1, BMAL1, c-Cbl, c-FLIP-L, c-FLIP-R, c-FLIP-S, c-Jun, c1r, C3aR	3	24.039	2.47355	0.002
huntingtin	huntingtin; huntingtin(h)	259	14-3-3gamma, 5'-nucleotidase, 5-HT-1A, 6-phosphogluconolactonase, A1R, A2A, ABP-280, AC2, ACCalpha, ACCbeta, ActR-I, ActR-IB, AIRE-isoform1, AKT-1, Albumin, ALEX, alpha-actin, alpha2A-AR, alpha2B-AR, angiotensinogen, Apo-AI, ApoE3, ApoE4, APP695, APP751, APP770, APT1, Aquaporin-5, Aquaporin7, AR-isoform1, ARG, AT2, AXL, B56alpha, Bcl-2-alpha, Beta-4A, beta-arrestin2-isoform1, beta-catenin, beta1-AR, beta3-AR, betaARK-1, betaENaC, Bid-isoform1, BMAL1, c-Cbl, c-FLIP-L, c-FLIP-R, c-FLIP-S, c-Jun, c1r	3	23.5238	1.99418	0.008
MKP-1	enzymes; hydrolases EC 3; hydrolases EC 3.1; phosphoric monoester hydrolases EC 3.1.3; protein phosphatases; MAPK phosphatases; MKP-1	244	5'-nucleotidase, 5-HT-1A, 6-phosphogluconolactonase, A1R, A2A, ABP-280, AC2, ACCalpha, ACCbeta, ActR-I, ActR-IB, AIRE-isoform1, AKT-1, Albumin, ALEX, alpha-actin, alpha2A-AR, alpha2B-AR, angiotensinogen, Apo-AI, ApoE3, ApoE4, APP695, APP751, APP770, APT1, Aquaporin-5, Aquaporin7, AR-isoform1, ARG, AXL, B56alpha, Bcl-2-alpha, Beta-4A, beta-arrestin2-isoform1, beta-catenin, beta1-AR, beta3-AR, betaARK-1, betaENaC, Bid-isoform1, c-Cbl, c-FLIP-L, c-FLIP-R, c-FLIP-S, c-Jun, C3aR, C5aR, CAD protein, calcyclin	3	23.0045	2.04744	0.046
SP1		243	5'-nucleotidase, 5-HT-1A, 6-phosphogluconolactonase, A1R, A2A, ABP-280, AC2, ACCalpha, ACCbeta, ActR-I, ActR-IB, AIRE-isoform1, AKT-1, Albumin, ALEX, alpha-actin, alpha2A-AR, alpha2B-AR, angiotensinogen, Apo-AI, ApoE3, ApoE4, APP695, APP751, APP770, APT1, Aquaporin-5, Aquaporin7, AR-isoform1, ARG, AT2, AXL, B56alpha, Bcl-2-alpha, Beta-4A, beta-arrestin2-isoform1, beta-catenin, beta1-AR, beta3-AR, betaARK-1, betaENaC, Bid-isoform1, BMAL1, c-Cbl, c-FLIP-L, c-FLIP-R, c-FLIP-S, c-Jun, c1r, C3aR	3	22.9974	3.66352	0
Cdk2	enzymes; transferases EC 2; transferases EC 2.7; phosphotransferases EC 2.7.1; protein kinases; protein serine/threonine kinases; CMGC family; Cdk; Cdc2 family; Cdk2; Cdk2(h)	247	5'-nucleotidase, 5-HT-1A, 6-phosphogluconolactonase, A1R, A2A, ABP-280, AC2, ACCalpha, ACCbeta, ActR-I, ActR-IB, AIRE-isoform1, AKT-1, Albumin, ALEX, alpha-actin, alpha2A-AR, alpha2B-AR, angiotensinogen, Apo-AI, ApoE3, ApoE4, APP695, APP751, APP770, APT1, Aquaporin-5, Aquaporin7, AR-isoform1, ARG, AT2, AXL, B56alpha, Bcl-2-alpha, Beta-4A, beta-arrestin2-isoform1, beta-catenin, beta1-AR, beta3-AR, betaARK-1, betaENaC, Bid-isoform1, BMAL1, c-Cbl, c-FLIP-L, c-FLIP-R, c-FLIP-S, c-Jun, c1r, C3aR	3	22.9134	2.23272	0
VHR	enzymes; hydrolases EC 3; hydrolases EC 3.1; phosphoric monoester hydrolases EC 3.1.3; protein phosphatases; MAPK phosphatases; VHR; enzymes; hydrolases EC 3; hydrolases EC 3.1; phosphoric monoester hydrolases EC 3.1.3; protein phosphatases; protein tyrosine/threonine phosphatases; VHR	240	5'-nucleotidase, 5-HT-1A, 6-phosphogluconolactonase, A1R, A2A, ABP-280, AC2, ACCalpha, ACCbeta, ActR-I, ActR-IB, AIRE-isoform1, AKT-1, Albumin, ALEX, alpha-actin, alpha2A-AR, alpha2B-AR, angiotensinogen, Apo-AI, ApoE3, ApoE4, APP695, APP751, APP770, APT1, Aquaporin-5, Aquaporin7, AR-isoform1, ARG, AXL, B56alpha, Bcl-2-alpha, Beta-4A, beta-arrestin2-isoform1, beta-catenin, beta1-AR, beta3-AR, betaARK-1, betaENaC, Bid-isoform1, c-Cbl, c-FLIP-L, c-FLIP-R, c-FLIP-S, c-Jun, C3aR, C5aR, CAD protein, calcyclin	3	22.6895	1.71896	0.04

Supplemental Table 8. Key node analysis (upstream & downstream)

MEKK1	enzymes; transferases EC 2; transferases EC 2.7; phosphotransferases EC 2.7.1; protein kinases; protein serine/threonine kinases; MAPKKKs; Ste11-like family; MEKKs; MEKK1; enzymes; transferases EC 2; transferases EC 2.7; phosphotransferases EC 2.7.1; protein kinases; protein serine/threonine kinases; STE family; Ste11-like family; MEKKs; MEKK1	237	5'-nucleotidase, 5-HT-1A, 6-phosphogluconolactonase, A1R, A2A, ABP-280, AC2, ACCalpha, ACCbeta, ActR-IB, AIRE-isoform1, AKT-1, Albumin, ALEX, alpha-actin, alpha2A-AR, alpha2B-AR, angiotensinogen, Apo-AI, ApoE3, ApoE4, APP695, APP751, APP770, APT1, Aquaporin-5, Aquaporin7, AR-isoform1, ARG, AT2, AXL, Bcl-2-alpha, beta-arrestin2-isoform1, beta-catenin, beta1-AR, beta3-AR, betaARK-1, betaENaC, Bid-isoform1, c-Cbl, c-FLIP-L, c-FLIP-R, c-FLIP-S, c-Jun, c1r, C3aR, C5aR, CAD protein, calcyclin, Calnexin	3	22.119	2.8265	0
AT2	membrane-transducing components; receptors; GPCR; rhodopsin like receptors (type A); peptide receptors; angiotensin receptors; AT2 receptor	236	5'-nucleotidase, 5-HT-1A, 6-phosphogluconolactonase, A1R, A2A, ABP-280, AC2, ACCalpha, ACCbeta, AIRE-isoform1, AKT-1, Albumin, ALEX, alpha-actin, alpha2A-AR, alpha2B-AR, angiotensinogen, Apo-AI, ApoE3, ApoE4, APP695, APP751, APP770, APT1, Aquaporin-5, Aquaporin7, AR-isoform1, ARG, AXL, B56alpha, Bcl-2-alpha, beta-arrestin2-isoform1, beta-catenin, beta1-AR, beta3-AR, betaARK-1, betaENaC, Bid-isoform1, c-Cbl, c-FLIP-L, c-FLIP-R, c-FLIP-S, c-Jun, c1r, C3aR, C5aR, CAD protein, calcyclin, Calnexin, CAR	3	21.9405	1.78623	0.036
proCaspase-1	enzymes; hydrolases EC 3; peptidases EC 3.4; cysteine endopeptidase EC 3.4.22; proCaspases; proCaspase-1	234	5'-nucleotidase, 5-HT-1A, 6-phosphogluconolactonase, A1R, A2A, ABP-280, AC2, ACCalpha, ACCbeta, ActR-I, ActR-IB, AKT-1, ALEX, alpha-actin, alpha2A-AR, alpha2B-AR, angiotensinogen, Apo-AI, ApoE3, ApoE4, APP695, APP751, APP770, APT1, Aquaporin-5, Aquaporin7, AR-isoform1, AXL, B56alpha, Bcl-2-alpha, Beta-4A, beta-arrestin2-isoform1, beta-catenin, beta1-AR, beta3-AR, betaARK-1, betaENaC, Bid-isoform1, c-Cbl, c-FLIP-L, c-FLIP-R, c-FLIP-S, c-Jun, C3aR, C5aR, CAD protein, calcyclin, Calnexin, CAR	3	21.701	1.95307	0.014
c-FLIP-L	inhibitors; intracellular inhibitors; FLIPs; c-FLIPs; c-FLIP; c-FLIP(h) (main classification); inhibitors; intracellular inhibitors; FLIPs; c-FLIPs; c-FLIP; c-FLIP-L (main classification); proto-oncogene; c-FLIPs; c-FLIP; c-FLIP(h); proto-oncogene; c-FLIPs; c-FLIP; c-FLIP-L	233	5'-nucleotidase, 5-HT-1A, 6-phosphogluconolactonase, A1R, A2A, ABP-280, AC2, ACCalpha, ACCbeta, ActR-IB, AIRE-isoform1, AKT-1, Albumin, ALEX, alpha-actin, alpha2A-AR, alpha2B-AR, angiotensinogen, Apo-AI, ApoE3, ApoE4, APP695, APP751, APP770, APT1, Aquaporin-5, Aquaporin7, AR-isoform1, ARG, AXL, B56alpha, Bcl-2-alpha, beta-catenin, beta1-AR, beta3-AR, betaARK-1, betaENaC, Bid-isoform1, c-Cbl, c-FLIP-R, c-FLIP-S, c-Jun, C3aR, C5aR, CAD protein, calcyclin, Calnexin, CAR, Caspase-8, cathepsinB	3	21.5386	1.63412	0.036
Cot-58kDa	enzymes; transferases EC 2; transferases EC 2.7; phosphotransferases EC 2.7.1; protein kinases; protein serine/threonine kinases; MAPKKKs; Ste11-like family; Cot; Cot(h); enzymes; transferases EC 2; transferases EC 2.7; phosphotransferases EC 2.7.1; protein kinases; protein serine/threonine kinases; STE family; Ste11-like family; Cot; Cot(h); oncoprotein; Cot; Cot(h)	229	5'-nucleotidase, 5-HT-1A, 6-phosphogluconolactonase, A1R, A2A, ABP-280, AC2, ACCalpha, ACCbeta, ActR-IB, AIRE-isoform1, AKT-1, Albumin, ALEX, alpha-actin, alpha2A-AR, alpha2B-AR, angiotensinogen, Apo-AI, ApoE3, ApoE4, APP695, APP751, APP770, APT1, Aquaporin-5, Aquaporin7, AR-isoform1, ARG, AXL, B56alpha, Bcl-2-alpha, Beta-4A, beta-catenin, beta1-AR, beta3-AR, betaARK-1, betaENaC, Bid-isoform1, c-Cbl, c-FLIP-L, c-FLIP-R, c-FLIP-S, c-Jun, C3aR, C5aR, CAD protein, calcyclin, Calnexin, CAR	3	21.4929	1.85361	0.018

Supplemental Table 8. Key node analysis (upstream & downstream)

Egr-1	Egr(h); Egr-1(h); transcription factors; Zinc-coordinating DNA-binding domains; Cys2His2 zinc finger domain; Developmental / cell cycle regulators; Egr/Krox; Egr-1; Egr-1(h)	232	5'-nucleotidase, 5-HT-1A, 6-phosphogluconolactonase, A1R, A2A, ABP-280, AC2, ACCalpha, ACCbeta, ActR-I, ActR-IB, AKT-1, Albumin, ALEX, alpha-actin, alpha2A-AR, alpha2B-AR, angiotensinogen, Apo-AI, ApoE3, ApoE4, APP695, APP751, APP770, APT1, Aquaporin-5, Aquaporin7, AR-isoform1, ARG, AT2, AXL, B56alpha, Bcl-2-alpha, Beta-4A, beta-arrestin2-isoform1, beta-catenin, beta1-AR, beta3-AR, betaARK-1, betaENaC, Bid-isoform1, c-Cbl, c-FLIP-L, c-FLIP-R, c-FLIP-S, c-Jun, C3aR, C5aR, CAD protein, calcyclin	3	21.3258	1.64773	0.034
SGK-1-isoform1	enzymes; transferases EC 2; transferases EC 2.7; phosphotransferases EC 2.7.1; protein kinases; protein serine/threonine kinases; AGC kinases; SGK family; SGK-1; SGK-1(h)	230	5'-nucleotidase, 5-HT-1A, 6-phosphogluconolactonase, A1R, A2A, ABP-280, AC2, ACCalpha, ACCbeta, ActR-I, AKT-1, Albumin, ALEX, alpha-actin, alpha2A-AR, alpha2B-AR, angiotensinogen, Apo-AI, ApoE3, ApoE4, APP695, APP751, APP770, APT1, Aquaporin-5, Aquaporin7, AR-isoform1, AXL, B56alpha, Bcl-2-alpha, Beta-4A, beta-arrestin2-isoform1, beta-catenin, beta1-AR, beta3-AR, betaARK-1, betaENaC, Bid-isoform1, c-Cbl, c-FLIP-L, c-FLIP-R, c-FLIP-S, c-Jun, C3aR, C5aR, CAD protein, calcyclin, Calnexin, CAR, Caspase-8	4	20.9446	1.63158	0.014
TC-PTPa	enzymes; hydrolases EC 3; hydrolases EC 3.1; phosphoric monoester hydrolases EC 3.1.3; protein phosphatases; protein tyrosine phosphatases; TC-PTP; TC-PTPa; enzymes; hydrolases EC 3; hydrolases EC 3.1; phosphoric monoester hydrolases EC 3.1.3; protein phosphatases; protein tyrosine phosphatases; TC-PTP; TC-PTP(h)	219	5'-nucleotidase, 5-HT-1A, 6-phosphogluconolactonase, A1R, A2A, ABP-280, AC2, ACCalpha, ACCbeta, AIRE-isoform1, AKT-1, Albumin, ALEX, alpha-actin, alpha2A-AR, alpha2B-AR, angiotensinogen, Apo-AI, ApoE3, ApoE4, APP695, APP751, APP770, APT1, Aquaporin7, AR-isoform1, ARG, AT2, AXL, B56alpha, Bcl-2-alpha, beta-catenin, beta1-AR, beta3-AR, betaARK-1, betaENaC, Bid-isoform1, c-Cbl, c-Jun, c1r, C3aR, C5aR, CAD protein, calcyclin, Calnexin, Caspase-8, cathepsinB, CBP, ccl26, CCR2A	3	20.8382	2.00796	0.004
TC-PTPb	enzymes; hydrolases EC 3; hydrolases EC 3.1; phosphoric monoester hydrolases EC 3.1.3; protein phosphatases; protein tyrosine phosphatases; TC-PTP; TC-PTPb	218	5'-nucleotidase, 5-HT-1A, 6-phosphogluconolactonase, A1R, A2A, ABP-280, AC2, ACCalpha, ACCbeta, AIRE-isoform1, AKT-1, Albumin, ALEX, alpha-actin, alpha2A-AR, alpha2B-AR, angiotensinogen, Apo-AI, ApoE3, ApoE4, APP695, APP751, APP770, APT1, Aquaporin7, AR-isoform1, ARG, AT2, AXL, B56alpha, Bcl-2-alpha, beta-catenin, beta1-AR, beta3-AR, betaARK-1, betaENaC, Bid-isoform1, c-Cbl, c-Jun, c1r, C3aR, C5aR, CAD protein, calcyclin, Calnexin, Caspase-8, cathepsinB, CBP, ccl26, CCR2A	3	20.7881	2.28245	0
EP300		224	5'-nucleotidase, 5-HT-1A, 6-phosphogluconolactonase, A1R, A2A, ABP-280, AC2, ACCalpha, ACCbeta, ActR-I, ActR-IB, AKT-1, Albumin, ALEX, alpha-actin, alpha2A-AR, alpha2B-AR, angiotensinogen, Apo-AI, ApoE3, ApoE4, APP695, APP751, APP770, APT1, Aquaporin-5, Aquaporin7, AR-isoform1, AT2, AXL, B56alpha, Bcl-2-alpha, Beta-4A, beta-arrestin2-isoform1, beta-catenin, beta1-AR, beta3-AR, betaARK-1, betaENaC, Bid-isoform1, c-Cbl, c-FLIP-L, c-FLIP-R, c-FLIP-S, c-Jun, C3aR, CAD protein, calcyclin, CAR, Caspase-8	4	20.5639	1.44596	0.024
IL-4R		215	5'-nucleotidase, 5-HT-1A, 6-phosphogluconolactonase, A1R, A2A, ABP-280, AC2, ACCalpha, ACCbeta, ActR-I, AIRE-isoform1, AKT-1, Albumin, ALEX, alpha-actin, alpha2A-AR, alpha2B-AR, angiotensinogen, Apo-AI, ApoE3, ApoE4, APP695, APP751, APP770, APT1, Aquaporin7, AR-isoform1, ARG, AXL, B56alpha, Bcl-2-alpha, beta-catenin, beta1-AR, beta3-AR, betaARK-1, betaENaC, Bid-isoform1, c-Cbl, c-Jun, C3aR, C5aR, CAD protein, calcyclin, Calnexin, cathepsinB, CBP, ccl26, CCR4, CCR7, CD14	3	20.4799	3.03722	0

Supplemental Table 8. Key node analysis (upstream & downstream)

p300{sumo}		223	5'-nucleotidase, 5-HT-1A, 6-phosphogluconolactonase, A1R, A2A, ABP-280, AC2, ACCalpha, ACCbeta, ActR-I, ActR-IB, AKT-1, Albumin, ALEX, alpha-actin, alpha2A-AR, alpha2B-AR, angiotensinogen, Apo-AI, ApoE3, ApoE4, APP695, APP751, APP770, APT1, Aquaporin-5, Aquaporin7, AR-isoform1, AT2, AXL, B56alpha, Bcl-2-alpha, Beta-4A, beta-arrestin2-isoform1, beta-catenin, beta1-AR, beta3-AR, betaARK-1, betaENaC, Bid-isoform1, c-Cbl, c-FLIP-L, c-FLIP-R, c-FLIP-S, c-Jun, C3aR, CAD protein, calcyclin, CAR, Caspase-8	4	20.4729	1.32876	0.048
Raf-1-isoform1	enzymes; transferases EC 2; transferases EC 2.7; phosphotransferases EC 2.7.1; protein kinases; protein serine/threonine kinases; MAPKKKs; Raf; Raf-1; Raf-1(h) (main classification); enzymes; transferases EC 2; transferases EC 2.7; phosphotransferases EC 2.7.1; protein kinases; protein serine/threonine kinases; MAPKKKs; Raf; Raf(h); Raf-1(h); enzymes; transferases EC 2; transferases EC 2.7; phosphotransferases EC 2.7.1; protein kinases; protein serine/threonine kinases; TKL family; Raf; Raf-1; Raf-1(h) (main classification); enzymes; transferases EC 2; transferases EC 2.7; phosphotransferases EC 2.7.1; protein kinases; [illegible]	220	5'-nucleotidase, 5-HT-1A, 6-phosphogluconolactonase, A1R, A2A, ABP-280, AC2, ACCalpha, ACCbeta, AIRE-isoform1, AKT-1, Albumin, ALEX, alpha-actin, alpha2A-AR, alpha2B-AR, angiotensinogen, Apo-AI, ApoE3, ApoE4, APP695, APP751, APP770, APT1, Aquaporin7, AR-isoform1, ARG, AXL, B56alpha, Bcl-2-alpha, beta-catenin, beta1-AR, beta3-AR, betaARK-1, betaENaC, Bid-isoform1, c-Cbl, c-FLIP-L, c-FLIP-R, c-FLIP-S, c-Jun, C3aR, C5aR, CAD protein, calcyclin, Calnexin, Caspase-8, cathepsinB, CBP, CCR4	3	20.3357	1.41346	0.038
MAPKAPK2	enzymes; transferases EC 2; transferases EC 2.7; phosphotransferases EC 2.7.1; protein kinases; protein serine/threonine kinases; CaMK homolog family; MAPKAPK homolog family; MAPKAPK; MAPKAPK2	224	14-3-3eta, 14-3-3gamma, 5'-nucleotidase, 5-HT-1A, 6-phosphogluconolactonase, A1R, A2A, ABP-280, AC2, ACCalpha, ACCbeta, ActR-I, ActR-IB, AKT-1, Albumin, ALEX, ALL1-isoform1, alpha-actin, alpha2A-AR, alpha2B-AR, angiotensinogen, Apo-AI, ApoE3, ApoE4, APP695, APP751, APP770, APT1, Aquaporin-5, Aquaporin7, AR-isoform1, ARG, AXL, Bcl-2-alpha, Bcl-G-L, Bcl-G-S, beta-catenin, beta1-AR, beta3-AR, betaARK-1, betaENaC, Bid-isoform1, c-Cbl, c-FLIP-L, c-FLIP-R, c-FLIP-S, c-Jun, C3aR, CAD protein, calcyclin	4	20.2013	2.12789	0.002
MEKK3	enzymes; transferases EC 2; transferases EC 2.7; phosphotransferases EC 2.7.1; protein kinases; protein serine/threonine kinases; MAPKKKs; Ste11-like family; MEKKs; MEKK3; enzymes; transferases EC 2; transferases EC 2.7; phosphotransferases EC 2.7.1; protein kinases; protein serine/threonine kinases; STE family; Ste11-like family; MEKKs; MEKK3	211	5'-nucleotidase, 5-HT-1A, 6-phosphogluconolactonase, A1R, A2A, ABP-280, AC2, ACCalpha, ACCbeta, AIRE-isoform1, AKT-1, Albumin, ALEX, alpha-actin, alpha2A-AR, alpha2B-AR, angiotensinogen, Apo-AI, ApoE3, ApoE4, APP695, APP751, APP770, APT1, Aquaporin-5, Aquaporin7, AR-isoform1, ARG, AXL, Bcl-2-alpha, beta-catenin, beta1-AR, beta3-AR, betaARK-1, betaENaC, Bid-isoform1, c-Cbl, c-FLIP-L, c-FLIP-R, c-FLIP-S, c-Jun, C3aR, C5aR, CAD protein, calcyclin, Calnexin, CAR, Caspase-8, cathepsinB, CBP	3	20.0611	3.1166	0
PRKCD		221	5'-nucleotidase, 5-HT-1A, A1R, A2A, ABP-280, AC2, ACCalpha, ACCbeta, ActR-IB, AKT-1, Albumin, ALEX, alpha-actin, alpha2A-AR, alpha2B-AR, angiotensinogen, Apo-AI, ApoE3, ApoE4, APP695, APP751, APP770, APT1, Aquaporin-5, Aquaporin7, AR-isoform1, ARG, AXL, B56alpha, Bcl-2-alpha, beta-arrestin2-isoform1, beta-catenin, beta1-AR, beta3-AR, betaARK-1, betaENaC, Bid-isoform1, c-Cbl, c-FLIP-L, c-FLIP-R, c-FLIP-S, c-Jun, C3aR, C5aR, CAD protein, calcyclin, Calnexin, CAR, Caspase-8, cathepsinB	4	20.051	2.49932	0.004

Supplemental Table 8. Key node analysis (upstream & downstream)

DAG: PKCdelta		221	5'-nucleotidase, 5-HT-1A, A1R, A2A, ABP-280, AC2, ACCalpha, ACCbeta, ActR-IB, AKT-1, Albumin, ALEX, alpha-actin, alpha2A-AR, alpha2B-AR, angiotensinogen, Apo-AI, ApoE3, ApoE4, APP695, APP751, APP770, APT1, Aquaporin-5, Aquaporin7, AR-isoform1, ARG, AXL, B56alpha, Bcl-2-alpha, beta-arrestin2-isoform1, beta-catenin, beta1-AR, beta3-AR, betaARK-1, betaENaC, Bid-isoform1, c-Cbl, c-FLIP-L, c-FLIP-R, c-FLIP-S, c-Jun, C3aR, C5aR, CAD protein, calcyclin, Calnexin, CAR, Caspase-8, cathepsinB	4	20.024	2.38771	0.004
MEKK2	enzymes; transferases EC 2; transferases EC 2.7; phosphotransferases EC 2.7.1; protein kinases; protein serine/threonine kinases; MAPKKKs; Ste11-like family; MEKKs; MEKK2; enzymes; transferases EC 2; transferases EC 2.7; phosphotransferases EC 2.7.1; protein kinases; protein serine/threonine kinases; STE family; Ste11-like family; MEKKs; MEKK2	210	5'-nucleotidase, 5-HT-1A, 6-phosphogluconolactonase, A1R, A2A, ABP-280, AC2, ACCalpha, ACCbeta, AIRE-isoform1, AKT-1, Albumin, ALEX, alpha-actin, alpha2A-AR, alpha2B-AR, angiotensinogen, Apo-AI, ApoE3, ApoE4, APP695, APP751, APP770, APT1, Aquaporin-5, Aquaporin7, AR-isoform1, ARG, AXL, Bcl-2-alpha, beta-catenin, beta1-AR, beta3-AR, betaARK-1, betaENaC, Bid-isoform1, c-Cbl, c-FLIP-L, c-FLIP-R, c-FLIP-S, c-Jun, C3aR, C5aR, CAD protein, calcyclin, Calnexin, CAR, Caspase-8, cathepsinB, CBP	3	20.0036	3.11982	0
Jak3{pY}		197	5'-nucleotidase, 5-HT-1A, 6-phosphogluconolactonase, A1R, A2A, ABP-280, AC2, ACCalpha, ACCbeta, AIRE-isoform1, AKT-1, Albumin, ALEX, alpha-actin, alpha2A-AR, alpha2B-AR, angiotensinogen, Apo-AI, ApoE3, ApoE4, APP695, APP751, APP770, APT1, Aquaporin7, AR-isoform1, ARG, AXL, Bcl-2-alpha, beta-catenin, beta1-AR, beta3-AR, betaARK-1, betaENaC, Bid-isoform1, c-Cbl, c-Jun, C3aR, C5aR, CAD protein, calcyclin, Calnexin, cathepsinB, CBP, ccl26, CCR4, CD14, CD3zeta-isoform1, CD4, CD8A	4	19.9861	4.33066	0
JAK3		197	5'-nucleotidase, 5-HT-1A, 6-phosphogluconolactonase, A1R, A2A, ABP-280, AC2, ACCalpha, ACCbeta, AIRE-isoform1, AKT-1, Albumin, ALEX, alpha-actin, alpha2A-AR, alpha2B-AR, angiotensinogen, Apo-AI, ApoE3, ApoE4, APP695, APP751, APP770, APT1, Aquaporin7, AR-isoform1, ARG, AXL, Bcl-2-alpha, beta-catenin, beta1-AR, beta3-AR, betaARK-1, betaENaC, Bid-isoform1, c-Cbl, c-Jun, C3aR, C5aR, CAD protein, calcyclin, Calnexin, cathepsinB, CBP, ccl26, CCR4, CD14, CD3zeta-isoform1, CD4, CD8A	4	19.9779	4.32133	0
SRC-1E		214	5'-nucleotidase, 5-HT-1A, 6-phosphogluconolactonase, A1R, A2A, ABP-280, ACCalpha, ACCbeta, ActR-I, ActR-IB, AKT-1, Albumin, ALEX, alpha-actin, alpha2A-AR, alpha2B-AR, angiotensinogen, Apo-AI, ApoE3, ApoE4, APP695, APP751, APP770, APT1, Aquaporin-5, Aquaporin7, AR-isoform1, ARG, AT2, AXL, Bcl-2-alpha, beta-catenin, beta1-AR, beta3-AR, betaARK-1, betaENaC, Bid-isoform1, BMAL1, c-Cbl, c-FLIP-L, c-FLIP-R, c-FLIP-S, c-Jun, C3aR, CAD protein, calcyclin, CAR, cathepsinB, CBP, CCR4	3	19.8316	3.3331	0
SRC-1 (-Q)		214	5'-nucleotidase, 5-HT-1A, 6-phosphogluconolactonase, A1R, A2A, ABP-280, ACCalpha, ACCbeta, ActR-I, ActR-IB, AKT-1, Albumin, ALEX, alpha-actin, alpha2A-AR, alpha2B-AR, angiotensinogen, Apo-AI, ApoE3, ApoE4, APP695, APP751, APP770, APT1, Aquaporin-5, Aquaporin7, AR-isoform1, ARG, AT2, AXL, Bcl-2-alpha, beta-catenin, beta1-AR, beta3-AR, betaARK-1, betaENaC, Bid-isoform1, BMAL1, c-Cbl, c-FLIP-L, c-FLIP-R, c-FLIP-S, c-Jun, C3aR, CAD protein, calcyclin, CAR, cathepsinB, CBP, CCR4	3	19.8274	3.30929	0

Supplemental Table 8. Key node analysis (upstream & downstream)

SRC-1A		214	5'-nucleotidase, 5-HT-1A, 6-phosphogluconolactonase, A1R, A2A, ABP-280, ACCalpha, ACCbeta, ActR-I, ActR-IB, AKT-1, Albumin, ALEX, alpha-actin, alpha2A-AR, alpha2B-AR, angiotensinogen, Apo-AI, ApoE3, ApoE4, APP695, APP751, APP770, APT1, Aquaporin-5, Aquaporin7, AR-isoform1, ARG, AT2, AXL, Bcl-2-alpha, beta-catenin, beta1-AR, beta3-AR, betaARK-1, betaENaC, Bid-isoform1, BMAL1, c-Cbl, c-FLIP-L, c-FLIP-R, c-FLIP-S, c-Jun, C3aR, CAD protein, calcyclin, CAR, cathepsinB, CBP, CCR4	3	19.8213	3.29609	0
IL-2Rbeta	IL-2R; IL-2Rbeta; IL-2Rbeta(h); membrane-transducing components; receptors; cytokine receptor family; class I cytokine receptor family; IL-2Rbeta; IL-2Rbeta(h)	214	5'-nucleotidase, 5-HT-1A, 6-phosphogluconolactonase, A1R, A2A, ABP-280, AC2, ACCalpha, ACCbeta, AIRE-isoform1, AKT-1, Albumin, ALEX, alpha-actin, alpha2A-AR, alpha2B-AR, angiotensinogen, Apo-AI, ApoE3, ApoE4, APP695, APP751, APP770, APT1, Aquaporin7, AR-isoform1, ARG, AXL, B56alpha, Bcl-2-alpha, beta-catenin, beta1-AR, beta3-AR, betaARK-1, betaENaC, Bid-isoform1, c-Cbl, c-FLIP-S, c-Jun, C3aR, C5aR, CAD protein, calcyclin, Calnexin, cathepsinB, CBP, ccl26, CCR2A, CCR2B, CCR4	3	19.7715	1.6453	0.012
AKT1		209	5'-nucleotidase, 5-HT-1A, A1R, A2A, ABP-280, ACCalpha, ACCbeta, ActR-I, ActR-IB, AKT-1, Albumin, ALEX, alpha-actin, alpha2A-AR, alpha2B-AR, angiotensinogen, Apo-AI, ApoE3, ApoE4, APP695, APP751, APP770, APT1, Aquaporin-5, Aquaporin7, AR-isoform1, ARG, AXL, Bcl-2-alpha, beta-catenin, beta1-AR, beta3-AR, betaARK-1, betaENaC, Bid-isoform1, c-Cbl, c-FLIP-L, c-FLIP-R, c-FLIP-S, c-Jun, C3aR, CAD protein, calcyclin, CAR, Caspase-8, cathepsinB, CBP, CCK2, CCR4, CD14	4	19.7054	3.17914	0
pdpk2	enzymes; transferases EC 2; transferases EC 2.7; phosphotransferases EC 2.7.1; protein kinases; protein serine/threonine kinases; AGC kinases; PDK	213	5'-nucleotidase, 5-HT-1A, A1R, A2A, ABP-280, ACCalpha, ACCbeta, ActR-I, ActR-IB, AKT-1, Albumin, ALEX, alpha-actin, alpha2A-AR, alpha2B-AR, angiotensinogen, Apo-AI, ApoE3, ApoE4, APP695, APP751, APP770, APT1, Aquaporin-5, Aquaporin7, AR-isoform1, ARG, AXL, Bcl-2-alpha, beta-catenin, beta1-AR, beta3-AR, betaARK-1, betaENaC, Bid-isoform1, c-Cbl, c-FLIP-L, c-FLIP-R, c-FLIP-S, c-Jun, C3aR, C5aR, CAD protein, calcyclin, Calnexin, CAR, Caspase-8, cathepsinB, CBP, CCK2	4	19.6155	2.54856	0
PP2Calpha 2	enzymes; hydrolases EC 3; hydrolases EC 3.1; phosphoric monoester hydrolases EC 3.1.3; protein phosphatases; protein serine/threonine phosphatases; PP2; PP2C; PP2Calpha; PP2Calpha(h)	212	5'-nucleotidase, 5-HT-1A, A1R, A2A, ABP-280, AC2, ACCalpha, ACCbeta, AIRE-isoform1, AKT-1, Albumin, ALEX, alpha-actin, alpha2A-AR, alpha2B-AR, angiotensinogen, Apo-AI, ApoE3, ApoE4, APP695, APP751, APP770, APT1, Aquaporin-5, Aquaporin7, AR-isoform1, ARG, AXL, Bcl-2-alpha, Beta-4A, beta-catenin, beta1-AR, beta3-AR, betaARK-1, betaENaC, Bid-isoform1, c-Cbl, c-FLIP-L, c-FLIP-R, c-FLIP-S, c-Jun, C3aR, C5aR, CAD protein, calcyclin, CAR, Caspase-8, cathepsinB, CBP, CCR4	4	19.5704	1.29949	0.046
ILK	enzymes; transferases EC 2; transferases EC 2.7; phosphotransferases EC 2.7.1; protein kinases; protein serine/threonine kinases; ILK	217	5'-nucleotidase, 5-HT-1A, A1R, A2A, ABP-280, AC2, ACCalpha, ACCbeta, ActR-I, ActR-IB, AKT-1, Albumin, ALEX, alpha-actin, alpha2A-AR, alpha2B-AR, angiotensinogen, Apo-AI, ApoE3, ApoE4, APP695, APP751, APP770, APT1, Aquaporin-5, Aquaporin7, AR-isoform1, ARG, AXL, Bcl-2-alpha, beta-catenin, beta1-AR, beta3-AR, betaARK-1, betaENaC, Bid-isoform1, c-Cbl, c-FLIP-L, c-FLIP-R, c-FLIP-S, c-Jun, C3aR, C5aR, CAD protein, calcyclin, CAR, Caspase-8, cathepsinB, CBP, CCK2	4	19.5187	1.63782	0.022
DUSP1		203	5'-nucleotidase, 5-HT-1A, 6-phosphogluconolactonase, A1R, A2A, ABP-280, AC2, ACCalpha, ACCbeta, AIRE-isoform1, AKT-1, Albumin, ALEX, alpha-actin, alpha2A-AR, alpha2B-AR, angiotensinogen, Apo-AI, ApoE3, ApoE4, APP695, APP751, APP770, APT1, Aquaporin-5, Aquaporin7, AR-isoform1, ARG, AXL, Bcl-2-alpha, beta-catenin, beta1-AR, beta3-AR, betaARK-1, betaENaC, Bid-isoform1, c-Cbl, c-FLIP-L, c-FLIP-R, c-FLIP-S, c-Jun, C3aR, C5aR, CAD protein, calcyclin, Calnexin, CAR, Caspase-8, cathepsinB, CBP	4	19.4965	3.94211	0

Supplemental Table 8. Key node analysis (upstream & downstream)

RIPK2		195	5'-nucleotidase, 5-HT-1A, 6-phosphogluconolactonase, A1R, A2A, ABP-280, AC2, ACCalpha, ACCbeta, AIRE-isoform1, AKT-1, Albumin, ALEX, alpha-actin, alpha2A-AR, alpha2B-AR, angiotensinogen, Apo-AI, ApoE3, ApoE4, APP695, APP751, APP770, APT1, Aquaporin-5, Aquaporin7, AR-isoform1, ARG, AXL, Bcl-2-alpha, beta-catenin, beta1-AR, beta3-AR, betaARK-1, betaENaC, Bid-isoform1, c-Cbl, c-FLIP-L, c-FLIP-R, c-FLIP-S, c-Jun, C3aR, CAD protein, calcyclin, Calnexin, Caspase-8, cathepsinB, CBP, CCR4, CCR6	4	19.4203	4.76854	0
KSR	adaptor proteins; KSR	202	5'-nucleotidase, 5-HT-1A, 6-phosphogluconolactonase, A1R, A2A, ABP-280, AC2, ACCalpha, ACCbeta, AIRE-isoform1, AKT-1, Albumin, ALEX, alpha-actin, alpha2A-AR, alpha2B-AR, angiotensinogen, Apo-AI, ApoE3, ApoE4, APP695, APP751, APP770, APT1, Aquaporin7, AR-isoform1, ARG, AXL, Bcl-2-alpha, beta-catenin, beta1-AR, beta3-AR, betaARK-1, betaENaC, Bid-isoform1, c-Cbl, c-Jun, C3aR, C5aR, CAD protein, calcyclin, Calnexin, Caspase-8, cathepsinB, CBP, CD14, CD3zeta-isoform1, CD4, CD8A, Cdc14A2	4	19.3032	3.68362	0
gamma-c	IL-2R; gamma-c; membrane-transducing components; receptors; cytokine receptor family; class I cytokine receptor family; gamma-c	196	5'-nucleotidase, 5-HT-1A, 6-phosphogluconolactonase, A1R, A2A, ABP-280, AC2, ACCalpha, ACCbeta, AIRE-isoform1, AKT-1, Albumin, ALEX, alpha-actin, alpha2A-AR, alpha2B-AR, angiotensinogen, Apo-AI, ApoE3, ApoE4, APP695, APP751, APP770, APT1, Aquaporin7, AR-isoform1, ARG, AXL, Bcl-2-alpha, beta-catenin, beta1-AR, beta3-AR, betaARK-1, betaENaC, Bid-isoform1, c-Cbl, c-Jun, C3aR, C5aR, CAD protein, calcyclin, Calnexin, cathepsinB, CBP, ccl26, CCR4, CD14, CD3zeta-isoform1, CD4, CD8A	4	19.147	4.00192	0
MAP2K1		190	5'-nucleotidase, 5-HT-1A, 6-phosphogluconolactonase, A1R, A2A, ABP-280, AC2, ACCalpha, ACCbeta, AIRE-isoform1, AKT-1, Albumin, ALEX, alpha-actin, alpha2A-AR, alpha2B-AR, angiotensinogen, Apo-AI, ApoE3, ApoE4, APP695, APP751, APP770, APT1, Aquaporin7, AR-isoform1, ARG, AXL, Bcl-2-alpha, beta-catenin, beta1-AR, beta3-AR, betaARK-1, betaENaC, Bid-isoform1, c-Cbl, c-Jun, C3aR, C5aR, CAD protein, calcyclin, Calnexin, cathepsinB, CBP, CD14, CD4, CD8A, complement C3, corticosteroid 11-beta-dehydrogenase isozyme 1, Cx43	4	19.1324	5.0623	0
DUSP7		193	5'-nucleotidase, 5-HT-1A, 6-phosphogluconolactonase, A1R, A2A, ABP-280, AC2, ACCalpha, ACCbeta, AIRE-isoform1, AKT-1, Albumin, ALEX, alpha-actin, alpha2A-AR, alpha2B-AR, angiotensinogen, Apo-AI, ApoE3, ApoE4, APP695, APP751, APP770, APT1, Aquaporin7, AR-isoform1, ARG, AXL, Bcl-2-alpha, beta-catenin, beta1-AR, beta3-AR, betaARK-1, betaENaC, Bid-isoform1, c-Cbl, c-Jun, C3aR, C5aR, CAD protein, calcyclin, Calnexin, CAR, Caspase-8, cathepsinB, CBP, CD14, CD4, CD8A, complement C3	4	19.1207	4.55538	0
DUSP9		193	5'-nucleotidase, 5-HT-1A, 6-phosphogluconolactonase, A1R, A2A, ABP-280, AC2, ACCalpha, ACCbeta, AIRE-isoform1, AKT-1, Albumin, ALEX, alpha-actin, alpha2A-AR, alpha2B-AR, angiotensinogen, Apo-AI, ApoE3, ApoE4, APP695, APP751, APP770, APT1, Aquaporin7, AR-isoform1, ARG, AXL, Bcl-2-alpha, beta-catenin, beta1-AR, beta3-AR, betaARK-1, betaENaC, Bid-isoform1, c-Cbl, c-Jun, C3aR, C5aR, CAD protein, calcyclin, Calnexin, CAR, Caspase-8, cathepsinB, CBP, CD14, CD4, CD8A, complement C3	4	19.1207	4.60937	0
proCaspase-2-1L	enzymes; hydrolases EC 3; peptidases EC 3.4; cysteine endopeptidase EC 3.4.22; proCaspases; proCaspase-2; proCaspase-2(h)	205	5'-nucleotidase, 5-HT-1A, A1R, A2A, ABP-280, AC2, ACCalpha, ACCbeta, AKT-1, Albumin, ALEX, alpha-actin, alpha2A-AR, alpha2B-AR, angiotensinogen, Apo-AI, ApoE3, ApoE4, APP695, APP751, APP770, APT1, Aquaporin-5, Aquaporin7, AR-isoform1, AXL, B56alpha, Bcl-2-alpha, Beta-4A, beta-arrestin2-isoform1, beta-catenin, beta1-AR, beta3-AR, betaARK-1, Bid-isoform1, c-Cbl, c-FLIP-L, c-FLIP-R, c-FLIP-S, c-Jun, C3aR, C5aR, CAD protein, calcyclin, Calnexin, CAR, Caspase-8, cathepsinB, CBP, CCK2	4	19.101	1.91508	0.014

Supplemental Table 8. Key node analysis (upstream & downstream)

proCaspase-2L-Pro	enzymes; hydrolases EC 3; peptidases EC 3.4; cysteine endopeptidase EC 3.4.22; proCaspases; proCaspase-2; proCaspase-2(h)	205	5'-nucleotidase, 5-HT-1A, A1R, A2A, ABP-280, AC2, ACCalpha, ACCbeta, AKT-1, Albumin, ALEX, alpha-actin, alpha2A-AR, alpha2B-AR, angiotensinogen, Apo-AI, ApoE3, ApoE4, APP695, APP751, APP770, APT1, Aquaporin-5, Aquaporin7, AR-isoform1, AXL, B56alpha, Bcl-2-alpha, Beta-4A, beta-arrestin2-isoform1, beta-catenin, beta1-AR, beta3-AR, betaARK-1, Bid-isoform1, c-Cbl, c-FLIP-L, c-FLIP-R, c-FLIP-S, c-Jun, C3aR, C5aR, CAD protein, calcyclin, Calnexin, CAR, Caspase-8, cathepsinB, CBP, CCK2	4	19.0986	1.92693	0.014
proCaspase-2-1S	enzymes; hydrolases EC 3; peptidases EC 3.4; cysteine endopeptidase EC 3.4.22; proCaspases; proCaspase-2; proCaspase-2(h)	205	5'-nucleotidase, 5-HT-1A, A1R, A2A, ABP-280, AC2, ACCalpha, ACCbeta, AKT-1, Albumin, ALEX, alpha-actin, alpha2A-AR, alpha2B-AR, angiotensinogen, Apo-AI, ApoE3, ApoE4, APP695, APP751, APP770, APT1, Aquaporin-5, Aquaporin7, AR-isoform1, AXL, B56alpha, Bcl-2-alpha, Beta-4A, beta-arrestin2-isoform1, beta-catenin, beta1-AR, beta3-AR, betaARK-1, Bid-isoform1, c-Cbl, c-FLIP-L, c-FLIP-R, c-FLIP-S, c-Jun, C3aR, C5aR, CAD protein, calcyclin, Calnexin, CAR, Caspase-8, cathepsinB, CBP, CCK2	4	19.0898	1.88738	0.014
MSK1	enzymes; transferases EC 2; transferases EC 2.7; phosphotransferases EC 2.7.1; protein kinases; protein serine/threonine kinases; AGC kinases; Rsk homolog family; MSK1; MSK1(h)	202	14-3-3eta, 14-3-3gamma, 5'-nucleotidase, 6-phosphogluconolactonase, A1R, A2A, ABP-280, ACCalpha, ActR-I, ActR-IB, AKT-1, Albumin, ALL1-isoform1, alpha-actin, alpha2A-AR, angiotensinogen, Apo-AI, ApoE3, ApoE4, APP695, APP751, APP770, APT1, Aquaporin7, AR-isoform1, ARG, AT2, B56alpha, Bcl-2-alpha, Bcl-G-L, Bcl-G-S, beta-arrestin2-isoform1, beta-catenin, beta1-AR, beta3-AR, betaARK-1, betaENaC, Bid-isoform1, c-Cbl, c-Jun, c1r, C3aR, C5aR, CAD protein, calcyclin, Calnexin, Caspase-8, cathepsinB, CBP, CCK2	3	19.0651	1.92994	0.02
DUSP4		198	5'-nucleotidase, 5-HT-1A, 6-phosphogluconolactonase, A1R, A2A, ABP-280, AC2, ACCalpha, ACCbeta, AIRE-isoform1, AKT-1, Albumin, ALEX, alpha-actin, alpha2A-AR, alpha2B-AR, angiotensinogen, Apo-AI, ApoE3, ApoE4, APP695, APP751, APP770, APT1, Aquaporin-5, Aquaporin7, AR-isoform1, ARG, AXL, Bcl-2-alpha, beta-catenin, beta1-AR, beta3-AR, betaARK-1, betaENaC, Bid-isoform1, c-Cbl, c-FLIP-L, c-FLIP-R, c-FLIP-S, c-Jun, C3aR, C5aR, CAD protein, calcyclin, Calnexin, Caspase-8, cathepsinB, CBP, CD14	4	19.0075	4.78821	0
MAP2K2		187	5'-nucleotidase, 5-HT-1A, 6-phosphogluconolactonase, A1R, A2A, ABP-280, AC2, ACCalpha, ACCbeta, AIRE-isoform1, AKT-1, Albumin, ALEX, alpha-actin, alpha2A-AR, alpha2B-AR, angiotensinogen, Apo-AI, ApoE3, ApoE4, APP695, APP751, APP770, APT1, Aquaporin7, AR-isoform1, ARG, AXL, Bcl-2-alpha, beta-catenin, beta1-AR, beta3-AR, betaARK-1, betaENaC, Bid-isoform1, c-Cbl, c-Jun, C3aR, C5aR, CAD protein, calcyclin, Calnexin, cathepsinB, CBP, CD14, CD3zeta-isoform1, CD4, CD8A, complement C3, corticosteroid 11-beta-dehydrogenase isozyme 1	4	19.0028	5.11415	0
HIP2-isoform1	enzymes; ligases EC 6; ligases EC 6.3; acid-D-amino-acid ligases EC 6.3.2; ubiquitin ligases; E2; HIP2; HIP2(h)	206	5'-nucleotidase, 5-HT-1A, A1R, A2A, ABP-280, AC2, ACCalpha, ACCbeta, AKT-1, Albumin, ALEX, alpha-actin, alpha2A-AR, alpha2B-AR, angiotensinogen, Apo-AI, ApoE3, ApoE4, APP695, APP751, APP770, APT1, Aquaporin-5, Aquaporin7, AR-isoform1, AXL, B56alpha, Bcl-2-alpha, Beta-4A, beta-arrestin2-isoform1, beta-catenin, beta1-AR, beta3-AR, betaARK-1, Bid-isoform1, c-Cbl, c-FLIP-L, c-FLIP-R, c-FLIP-S, c-Jun, CAD protein, calcyclin, CAR, Caspase-8, cathepsinB, CBP, CCK2, CCR4, CCR6, CD14	4	18.9784	1.75238	0.022
ING1b	ING1; ING1b; ING1; ING1(h)	206	5'-nucleotidase, 5-HT-1A, 6-phosphogluconolactonase, A1R, A2A, ABP-280, ACCalpha, ACCbeta, ActR-IB, Albumin, ALEX, alpha-actin, alpha2A-AR, alpha2B-AR, angiotensinogen, Apo-AI, ApoE3, ApoE4, APP695, APP751, APP770, APT1, Aquaporin-5, Aquaporin7, AR-isoform1, ARG, AXL, B56alpha, Bcl-2-alpha, beta-arrestin2-isoform1, beta-catenin, beta1-AR, beta3-AR, betaARK-1, betaENaC, Bid-isoform1, c-Cbl, c-FLIP-L, c-FLIP-R, c-FLIP-S, c-Jun, C3aR, CAD protein, calcyclin, Calnexin, CAR, Caspase-8, cathepsinB, CBP, CCR4	4	18.9309	3.1497	0

Supplemental Table 8. Key node analysis (upstream & downstream)

DUSP3		196	5'-nucleotidase, 5-HT-1A, 6-phosphogluconolactonase, A1R, A2A, ABP-280, AC2, ACCalpha, ACCbeta, AIRE-isoform1, AKT-1, Albumin, ALEX, alpha-actin, alpha2A-AR, alpha2B-AR, angiotensinogen, Apo-AI, ApoE3, ApoE4, APP695, APP751, APP770, APT1, Aquaporin-5, Aquaporin7, AR-isoform1, ARG, AXL, Bcl-2-alpha, beta-catenin, beta1-AR, beta3-AR, betaARK-1, betaENaC, Bid-isoform1, c-Cbl, c-FLIP-L, c-FLIP-R, c-FLIP-S, c-Jun, C3aR, C5aR, CAD protein, calcyclin, Calnexin, Caspase-8, cathepsinB, CBP, CD14	4	18.871	4.79619	0
MLK2: huntingtin: hap1: NeuroD		201	5'-nucleotidase, 5-HT-1A, A1R, A2A, ABP-280, AC2, ACCalpha, ACCbeta, AKT-1, Albumin, ALEX, alpha-actin, alpha2A-AR, alpha2B-AR, angiotensinogen, Apo-AI, ApoE3, ApoE4, APP695, APP751, APP770, APT1, Aquaporin-5, Aquaporin7, AR-isoform1, AXL, B56alpha, Bcl-2-alpha, Beta-4A, beta-arrestin2-isoform1, beta-catenin, beta1-AR, beta3-AR, betaARK-1, Bid-isoform1, c-Cbl, c-FLIP-L, c-FLIP-R, c-FLIP-S, c-Jun, CAD protein, calcyclin, CAR, Caspase-8, cathepsinB, CBP, CCK2, CCR4, CCR6, CD14	4	18.6162	1.82754	0.018
B-Raf	enzymes; transferases EC 2; transferases EC 2.7; phosphotransferases EC 2.7.1; protein kinases; protein serine/threonine kinases; MAPKKKs; Raf; B-Raf; enzymes; transferases EC 2; transferases EC 2.7; phosphotransferases EC 2.7.1; protein kinases; protein serine/threonine kinases; MAPKKKs; Raf; Raf(h); enzymes; transferases EC 2; transferases EC 2.7; phosphotransferases EC 2.7.1; protein kinases; protein serine/threonine kinases; TKL family; Raf; B-Raf; enzymes; transferases EC 2; transferases EC 2.7; phosphotransferases EC 2.7.1; protein kinases; protein serine/threonine kinases; TKL family; Raf; [illegible]	191	5'-nucleotidase, 5-HT-1A, 6-phosphogluconolactonase, A1R, A2A, ABP-280, AC2, ACCalpha, ACCbeta, AIRE-isoform1, AKT-1, Albumin, ALEX, alpha-actin, alpha2A-AR, alpha2B-AR, angiotensinogen, Apo-AI, ApoE3, ApoE4, APP695, APP751, APP770, APT1, Aquaporin7, AR-isoform1, ARG, AXL, Bcl-2-alpha, beta-catenin, beta1-AR, beta3-AR, betaARK-1, betaENaC, Bid-isoform1, c-Cbl, c-Jun, C3aR, C5aR, CAD protein, calcyclin, Calnexin, cathepsinB, CBP, CD14, CD3zeta-isoform1, CD4, CD8A, complement C3, corticosteroid 11-beta-dehydrogenase isozyme 1	4	18.5518	4.91809	0
A-Raf	enzymes; transferases EC 2; transferases EC 2.7; phosphotransferases EC 2.7.1; protein kinases; protein serine/threonine kinases; MAPKKKs; Raf; A-Raf; A-Raf(h); enzymes; transferases EC 2; transferases EC 2.7; phosphotransferases EC 2.7.1; protein kinases; protein serine/threonine kinases; TKL family; Raf; A-Raf; A-Raf(h)	190	5'-nucleotidase, 5-HT-1A, 6-phosphogluconolactonase, A1R, A2A, ABP-280, AC2, ACCalpha, ACCbeta, AIRE-isoform1, AKT-1, Albumin, ALEX, alpha-actin, alpha2A-AR, alpha2B-AR, angiotensinogen, Apo-AI, ApoE3, ApoE4, APP695, APP751, APP770, APT1, Aquaporin7, AR-isoform1, ARG, AXL, Bcl-2-alpha, beta-catenin, beta1-AR, beta3-AR, betaARK-1, betaENaC, Bid-isoform1, c-Cbl, c-Jun, C3aR, C5aR, CAD protein, calcyclin, Calnexin, cathepsinB, CBP, CD14, CD4, CD8A, complement C3, corticosteroid 11-beta-dehydrogenase isozyme 1, Cx43	4	18.543	4.91858	0
DUSP2		188	5'-nucleotidase, 5-HT-1A, 6-phosphogluconolactonase, A1R, A2A, ABP-280, AC2, ACCalpha, ACCbeta, AIRE-isoform1, AKT-1, Albumin, ALEX, alpha-actin, alpha2A-AR, alpha2B-AR, angiotensinogen, Apo-AI, ApoE3, ApoE4, APP695, APP751, APP770, APT1, Aquaporin7, AR-isoform1, ARG, AXL, Bcl-2-alpha, beta-catenin, beta1-AR, beta3-AR, betaARK-1, betaENaC, Bid-isoform1, c-Cbl, c-Jun, C3aR, CAD protein, calcyclin, Calnexin, CAR, Caspase-8, cathepsinB, CBP, CD14, CD4, CD8A, complement C3, corticosteroid 11-beta-dehydrogenase isozyme 1	4	18.5048	4.46547	0

Supplemental Table 8. Key node analysis (upstream & downstream)

MKK3	enzymes; transferases EC 2; transferases EC 2.7; phosphotransferases EC 2.7.1; protein kinases; protein serine/threonine kinases; STE family; MAPKKs; SEK; MKK3; enzymes; transferases EC 2; transferases EC 2.7; phosphotransferases EC 2.7.1; protein kinases; protein tyrosine kinases; MAPKKs; SEK; MKK3	196	5'-nucleotidase, 5-HT-1A, A1R, A2A, ABP-280, AC2, ACCalpha, ACCbeta, AKT-1, Albumin, ALEX, alpha-actin, alpha2A-AR, alpha2B-AR, angiotensinogen, Apo-AI, ApoE3, ApoE4, APP695, APP751, APP770, APT1, Aquaporin7, AR-isoform1, ARG, AXL, Bcl-2-alpha, Beta-4A, beta-arrestin2-isoform1, beta-catenin, beta1-AR, beta3-AR, betaARK-1, betaENaC, Bid-isoform1, c-Cbl, c-Jun, C3aR, calcyclin, CAR, Caspase-8, cathepsinB, CBP, CCR4, CCR7, CD14, CD3zeta-isoform1, CD4, Cdc14A2, Cx43	4	18.4523	1.6823	0.022
E6AP	enzymes; ligases EC 6; ligases EC 6.3; acid-D-amino-acid ligases EC 6.3.2; ubiquitin ligases; E3; HECT proteins; E6AP	204	5'-nucleotidase, 5-HT-1A, A1R, A2A, ABP-280, ACCalpha, ACCbeta, ActR-IB, AKT-1, Albumin, ALEX, alpha-actin, alpha2A-AR, alpha2B-AR, angiotensinogen, Apo-AI, ApoE3, ApoE4, APP695, APP751, APP770, APT1, Aquaporin-5, Aquaporin7, AR-isoform1, AXL, B56alpha, Bcl-2-alpha, beta-arrestin2-isoform1, beta-catenin, beta1-AR, beta3-AR, betaARK-1, betaENaC, Bid-isoform1, c-Cbl, c-FLIP-L, c-FLIP-R, c-FLIP-S, c-Jun, C3aR, C5aR, CAD protein, calcyclin, CAR, cathepsinB, CBP, CCR7, CD14, CD4	4	18.4209	3.01774	0
PTPN5		186	5'-nucleotidase, 5-HT-1A, 6-phosphogluconolactonase, A1R, A2A, ABP-280, AC2, ACCalpha, ACCbeta, AIRE-isoform1, AKT-1, Albumin, ALEX, alpha-actin, alpha2A-AR, alpha2B-AR, angiotensinogen, Apo-AI, ApoE3, ApoE4, APP695, APP751, APP770, APT1, Aquaporin7, AR-isoform1, ARG, AXL, Bcl-2-alpha, beta-catenin, beta1-AR, beta3-AR, betaARK-1, betaENaC, Bid-isoform1, c-Cbl, c-Jun, C3aR, C5aR, CAD protein, calcyclin, Calnexin, cathepsinB, CBP, CD14, CD4, CD8A, complement C3, corticosteroid 11-beta-dehydrogenase isozyme 1, Cx43	4	18.3671	5.00303	0
PTPRR		186	5'-nucleotidase, 5-HT-1A, 6-phosphogluconolactonase, A1R, A2A, ABP-280, AC2, ACCalpha, ACCbeta, AIRE-isoform1, AKT-1, Albumin, ALEX, alpha-actin, alpha2A-AR, alpha2B-AR, angiotensinogen, Apo-AI, ApoE3, ApoE4, APP695, APP751, APP770, APT1, Aquaporin7, AR-isoform1, ARG, AXL, Bcl-2-alpha, beta-catenin, beta1-AR, beta3-AR, betaARK-1, betaENaC, Bid-isoform1, c-Cbl, c-Jun, C3aR, C5aR, CAD protein, calcyclin, Calnexin, cathepsinB, CBP, CD14, CD4, CD8A, complement C3, corticosteroid 11-beta-dehydrogenase isozyme 1, Cx43	4	18.3671	5.04898	0
p38alpha{pT180}{pY182}		188	5'-nucleotidase, 5-HT-1A, A1R, A2A, ABP-280, AC2, ACCalpha, ACCbeta, AKT-1, ALEX, alpha-actin, alpha2A-AR, alpha2B-AR, angiotensinogen, Apo-AI, ApoE3, ApoE4, APP695, APP751, APP770, APT1, Aquaporin7, AR-isoform1, ARG, AXL, Bcl-2-alpha, Beta-4A, beta-catenin, beta1-AR, beta3-AR, betaARK-1, betaENaC, Bid-isoform1, c-Cbl, c-Jun, C3aR, calcyclin, CAR, Caspase-8, cathepsinB, CBP, CCR4, CD14, CD3zeta-isoform1, CD4, Cdc14A2, Cx43, CXCR2, CYP7, D2-isoform1	4	18.3374	2.33414	0
SP3		182	5'-nucleotidase, 5-HT-1A, 6-phosphogluconolactonase, A2A, ABP-280, AC2, ACCalpha, ACCbeta, ActR-IB, AKT-1, Albumin, ALEX, alpha-actin, alpha2A-AR, alpha2B-AR, angiotensinogen, Apo-AI, ApoE3, ApoE4, APP695, APP751, APP770, APT1, AR-isoform1, AXL, B56alpha, Bcl-2-alpha, beta-arrestin2-isoform1, beta-catenin, beta1-AR, beta3-AR, betaARK-1, Bid-isoform1, BMAL1, c-Cbl, c-Jun, calcyclin, Calnexin, CaSR, cathepsinB, CBP, CCR2A, CCR2B, CD14, CD3zeta-isoform1, complement C3, corticosteroid 11-beta-dehydrogenase isozyme 1, Csk, Cx43, CXCR2	4	18.3317	3.73164	0

Supplemental Table 8. Key node analysis (upstream & downstream)

MOS		187	5'-nucleotidase, 5-HT-1A, 6-phosphogluconolactonase, A1R, A2A, ABP-280, AC2, ACCalpha, ACCbeta, AIRE-isoform1, AKT-1, Albumin, ALEX, alpha-actin, alpha2A-AR, alpha2B-AR, angiotensinogen, Apo-AI, ApoE3, ApoE4, APP695, APP751, APP770, APT1, Aquaporin7, AR-isoform1, ARG, AXL, Bcl-2-alpha, beta-catenin, beta1-AR, beta3-AR, betaARK-1, betaENaC, Bid-isoform1, c-Cbl, c-Jun, C3aR, C5aR, CAD protein, calcyclin, Calnexin, cathepsinB, CBP, CD14, CD4, CD8A, complement C3, corticosteroid 11-beta-dehydrogenase isozyme 1, Cx43	4	18.3226	4.72822	0
MEK1{pS}		189	5'-nucleotidase, 5-HT-1A, 6-phosphogluconolactonase, A1R, A2A, ABP-280, AC2, ACCalpha, ACCbeta, AIRE-isoform1, AKT-1, Albumin, ALEX, alpha-actin, alpha2A-AR, alpha2B-AR, angiotensinogen, Apo-AI, ApoE3, ApoE4, APP695, APP751, APP770, APT1, Aquaporin7, AR-isoform1, ARG, AXL, Bcl-2-alpha, beta-catenin, beta1-AR, beta3-AR, betaARK-1, betaENaC, Bid-isoform1, c-Cbl, c-Jun, C3aR, C5aR, CAD protein, calcyclin, Calnexin, cathepsinB, CBP, CD14, CD4, CD8A, complement C3, corticosteroid 11-beta-dehydrogenase isozyme 1, Cx43	4	18.3079	4.90451	0
ERK1{p}; ERK2{p}: RSK1{pS380} {pT573}		189	5'-nucleotidase, 5-HT-1A, 6-phosphogluconolactonase, A1R, A2A, ABP-280, AC2, ACCalpha, ACCbeta, AIRE-isoform1, AKT-1, Albumin, ALEX, alpha-actin, alpha2A-AR, alpha2B-AR, angiotensinogen, Apo-AI, ApoE3, ApoE4, APP695, APP751, APP770, APT1, Aquaporin7, AR-isoform1, ARG, AXL, Bcl-2-alpha, beta-catenin, beta1-AR, beta3-AR, betaARK-1, betaENaC, Bid-isoform1, c-Cbl, c-Jun, C3aR, C5aR, CAD protein, calcyclin, Calnexin, cathepsinB, CBP, CD14, CD4, CD8A, complement C3, corticosteroid 11-beta-dehydrogenase isozyme 1, Cx43	4	18.2883	4.92948	0
DUSP6		186	5'-nucleotidase, 5-HT-1A, 6-phosphogluconolactonase, A1R, A2A, ABP-280, AC2, ACCalpha, ACCbeta, AIRE-isoform1, AKT-1, Albumin, ALEX, alpha-actin, alpha2A-AR, alpha2B-AR, angiotensinogen, Apo-AI, ApoE3, ApoE4, APP695, APP751, APP770, APT1, Aquaporin7, AR-isoform1, ARG, AXL, Bcl-2-alpha, beta-catenin, beta1-AR, beta3-AR, betaARK-1, betaENaC, Bid-isoform1, c-Cbl, c-Jun, C3aR, C5aR, CAD protein, calcyclin, Calnexin, cathepsinB, CBP, CD14, CD4, CD8A, complement C3, corticosteroid 11-beta-dehydrogenase isozyme 1, Cx43	4	18.267	4.97326	0
HTT		196	5'-nucleotidase, 5-HT-1A, A1R, A2A, ABP-280, AC2, ACCalpha, ACCbeta, AKT-1, Albumin, ALEX, alpha-actin, alpha2A-AR, alpha2B-AR, angiotensinogen, Apo-AI, ApoE3, ApoE4, APP695, APP751, APP770, APT1, Aquaporin-5, Aquaporin7, AR-isoform1, AXL, B56alpha, Bcl-2-alpha, Beta-4A, beta-arrestin2-isoform1, beta-catenin, beta1-AR, beta3-AR, Bid-isoform1, c-Cbl, c-FLIP-L, c-FLIP-R, c-FLIP-S, c-Jun, CAD protein, calcyclin, CAR, Caspase-8, cathepsinB, CBP, CCK2, CCR4, CCR6, CD14, CD4	4	18.1944	2.10851	0.006
calpain-2	enzymes; hydrolases EC 3; peptidases EC 3.4; calcium activated proteases; calpain; calpain-2; enzymes; hydrolases EC 3; peptidases EC 3.4; calcium activated proteases; calpain; calpain(h); enzymes; hydrolases EC 3; peptidases EC 3.4; cysteine endopeptidase EC 3.4.22; calpain; calpain-2; enzymes; hydrolases EC 3; peptidases EC 3.4; cysteine endopeptidase EC 3.4.22; calpain; calpain(h)	196	5'-nucleotidase, 5-HT-1A, A1R, A2A, ABP-280, AC2, ACCalpha, ACCbeta, AKT-1, Albumin, ALEX, alpha-actin, alpha2A-AR, alpha2B-AR, angiotensinogen, Apo-AI, ApoE3, ApoE4, APP695, APP751, APP770, APT1, Aquaporin-5, Aquaporin7, AR-isoform1, AXL, B56alpha, Bcl-2-alpha, Beta-4A, beta-arrestin2-isoform1, beta-catenin, beta1-AR, beta3-AR, Bid-isoform1, c-Cbl, c-FLIP-L, c-FLIP-R, c-FLIP-S, c-Jun, CAD protein, calcyclin, CAR, Caspase-8, cathepsinB, CBP, CCK2, CCR4, CCR6, CD14, CD4	4	18.1506	2.05204	0.006

Supplemental Table 8. Key node analysis (upstream & downstream)

p38alpha: Max		188	5'-nucleotidase, 5-HT-1A, A1R, A2A, ABP-280, AC2, ACCalpha, ACCbeta, AKT-1, ALEX, alpha-actin, alpha2A-AR, alpha2B-AR, angiotensinogen, Apo-AI, ApoE3, ApoE4, APP695, APP751, APP770, APT1, Aquaporin7, AR-isoform1, ARG, AXL, Bcl-2-alpha, Beta-4A, beta-catenin, beta1-AR, beta3-AR, betaARK-1, betaENaC, Bid-isoform1, c-Cbl, c-Jun, C3aR, calcyclin, CAR, Caspase-8, cathepsinB, CBP, CCR4, CD14, CD3zeta-isoform1, CD4, Cdc14A2, Cx43, CXCR2, CYP7, D2-isoform1	4	17.9261	1.85663	0.006

Supplemental Table 8. Key node analysis (upstream & downstream)

Downstream Key Nodes							
Molecule name	Molecule classification	#Hits in network	Hits list	Distance	Score	Z-Score	FDR
Src-isoform1	enzymes; transferases EC 2; transferases EC 2.7; phosphotransferases EC 2.7.1; protein kinases; protein tyrosine kinases; Src family; Src(h); Src(h); enzymes; transferases EC 2; transferases EC 2.7; phosphotransferases EC 2.7.1; protein kinases; protein tyrosine kinases; Src family; Src; Src(h); proto-oncogene; Src(h); Src(h); proto-oncogene; Src; Src(h)	162	14-3-3eta, 5-HT-1A, A1R, AC2, ActR-I, AKT-1, alpha2A-AR, alpha2B-AR, APP695, APP751, APP770, AR-isoform1, ARG, AT2, ATR-isoform1, Bcl-2-alpha, beta-arrestin2-isoform1, beta-catenin, beta3-AR, betaARK-1, c-Cbl, c-FLIP-L, c-FLIP-S, c-Jun, C5aR, calmodulin, Caspase-8, CBP, CCK2, CCR2A, CCR2B, CCR6, CCR7, CD14, CD3zeta-isoform1, CD4, Cdc14A2, cdk9, CHIP, Csk, CTF-1, CTF-2, CysLT1, D2-isoform1, Delta40p53, DLK, DOR, Dyrk1B, Egr-1, Elk1-isoform1	3	17.8922	0.812928	0.036
TIMP-1		151	14-3-3eta, 14-3-3gamma, 5-HT-1A, A1R, ActR-I, ActR-IB, AKT-1, angiotensinogen, APP695, APP751, APP770, AR-isoform1, ARG, AT2, ATR-isoform1, beta-arrestin2-isoform1, beta-catenin, betaARK-1, c-Cbl, c-FLIP-L, c-FLIP-S, c-Jun, calmodulin, CAR, Caspase-8, CBP, CD14, CD3zeta-isoform1, CD4, Cdc14A2, cdk9, CHIP, CNTF, Csk, CtBP1, CTF-1, CTF-2, D2-isoform1, Delta40p53, DLK, DOR, Dyrk1B, Egr-1, Elk1-isoform1, ephrin-A1, EpoR, ER-alpha-L, ER-beta-isoform1, ErbB1-p170, ERK1	3	17.1758	0.873762	0.046
ErbB1: STAT3		143	14-3-3eta, 14-3-3gamma, 5-HT-1A, A1R, ActR-I, ActR-IB, AKT-1, alpha2A-AR, alpha2B-AR, APP695, APP751, APP770, AR-isoform1, ARG, AT2, ATR-isoform1, Bcl-2-alpha, beta-arrestin2-isoform1, beta3-AR, betaARK-1, c-Cbl, c-FLIP-L, c-FLIP-S, c-Jun, C5aR, calmodulin, Caspase-8, CBP, CD14, CD3zeta-isoform1, CD4, Cdc14A2, cdk9, CHIP, CNTF, Csk, CtBP1, CTF-1, CTF-2, D2-isoform1, Delta40p53, DLK, DOR, Dyrk1B, Egr-1, Elk1-isoform1, ephrin-A1, EpoR, ER-alpha-L, ER-beta-isoform1	3	16.8926	1.19859	0.004
Pyk2-isoform1	enzymes; transferases EC 2; transferases EC 2.7; phosphotransferases EC 2.7.1; protein kinases; protein tyrosine kinases; FAK family; Pyk2; Pyk2; enzymes; transferases EC 2; transferases EC 2.7; phosphotransferases EC 2.7.1; protein kinases; protein tyrosine kinases; FAK family; Pyk2; Pyk2(h)	145	14-3-3eta, 5-HT-1A, A1R, ActR-I, ActR-IB, AKT-1, APP695, APP751, APP770, AR-isoform1, ARG, AT2, ATR-isoform1, Bcl-2-alpha, beta-arrestin2-isoform1, beta-catenin, betaARK-1, c-Cbl, c-FLIP-L, c-FLIP-S, c-Jun, calmodulin, Caspase-8, CBP, CCR2A, CCR2B, CCR6, CCR7, CD3zeta-isoform1, CD4, Cdc14A2, cdk9, CHIP, Csk, CtBP1, CTF-1, CTF-2, D2-isoform1, Delta40p53, DLK, DOR, Dyrk1B, Egr-1, Elk1-isoform1, ephrin-A1, EpoR, ER-alpha-L, ER-beta-isoform1, ErbB1-p170, ERK1	3	16.2852	1.07147	0.04
STAT3{p}		143	14-3-3eta, 14-3-3gamma, 5-HT-1A, A1R, ActR-I, ActR-IB, AKT-1, APP695, APP751, APP770, AR-isoform1, ARG, AT2, ATR-isoform1, beta-arrestin2-isoform1, betaARK-1, c-Cbl, c-FLIP-L, c-FLIP-S, c-Jun, calmodulin, Caspase-8, CBP, CCK2, CD14, CD3zeta-isoform1, CD4, Cdc14A2, cdk9, CHIP, CNTF, Csk, CtBP1, CTF-1, CTF-2, CysLT1, D2-isoform1, Delta40p53, DLK, DOR, Dyrk1B, Egr-1, Elk1-isoform1, ephrin-A1, EpoR, ER-alpha-L, ER-beta-isoform1, ErbB1-p170, ERK1, ETA	3	16.2496	1.02857	0.034
p21Cip1: STAT3		138	14-3-3eta, 14-3-3gamma, ActR-I, ActR-IB, AKT-1, APP695, APP751, APP770, AR-isoform1, ARG, AT2, ATR-isoform1, beta-arrestin2-isoform1, beta-catenin, betaARK-1, c-Cbl, c-FLIP-L, c-FLIP-S, c-Jun, calmodulin, Caspase-8, CBP, CD14, CD4, Cdc14A2, cdk9, CHIP, CNTF, Csk, CtBP1, CTF-1, CTF-2, Delta40p53, DLK, Dyrk1B, Egr-1, Elk1-isoform1, ephrin-A1, EpoR, ER-alpha-L, ER-beta-isoform1, ErbB1-p170, ERK1, FADD, FAF1, Fas, Fetua, fgfr1, fibronectin, FosB	3	16.166	1.18238	0.016

Supplemental Table 8. Key node analysis (upstream & downstream)

STAT3: p21Cip1		138	14-3-3eta, 14-3-3gamma, ActR-I, ActR-IB, AKT-1, APP695, APP751, APP770, AR-isoform1, ARG, AT2, ATR-isoform1, beta-arrestin2-isoform1, beta-catenin, betaARK-1, c-Cbl, c-FLIP-L, c-FLIP-S, c-Jun, calmodulin, Caspase-8, CBP, CD14, CD4, Cdc14A2, cdk9, CHIP, CNTF, Csk, CtBP1, CTF-1, CTF-2, Delta40p53, DLK, Dyrk1B, Egr-1, Elk1-isoform1, ephrin-A1, EpoR, ER-alpha-L, ER-beta-isoform1, ErbB1-p170, ERK1, FADD, FAF1, Fas, Fetua, fgfr1, fibronectin, FosB	3	16.166	1.18238	0.016
Syk-isoform1	enzymes; transferases EC 2; transferases EC 2.7; phosphotransferases EC 2.7.1; protein kinases; protein tyrosine kinases; ZAP-70/Syk family; Syk subfamily; Syk; Syk(h)	142	14-3-3eta, 5-HT-1A, A1R, ActR-I, ActR-IB, AKT-1, angiotensinogen, APP695, APP751, APP770, AR-isoform1, ARG, AT2, ATR-isoform1, beta-arrestin1A, beta-arrestin2-isoform1, beta-catenin, betaARK-1, c-Cbl, c-FLIP-L, c-FLIP-S, c-Jun, calmodulin, Caspase-8, CBP, CD3zeta-isoform1, CD4, Cdc14A2, cdk9, CHIP, Csk, CtBP1, CTF-1, CTF-2, D2-isoform1, Delta40p53, DLK, DOR, Dyrk1B, Egr-1, Elk1-isoform1, ephrin-A1, EpoR, ER-alpha-L, ER-beta-isoform1, ErbB1-p170, ERK1, FADD, FAF1, Fas	3	16.0434	1.03976	0.048
Smad4	transcription factors; beta-Scaffold Factors with Minor Groove Contacts; SMAD/NF-1; Smads; Co-Smads; Smad4; Smad4(h); transcription factors; beta-Scaffold Factors with Minor Groove Contacts; SMAD/NF-1; Smads; Smad(h); Smad4(h)	139	14-3-3eta, 5-HT-1A, A1R, ActR-I, ActR-IB, AKT-1, APP695, APP751, APP770, AR-isoform1, ARG, AT2, ATR-isoform1, B56alpha, Bcl-2-alpha, beta-arrestin2-isoform1, beta-catenin, betaARK-1, c-Cbl, c-FLIP-L, c-FLIP-S, c-Jun, calmodulin, Caspase-8, CBP, CD3zeta-isoform1, CD4, Cdc14A2, cdk9, CHIP, Csk, CtBP1, CTF-1, CTF-2, D2-isoform1, delta-aminolevulinic acid dehydratase, Delta40p53, DLK, DOR, Dyrk1B, Egr-1, Elk1-isoform1, ephrin-A1, EpoR, ER-alpha-L, ER-beta-isoform1, ErbB1-p170, ERK1, ERK5, FADD	4	16.0202	1.04579	0.048
ZFP36		135	14-3-3eta, 5-HT-1A, A1R, ActR-I, ActR-IB, AKT-1, angiotensinogen, APP695, APP751, APP770, AR-isoform1, ARG, AT2, ATR-isoform1, Bcl-2-alpha, beta-arrestin2-isoform1, beta-catenin, betaARK-1, c-Cbl, c-FLIP-L, c-FLIP-S, c-Jun, calmodulin, Caspase-8, CBP, CD4, Cdc14A2, cdk9, CHIP, Csk, CtBP1, CTF-1, CTF-2, D2-isoform1, Delta40p53, DLK, DOR, Dyrk1B, Egr-1, Elk1-isoform1, ephrin-A1, EpoR, ER-alpha-L, ER-beta-isoform1, ErbB1-p170, ERK1, FADD, FAF1, Fas, Fetua	3	15.8308	1.24565	0.022
Hsp90: caveolin-1: STAT3		138	14-3-3eta, 14-3-3gamma, 5-HT-1A, A1R, ActR-I, ActR-IB, AKT-1, APP695, APP751, APP770, AR-isoform1, ARG, AT2, ATR-isoform1, beta-arrestin2-isoform1, betaARK-1, c-Cbl, c-FLIP-L, c-FLIP-S, c-Jun, calmodulin, Caspase-8, CBP, CD14, CD3zeta-isoform1, CD4, Cdc14A2, cdk9, CHIP, CNTF, Csk, CtBP1, CTF-1, CTF-2, D2-isoform1, Delta40p53, DLK, DOR, Dyrk1B, Egr-1, Elk1-isoform1, ephrin-A1, EpoR, ER-alpha-L, ER-beta-isoform1, ErbB1-p170, ERK1, FADD, FAF1, Fas	4	15.7279	1.17405	0.048
caveolin-1: STAT3		138	14-3-3eta, 14-3-3gamma, 5-HT-1A, A1R, ActR-I, ActR-IB, AKT-1, APP695, APP751, APP770, AR-isoform1, ARG, AT2, ATR-isoform1, beta-arrestin2-isoform1, betaARK-1, c-Cbl, c-FLIP-L, c-FLIP-S, c-Jun, calmodulin, Caspase-8, CBP, CD14, CD3zeta-isoform1, CD4, Cdc14A2, cdk9, CHIP, CNTF, Csk, CtBP1, CTF-1, CTF-2, D2-isoform1, Delta40p53, DLK, DOR, Dyrk1B, Egr-1, Elk1-isoform1, ephrin-A1, EpoR, ER-alpha-L, ER-beta-isoform1, ErbB1-p170, ERK1, FADD, FAF1, Fas	4	15.7279	1.17405	0.048
REG1A		129	14-3-3eta, 14-3-3gamma, ActR-I, ActR-IB, AKT-1, APP695, AR-isoform1, ARG, AT2, ATR-isoform1, beta-arrestin2-isoform1, beta-catenin, betaARK-1, c-Cbl, c-FLIP-L, c-FLIP-S, c-Jun, calmodulin, Caspase-8, CBP, CD14, CD4, Cdc14A2, cdk9, CHIP, CNTF, Csk, CtBP1, CTF-1, CTF-2, Delta40p53, DLK, Dyrk1B, Egr-1, Elk1-isoform1, ephrin-A1, EpoR, ER-alpha-L, ER-beta-isoform1, ErbB1-p170, ERK1, FADD, FAF1, Fas, Fetua, fgfr1, fibronectin, FosB, gamma-c, GATA-3	4	15.5839	1.30319	0.028
PKR: STAT3		135	14-3-3eta, 14-3-3gamma, 5-HT-1A, A1R, ActR-I, ActR-IB, AKT-1, APP695, APP751, APP770, AR-isoform1, ARG, AT2, ATR-isoform1, beta-arrestin2-isoform1, betaARK-1, c-Cbl, c-FLIP-L, c-FLIP-S, c-Jun, calmodulin, Caspase-8, CBP, CD14, CD4, Cdc14A2, cdk9, CHIP, CNTF, Csk, CtBP1, CTF-1, CTF-2, D2-isoform1, Delta40p53, DLK, DOR, Dyrk1B, Egr-1, Elk1-isoform1, ephrin-A1, EpoR, ER-alpha-L, ER-beta-isoform1, ErbB1-p170, ERK1, FADD, FAF1, Fas, Fetua	4	15.5348	1.33337	0.03

Supplemental Table 8. Key node analysis (upstream & downstream)

SHP1-isoform2: ErbB1		131	14-3-3eta, 5-HT-1A, A1R, AKT-1, alpha2A-AR, alpha2B-AR, angiotensinogen, APP695, APP751, APP770, AR-isoform1, ARG, AT2, ATR-isoform1, Bcl-2-alpha, beta-arrestin2-isoform1, beta-catenin, beta3-AR, betaARK-1, c-Cbl, c-FLIP-L, c-FLIP-S, c-Jun, C5aR, calmodulin, Caspase-8, CBP, CD3zeta-isoform1, CD4, Cdc14A2, cdk9, CHIP, Csk, CTF-1, CTF-2, D2-isoform1, Delta40p53, DLK, DOR, Egr-1, Elk1-isoform1, ephrin-A1, EpoR, ER-alpha-L, ER-beta-isoform1, ErbB1-p170, ERK1, FADD, FAF1, Fas	4	15.4941	1.39481	0.016
Raf-1: pRb		131	14-3-3eta, 5-HT-1A, A1R, ActR-I, AKT-1, alpha2A-AR, alpha2B-AR, APP695, APP751, APP770, AR-isoform1, ARG, AT2, ATR-isoform1, Bcl-2-alpha, beta-arrestin2-isoform1, beta-catenin, beta3-AR, betaARK-1, Bid-isoform1, c-Cbl, c-FLIP-L, c-FLIP-S, c-Jun, C5aR, Caspase-8, CBP, CD3zeta-isoform1, CD4, Cdc14A2, cdk9, CHIP, Csk, CTF-1, CTF-2, D2-isoform1, DCR2, Delta40p53, DLK, DOR, Dyrk1B, Egr-1, Elk1-isoform1, ephrin-A1, EpoR, ER-alpha-L, ER-beta-isoform1, ErbB1-p170, ERK1, FADD	4	15.4173	1.36414	0.04
Raf-1{pS}		129	14-3-3eta, 5-HT-1A, A1R, ActR-I, ActR-IB, AKT-1, alpha2A-AR, alpha2B-AR, APP695, APP751, APP770, AR-isoform1, ARG, AT2, ATR-isoform1, beta-arrestin2-isoform1, beta3-AR, betaARK-1, Bid-isoform1, c-Cbl, c-FLIP-L, c-FLIP-S, c-Jun, C5aR, Caspase-8, CBP, CD3zeta-isoform1, CD4, Cdc14A2, cdk9, CHIP, Csk, CtBP1, CTF-1, CTF-2, D2-isoform1, DCR2, Delta40p53, DLK, DOR, Dyrk1B, Egr-1, Elk1-isoform1, ephrin-A1, EpoR, ER-alpha-L, ER-beta-isoform1, ErbB1-p170, ERK1, FADD	4	15.3647	1.20946	0.032
PKCdelta: DAG: Raf-1		130	14-3-3eta, 5-HT-1A, A1R, ActR-I, AKT-1, alpha2A-AR, alpha2B-AR, APP695, APP751, APP770, AR-isoform1, ARG, AT2, ATR-isoform1, beta-arrestin2-isoform1, beta3-AR, betaARK-1, Bid-isoform1, c-Cbl, c-FLIP-L, c-FLIP-S, c-Jun, C5aR, Caspase-8, CBP, CCK2, CD3zeta-isoform1, CD4, Cdc14A2, cdk9, CHIP, Csk, CTF-1, CTF-2, CXCR2, CysLT1, D2-isoform1, DCR2, Delta40p53, DLK, DOR, Dyrk1B, Egr-1, Elk1-isoform1, ephrin-A1, EpoR, ER-alpha-L, ER-beta-isoform1, ErbB1-p170, ERK1	4	15.3628	1.36687	0.034
EGF: (ErbB1)2		132	14-3-3eta, 5-HT-1A, A1R, AKT-1, alpha2A-AR, alpha2B-AR, angiotensinogen, APP695, APP751, APP770, AR-isoform1, ARG, AT2, ATR-isoform1, B56alpha, Bcl-2-alpha, beta-arrestin2-isoform1, beta-catenin, beta3-AR, betaARK-1, c-Cbl, c-FLIP-L, c-FLIP-S, c-Jun, C5aR, calmodulin, Caspase-8, CBP, CD3zeta-isoform1, CD4, Cdc14A2, cdk9, CHIP, Csk, CTF-1, CTF-2, D2-isoform1, Delta40p53, DLK, DOR, Egr-1, Elk1-isoform1, ephrin-A1, EpoR, ER-alpha-L, ER-beta-isoform1, ErbB1-p170, ERK1, FADD, FAF1	4	15.3601	1.37794	0.016
mmp2	enzymes; hydrolases EC 3; peptidases EC 3.4; metalloendopeptidases EC 3.4.24; MMPs; mmp2; mmp2(h)	130	14-3-3eta, 14-3-3gamma, ActR-IB, AKT-1, APP695, APP751, APP770, AR-isoform1, ARG, AT2, ATR-isoform1, Bcl-2-alpha, Beta-4A, beta-catenin, betaARK-1, c-Cbl, c-FLIP-L, c-FLIP-S, c-Jun, calmodulin, Caspase-8, CBP, CD4, Cdc14A2, cdk9, CHIP, Csk, CtBP1, CTF-1, CTF-2, delta-aminolevulinic acid dehydratase, Delta40p53, DLK, Egr-1, Elk1-isoform1, ephrin-A1, EpoR, ER-alpha-L, ER-beta-isoform1, ErbB1-p170, ERK1, ERK5, FADD, FAF1, Fas, Fetua, fgfr1, fibronectin, FosB, gamma-c	4	15.3561	1.07558	0.048
EpoR	membrane-transducing components; receptors; cytokine receptor family; class I cytokine receptor family; EpoR	136	14-3-3eta, 5-HT-1A, A1R, ActR-I, ActR-IB, AKT-1, APP695, APP751, APP770, AR-isoform1, ARG, AT2, ATR-isoform1, beta-arrestin2-isoform1, beta-catenin, betaARK-1, c-Cbl, c-FLIP-L, c-FLIP-S, c-Jun, calmodulin, Caspase-8, CBP, CCK2, CD3zeta-isoform1, CD4, Cdc14A2, cdk9, CHIP, Csk, CtBP1, CTF-1, CTF-2, CysLT1, D2-isoform1, Delta40p53, DLK, DOR, Dyrk1B, Egr-1, Elk1-isoform1, ephrin-A1, ER-alpha-L, ER-beta-isoform1, ErbB1-p170, ERK1, ETA, FADD, FAF1, Fas	4	15.3348	1.20912	0.048

Supplemental Table 8. Key node analysis (upstream & downstream)

Raf-1: H-Ras		127	14-3-3eta, 5-HT-1A, A1R, ActR-I, AKT-1, alpha2A-AR, alpha2B-AR, APP695, APP751, APP770, AR-isoform1, ARG, AT2, ATR-isoform1, beta-arrestin2-isoform1, beta3-AR, betaARK-1, Bid-isoform1, c-Cbl, c-FLIP-L, c-FLIP-S, c-Jun, C5aR, Caspase-8, CBP, CD3zeta-isoform1, CD4, Cdc14A2, cdk9, CHIP, Csk, CTF-1, CTF-2, D2-isoform1, DCR2, Delta40p53, DLK, DOR, Dyrk1B, Egr-1, Elk1-isoform1, ephrin-A1, EpoR, ER-alpha-L, ER-beta-isoform1, ErbB1-p170, ERK1, FADD, FAF1, Fas	4	15.2967	1.42672	0.02
STAT1: c-Jun		131	14-3-3eta, 5-HT-1A, A1R, ActR-I, ActR-IB, AKT-1, angiotensinogen, APP695, AR-isoform1, ARG, AT2, ATR-isoform1, beta-arrestin2-isoform1, beta-catenin, betaARK-1, c-Cbl, c-FLIP-L, c-FLIP-S, c-Jun, calmodulin, Caspase-8, CBP, CD3zeta-isoform1, CD4, Cdc14A2, cdk9, CHIP, Csk, CtBP1, CTF-1, CTF-2, D2-isoform1, Delta40p53, DLK, DOR, Dyrk1B, Egr-1, Elk1-isoform1, ephrin-A1, EpoR, ER-alpha-L, ER-beta-isoform1, ErbB1-p170, ERK1, ERK5, FADD, FAF1, Fas, Fetua, fgfr1	4	15.2327	1.32833	0.04

Komarov et al. Functional genetics-directed identification of novel pharmacological inhibitors of FAS- and TNF-dependent apoptosis that protect mice from acute liver failure

Supplemental Table 9:

DT Network of primary screen target genes

Supplemental Table 9. DT Network of primary screen target genes

Drug Targets				
Gene Symbol	Description	Drug	Interaction Type	Database
ABCC5	ABCC5 - ATP-binding cassette, sub-family C (CFTR/MRP), member 5	FLUOROURACIL	n/a	PharmGKB
ABCC5	ABCC5 - ATP-binding cassette, sub-family C (CFTR/MRP), member 5	IRINOTECAN	n/a	PharmGKB
ABL2	ABL2 - v-abl Abelson murine leukemia viral oncogene homolog 2	DASATINIB	n/a	TEND
ABL2	ABL2 - v-abl Abelson murine leukemia viral oncogene homolog 2	5-AMINO-3-{[4-(AMINOSULFONYL)PHENYL]AMINO}-N-(2)	n/a	DrugBank
ABL2	ABL2 - v-abl Abelson murine leukemia viral oncogene homolog 2	ADENOSINE TRIPHOSPHATE	inhibitor	DrugBank
ABL2	ABL2 - v-abl Abelson murine leukemia viral oncogene homolog 2	DASATINIB	multitarget	DrugBank
ACACA	ACACA - acetyl-CoA carboxylase alpha	BIOTIN	n/a	DrugBank
ACADM	ACADM - acyl-CoA dehydrogenase, C-4 to C-12 straight chain	OCTANOYL-COENZYME A	n/a	DrugBank
ACADM	ACADM - acyl-CoA dehydrogenase, C-4 to C-12 straight chain	FLAVIN-ADENINE DINUCLEOTIDE	n/a	DrugBank
ACADM	ACADM - acyl-CoA dehydrogenase, C-4 to C-12 straight chain	3-THIAOCTANOYL-COENZYME A	n/a	DrugBank
ACTA1	ACTA1 - actin, alpha 1, skeletal muscle	JASPISAMIDE A	n/a	DrugBank
ACTA1	ACTA1 - actin, alpha 1, skeletal muscle	REIDISPONGIOLIDE C	n/a	DrugBank
ACTA1	ACTA1 - actin, alpha 1, skeletal muscle	REIDISPONGIOLIDE A	n/a	DrugBank
ACTA1	ACTA1 - actin, alpha 1, skeletal muscle	PHOSPHOAMINOPHOSPHONIC ACID-ADENYLATE ESTER	n/a	DrugBank
ACTA1	ACTA1 - actin, alpha 1, skeletal muscle	SUCROSE	n/a	DrugBank
ACTA1	ACTA1 - actin, alpha 1, skeletal muscle	LATRUNCULIN A	inhibitor	DrugBank
ACTA1	ACTA1 - actin, alpha 1, skeletal muscle	LATRUNCULIN B	n/a	DrugBank
ACTA1	ACTA1 - actin, alpha 1, skeletal muscle	4-METHYL-HISTIDINE	n/a	DrugBank
ACTA1	ACTA1 - actin, alpha 1, skeletal muscle	TMR	n/a	DrugBank
ACTA1	ACTA1 - actin, alpha 1, skeletal muscle	APLYRONINE A	n/a	DrugBank
ACTA1	ACTA1 - actin, alpha 1, skeletal muscle	SPHINXOLIDE B	n/a	DrugBank
ACTA1	ACTA1 - actin, alpha 1, skeletal muscle	KABIRAMIDE C	n/a	DrugBank
ACTA1	ACTA1 - actin, alpha 1, skeletal muscle	ADENOSINE-5'-DIPHOSPHATE	n/a	DrugBank
ACTA1	ACTA1 - actin, alpha 1, skeletal muscle	ULAPUALIDE A	n/a	DrugBank
ACVR1	ACVR1 - activin A receptor, type I	6-[4-(2-PIPERIDIN-1-YLETHOXY)PHENYL]-3-PYRIDIN-...	n/a	DrugBank
ACVR1	ACVR1 - activin A receptor, type I	ADENOSINE TRIPHOSPHATE	n/a	DrugBank
ADCY2	ADCY2 - adenylate cyclase 2 (brain)	FORSKOLIN	n/a	DrugBank
ADCY2	ADCY2 - adenylate cyclase 2 (brain)	2',5'-DIDEOXY-ADENOSINE 3'-MONOPHOSPHATE	n/a	DrugBank
ADORA1	ADORA1 - adenosine A1 receptor	THEOPHYLLINE	n/a	TEND
ADORA1	ADORA1 - adenosine A1 receptor	CAFFEINE	n/a	TEND
ADORA1	ADORA1 - adenosine A1 receptor	DYPHYLLINE	antagonist	DrugBank
ADORA1	ADORA1 - adenosine A1 receptor	CAFFEINE	multitarget/antagonist	DrugBank
ADORA1	ADORA1 - adenosine A1 receptor	AMINOPHYLLINE	antagonist	DrugBank
ADORA1	ADORA1 - adenosine A1 receptor	OXTRIPHYLLINE	antagonist	DrugBank
ADORA1	ADORA1 - adenosine A1 receptor	THEOPHYLLINE	antagonist	DrugBank
ADORA1	ADORA1 - adenosine A1 receptor	THEOBROMINE	antagonist	DrugBank
ADORA1	ADORA1 - adenosine A1 receptor	ADENOSINE	agonist	DrugBank
ADORA1	ADORA1 - adenosine A1 receptor	GABAPENTIN	agonist	DrugBank
ADORA1	ADORA1 - adenosine A1 receptor	DEFIBROTIDE	n/a	DrugBank
ADORA1	ADORA1 - adenosine A1 receptor	PENTOXIFYLLINE	antagonist	DrugBank
ADORA1	ADORA1 - adenosine A1 receptor	ROLOFYLLINE	antagonist	TTD
ADORA1	ADORA1 - adenosine A1 receptor	TECADENOSON	agonist	TTD
ADORA1	ADORA1 - adenosine A1 receptor	CAFFEINE	antagonist	TTD
ADORA1	ADORA1 - adenosine A1 receptor	MK-7418	antagonist	TTD
ADORA1	ADORA1 - adenosine A1 receptor	CVT-124	antagonist	TTD
ADORA1	ADORA1 - adenosine A1 receptor	SLV320	antagonist	TTD
ADRA2A	ADRA2A - adrenoceptor alpha 2A	DEBRISOQUIN	n/a	TEND
ADRA2A	ADRA2A - adrenoceptor alpha 2A	PHENTOLAMINE	n/a	TEND
ADRA2A	ADRA2A - adrenoceptor alpha 2A	MIANSERIN	n/a	TEND
ADRA2A	ADRA2A - adrenoceptor alpha 2A	BETHANIDINE	n/a	TEND
ADRA2A	ADRA2A - adrenoceptor alpha 2A	NOREPINEPHRINE	n/a	TEND
ADRA2A	ADRA2A - adrenoceptor alpha 2A	YOHIMBINE	n/a	TEND
ADRA2A	ADRA2A - adrenoceptor alpha 2A	ERGOLOID MESYLATE	n/a	TEND
ADRA2A	ADRA2A - adrenoceptor alpha 2A	GUANFACINE	n/a	TEND
ADRA2A	ADRA2A - adrenoceptor alpha 2A	OXYMETAZOLINE	n/a	TEND
ADRA2A	ADRA2A - adrenoceptor alpha 2A	AMPHETAMINE	n/a	TEND
ADRA2A	ADRA2A - adrenoceptor alpha 2A	FENOLDOPAM	n/a	TEND
ADRA2A	ADRA2A - adrenoceptor alpha 2A	BRIMONIDINE	n/a	TEND
ADRA2A	ADRA2A - adrenoceptor alpha 2A	APRACLONIDINE	n/a	TEND
ADRA2A	ADRA2A - adrenoceptor alpha 2A	DEXMEDETOMIDINE	n/a	TEND

Supplemental Table 9. DT Network of primary screen target genes

ADRA2A	ADRA2A - adrenoceptor alpha 2A	PSEUDOEPHEDRINE	n/a	TEND
ADRA2A	ADRA2A - adrenoceptor alpha 2A	MIRTAZAPINE	n/a	TEND
ADRA2A	ADRA2A - adrenoceptor alpha 2A	TIZANIDINE	n/a	TEND
ADRA2A	ADRA2A - adrenoceptor alpha 2A	CLONIDINE	n/a	TEND
ADRA2A	ADRA2A - adrenoceptor alpha 2A	PHENYLPROPANOLAMINE	n/a	TEND
ADRA2A	ADRA2A - adrenoceptor alpha 2A	DIPIVEFRIN	n/a	TEND
ADRA2A	ADRA2A - adrenoceptor alpha 2A	TRAZODONE	n/a	TEND
ADRA2A	ADRA2A - adrenoceptor alpha 2A	LOFEXIDINE	n/a	TEND
ADRA2A	ADRA2A - adrenoceptor alpha 2A	GUANABENZ	n/a	TEND
ADRA2A	ADRA2A - adrenoceptor alpha 2A	METHYLDOPA	n/a	TEND
ADRA2A	ADRA2A - adrenoceptor alpha 2A	BENZPHETAMINE	n/a	TEND
ADRA2A	ADRA2A - adrenoceptor alpha 2A	TOLAZOLINE	antagonist	DrugBank
ADRA2A	ADRA2A - adrenoceptor alpha 2A	CABERGOLINE	antagonist	DrugBank
ADRA2A	ADRA2A - adrenoceptor alpha 2A	FENOLDOPAM	antagonist	DrugBank
ADRA2A	ADRA2A - adrenoceptor alpha 2A	PERGOLIDE	agonist	DrugBank
ADRA2A	ADRA2A - adrenoceptor alpha 2A	PHENYLPROPANOLAMINE	agonist	DrugBank
ADRA2A	ADRA2A - adrenoceptor alpha 2A	METHYLDOPA	n/a	DrugBank
ADRA2A	ADRA2A - adrenoceptor alpha 2A	BETHANIDINE	agonist	DrugBank
ADRA2A	ADRA2A - adrenoceptor alpha 2A	4-METHOXYAMPHETAMINE	agonist	DrugBank
ADRA2A	ADRA2A - adrenoceptor alpha 2A	TRAZODONE	antagonist	DrugBank
ADRA2A	ADRA2A - adrenoceptor alpha 2A	OLANZAPINE	antagonist	DrugBank
ADRA2A	ADRA2A - adrenoceptor alpha 2A	ROPINIROLE	agonist	DrugBank
ADRA2A	ADRA2A - adrenoceptor alpha 2A	BROMOCRIPTINE	agonist	DrugBank
ADRA2A	ADRA2A - adrenoceptor alpha 2A	ARIPIPRAZOLE	antagonist	DrugBank
ADRA2A	ADRA2A - adrenoceptor alpha 2A	PHENTOLAMINE	antagonist	DrugBank
ADRA2A	ADRA2A - adrenoceptor alpha 2A	EPHEDRA	agonist	DrugBank
ADRA2A	ADRA2A - adrenoceptor alpha 2A	TIZANIDINE	agonist	DrugBank
ADRA2A	ADRA2A - adrenoceptor alpha 2A	EPINEPHRINE	agonist	DrugBank
ADRA2A	ADRA2A - adrenoceptor alpha 2A	GUANABENZ	agonist	DrugBank
ADRA2A	ADRA2A - adrenoceptor alpha 2A	LOFEXIDINE	agonist	DrugBank
ADRA2A	ADRA2A - adrenoceptor alpha 2A	METHOTRIMEPRAZINE	antagonist	DrugBank
ADRA2A	ADRA2A - adrenoceptor alpha 2A	APRACLONIDINE	agonist	DrugBank
ADRA2A	ADRA2A - adrenoceptor alpha 2A	NOREPINEPHRINE	agonist	DrugBank
ADRA2A	ADRA2A - adrenoceptor alpha 2A	DROXIDOPA	agonist	DrugBank
ADRA2A	ADRA2A - adrenoceptor alpha 2A	PRAMIPEXOLE	partial agonist	DrugBank
ADRA2A	ADRA2A - adrenoceptor alpha 2A	MIRTAZAPINE	antagonist	DrugBank
ADRA2A	ADRA2A - adrenoceptor alpha 2A	GUANFACINE	agonist	DrugBank
ADRA2A	ADRA2A - adrenoceptor alpha 2A	AMOXAPINE	antagonist	DrugBank
ADRA2A	ADRA2A - adrenoceptor alpha 2A	DIHYDROERGOTAMINE	agonist	DrugBank
ADRA2A	ADRA2A - adrenoceptor alpha 2A	CLOZAPINE	antagonist	DrugBank
ADRA2A	ADRA2A - adrenoceptor alpha 2A	DOXEPIN	antagonist	DrugBank
ADRA2A	ADRA2A - adrenoceptor alpha 2A	PROPERICIAZINE	antagonist	DrugBank
ADRA2A	ADRA2A - adrenoceptor alpha 2A	MIANSERIN	antagonist	DrugBank
ADRA2A	ADRA2A - adrenoceptor alpha 2A	AMITRIPTYLINE	antagonist	DrugBank
ADRA2A	ADRA2A - adrenoceptor alpha 2A	NEFAZODONE	antagonist	DrugBank
ADRA2A	ADRA2A - adrenoceptor alpha 2A	BRIMONIDINE	agonist	DrugBank
ADRA2A	ADRA2A - adrenoceptor alpha 2A	DIHYDROERGOTOXINE	antagonist	DrugBank
ADRA2A	ADRA2A - adrenoceptor alpha 2A	YOHIMBINE	antagonist	DrugBank
ADRA2A	ADRA2A - adrenoceptor alpha 2A	DEXMEDETOMIDINE	agonist	DrugBank
ADRA2A	ADRA2A - adrenoceptor alpha 2A	PHENOXYBENZAMINE	antagonist	DrugBank
ADRA2A	ADRA2A - adrenoceptor alpha 2A	PALIPERIDONE	antagonist	DrugBank
ADRA2A	ADRA2A - adrenoceptor alpha 2A	RISPERIDONE	antagonist	DrugBank
ADRA2A	ADRA2A - adrenoceptor alpha 2A	ERGOTAMINE	partial agonist	DrugBank
ADRA2A	ADRA2A - adrenoceptor alpha 2A	NAPHAZOLINE	agonist	DrugBank
ADRA2A	ADRA2A - adrenoceptor alpha 2A	EPINASTINE	other/unknown	DrugBank
ADRA2A	ADRA2A - adrenoceptor alpha 2A	PSEUDOEPHEDRINE	agonist	DrugBank
ADRA2A	ADRA2A - adrenoceptor alpha 2A	BENZPHETAMINE	agonist	DrugBank
ADRA2A	ADRA2A - adrenoceptor alpha 2A	DIPIVEFRIN	agonist	DrugBank
ADRA2A	ADRA2A - adrenoceptor alpha 2A	ZIPRASIDONE	antagonist	DrugBank
ADRA2A	ADRA2A - adrenoceptor alpha 2A	OXYMETAZOLINE	agonist	DrugBank
ADRA2A	ADRA2A - adrenoceptor alpha 2A	XYLOMETAZOLINE	agonist	DrugBank
ADRA2A	ADRA2A - adrenoceptor alpha 2A	LISURIDE	other/unknown	DrugBank
ADRA2A	ADRA2A - adrenoceptor alpha 2A	ERGOLOID MESYLATE	antagonist	DrugBank
ADRA2A	ADRA2A - adrenoceptor alpha 2A	QUETIAPINE	antagonist	DrugBank
ADRA2A	ADRA2A - adrenoceptor alpha 2A	METHAMPHETAMINE	agonist	DrugBank
ADRA2A	ADRA2A - adrenoceptor alpha 2A	APOMORPHINE	agonist	DrugBank
ADRA2A	ADRA2A - adrenoceptor alpha 2A	CLONIDINE	agonist	DrugBank
ADRA2A	ADRA2A - adrenoceptor alpha 2A	ZUCLOPENTHIXOL	antagonist	DrugBank
ADRA2A	ADRA2A - adrenoceptor alpha 2A	CONNEXYN	agonist	TTD
ADRA2A	ADRA2A - adrenoceptor alpha 2A	BRIMONIDINE	agonist	TTD
ADRA2A	ADRA2A - adrenoceptor alpha 2A	DEXMEDETOMIDINE	n/a	PharmGKB
ADRB1	ADRB1 - adrenoceptor beta 1	DESIPRAMINE	n/a	TEND
ADRB1	ADRB1 - adrenoceptor beta 1	PENBUTOLOL	n/a	TEND
ADRB1	ADRB1 - adrenoceptor beta 1	ALPRENOLOL	n/a	TEND
ADRB1	ADRB1 - adrenoceptor beta 1	ISOPROTERENOL	n/a	TEND
ADRB1	ADRB1 - adrenoceptor beta 1	ISOETHARINE	n/a	TEND
ADRB1	ADRB1 - adrenoceptor beta 1	ZIPRASIDONE	n/a	TEND
ADRB1	ADRB1 - adrenoceptor beta 1	CARVEDILOL	n/a	TEND
ADRB1	ADRB1 - adrenoceptor beta 1	PROPRANOLOL	n/a	TEND

Supplemental Table 9. DT Network of primary screen target genes

ADRB1	ADRB1 - adrenoceptor beta 1	LABETALOL	n/a	TEND
ADRB1	ADRB1 - adrenoceptor beta 1	EPINEPHRINE	n/a	TEND
ADRB1	ADRB1 - adrenoceptor beta 1	CARTEOLOL	n/a	TEND
ADRB1	ADRB1 - adrenoceptor beta 1	LEVOBUNOLOL	n/a	TEND
ADRB1	ADRB1 - adrenoceptor beta 1	PRACTOLOL	n/a	TEND
ADRB1	ADRB1 - adrenoceptor beta 1	NADOLOL	n/a	TEND
ADRB1	ADRB1 - adrenoceptor beta 1	DRONEDARONE	n/a	TEND
ADRB1	ADRB1 - adrenoceptor beta 1	PINDOLOL	n/a	TEND
ADRB1	ADRB1 - adrenoceptor beta 1	OXPRENOLOL	n/a	TEND
ADRB1	ADRB1 - adrenoceptor beta 1	ACEBUTOLOL	n/a	TEND
ADRB1	ADRB1 - adrenoceptor beta 1	AMIODARONE	n/a	TEND
ADRB1	ADRB1 - adrenoceptor beta 1	NEBIVOLOL	n/a	TEND
ADRB1	ADRB1 - adrenoceptor beta 1	BEVANTOLOL	n/a	TEND
ADRB1	ADRB1 - adrenoceptor beta 1	METIPRANOLOL	n/a	TEND
ADRB1	ADRB1 - adrenoceptor beta 1	BETAXOLOL	n/a	TEND
ADRB1	ADRB1 - adrenoceptor beta 1	TIMOLOL	n/a	TEND
ADRB1	ADRB1 - adrenoceptor beta 1	BISOPROLOL	n/a	TEND
ADRB1	ADRB1 - adrenoceptor beta 1	ESMOLOL	n/a	TEND
ADRB1	ADRB1 - adrenoceptor beta 1	DOBUTAMINE	n/a	TEND
ADRB1	ADRB1 - adrenoceptor beta 1	ARBUTAMINE	n/a	TEND
ADRB1	ADRB1 - adrenoceptor beta 1	METOPROLOL	n/a	TEND
ADRB1	ADRB1 - adrenoceptor beta 1	ATENOLOL	n/a	TEND
ADRB1	ADRB1 - adrenoceptor beta 1	EPHEDRA	agonist	DrugBank
ADRB1	ADRB1 - adrenoceptor beta 1	PINDOLOL	partial agonist	DrugBank
ADRB1	ADRB1 - adrenoceptor beta 1	DOBUTAMINE	agonist	DrugBank
ADRB1	ADRB1 - adrenoceptor beta 1	LABETALOL	antagonist	DrugBank
ADRB1	ADRB1 - adrenoceptor beta 1	ATENOLOL	antagonist	DrugBank
ADRB1	ADRB1 - adrenoceptor beta 1	METIPRANOLOL	antagonist	DrugBank
ADRB1	ADRB1 - adrenoceptor beta 1	ISOPROTERENOL	agonist	DrugBank
ADRB1	ADRB1 - adrenoceptor beta 1	CARTEOLOL	partial agonist	DrugBank
ADRB1	ADRB1 - adrenoceptor beta 1	DROXIDOPA	agonist	DrugBank
ADRB1	ADRB1 - adrenoceptor beta 1	SOTALOL	antagonist	DrugBank
ADRB1	ADRB1 - adrenoceptor beta 1	ARBUTAMINE	agonist	DrugBank
ADRB1	ADRB1 - adrenoceptor beta 1	BUPRANOLOL	antagonist	DrugBank
ADRB1	ADRB1 - adrenoceptor beta 1	ALPRENOLOL	antagonist	DrugBank
ADRB1	ADRB1 - adrenoceptor beta 1	DESIPRAMINE	other/unknown	DrugBank
ADRB1	ADRB1 - adrenoceptor beta 1	LEVOBUNOLOL	antagonist	DrugBank
ADRB1	ADRB1 - adrenoceptor beta 1	TIMOLOL	antagonist	DrugBank
ADRB1	ADRB1 - adrenoceptor beta 1	BEVANTOLOL	antagonist	DrugBank
ADRB1	ADRB1 - adrenoceptor beta 1	EPINEPHRINE	agonist	DrugBank
ADRB1	ADRB1 - adrenoceptor beta 1	METOPROLOL	antagonist	DrugBank
ADRB1	ADRB1 - adrenoceptor beta 1	4-{[(2S)-3-(TERT-BUTYLAMINO)-2-HYDROXYPROPYL]OX...	n/a	DrugBank
ADRB1	ADRB1 - adrenoceptor beta 1	PROPRANOLOL	antagonist	DrugBank
ADRB1	ADRB1 - adrenoceptor beta 1	ACEBUTOLOL	partial agonist	DrugBank
ADRB1	ADRB1 - adrenoceptor beta 1	OXPRENOLOL	antagonist	DrugBank
ADRB1	ADRB1 - adrenoceptor beta 1	PIRBUTEROL	agonist	DrugBank
ADRB1	ADRB1 - adrenoceptor beta 1	ESMOLOL	antagonist	DrugBank
ADRB1	ADRB1 - adrenoceptor beta 1	AMIODARONE	antagonist	DrugBank
ADRB1	ADRB1 - adrenoceptor beta 1	BISOPROLOL	antagonist	DrugBank
ADRB1	ADRB1 - adrenoceptor beta 1	FENOTEROL	agonist	DrugBank
ADRB1	ADRB1 - adrenoceptor beta 1	ISOETHARINE	agonist	DrugBank
ADRB1	ADRB1 - adrenoceptor beta 1	PENBUTOLOL	antagonist	DrugBank
ADRB1	ADRB1 - adrenoceptor beta 1	CARVEDILOL	antagonist	DrugBank
ADRB1	ADRB1 - adrenoceptor beta 1	BOPINDOLOL	partial agonist	DrugBank
ADRB1	ADRB1 - adrenoceptor beta 1	SALBUTAMOL	agonist	DrugBank
ADRB1	ADRB1 - adrenoceptor beta 1	CLENBUTEROL	agonist	DrugBank
ADRB1	ADRB1 - adrenoceptor beta 1	PHENYLPROPANOLAMINE	agonist	DrugBank
ADRB1	ADRB1 - adrenoceptor beta 1	NOREPINEPHRINE	agonist	DrugBank
ADRB1	ADRB1 - adrenoceptor beta 1	2-HYDROXYMETHYL-6-OCTYLSULFANYL-TETRAHYDRO-PYRA...	n/a	DrugBank
ADRB1	ADRB1 - adrenoceptor beta 1	BETAXOLOL	antagonist	DrugBank
ADRB1	ADRB1 - adrenoceptor beta 1	NEBIVOLOL	antagonist	DrugBank
ADRB1	ADRB1 - adrenoceptor beta 1	PSEUDOEPHEDRINE	partial agonist/agonist	DrugBank
ADRB1	ADRB1 - adrenoceptor beta 1	PRACTOLOL	antagonist	DrugBank
ADRB1	ADRB1 - adrenoceptor beta 1	NADOLOL	antagonist	DrugBank
ADRB1	ADRB1 - adrenoceptor beta 1	SOTALOL	antagonist	TTD
ADRB1	ADRB1 - adrenoceptor beta 1	PRACTOLOL	antagonist	TTD
ADRB1	ADRB1 - adrenoceptor beta 1	DOBUTAMINE	agonist	TTD
ADRB1	ADRB1 - adrenoceptor beta 1	ATENOLOL	antagonist	TTD
ADRB1	ADRB1 - adrenoceptor beta 1	BETAXOLOL	antagonist	TTD
ADRB1	ADRB1 - adrenoceptor beta 1	ALPRENOLOL	antagonist	TTD
ADRB1	ADRB1 - adrenoceptor beta 1	METOPROLOL	antagonist	TTD
ADRB1	ADRB1 - adrenoceptor beta 1	OXPRENOLOL	antagonist	TTD
ADRB1	ADRB1 - adrenoceptor beta 1	NEBIVOLOL	antagonist	TTD
ADRB1	ADRB1 - adrenoceptor beta 1	ACEBUTOLOL	antagonist	TTD
ADRB1	ADRB1 - adrenoceptor beta 1	ESMOLOL	antagonist	TTD

Supplemental Table 9. DT Network of primary screen target genes

ADRB1	ADRB1 - adrenoceptor beta 1	BISOPROLOL	antagonist	TTD
ADRB1	ADRB1 - adrenoceptor beta 1	LEVOBETAXOLOL	antagonist	TTD
ADRB1	ADRB1 - adrenoceptor beta 1	ISOPROTERENOL	n/a	PharmGKB
ADRB1	ADRB1 - adrenoceptor beta 1	EPINEPHRINE	n/a	PharmGKB
ADRB1	ADRB1 - adrenoceptor beta 1	XENOBIOTICS	n/a	PharmGKB
ADRB1	ADRB1 - adrenoceptor beta 1	NOREPINEPHRINE	n/a	PharmGKB
ADRB1	ADRB1 - adrenoceptor beta 1	DOBUTAMINE	n/a	PharmGKB
ADRB1	ADRB1 - adrenoceptor beta 1	ACEBUTOLOL	n/a	PharmGKB
ADRB1	ADRB1 - adrenoceptor beta 1	DOPAMINE	n/a	PharmGKB
ADRB1	ADRB1 - adrenoceptor beta 1	MURAGLITAZAR	n/a	PharmGKB
ADRB1	ADRB1 - adrenoceptor beta 1	SELECTIVE BETA-2-ADRENORECEPTOR AGONISTS	n/a	PharmGKB
ADRB1	ADRB1 - adrenoceptor beta 1	ANTIDEPRESSANTS	n/a	PharmGKB
ADRB1	ADRB1 - adrenoceptor beta 1	BUCINDOLOL	n/a	PharmGKB
ADRB3	ADRB3 - adrenoceptor beta 3	NOREPINEPHRINE	agonist	DrugBank
ADRB3	ADRB3 - adrenoceptor beta 3	BOPINDOLOL	n/a	DrugBank
ADRB3	ADRB3 - adrenoceptor beta 3	EPHEDRA	agonist	DrugBank
ADRB3	ADRB3 - adrenoceptor beta 3	BUPRANOLOL	antagonist	DrugBank
ADRB3	ADRB3 - adrenoceptor beta 3	CLENBUTEROL	agonist	DrugBank
ADRB3	ADRB3 - adrenoceptor beta 3	PROPRANOLOL	antagonist	DrugBank
ADRB3	ADRB3 - adrenoceptor beta 3	ISOPROTERENOL	agonist	DrugBank
ADRB3	ADRB3 - adrenoceptor beta 3	ARBUTAMINE	agonist	DrugBank
ADRB3	ADRB3 - adrenoceptor beta 3	FENOTEROL	agonist	DrugBank
ADRB3	ADRB3 - adrenoceptor beta 3	DROXIDOPA	agonist	DrugBank
ADRB3	ADRB3 - adrenoceptor beta 3	AZ-40140	agonist	TTD
ADRB3	ADRB3 - adrenoceptor beta 3	GCR-1087	agonist	TTD
ADRB3	ADRB3 - adrenoceptor beta 3	GW-427353	agonist	TTD
ADRB3	ADRB3 - adrenoceptor beta 3	L-742791	agonist	TTD
ADRB3	ADRB3 - adrenoceptor beta 3	N-5984	agonist	TTD
ADRB3	ADRB3 - adrenoceptor beta 3	AMIBEGRON	agonist	TTD
ADRB3	ADRB3 - adrenoceptor beta 3	BETA3-ADRENOCEPTOR AGONIST	agonist	TTD
ADRB3	ADRB3 - adrenoceptor beta 3	LY-362884	agonist	TTD
ADRB3	ADRB3 - adrenoceptor beta 3	L-751250	agonist	TTD
ADRB3	ADRB3 - adrenoceptor beta 3	CP-114271	agonist	TTD
ADRB3	ADRB3 - adrenoceptor beta 3	CL-314698	agonist	TTD
ADRB3	ADRB3 - adrenoceptor beta 3	THIAZIDES, PLAIN	n/a	PharmGKB
ADRB3	ADRB3 - adrenoceptor beta 3	OLANZAPINE	n/a	PharmGKB
AGXT	AGXT - alanine-glyoxylate aminotransferase	GLYCINE	n/a	DrugBank
AGXT	AGXT - alanine-glyoxylate aminotransferase	4-(2-AMINOPHENYL)-4-OXOBUTANOIC ACID	n/a	DrugBank
AGXT	AGXT - alanine-glyoxylate aminotransferase	L-SERINE	n/a	DrugBank
AGXT	AGXT - alanine-glyoxylate aminotransferase	PYRIDOXAL PHOSPHATE	cofactor	DrugBank
AGXT	AGXT - alanine-glyoxylate aminotransferase	N'-PYRIDOXYL-LYSINE-5'-MONOPHOSPHATE	n/a	DrugBank
AGXT	AGXT - alanine-glyoxylate aminotransferase	(AMINOOXY)ACETIC ACID	n/a	DrugBank
AGXT	AGXT - alanine-glyoxylate aminotransferase	L-ALANINE	n/a	DrugBank
AGXT	AGXT - alanine-glyoxylate aminotransferase	PYRIDOXAL PHOSPHATE	cofactor	DrugBank
ALAD	ALAD - aminolevulinate dehydratase	AMINOLEVULINIC ACID	inducer	DrugBank
ALAD	ALAD - aminolevulinate dehydratase	LAEVULINIC ACID	n/a	DrugBank
ALAD	ALAD - aminolevulinate dehydratase	4-OXOSEBACIC ACID	n/a	DrugBank
ALAD	ALAD - aminolevulinate dehydratase	3-(2-AMINOETHYL)-4-(AMINOMETHYL)HEPTANEDIOIC ACID	n/a	DrugBank
ALAD	ALAD - aminolevulinate dehydratase	DELTA-AMINO VALERIC ACID	n/a	DrugBank
ALAD	ALAD - aminolevulinate dehydratase	PORPHOBILINOGEN	n/a	DrugBank
ALAD	ALAD - aminolevulinate dehydratase	5-HYDROXYVALERIC ACID	n/a	DrugBank
ALAD	ALAD - aminolevulinate dehydratase	4,7-DIOXOSEBACIC ACID	n/a	DrugBank
ALAD	ALAD - aminolevulinate dehydratase	AMINOLEVULINIC ACID	inhibitor	TTD
ALPPL2	ALPPL2 - alkaline phosphatase, placental-like 2	AMIFOSTINE	n/a	TEND
ALPPL2	ALPPL2 - alkaline phosphatase, placental-like 2	AMIFOSTINE	inducer	DrugBank
ALPPL2	ALPPL2 - alkaline phosphatase, placental-like 2	LEVAMISOLE	inhibitor	DrugBank
AMY1A	AMY1A - amylase, alpha 1A (salivary)	5-HYDROXYMETHYL-CHONDURITOL	n/a	DrugBank
AMY1A	AMY1A - amylase, alpha 1A (salivary)	4,6-DIDEOXY-4-AMINO-ALPHA-D-GLUCOSE	n/a	DrugBank
AMY1A	AMY1A - amylase, alpha 1A (salivary)	TRIS(HYDROXYETHYL)AMINOMETHANE	n/a	DrugBank
AMY1A	AMY1A - amylase, alpha 1A (salivary)	BETA-D-GLUCOSE	n/a	DrugBank
AMY1A	AMY1A - amylase, alpha 1A (salivary)	PYROGLUTAMIC ACID	n/a	DrugBank
APCS	APCS - amyloid P component, serum	METHYL 4,6-O-[(1R)-1-CARBOXYETHYLIDENE]-BETA-D-...	n/a	DrugBank

Supplemental Table 9. DT Network of primary screen target genes

APCS	APCS - amyloid P component, serum	BIS-1,2-{[(Z)-2-CARBOXY-2-METHYL-1,3-DIOXANE]-5...	n/a	DrugBank
APCS	APCS - amyloid P component, serum	BIS-1,2-{[(Z)-2CARBOXY-2-METHYL-1,3-DIOXANE]-5-...	n/a	DrugBank
APOA1	APOA1 - apolipoprotein A-I	FENOFIBRATE	n/a	PharmGKB
APOA1	APOA1 - apolipoprotein A-I	RITONAVIR	n/a	PharmGKB
APOA1	APOA1 - apolipoprotein A-I	PROTEASE INHIBITORS	n/a	PharmGKB
AQP5	AQP5 - aquaporin 5	THIAZOLIDINEDIONES	n/a	PharmGKB
ATR	ATR - ataxia telangiectasia and Rad3 related	E7449	n/a	ClearityFoundationBiomarkers
ATR	ATR - ataxia telangiectasia and Rad3 related	BMN673	n/a	ClearityFoundationBiomarkers
ATR	ATR - ataxia telangiectasia and Rad3 related	RUCAPARIB	n/a	ClearityFoundationBiomarkers
ATR	ATR - ataxia telangiectasia and Rad3 related	VELIPARIB	n/a	ClearityFoundationBiomarkers
ATR	ATR - ataxia telangiectasia and Rad3 related	NIRAPARIB	n/a	ClearityFoundationBiomarkers
ATR	ATR - ataxia telangiectasia and Rad3 related	OLAPARIB	n/a	ClearityFoundationBiomarkers
AZGP1	AZGP1 - alpha-2-glycoprotein 1, zinc-binding	ALPHA-D-MANNOSE	n/a	DrugBank
AZGP1	AZGP1 - alpha-2-glycoprotein 1, zinc-binding	O-SIALIC ACID	n/a	DrugBank
BCHE	BCHE - butyrylcholinesterase	ECHOTHIOPHATE IODIDE	n/a	TEND
BCHE	BCHE - butyrylcholinesterase	RIVASTIGMINE	n/a	TEND
BCHE	BCHE - butyrylcholinesterase	HEXAFLURONIUM BROMIDE	n/a	TEND
BCHE	BCHE - butyrylcholinesterase	ISOFLUROPHATE	n/a	TEND
BCHE	BCHE - butyrylcholinesterase	DEMECARIUM BROMIDE	n/a	TEND
BCHE	BCHE - butyrylcholinesterase	TACRINE	inhibitor	DrugBank
BCHE	BCHE - butyrylcholinesterase	METHYLPHOSPHINIC ACID	n/a	DrugBank
BCHE	BCHE - butyrylcholinesterase	9-N-PHENYLMETHYLAMINO-TACRINE	n/a	DrugBank
BCHE	BCHE - butyrylcholinesterase	PYRIDOSTIGMINE	antagonist	DrugBank
BCHE	BCHE - butyrylcholinesterase	PRALIDOXIME	activator	DrugBank
BCHE	BCHE - butyrylcholinesterase	ETHYL DIHYDROGEN PHOSPHATE	n/a	DrugBank
BCHE	BCHE - butyrylcholinesterase	(1S)-MENTHYL HEXYL PHOSPHONATE GROUP	n/a	DrugBank
BCHE	BCHE - butyrylcholinesterase	MALATHION	inhibitor	DrugBank
BCHE	BCHE - butyrylcholinesterase	DEMECARIUM	inhibitor	DrugBank
BCHE	BCHE - butyrylcholinesterase	DODECANESULFONATE ION	n/a	DrugBank
BCHE	BCHE - butyrylcholinesterase	2-(N-MORPHOLINO)-ETHANESULFONIC ACID	n/a	DrugBank
BCHE	BCHE - butyrylcholinesterase	MONOISOPROPYL ESTER PHOSPHONIC ACID GROUP	n/a	DrugBank
BCHE	BCHE - butyrylcholinesterase	RIVASTIGMINE	inhibitor	DrugBank
BCHE	BCHE - butyrylcholinesterase	CHOLINE	product of	DrugBank
BCHE	BCHE - butyrylcholinesterase	DIPIVEFRIN	potentiator	DrugBank
BCHE	BCHE - butyrylcholinesterase	ALPHA-D-MANNOSE	n/a	DrugBank
BCHE	BCHE - butyrylcholinesterase	EDROPHONIUM	inhibitor	DrugBank
BCHE	BCHE - butyrylcholinesterase	9-(3-IODOBENZYLAMINO)-1,2,3,4-TETRAHYDROACRIDINE	n/a	DrugBank
BCHE	BCHE - butyrylcholinesterase	ISOFLUROPHATE	inhibitor	DrugBank
BCHE	BCHE - butyrylcholinesterase	BETA-D-MANNOSE	n/a	DrugBank
BCHE	BCHE - butyrylcholinesterase	DIETHYLPHOSPHONO GROUP	n/a	DrugBank
BCHE	BCHE - butyrylcholinesterase	BUTANOIC ACID	n/a	DrugBank
BCHE	BCHE - butyrylcholinesterase	MIVACURIUM	n/a	DrugBank
BCHE	BCHE - butyrylcholinesterase	BUTYRYLTHIOCHOLINE	n/a	DrugBank
BCHE	BCHE - butyrylcholinesterase	HEXAFLURONIUM	inhibitor	DrugBank
BCHE	BCHE - butyrylcholinesterase	GALANTAMINE	inhibitor	DrugBank
BCHE	BCHE - butyrylcholinesterase	ECHOTHIOPHATE	inhibitor	DrugBank
BCHE	BCHE - butyrylcholinesterase	(1R)-MENTHYL HEXYL PHOSPHONATE GROUP	n/a	DrugBank
BCHE	BCHE - butyrylcholinesterase	ETHYL HYDROGEN DIETHYLAMIDOPHOSPHATE	n/a	DrugBank
BCHE	BCHE - butyrylcholinesterase	HEXAFLURONIUM BROMIDE	inhibitor	TTD
BCHE	BCHE - butyrylcholinesterase	ECHOTHIOPHATE IODIDE	inhibitor	TTD
BCHE	BCHE - butyrylcholinesterase	SUCCINYLCHOLINE	n/a	PharmGKB
BHMT	BHMT - betaine--homocysteine S-methyltransferase	CITRIC ACID	n/a	DrugBank
BHMT	BHMT - betaine--homocysteine S-methyltransferase	L-METHIONINE	product of	DrugBank
BHMT	BHMT - betaine--homocysteine S-methyltransferase	S-(D-CARBOXYBUTYL)-L-HOMOCYSTEINE	n/a	DrugBank
BHMT2	BHMT2 - betaine--homocysteine S-methyltransferase 2	L-METHIONINE	product of	DrugBank
C3	C3 - complement component 3	S-HYDROXYCYSTEINE	n/a	DrugBank
C3	C3 - complement component 3	INTRAVENOUS IMMUNOGLOBULIN	binder	DrugBank
CA13	CA13 - carbonic anhydrase XIII	ZONISAMIDE	inhibitor	DrugBank

Supplemental Table 9. DT Network of primary screen target genes

CA13	CA13 - carbonic anhydrase XIII	N-(4-CHLOROBENZYL)-N-METHYLBENZENE-1,4-DISULFON...	n/a	DrugBank
CA14	CA14 - carbonic anhydrase XIV	ZONISAMIDE	inhibitor	DrugBank
CA14	CA14 - carbonic anhydrase XIV	ACETAZOLAMIDE	inhibitor	DrugBank
CA14	CA14 - carbonic anhydrase XIV	BETA-D-MANNOSE	n/a	DrugBank
CACNA2D4	CACNA2D4 - calcium channel, voltage-dependent, alpha 2/delta subunit 4	CELECOXIB	n/a	PharmGKB
CASR	CASR - calcium-sensing receptor	CINACALCET	n/a	TEND
CASR	CASR - calcium-sensing receptor	CINACALCET	agonist	DrugBank
CASR	CASR - calcium-sensing receptor	RONACALERET	antagonist	TTD
CASR	CASR - calcium-sensing receptor	CINACALCET	agonist	TTD
CCKBR	CCKBR - cholecystokinin B receptor	PENTAGASTRIN	n/a	TEND
CCKBR	CCKBR - cholecystokinin B receptor	PENTAGASTRIN	agonist	DrugBank
CCKBR	CCKBR - cholecystokinin B receptor	GG-8573	antagonist	TTD
CCKBR	CCKBR - cholecystokinin B receptor	PENTAGASTRIN	agonist	TTD
CCKBR	CCKBR - cholecystokinin B receptor	CERULETIDE	agonist	TTD
CCKBR	CCKBR - cholecystokinin B receptor	PD-170292	agonist	TTD
CCR2	CCR2 - chemokine (C-C motif) receptor 2	MCP-1	antagonist	TTD
CCR2	CCR2 - chemokine (C-C motif) receptor 2	INCB3284	antagonist	TTD
CCR2	CCR2 - chemokine (C-C motif) receptor 2	INCB-3284	antagonist	TTD
CCR2	CCR2 - chemokine (C-C motif) receptor 2	MLN1202	antagonist	TTD
CCR2	CCR2 - chemokine (C-C motif) receptor 2	RS-504393	antagonist	TTD
CCR2	CCR2 - chemokine (C-C motif) receptor 2	INCB8696	antagonist	TTD
CCR2	CCR2 - chemokine (C-C motif) receptor 2	CCX915	antagonist	TTD
CCR4	CCR4 - chemokine (C-C motif) receptor 4	AMG 761	antibody	TTD
CCR4	CCR4 - chemokine (C-C motif) receptor 4	AT008	antibody	TTD
CCR4	CCR4 - chemokine (C-C motif) receptor 4	MOGAMULIZUMAB	antibody	MyCancerGenome
CD4	CD4 - CD4 molecule	ANTITHYMOCYTE GLOBULIN	n/a	DrugBank
CD4	CD4 - CD4 molecule	TNX-355	binder	TTD
CD4	CD4 - CD4 molecule	ANTI-CD4	antibody	TTD
CDK9	CDK9 - cyclin-dependent kinase 9	FLAVOPIRIDOL	n/a	DrugBank
CDK9	CDK9 - cyclin-dependent kinase 9	SNS-032	inhibitor	TTD
CDK9	CDK9 - cyclin-dependent kinase 9	P276-00	inhibitor	TTD
CDK9	CDK9 - cyclin-dependent kinase 9	SCH 727965	inhibitor	TTD
CDK9	CDK9 - cyclin-dependent kinase 9	ZK 304709	inhibitor	TTD
CDK9	CDK9 - cyclin-dependent kinase 9	METHOTREXATE	n/a	PharmGKB
CDK9	CDK9 - cyclin-dependent kinase 9	ALVOCIDIB	inhibitor	MyCancerGenome
CDK9	CDK9 - cyclin-dependent kinase 9	SELICICLIB	inhibitor	MyCancerGenome
CDK9	CDK9 - cyclin-dependent kinase 9	DINACICLIB	inhibitor	MyCancerGenome
CDK9	CDK9 - cyclin-dependent kinase 9	DINACICLIB	n/a	ClearityFoundationClinicalTrial
CDK9	CDK9 - cyclin-dependent kinase 9	DINACICLIB	n/a	ClearityFoundationClinicalTrial
CDK9	CDK9 - cyclin-dependent kinase 9	N/A	n/a	ClearityFoundationClinicalTrial
CDK9	CDK9 - cyclin-dependent kinase 9	SNS-032	inhibitor	CancerCommons
CDK9	CDK9 - cyclin-dependent kinase 9	BAY1000394	inhibitor	CancerCommons
CDK9	CDK9 - cyclin-dependent kinase 9	SCH727965	inhibitor	CancerCommons
CDKN1A	CDKN1A - cyclin-dependent kinase inhibitor 1A (p21, Cip1)	CELECOXIB	n/a	PharmGKB
CHRNA3	CHRNA3 - cholinergic receptor, nicotinic, alpha 3 (neuronal)	GALANTAMINE	allosteric modulator	DrugBank
CHRNA3	CHRNA3 - cholinergic receptor, nicotinic, alpha 3 (neuronal)	VARENICLINE	partial agonist	DrugBank
CHRNA3	CHRNA3 - cholinergic receptor, nicotinic, alpha 3 (neuronal)	LEVOMETHADYL ACETATE	antagonist	DrugBank
CHRNA3	CHRNA3 - cholinergic receptor, nicotinic, alpha 3 (neuronal)	PENTOLINIUM	antagonist	DrugBank
CHRNA3	CHRNA3 - cholinergic receptor, nicotinic, alpha 3 (neuronal)	LEVAMISOLE	agonist	DrugBank
CHRNA3	CHRNA3 - cholinergic receptor, nicotinic, alpha 3 (neuronal)	NICOTINE	agonist	DrugBank
CHRNA3	CHRNA3 - cholinergic receptor, nicotinic, alpha 3 (neuronal)	BUPROPION	antagonist	DrugBank
CKMT2	CKMT2 - creatine kinase, mitochondrial 2 (sarcomeric)	CREATINE	ligand	DrugBank
CLCN2	CLCN2 - chloride channel, voltage-sensitive 2	LUBIPROSTONE	n/a	TEND

Supplemental Table 9. DT Network of primary screen target genes

CLCN2	CLCN2 - chloride channel, voltage-sensitive 2	LUBIPROSTONE	inducer	DrugBank
CLCN2	CLCN2 - chloride channel, voltage-sensitive 2	LUBIPROSTONE	activator	TTD
CNTF	CNTF - ciliary neurotrophic factor	ILOPERIDONE	n/a	PharmGKB
CRTC1	CRTC1 - CREB regulated transcription coactivator 1	XL765	n/a	ClearityFoundationClinicalTrial
CRTC1	CRTC1 - CREB regulated transcription coactivator 1	N/A	n/a	ClearityFoundationClinicalTrial
CRTC1	CRTC1 - CREB regulated transcription coactivator 1	N/A	n/a	ClearityFoundationClinicalTrial
CRTC1	CRTC1 - CREB regulated transcription coactivator 1	N/A	n/a	ClearityFoundationClinicalTrial
CRTC1	CRTC1 - CREB regulated transcription coactivator 1	NVP-BEZ235	n/a	ClearityFoundationClinicalTrial
CRTC1	CRTC1 - CREB regulated transcription coactivator 1	NVP-BEZ235	n/a	ClearityFoundationClinicalTrial
CRTC1	CRTC1 - CREB regulated transcription coactivator 1	PKI-587	n/a	ClearityFoundationClinicalTrial
CRTC1	CRTC1 - CREB regulated transcription coactivator 1	NVP-BEZ235	n/a	ClearityFoundationClinicalTrial
CSK	CSK - c-src tyrosine kinase	STAUROSPORINE	n/a	DrugBank
CSNK1G1	CSNK1G1 - casein kinase 1, gamma 1	2-(2-HYDROXYETHYLAMINO)-6-(3-CHLOROANILINO)-9-I...	n/a	DrugBank
CSNK1G2	CSNK1G2 - casein kinase 1, gamma 2	5-IODOTUBERCIDIN	n/a	DrugBank
CSNK1G2	CSNK1G2 - casein kinase 1, gamma 2	IC261	n/a	DrugBank
CSNK1G2	CSNK1G2 - casein kinase 1, gamma 2	N-(2-AMINOETHYL)-5-CHLOROISOQUINOLINE-8-SULFONA...	n/a	DrugBank
CTBP1	CTBP1 - C-terminal binding protein 1	FORMIC ACID	n/a	DrugBank
CTBP1	CTBP1 - C-terminal binding protein 1	NICOTINAMIDE-ADENINE-DINUCLEOTIDE	n/a	DrugBank
CTSB	CTSB - cathepsin B	N-{[(2S,3S)-3-(ETHOXYCARBONYL)OXIRAN-2-YL]CARBO...	n/a	DrugBank
CTSB	CTSB - cathepsin B	2-AMINOETHANIMIDIC ACID	n/a	DrugBank
CTSB	CTSB - cathepsin B	METHYL N-({(2S,3S)-3-[(PROPYLAMINO)CARBONYL]OXI...	n/a	DrugBank
CTSB	CTSB - cathepsin B	3-AMINO-4-OXYBENZYL-2-BUTANONE	n/a	DrugBank
CTSB	CTSB - cathepsin B	DIPHENYLACETIC ACID	n/a	DrugBank
CTSB	CTSB - cathepsin B	N-{[(2S,3S)-3-(ETHOXYCARBONYL)OXIRAN-2-YL]CARBO...	n/a	DrugBank
CTSB	CTSB - cathepsin B	BENZYL N-({(2S,3S)-3-[(PROPYLAMINO)CARBONYL]OXI...	n/a	DrugBank
CTSB	CTSB - cathepsin B	N-{[(2S,3S)-3-(ETHOXYCARBONYL)OXIRAN-2-YL]CARBO...	n/a	DrugBank
CTSB	CTSB - cathepsin B	N-({(2S,3S)-3-[(BENZYLAMINO)CARBONYL]OXIRAN-2-Y...	n/a	DrugBank
CTSB	CTSB - cathepsin B	N-{[(2S,3S)-3-(ETHOXYCARBONYL)OXIRAN-2-YL]CARBO...	n/a	DrugBank
CTSB	CTSB - cathepsin B	N-(3-PROPYLCARBAMOYLOXIRANE-2-CARBONYL)-ISOLEUC...	n/a	DrugBank
CTSB	CTSB - cathepsin B	N-[1-HYDROXYCARBOXYETHYL-CARBONYL]LEUCYLAMINO-2...	n/a	DrugBank
CTSB	CTSB - cathepsin B	3-METHYLPHENYLALANINE	n/a	DrugBank
CTSB	CTSB - cathepsin B	2-PYRIDINETHIOL	n/a	DrugBank
CXCL12	CXCL12 - chemokine (C-X-C motif) ligand 12	TINZAPARIN	binder	DrugBank
CYP2C9	CYP2C9 - cytochrome P450, family 2, subfamily C, polypeptide 9	4-HYDROXY-3-[(1S)-3-OXO-1-PHENYLBUTYL]-2H-CHROM...	n/a	DrugBank
CYP2C9	CYP2C9 - cytochrome P450, family 2, subfamily C, polypeptide 9	HEME C	n/a	DrugBank
CYP2C9	CYP2C9 - cytochrome P450, family 2, subfamily C, polypeptide 9	HEME	n/a	DrugBank
CYP2C9	CYP2C9 - cytochrome P450, family 2, subfamily C, polypeptide 9	PIROXICAM	n/a	PharmGKB
CYP2C9	CYP2C9 - cytochrome P450, family 2, subfamily C, polypeptide 9	DIAZEPAM	n/a	PharmGKB
CYP2C9	CYP2C9 - cytochrome P450, family 2, subfamily C, polypeptide 9	ANTIEPILEPTICS	n/a	PharmGKB
CYP2C9	CYP2C9 - cytochrome P450, family 2, subfamily C, polypeptide 9	LOSARTAN	n/a	PharmGKB
CYP2C9	CYP2C9 - cytochrome P450, family 2, subfamily C, polypeptide 9	THEOPHYLLINE	n/a	PharmGKB

Supplemental Table 9. DT Network of primary screen target genes

CYP2C9	CYP2C9 - cytochrome P450, family 2, subfamily C, polypeptide 9	ANTIINFLAMMATORY AGENTS, NON-STEROIDS	n/a	PharmGKB
CYP2C9	CYP2C9 - cytochrome P450, family 2, subfamily C, polypeptide 9	IRBESARTAN	n/a	PharmGKB
CYP2C9	CYP2C9 - cytochrome P450, family 2, subfamily C, polypeptide 9	FLUVASTATIN	n/a	PharmGKB
CYP2C9	CYP2C9 - cytochrome P450, family 2, subfamily C, polypeptide 9	GLIPIZIDE	n/a	PharmGKB
CYP2C9	CYP2C9 - cytochrome P450, family 2, subfamily C, polypeptide 9	NAPROXEN	n/a	PharmGKB
CYP2C9	CYP2C9 - cytochrome P450, family 2, subfamily C, polypeptide 9	MELOXICAM	n/a	PharmGKB
CYP2C9	CYP2C9 - cytochrome P450, family 2, subfamily C, polypeptide 9	CYCLOPHOSPHAMIDE	n/a	PharmGKB
CYP2C9	CYP2C9 - cytochrome P450, family 2, subfamily C, polypeptide 9	CAFFEINE	n/a	PharmGKB
CYP2C9	CYP2C9 - cytochrome P450, family 2, subfamily C, polypeptide 9	FLUVOXAMINE	n/a	PharmGKB
CYP2C9	CYP2C9 - cytochrome P450, family 2, subfamily C, polypeptide 9	IBUPROFEN	n/a	PharmGKB
CYP2C9	CYP2C9 - cytochrome P450, family 2, subfamily C, polypeptide 9	SULFONAMIDES, UREA DERIVATIVES	n/a	PharmGKB
CYP2C9	CYP2C9 - cytochrome P450, family 2, subfamily C, polypeptide 9	TAMOXIFEN	n/a	PharmGKB
CYP2C9	CYP2C9 - cytochrome P450, family 2, subfamily C, polypeptide 9	FLURBIPROFEN	n/a	PharmGKB
CYP2C9	CYP2C9 - cytochrome P450, family 2, subfamily C, polypeptide 9	DICLOFENAC	n/a	PharmGKB
CYP7A1	CYP7A1 - cytochrome P450, family 7, subfamily A, polypeptide 1	ATORVASTATIN	n/a	PharmGKB
CYSLTR1	CYSLTR1 - cysteinyl leukotriene receptor 1	MONTELUKAST	n/a	TEND
CYSLTR1	CYSLTR1 - cysteinyl leukotriene receptor 1	PRANLUKAST	n/a	TEND
CYSLTR1	CYSLTR1 - cysteinyl leukotriene receptor 1	NEDOCROMIL	n/a	TEND
CYSLTR1	CYSLTR1 - cysteinyl leukotriene receptor 1	ZAFIRLUKAST	n/a	TEND
CYSLTR1	CYSLTR1 - cysteinyl leukotriene receptor 1	CINALUKAST	n/a	TEND
CYSLTR1	CYSLTR1 - cysteinyl leukotriene receptor 1	NEDOCROMIL	suppressor	DrugBank
CYSLTR1	CYSLTR1 - cysteinyl leukotriene receptor 1	ZAFIRLUKAST	antagonist	DrugBank
CYSLTR1	CYSLTR1 - cysteinyl leukotriene receptor 1	PRANLUKAST	antagonist	DrugBank
CYSLTR1	CYSLTR1 - cysteinyl leukotriene receptor 1	CINALUKAST	antagonist	DrugBank
CYSLTR1	CYSLTR1 - cysteinyl leukotriene receptor 1	MONTELUKAST	antagonist	DrugBank
CYSLTR1	CYSLTR1 - cysteinyl leukotriene receptor 1	ZAFIRLUKAST	antagonist	TTD
CYSLTR1	CYSLTR1 - cysteinyl leukotriene receptor 1	MONTELUKAST	antagonist	TTD
CYSLTR1	CYSLTR1 - cysteinyl leukotriene receptor 1	CINALUKAST	antagonist	TTD
CYSLTR1	CYSLTR1 - cysteinyl leukotriene receptor 1	PRANLUKAST	antagonist	TTD
DCI	ECI1 - enoyl-CoA delta isomerase 1	BENZAMIDINE	n/a	DrugBank
DCI	ECI1 - enoyl-CoA delta isomerase 1	OCTANOYL-COENZYME A	n/a	DrugBank
DHFR	DHFR - dihydrofolate reductase	PRALATREXATE	n/a	TEND
DHFR	DHFR - dihydrofolate reductase	PEMETREXED	n/a	TEND
DHFR	DHFR - dihydrofolate reductase	TRIMETREXATE	n/a	TEND
DHFR	DHFR - dihydrofolate reductase	2,4-DIAMINO-5-METHYL-6-[(3,4,5-TRIMETHOXY-N-MET...	n/a	DrugBank
DHFR	DHFR - dihydrofolate reductase	2,4-DIAMINO-5-(3,4,5-TRIMETHOXY-BENZYL)-PYRIMID...	n/a	DrugBank
DHFR	DHFR - dihydrofolate reductase	[N-(2,4-DIAMINOPTERIDIN-6-YL)-METHYL]-DIBENZ[B,...	n/a	DrugBank
DHFR	DHFR - dihydrofolate reductase	(4AS)-5-[(2,4-DIAMINOPTERIDIN-6-YL)METHYL]-4A,5...	n/a	DrugBank
DHFR	DHFR - dihydrofolate reductase	BIOPTERIN	n/a	DrugBank
DHFR	DHFR - dihydrofolate reductase	5-[(3R)-3-(5-METHOXYBIPHENYL-3-YL)BUT-1-YN-1-YL...	n/a	DrugBank
DHFR	DHFR - dihydrofolate reductase	2,4-DIAMINO-6-[N-(3',5'-DIMETHOXYBENZYL)-N-METH...	n/a	DrugBank
DHFR	DHFR - dihydrofolate reductase	5-[(3R)-3-(5-METHOXY-4'-METHYLBIPHENYL-3-YL)BUT...	n/a	DrugBank

Supplemental Table 9. DT Network of primary screen target genes

DHFR	DHFR - dihydrofolate reductase	6-(2,5-DIMETHOXY-BENZYL)-5-METHYL-PYRIDO[2,3-D]...	n/a	DrugBank
DHFR	DHFR - dihydrofolate reductase	PYRIMETHAMINE	inhibitor	DrugBank
DHFR	DHFR - dihydrofolate reductase	SRI-9662	n/a	DrugBank
DHFR	DHFR - dihydrofolate reductase	2,4-DIAMINO-6-[N-(2',5'-DIMETHOXYBENZYL)-N-METH...	n/a	DrugBank
DHFR	DHFR - dihydrofolate reductase	PEMETREXED	inhibitor	DrugBank
DHFR	DHFR - dihydrofolate reductase	2,4-DIAMINO-6-[N-(3',4',5'-TRIMETHOXYBENZYL)-N-...	n/a	DrugBank
DHFR	DHFR - dihydrofolate reductase	5-[(3R)-3-(5-METHOXY-3',5'-DIMETHYLBIPHENYL-3-Y...	n/a	DrugBank
DHFR	DHFR - dihydrofolate reductase	TRIMETREXATE	inhibitor	DrugBank
DHFR	DHFR - dihydrofolate reductase	NADH	n/a	DrugBank
DHFR	DHFR - dihydrofolate reductase	(2R,6S)-6-{[METHYL(3,4,5-TRIMETHOXYPHENYL)AMINO...	n/a	DrugBank
DHFR	DHFR - dihydrofolate reductase	2'-MONOPHOSPHOADENOSINE 5'-DIPHOSPHORIBOSE	n/a	DrugBank
DHFR	DHFR - dihydrofolate reductase	PROGUANIL	inhibitor	DrugBank
DHFR	DHFR - dihydrofolate reductase	5-[3-(2,5-DIMETHOXYPHENYL)PROP-1-YN-1-YL]-6-ETH...	n/a	DrugBank
DHFR	DHFR - dihydrofolate reductase	N6-(2,5-DIMETHOXY-BENZYL)-N6-METHYL-PYRIDO[2,3-...	n/a	DrugBank
DHFR	DHFR - dihydrofolate reductase	TRIMETHOPRIM	inhibitor	DrugBank
DHFR	DHFR - dihydrofolate reductase	SRI-9439	n/a	DrugBank
DHFR	DHFR - dihydrofolate reductase	5-[(3R)-3-(5-METHOXY-2',6'-DIMETHYLBIPHENYL-3-Y...	n/a	DrugBank
DHFR	DHFR - dihydrofolate reductase	6-(OCTAHYDRO-1H-INDOL-1-YLMETHYL)DECAHYDROQUINA...	n/a	DrugBank
DHFR	DHFR - dihydrofolate reductase	METHOTREXATE	inhibitor	DrugBank
DHFR	DHFR - dihydrofolate reductase	MULTIVITAMINS, PLAIN	n/a	PharmGKB
DHFR	DHFR - dihydrofolate reductase	PEMETREXED	n/a	ClearityFoundationClinicalTrial
DHFR	DHFR - dihydrofolate reductase	PEMETREXED	n/a	ClearityFoundationClinicalTrial
DHFR	DHFR - dihydrofolate reductase	PEMETREXED	n/a	ClearityFoundationClinicalTrial
DHFR	DHFR - dihydrofolate reductase	PRALATREXATE	n/a	ClearityFoundationClinicalTrial
DRD2	DRD2 - dopamine receptor D2	ZUCLOPENTHIXOL	n/a	TEND
DRD2	DRD2 - dopamine receptor D2	THIETHYLPERAZINE	n/a	TEND
DRD2	DRD2 - dopamine receptor D2	FLUPENTHIXOL	n/a	TEND
DRD2	DRD2 - dopamine receptor D2	RISPERIDONE	n/a	TEND
DRD2	DRD2 - dopamine receptor D2	MESORIDAZINE	n/a	TEND
DRD2	DRD2 - dopamine receptor D2	TRIFLUPROMAZINE	n/a	TEND
DRD2	DRD2 - dopamine receptor D2	FLUPHENAZINE	n/a	TEND
DRD2	DRD2 - dopamine receptor D2	SERTINDOLE	n/a	TEND
DRD2	DRD2 - dopamine receptor D2	PALIPERIDONE	n/a	TEND
DRD2	DRD2 - dopamine receptor D2	BUSPIRONE	n/a	TEND
DRD2	DRD2 - dopamine receptor D2	METOCLOPRAMIDE	n/a	TEND
DRD2	DRD2 - dopamine receptor D2	ROTIGOTINE	n/a	TEND
DRD2	DRD2 - dopamine receptor D2	ASENAPINE	n/a	TEND
DRD2	DRD2 - dopamine receptor D2	PROMAZINE	n/a	TEND
DRD2	DRD2 - dopamine receptor D2	LISURIDE	n/a	TEND
DRD2	DRD2 - dopamine receptor D2	AMISULPRIDE	n/a	TEND
DRD2	DRD2 - dopamine receptor D2	CLOZAPINE	n/a	TEND
DRD2	DRD2 - dopamine receptor D2	QUETIAPINE	n/a	TEND
DRD2	DRD2 - dopamine receptor D2	MOLINDONE	n/a	TEND
DRD2	DRD2 - dopamine receptor D2	PERPHENAZINE	n/a	TEND
DRD2	DRD2 - dopamine receptor D2	DOMPERIDONE	n/a	TEND
DRD2	DRD2 - dopamine receptor D2	ROPINIROLE	n/a	TEND
DRD2	DRD2 - dopamine receptor D2	LOXAPINE	n/a	TEND
DRD2	DRD2 - dopamine receptor D2	ILOPERIDONE	n/a	TEND
DRD2	DRD2 - dopamine receptor D2	LEVODOPA	n/a	TEND
DRD2	DRD2 - dopamine receptor D2	DROPERIDOL	n/a	TEND
DRD2	DRD2 - dopamine receptor D2	OLANZAPINE	n/a	TEND
DRD2	DRD2 - dopamine receptor D2	THIORIDAZINE	n/a	TEND
DRD2	DRD2 - dopamine receptor D2	PRAMIPEXOLE	n/a	TEND
DRD2	DRD2 - dopamine receptor D2	REMOXIPRIDE	n/a	TEND
DRD2	DRD2 - dopamine receptor D2	HALOPERIDOL	n/a	TEND
DRD2	DRD2 - dopamine receptor D2	SULPIRIDE	n/a	TEND
DRD2	DRD2 - dopamine receptor D2	ACETOPHENAZINE	n/a	TEND
DRD2	DRD2 - dopamine receptor D2	CHLORPROTHIXENE	n/a	TEND
DRD2	DRD2 - dopamine receptor D2	LURASIDONE	n/a	TEND
DRD2	DRD2 - dopamine receptor D2	BROMOCRIPTINE	n/a	TEND
DRD2	DRD2 - dopamine receptor D2	CARPHENAZINE	n/a	TEND
DRD2	DRD2 - dopamine receptor D2	ARIPIPRAZOLE	n/a	TEND
DRD2	DRD2 - dopamine receptor D2	PERGOLIDE	n/a	TEND
DRD2	DRD2 - dopamine receptor D2	TRIFLUOPERAZINE	n/a	TEND
DRD2	DRD2 - dopamine receptor D2	MINAPRINE	n/a	TEND

Supplemental Table 9. DT Network of primary screen target genes

DRD2	DRD2 - dopamine receptor D2	APOMORPHINE	n/a	TEND
DRD2	DRD2 - dopamine receptor D2	FLUSPIRILENE	n/a	TEND
DRD2	DRD2 - dopamine receptor D2	PIMOZIDE	n/a	TEND
DRD2	DRD2 - dopamine receptor D2	CABERGOLINE	n/a	TEND
DRD2	DRD2 - dopamine receptor D2	PROCHLORPERAZINE	n/a	TEND
DRD2	DRD2 - dopamine receptor D2	ZIPRASIDONE	n/a	TEND
DRD2	DRD2 - dopamine receptor D2	PROPIOMAZINE	n/a	TEND
DRD2	DRD2 - dopamine receptor D2	ZUCLOPENTHIXOL	antagonist	DrugBank
DRD2	DRD2 - dopamine receptor D2	MOLINDONE	antagonist	DrugBank
DRD2	DRD2 - dopamine receptor D2	PROPIOMAZINE	antagonist	DrugBank
DRD2	DRD2 - dopamine receptor D2	ROTIGOTINE	agonist	DrugBank
DRD2	DRD2 - dopamine receptor D2	METHOTRIMEPRAZINE	antagonist	DrugBank
DRD2	DRD2 - dopamine receptor D2	CABERGOLINE	agonist	DrugBank
DRD2	DRD2 - dopamine receptor D2	DOMPERIDONE	antagonist	DrugBank
DRD2	DRD2 - dopamine receptor D2	BROMOCRIPTINE	agonist	DrugBank
DRD2	DRD2 - dopamine receptor D2	AMANTADINE	agonist	DrugBank
DRD2	DRD2 - dopamine receptor D2	TRIMIPRAMINE	other/unknown	DrugBank
DRD2	DRD2 - dopamine receptor D2	HALOPERIDOL	antagonist	DrugBank
DRD2	DRD2 - dopamine receptor D2	YOHIMBINE	antagonist	DrugBank
DRD2	DRD2 - dopamine receptor D2	DOPAMINE	agonist	DrugBank
DRD2	DRD2 - dopamine receptor D2	AMOXAPINE	antagonist	DrugBank
DRD2	DRD2 - dopamine receptor D2	THIORIDAZINE	antagonist	DrugBank
DRD2	DRD2 - dopamine receptor D2	PERPHENAZINE	antagonist	DrugBank
DRD2	DRD2 - dopamine receptor D2	ACETOPHENAZINE	antagonist	DrugBank
DRD2	DRD2 - dopamine receptor D2	OLANZAPINE	antagonist/agonist	DrugBank
DRD2	DRD2 - dopamine receptor D2	LOXAPINE	antagonist	DrugBank
DRD2	DRD2 - dopamine receptor D2	ACEPROMAZINE	antagonist	DrugBank
DRD2	DRD2 - dopamine receptor D2	REMOXIPRIDE	antagonist	DrugBank
DRD2	DRD2 - dopamine receptor D2	ROLICYCLIDINE	n/a	DrugBank
DRD2	DRD2 - dopamine receptor D2	CLOZAPINE	antagonist	DrugBank
DRD2	DRD2 - dopamine receptor D2	FLUSPIRILENE	antagonist	DrugBank
DRD2	DRD2 - dopamine receptor D2	SULPIRIDE	antagonist	DrugBank
DRD2	DRD2 - dopamine receptor D2	CHLORPROTHIXENE	antagonist	DrugBank
DRD2	DRD2 - dopamine receptor D2	PERGOLIDE	agonist	DrugBank
DRD2	DRD2 - dopamine receptor D2	PRAMIPEXOLE	agonist	DrugBank
DRD2	DRD2 - dopamine receptor D2	ERGOTAMINE	agonist	DrugBank
DRD2	DRD2 - dopamine receptor D2	ALIZAPRIDE	antagonist	DrugBank
DRD2	DRD2 - dopamine receptor D2	FLUPHENAZINE	antagonist	DrugBank
DRD2	DRD2 - dopamine receptor D2	APOMORPHINE	agonist	DrugBank
DRD2	DRD2 - dopamine receptor D2	KETAMINE	partial agonist/agonist	DrugBank
DRD2	DRD2 - dopamine receptor D2	THIOPROPERAZINE	antagonist	DrugBank
DRD2	DRD2 - dopamine receptor D2	LEVODOPA	agonist	DrugBank
DRD2	DRD2 - dopamine receptor D2	LISURIDE	agonist	DrugBank
DRD2	DRD2 - dopamine receptor D2	MESORIDAZINE	antagonist	DrugBank
DRD2	DRD2 - dopamine receptor D2	PROMAZINE	antagonist	DrugBank
DRD2	DRD2 - dopamine receptor D2	TRIFLUOPERAZINE	antagonist	DrugBank
DRD2	DRD2 - dopamine receptor D2	DOXEPIN	antagonist	DrugBank
DRD2	DRD2 - dopamine receptor D2	CARPHENAZINE	antagonist	DrugBank
DRD2	DRD2 - dopamine receptor D2	ZIPRASIDONE	antagonist	DrugBank
DRD2	DRD2 - dopamine receptor D2	BUSPIRONE	antagonist	DrugBank
DRD2	DRD2 - dopamine receptor D2	PIPOTIAZINE	antagonist	DrugBank
DRD2	DRD2 - dopamine receptor D2	DROPERIDOL	antagonist	DrugBank
DRD2	DRD2 - dopamine receptor D2	AMISULPRIDE	antagonist	DrugBank
DRD2	DRD2 - dopamine receptor D2	ROPINIROLE	agonist	DrugBank
DRD2	DRD2 - dopamine receptor D2	THIETHYLPERAZINE	antagonist	DrugBank
DRD2	DRD2 - dopamine receptor D2	THIOTHIXENE	antagonist	DrugBank
DRD2	DRD2 - dopamine receptor D2	PROMETHAZINE	antagonist	DrugBank
DRD2	DRD2 - dopamine receptor D2	METOCLOPRAMIDE	antagonist	DrugBank
DRD2	DRD2 - dopamine receptor D2	TRIFLUPROMAZINE	antagonist	DrugBank
DRD2	DRD2 - dopamine receptor D2	MINAPRINE	agonist	DrugBank
DRD2	DRD2 - dopamine receptor D2	QUETIAPINE	antagonist	DrugBank
DRD2	DRD2 - dopamine receptor D2	FLUPENTHIXOL	antagonist	DrugBank
DRD2	DRD2 - dopamine receptor D2	PIMOZIDE	antagonist	DrugBank
DRD2	DRD2 - dopamine receptor D2	SERTINDOLE	antagonist	DrugBank
DRD2	DRD2 - dopamine receptor D2	CHLORPROMAZINE	antagonist	DrugBank
DRD2	DRD2 - dopamine receptor D2	ANIRACETAM	n/a	DrugBank
DRD2	DRD2 - dopamine receptor D2	RISPERIDONE	antagonist	DrugBank
DRD2	DRD2 - dopamine receptor D2	CINNARIZINE	other/unknown	DrugBank
DRD2	DRD2 - dopamine receptor D2	ARIPIPRAZOLE	antagonist/partial agonist	DrugBank
DRD2	DRD2 - dopamine receptor D2	PROCHLORPERAZINE	antagonist	DrugBank
DRD2	DRD2 - dopamine receptor D2	PALIPERIDONE	antagonist	DrugBank
DRD2	DRD2 - dopamine receptor D2	CHLORPROMAZINE	antagonist	TTD
DRD2	DRD2 - dopamine receptor D2	BROMOCRIPTINE	agonist	TTD
DRD2	DRD2 - dopamine receptor D2	DOMPERIDONE MALEATE	antagonist	TTD
DRD2	DRD2 - dopamine receptor D2	S32504	agonist	TTD
DRD2	DRD2 - dopamine receptor D2	ACETOPHENAZINE	antagonist	TTD
DRD2	DRD2 - dopamine receptor D2	Y-931	binder	TTD

Supplemental Table 9. DT Network of primary screen target genes

DRD2	DRD2 - dopamine receptor D2	FLUSPIRILENE	antagonist	TTD
DRD2	DRD2 - dopamine receptor D2	QUETIAPINE SR	antagonist	TTD
DRD2	DRD2 - dopamine receptor D2	DEXAMFETAMINE	agonist	TTD
DRD2	DRD2 - dopamine receptor D2	BIFEPRUNOX	agonist	TTD
DRD2	DRD2 - dopamine receptor D2	DOPAMINE	agonist	TTD
DRD2	DRD2 - dopamine receptor D2	NOMIFENSINE	antagonist	TTD
DRD2	DRD2 - dopamine receptor D2	DIHYDROERGOTOXINE	agonist	TTD
DRD2	DRD2 - dopamine receptor D2	QUETIAPINE	agonist	TTD
DRD2	DRD2 - dopamine receptor D2	REMOXIPRIDE	antagonist	TTD
DRD2	DRD2 - dopamine receptor D2	PD-158771	antagonist	TTD
DRD2	DRD2 - dopamine receptor D2	ASENAPINE	antagonist	TTD
DRD2	DRD2 - dopamine receptor D2	SLV-310	antagonist	TTD
DRD2	DRD2 - dopamine receptor D2	FLUPENTHIXOL	antagonist	TTD
DRD2	DRD2 - dopamine receptor D2	CHLORPROTHIXENE	antagonist	TTD
DRD2	DRD2 - dopamine receptor D2	METHAMFETAMINE	agonist	TTD
DRD2	DRD2 - dopamine receptor D2	HALOPERIDOL	antagonist	TTD
DRD2	DRD2 - dopamine receptor D2	CABERGOLINE	agonist	TTD
DRD2	DRD2 - dopamine receptor D2	SLV-313	agonist	TTD
DRD2	DRD2 - dopamine receptor D2	NEMONAPRIDE	agonist	TTD
DRD2	DRD2 - dopamine receptor D2	ROPINIROLE	agonist	TTD
DRD2	DRD2 - dopamine receptor D2	DOPAMINE ANTAGS	binder	TTD
DRD2	DRD2 - dopamine receptor D2	SUMANIROLE	agonist	TTD
DRD2	DRD2 - dopamine receptor D2	PALIPERIDONE	antagonist	TTD
DRD2	DRD2 - dopamine receptor D2	ZUCLOPENTHIXOL	antagonist	TTD
DRD2	DRD2 - dopamine receptor D2	PIMOZIDE	antagonist	TTD
DRD2	DRD2 - dopamine receptor D2	OLANZAPINE	agonist	TTD
DRD2	DRD2 - dopamine receptor D2	BLONANSERIN	antagonist	TTD
DRD2	DRD2 - dopamine receptor D2	LAMECTACIN	antagonist	TTD
DRD2	DRD2 - dopamine receptor D2	D1 AGONIST D2 ANTAGONIST	antagonist	TTD
DRD2	DRD2 - dopamine receptor D2	SERTINDOLE	antagonist	TTD
DRD2	DRD2 - dopamine receptor D2	ZIPRASIDONE	agonist	TTD
DRD2	DRD2 - dopamine receptor D2	APLINDORE FUMARATE	agonist	TTD
DRD2	DRD2 - dopamine receptor D2	PD-157695	antagonist	TTD
DRD2	DRD2 - dopamine receptor D2	OCAPERIDONE	antagonist	TTD
DRD2	DRD2 - dopamine receptor D2	LOXAPINE	antagonist	TTD
DRD2	DRD2 - dopamine receptor D2	N-0923	agonist	TTD
DRD2	DRD2 - dopamine receptor D2	SIBENADET	agonist	TTD
DRD2	DRD2 - dopamine receptor D2	1192U90	antagonist	TTD
DRD2	DRD2 - dopamine receptor D2	PROCHLORPERAZINE	antagonist	TTD
DRD2	DRD2 - dopamine receptor D2	PERPHENAZINE	antagonist	TTD
DRD2	DRD2 - dopamine receptor D2	ILOPERIDONE	antagonist	TTD
DRD2	DRD2 - dopamine receptor D2	ZD-3638	binder	TTD
DRD2	DRD2 - dopamine receptor D2	(S)-AMISULPRIDE	antagonist	TTD
DRD2	DRD2 - dopamine receptor D2	QUINAGOLIDE	agonist	TTD
DRD2	DRD2 - dopamine receptor D2	SSR-181507	binder	TTD
DRD2	DRD2 - dopamine receptor D2	FLUPHENAZINE	antagonist	TTD
DRD2	DRD2 - dopamine receptor D2	AMISULPRIDE	antagonist	TTD
DRD2	DRD2 - dopamine receptor D2	ORG-23366	antagonist	TTD
DRD2	DRD2 - dopamine receptor D2	PERGOLIDE	agonist	TTD
DRD2	DRD2 - dopamine receptor D2	CARPHENAZINE	antagonist	TTD
DRD2	DRD2 - dopamine receptor D2	(-)-3PPP, MARYLAND	agonist	TTD
DRD2	DRD2 - dopamine receptor D2	GMC-283	antagonist	TTD
DRD2	DRD2 - dopamine receptor D2	DAPOTUM D	antagonist	TTD
DRD2	DRD2 - dopamine receptor D2	RISPERIDONE	antagonist	TTD
DRD2	DRD2 - dopamine receptor D2	QUINAGOLIDE HYDROCHLORIDE	agonist	TTD
DRD2	DRD2 - dopamine receptor D2	LURASIDONE HYDROCHLORIDE	antagonist	TTD
DRD2	DRD2 - dopamine receptor D2	MESORIDAZINE	agonist	TTD
DRD2	DRD2 - dopamine receptor D2	ARIPIPRAZOLE	agonist	TTD
DRD2	DRD2 - dopamine receptor D2	CLOZAPINE	antagonist	TTD
DRD2	DRD2 - dopamine receptor D2	SDZ-MAR-327	agonist	TTD
DRD2	DRD2 - dopamine receptor D2	ABAPERIDONE HYDROCHLORIDE	antagonist	TTD
DRD2	DRD2 - dopamine receptor D2	PD-172760	antagonist	TTD
DRD2	DRD2 - dopamine receptor D2	SDZ-HDC-912	binder	TTD
DRD2	DRD2 - dopamine receptor D2	PARDOPRUNOX	agonist	TTD
DRD2	DRD2 - dopamine receptor D2	SULPIRIDE	antagonist	TTD
DRD2	DRD2 - dopamine receptor D2	METOCLOPRAMIDE	antagonist	TTD
DRD2	DRD2 - dopamine receptor D2	HMR-2934	antagonist	TTD
DRD2	DRD2 - dopamine receptor D2	PRAMIPEXOLE	agonist	TTD
DRD2	DRD2 - dopamine receptor D2	MOLINDONE	binder	TTD
DRD2	DRD2 - dopamine receptor D2	ROTIGOTINE PATCH	agonist	TTD
DRD2	DRD2 - dopamine receptor D2	RISPERIDONE CONSTA	antagonist	TTD
DRD2	DRD2 - dopamine receptor D2	SLV-319	antagonist	TTD
DRD2	DRD2 - dopamine receptor D2	OLANZAPINE	n/a	PharmGKB
DRD2	DRD2 - dopamine receptor D2	CAFFEINE	n/a	PharmGKB
DRD2	DRD2 - dopamine receptor D2	CHLORPROMAZINE	n/a	PharmGKB
DRD2	DRD2 - dopamine receptor D2	NEMONAPRIDE	n/a	PharmGKB
DRD2	DRD2 - dopamine receptor D2	NICOTINE	n/a	PharmGKB
DRD2	DRD2 - dopamine receptor D2	BROMPERIDOL	n/a	PharmGKB
DRD2	DRD2 - dopamine receptor D2	BUPROPION	n/a	PharmGKB

Supplemental Table 9. DT Network of primary screen target genes

EGFR	EGFR - epidermal growth factor receptor	GEFITINIB	n/a	TEND
EGFR	EGFR - epidermal growth factor receptor	LAPATINIB	n/a	TEND
EGFR	EGFR - epidermal growth factor receptor	PANITUMUMAB	n/a	TEND
EGFR	EGFR - epidermal growth factor receptor	ERLOTINIB	n/a	TEND
EGFR	EGFR - epidermal growth factor receptor	TRASTUZUMAB	n/a	TEND
EGFR	EGFR - epidermal growth factor receptor	CETUXIMAB	n/a	TEND
EGFR	EGFR - epidermal growth factor receptor	GEFITINIB	antagonist	DrugBank
EGFR	EGFR - epidermal growth factor receptor	ERLOTINIB	antagonist	DrugBank
EGFR	EGFR - epidermal growth factor receptor	PANITUMUMAB	suppressor	DrugBank
EGFR	EGFR - epidermal growth factor receptor	FLAVOPIRIDOL	n/a	DrugBank
EGFR	EGFR - epidermal growth factor receptor	TRASTUZUMAB	n/a	DrugBank
EGFR	EGFR - epidermal growth factor receptor	LIDOCAINE	antagonist	DrugBank
EGFR	EGFR - epidermal growth factor receptor	LAPATINIB	antagonist	DrugBank
EGFR	EGFR - epidermal growth factor receptor	N-[4-(3-BROMO-PHENYLAMINO)-QUINAZOLIN-6-YL]-ACR...	n/a	DrugBank
EGFR	EGFR - epidermal growth factor receptor	CETUXIMAB	antagonist	DrugBank
EGFR	EGFR - epidermal growth factor receptor	S-{3-[(4-ANILINOQUINAZOLIN-6-YL)AMINO]-3-OXOPRO...	n/a	DrugBank
EGFR	EGFR - epidermal growth factor receptor	TYVERB/TYKERB	inhibitor	TTD
EGFR	EGFR - epidermal growth factor receptor	GEFITINIB	inhibitor	TTD
EGFR	EGFR - epidermal growth factor receptor	CETUXIMAB	antibody	TTD
EGFR	EGFR - epidermal growth factor receptor	HKI-272	inhibitor	TTD
EGFR	EGFR - epidermal growth factor receptor	BIBW 2992	inhibitor	TTD
EGFR	EGFR - epidermal growth factor receptor	S-222611	inhibitor	TTD
EGFR	EGFR - epidermal growth factor receptor	TAK165	inhibitor	TTD
EGFR	EGFR - epidermal growth factor receptor	TYKERB	inhibitor	TTD
EGFR	EGFR - epidermal growth factor receptor	PF-299804	inhibitor	TTD
EGFR	EGFR - epidermal growth factor receptor	PANITUMUMAB	n/a	TTD
EGFR	EGFR - epidermal growth factor receptor	PAZOPANIB TYVERB/TYKERB	inhibitor	TTD
EGFR	EGFR - epidermal growth factor receptor	DWP-401	activator	TTD
EGFR	EGFR - epidermal growth factor receptor	CI-1033	inhibitor	TTD
EGFR	EGFR - epidermal growth factor receptor	VANDETANIB	inhibitor	TTD
EGFR	EGFR - epidermal growth factor receptor	AZD4769	inhibitor	TTD
EGFR	EGFR - epidermal growth factor receptor	ANTI-HER3/EGFR DAF	antibody	TTD
EGFR	EGFR - epidermal growth factor receptor	HEGF	activator	TTD
EGFR	EGFR - epidermal growth factor receptor	ERLOTINIB BEVACIZUMAB	inhibitor	TTD
EGFR	EGFR - epidermal growth factor receptor	LAPATINIB	inhibitor	TTD
EGFR	EGFR - epidermal growth factor receptor	BMS-599626	inhibitor	TTD
EGFR	EGFR - epidermal growth factor receptor	ERLOTINIB	inhibitor	TTD
EGFR	EGFR - epidermal growth factor receptor	BEVACIZUMAB ERLOTINIB	inhibitor	TTD
EGFR	EGFR - epidermal growth factor receptor	XL647	inhibitor	TTD

Supplemental Table 9. DT Network of primary screen target genes

EGFR	EGFR - epidermal growth factor receptor	GEFITINIB	n/a	PharmGKB
EGFR	EGFR - epidermal growth factor receptor	ERLOTINIB	n/a	PharmGKB
EGFR	EGFR - epidermal growth factor receptor	ALKYLATING AGENTS	n/a	PharmGKB
EGFR	EGFR - epidermal growth factor receptor	TOPOISOMERASE I INHIBITORS	n/a	PharmGKB
EGFR	EGFR - epidermal growth factor receptor	GELDANAMYCIN	n/a	PharmGKB
EGFR	EGFR - epidermal growth factor receptor	ANTINEOPLASTIC AGENTS	n/a	PharmGKB
EGFR	EGFR - epidermal growth factor receptor	EGFR INHIBITORS	n/a	PharmGKB
EGFR	EGFR - epidermal growth factor receptor	NIMOTUZUMAB	antibody	MyCancerGenome
EGFR	EGFR - epidermal growth factor receptor	XL647	inhibitor	MyCancerGenome
EGFR	EGFR - epidermal growth factor receptor	GEFITINIB	inhibitor	MyCancerGenome
EGFR	EGFR - epidermal growth factor receptor	BMS-690514	inhibitor	MyCancerGenome
EGFR	EGFR - epidermal growth factor receptor	PANITUMUMAB	antibody	MyCancerGenome
EGFR	EGFR - epidermal growth factor receptor	MEHD7945A	antibody	MyCancerGenome
EGFR	EGFR - epidermal growth factor receptor	AEE788	inhibitor	MyCancerGenome
EGFR	EGFR - epidermal growth factor receptor	VANDETANIB	inhibitor	MyCancerGenome
EGFR	EGFR - epidermal growth factor receptor	AFATINIB	inhibitor	MyCancerGenome
EGFR	EGFR - epidermal growth factor receptor	CANERTINIB	inhibitor	MyCancerGenome
EGFR	EGFR - epidermal growth factor receptor	SYM004	antibody	MyCancerGenome
EGFR	EGFR - epidermal growth factor receptor	MM-151	antibody	MyCancerGenome
EGFR	EGFR - epidermal growth factor receptor	CETUXIMAB	antibody	MyCancerGenome
EGFR	EGFR - epidermal growth factor receptor	CO-1686	inhibitor	MyCancerGenome
EGFR	EGFR - epidermal growth factor receptor	ICOTINIB	inhibitor	MyCancerGenome
EGFR	EGFR - epidermal growth factor receptor	PELITINIB	inhibitor	MyCancerGenome
EGFR	EGFR - epidermal growth factor receptor	AZD8931	inhibitor	MyCancerGenome
EGFR	EGFR - epidermal growth factor receptor	RO5083945	antibody	MyCancerGenome
EGFR	EGFR - epidermal growth factor receptor	NECITUMUMAB	antibody	MyCancerGenome
EGFR	EGFR - epidermal growth factor receptor	ERLOTINIB	inhibitor	MyCancerGenome
EGFR	EGFR - epidermal growth factor receptor	ABT-806	antibody	MyCancerGenome
EGFR	EGFR - epidermal growth factor receptor	DACOMITINIB	inhibitor	MyCancerGenome
EGFR	EGFR - epidermal growth factor receptor	BMS-599626	inhibitor	MyCancerGenome
EGFR	EGFR - epidermal growth factor receptor	AKOS004227608	n/a	ClearityFoundationClinicalTrial
EGFR	EGFR - epidermal growth factor receptor	VANDETANIB	n/a	ClearityFoundationClinicalTrial
EGFR	EGFR - epidermal growth factor receptor	AKOS004227608	n/a	ClearityFoundationClinicalTrial
EGFR	EGFR - epidermal growth factor receptor	ERLOTINIB	n/a	ClearityFoundationClinicalTrial
EGFR	EGFR - epidermal growth factor receptor	LAPATINIB	n/a	ClearityFoundationClinicalTrial
EGFR	EGFR - epidermal growth factor receptor	LAPATINIB	n/a	ClearityFoundationClinicalTrial
EGFR	EGFR - epidermal growth factor receptor	CUDC-101	inhibitor	TALC
EGFR	EGFR - epidermal growth factor receptor	CETUXIMAB	inhibitor	TALC
EGFR	EGFR - epidermal growth factor receptor	ERLOTINIB	inhibitor	TALC

Supplemental Table 9. DT Network of primary screen target genes

EGFR	EGFR - epidermal growth factor receptor	BMS-690514	inhibitor	TALC
EGFR	EGFR - epidermal growth factor receptor	ARRY 380	inhibitor	TALC
EGFR	EGFR - epidermal growth factor receptor	MM151	inhibitor	TALC
EGFR	EGFR - epidermal growth factor receptor	MM111	inhibitor	TALC
EGFR	EGFR - epidermal growth factor receptor	RO5083945	inhibitor	TALC
EGFR	EGFR - epidermal growth factor receptor	MOMELOTINIB	inhibitor	TALC
EGFR	EGFR - epidermal growth factor receptor	RECOMBINANT HUMANEGF-RP64K/MONTANIDE	vaccine	TALC
EGFR	EGFR - epidermal growth factor receptor	LAPATINIB	inhibitor	TALC
EGFR	EGFR - epidermal growth factor receptor	AFATINIB	inhibitor	TALC
EGFR	EGFR - epidermal growth factor receptor	ICOTINIB	inhibitor	TALC
EGFR	EGFR - epidermal growth factor receptor	NIMOTUZUMAB	inhibitor	TALC
EGFR	EGFR - epidermal growth factor receptor	MM121	inhibitor	TALC
EGFR	EGFR - epidermal growth factor receptor	AV-412	inhibitor	TALC
EGFR	EGFR - epidermal growth factor receptor	MEHD7945	inhibitor	TALC
EGFR	EGFR - epidermal growth factor receptor	AV 203	inhibitor	TALC
EGFR	EGFR - epidermal growth factor receptor	CANERTINIB	inhibitor	TALC
EGFR	EGFR - epidermal growth factor receptor	MATUZUMAB	inhibitor	TALC
EGFR	EGFR - epidermal growth factor receptor	ZALUTUMUMAB	inhibitor	TALC
EGFR	EGFR - epidermal growth factor receptor	VANDETANIB	inhibitor	TALC
EGFR	EGFR - epidermal growth factor receptor	XL647	inhibitor	TALC
EGFR	EGFR - epidermal growth factor receptor	AEE 788	inhibitor	TALC
EGFR	EGFR - epidermal growth factor receptor	GEFITINIB	inhibitor	TALC
EGFR	EGFR - epidermal growth factor receptor	PANITUMUMAB	inhibitor	TALC
EGFR	EGFR - epidermal growth factor receptor	NECITUMUMAB	inhibitor	TALC
EGFR	EGFR - epidermal growth factor receptor	NERATINIB	inhibitor	TALC
EGFR	EGFR - epidermal growth factor receptor	DACOMITINIB	inhibitor	TALC
EGFR	EGFR - epidermal growth factor receptor	PERTUZUMAB	inhibitor	TALC
EGFR	EGFR - epidermal growth factor receptor	AMG888	inhibitor	TALC
EGFR	EGFR - epidermal growth factor receptor	CO 1686	inhibitor	TALC
EGFR	EGFR - epidermal growth factor receptor	PELITINIB	inhibitor	TALC
EGFR	EGFR - epidermal growth factor receptor	BMS-599626	inhibitor	TALC
EGFR	EGFR - epidermal growth factor receptor	PF-00299804	inhibitor	CancerCommons
EGFR	EGFR - epidermal growth factor receptor	GELFITINIB	inhibitor	CancerCommons
EGFR	EGFR - epidermal growth factor receptor	LAPATINIB	inhibitor	CancerCommons
EGFR	EGFR - epidermal growth factor receptor	CETUXIMAB	antibody	CancerCommons
EGFR	EGFR - epidermal growth factor receptor	ERLOTINIB	inhibitor	CancerCommons
EGFR	EGFR - epidermal growth factor receptor	BIBW2992	inhibitor	CancerCommons
EGFR	EGFR - epidermal growth factor receptor	ERLOTINIB	n/a	ClearityFoundationBiomarkers
EGFR	EGFR - epidermal growth factor receptor	VANDETANIB	n/a	ClearityFoundationBiomarkers

Supplemental Table 9. DT Network of primary screen target genes

EGFR	EGFR - epidermal growth factor receptor	PANITUMUMAB	n/a	ClearityFoundationBiomarkers
EGFR	EGFR - epidermal growth factor receptor	CETUXIMAB	n/a	ClearityFoundationBiomarkers
EGFR	EGFR - epidermal growth factor receptor	GEFITINIB	n/a	ClearityFoundationBiomarkers
EPOR	EPOR - erythropoietin receptor	METHOXY POLYETHYLENE GLYCOL-EPOETIN BETA	n/a	TEND
EPOR	EPOR - erythropoietin receptor	EPOETIN ALFA	n/a	TEND
EPOR	EPOR - erythropoietin receptor	DARBEPOETIN ALFA	n/a	TEND
EPOR	EPOR - erythropoietin receptor	EPOETIN ALFA	agonist	DrugBank
EPOR	EPOR - erythropoietin receptor	3,5 DIBROMOTYROSINE	n/a	DrugBank
EPOR	EPOR - erythropoietin receptor	DARBEPOETIN ALFA	agonist	DrugBank
EPOR	EPOR - erythropoietin receptor	DARBEPOETIN ALFA	agonist	TTD
EPOR	EPOR - erythropoietin receptor	EPOETIN ALFA	agonist	TTD
EPOR	EPOR - erythropoietin receptor	HEMATIDE	agonist	TTD
ESR1	ESR1 - estrogen receptor 1	NORGESTIMATE	n/a	TEND
ESR1	ESR1 - estrogen receptor 1	ESTRONE	n/a	TEND
ESR1	ESR1 - estrogen receptor 1	ETONOGESTREL	n/a	TEND
ESR1	ESR1 - estrogen receptor 1	LEVONORGESTREL	n/a	TEND
ESR1	ESR1 - estrogen receptor 1	DESOGESTREL	n/a	TEND
ESR1	ESR1 - estrogen receptor 1	TOREMIFENE	n/a	TEND
ESR1	ESR1 - estrogen receptor 1	RALOXIFENE	n/a	TEND
ESR1	ESR1 - estrogen receptor 1	MESTRANOL	n/a	TEND
ESR1	ESR1 - estrogen receptor 1	DIENESTROL	n/a	TEND
ESR1	ESR1 - estrogen receptor 1	CLOMIFENE	n/a	TEND
ESR1	ESR1 - estrogen receptor 1	FULVESTRANT	n/a	TEND
ESR1	ESR1 - estrogen receptor 1	QUINESTROL	n/a	TEND
ESR1	ESR1 - estrogen receptor 1	DIETHYLSTILBESTROL	n/a	TEND
ESR1	ESR1 - estrogen receptor 1	ESTRIOL	n/a	TEND
ESR1	ESR1 - estrogen receptor 1	NORGESTREL	n/a	TEND
ESR1	ESR1 - estrogen receptor 1	CHLOROTRIANISENE	n/a	TEND
ESR1	ESR1 - estrogen receptor 1	MEGESTROL	n/a	TEND
ESR1	ESR1 - estrogen receptor 1	ETHINYL ESTRADIOL	n/a	TEND
ESR1	ESR1 - estrogen receptor 1	TAMOXIFEN	n/a	TEND
ESR1	ESR1 - estrogen receptor 1	ETHYNODIOL DIACETATE	n/a	TEND
ESR1	ESR1 - estrogen receptor 1	PROGESTERONE	n/a	TEND
ESR1	ESR1 - estrogen receptor 1	ESTRADIOL	n/a	TEND
ESR1	ESR1 - estrogen receptor 1	DANAZOL	n/a	TEND
ESR1	ESR1 - estrogen receptor 1	CONJUGATED ESTROGENS	n/a	TEND
ESR1	ESR1 - estrogen receptor 1	COMPOUND 4-D	n/a	DrugBank
ESR1	ESR1 - estrogen receptor 1	NORGESTIMATE	agonist	DrugBank
ESR1	ESR1 - estrogen receptor 1	3-CHLORO-2-(4-HYDROXYPHENYL)-2H-INDAZOL-5-OL	n/a	DrugBank
ESR1	ESR1 - estrogen receptor 1	ETONOGESTREL	agonist	DrugBank
ESR1	ESR1 - estrogen receptor 1	(9BETA,11ALPHA,13ALPHA,14BETA,17ALPHA)-11-(METH...	n/a	DrugBank
ESR1	ESR1 - estrogen receptor 1	ESTROPIPATE	agonist	DrugBank
ESR1	ESR1 - estrogen receptor 1	4-[(1S,2R,5S)-4,4,8-TRIMETHYL-3-OXABICYCLO[3.3....	n/a	DrugBank
ESR1	ESR1 - estrogen receptor 1	CONJUGATED ESTROGENS	agonist	DrugBank
ESR1	ESR1 - estrogen receptor 1	QUINESTROL	agonist/modulator	DrugBank
ESR1	ESR1 - estrogen receptor 1	DIMETHYL (1R,4S)-5,6-BIS(4-HYDROXYPHENYL)-7-OXA...	n/a	DrugBank
ESR1	ESR1 - estrogen receptor 1	2-PHENYL-1-[4-(2-PIPERIDIN-1-YL-ETHOXY)-PHENYL]...	n/a	DrugBank
ESR1	ESR1 - estrogen receptor 1	RALOXIFENE CORE	n/a	DrugBank
ESR1	ESR1 - estrogen receptor 1	TRILOSTANE	allosteric modulator	DrugBank
ESR1	ESR1 - estrogen receptor 1	(3AS,4R,9BR)-4-(4-HYDROXYPHENYL)-6-(METHOXYMETH...	n/a	DrugBank
ESR1	ESR1 - estrogen receptor 1	FULVESTRANT	antagonist	DrugBank
ESR1	ESR1 - estrogen receptor 1	COMPOUND 18	n/a	DrugBank
ESR1	ESR1 - estrogen receptor 1	ESTRAMUSTINE	agonist	DrugBank
ESR1	ESR1 - estrogen receptor 1	4-[(1S,2S,5S)-5-(HYDROXYMETHYL)-6,8,9-TRIMETHYL...	n/a	DrugBank
ESR1	ESR1 - estrogen receptor 1	DIETHYL (1R,2S,3R,4S)-5,6-BIS(4-HYDROXYPHENYL)-...	n/a	DrugBank
ESR1	ESR1 - estrogen receptor 1	N-[(1R)-3-(4-HYDROXYPHENYL)-1-METHYLPROPYL]-2-(...	n/a	DrugBank
ESR1	ESR1 - estrogen receptor 1	DESOGESTREL	agonist	DrugBank
ESR1	ESR1 - estrogen receptor 1	2-AMINO-1-METHYL-6-PHENYLIMIDAZO[4,5-B]PYRIDINE	n/a	DrugBank
ESR1	ESR1 - estrogen receptor 1	NORELGESTROMIN	agonist	DrugBank
ESR1	ESR1 - estrogen receptor 1	ETHYNODIOL DIACETATE	agonist	DrugBank
ESR1	ESR1 - estrogen receptor 1	[5-HYDROXY-2-(4-HYDROXYPHENYL)-1-BENZOFURAN-7-Y...	n/a	DrugBank
ESR1	ESR1 - estrogen receptor 1	CLOMIFENE	antagonist/agonist	DrugBank
ESR1	ESR1 - estrogen receptor 1	ETHINYL ESTRADIOL	agonist	DrugBank

Supplemental Table 9. DT Network of primary screen target genes

ESR1	ESR1 - estrogen receptor 1	4-[(1S,2S,5S)-5-(HYDROXYMETHYL)-8-METHYL-3-OXAB...	n/a	DrugBank
ESR1	ESR1 - estrogen receptor 1	FLUOXYMESTERONE	antagonist	DrugBank
ESR1	ESR1 - estrogen receptor 1	CHLOROTRIANISENE	agonist	DrugBank
ESR1	ESR1 - estrogen receptor 1	TOREMIFENE	modulator	DrugBank
ESR1	ESR1 - estrogen receptor 1	NALOXONE	other/unknown	DrugBank
ESR1	ESR1 - estrogen receptor 1	17-METHYL-17-ALPHA-DIHYDROEQUILENIN	n/a	DrugBank
ESR1	ESR1 - estrogen receptor 1	(9ALPHA,13BETA,17BETA)-2-[(1Z)-BUT-1-EN-1-YL]ES...	n/a	DrugBank
ESR1	ESR1 - estrogen receptor 1	DANAZOL	agonist	DrugBank
ESR1	ESR1 - estrogen receptor 1	ALLYLESTRENOL	agonist	DrugBank
ESR1	ESR1 - estrogen receptor 1	RALOXIFENE	agonist	DrugBank
ESR1	ESR1 - estrogen receptor 1	LEVONORGESTREL	other/unknown	DrugBank
ESR1	ESR1 - estrogen receptor 1	(2R,3R,4S)-3-(4-HYDROXYPHENYL)-4-METHYL-2-[4-(2...	n/a	DrugBank
ESR1	ESR1 - estrogen receptor 1	GENISTEIN	n/a	DrugBank
ESR1	ESR1 - estrogen receptor 1	ESTRADIOL	agonist	DrugBank
ESR1	ESR1 - estrogen receptor 1	1-[4-(OCTAHYDRO-PYRIDO[1,2-A]PYRAZIN-2-YL)-PHEN...	n/a	DrugBank
ESR1	ESR1 - estrogen receptor 1	COMPOUND 19	n/a	DrugBank
ESR1	ESR1 - estrogen receptor 1	MEDROXYPROGESTERONE	agonist	DrugBank
ESR1	ESR1 - estrogen receptor 1	ESTRIOL	agonist	DrugBank
ESR1	ESR1 - estrogen receptor 1	MESTRANOL	agonist	DrugBank
ESR1	ESR1 - estrogen receptor 1	MELATONIN	antagonist	DrugBank
ESR1	ESR1 - estrogen receptor 1	PROGESTERONE	agonist	DrugBank
ESR1	ESR1 - estrogen receptor 1	4-(6-HYDROXY-1H-INDAZOL-3-YL)BENZENE-1,3-DIOL	n/a	DrugBank
ESR1	ESR1 - estrogen receptor 1	DIETHYLSTILBESTROL	agonist	DrugBank
ESR1	ESR1 - estrogen receptor 1	TAMOXIFEN	agonist/antagonist	DrugBank
ESR1	ESR1 - estrogen receptor 1	4-(2-AMINO-1-METHYL-1H-IMIDAZO[4,5-B]PYRIDIN-6-...	n/a	DrugBank
ESR1	ESR1 - estrogen receptor 1	4-[1-ALLYL-7-(TRIFLUOROMETHYL)-1H-INDAZOL-3-YL]...	n/a	DrugBank
ESR1	ESR1 - estrogen receptor 1	(3AS,4R,9BR)-4-(4-HYDROXYPHENYL)-1,2,3,3A,4,9B-...	n/a	DrugBank
ESR1	ESR1 - estrogen receptor 1	(3AS,4R,9BR)-2,2-DIFLUORO-4-(4-HYDROXYPHENYL)-1...	n/a	DrugBank
ESR1	ESR1 - estrogen receptor 1	ESTRONE	agonist	DrugBank
ESR1	ESR1 - estrogen receptor 1	4-[(1S,2S,5S,9R)-5-(HYDROXYMETHYL)-8,9-DIMETHYL...	n/a	DrugBank
ESR1	ESR1 - estrogen receptor 1	DIENESTROL	agonist	DrugBank
ESR1	ESR1 - estrogen receptor 1	3-ETHYL-2-(4-HYDROXYPHENYL)-2H-INDAZOL-5-OL	n/a	DrugBank
ESR1	ESR1 - estrogen receptor 1	(3AS,4R,9BR)-4-(4-HYDROXYPHENYL)-1,2,3,3A,4,9B-...	n/a	DrugBank
ESR1	ESR1 - estrogen receptor 1	LEFLUNOMIDE	n/a	PharmGKB
ESR1	ESR1 - estrogen receptor 1	ALKYLATING AGENTS	n/a	PharmGKB
ESR1	ESR1 - estrogen receptor 1	MEDROXYPROGESTERONE	n/a	PharmGKB
ESR1	ESR1 - estrogen receptor 1	EXEMESTANE	n/a	PharmGKB
ESR1	ESR1 - estrogen receptor 1	CONJUGATED ESTROGENS	n/a	PharmGKB
ESR1	ESR1 - estrogen receptor 1	CISPLATIN	n/a	PharmGKB
ESR1	ESR1 - estrogen receptor 1	FULVESTRANT	n/a	PharmGKB
ESR1	ESR1 - estrogen receptor 1	ENDOXIFEN	n/a	ClearityFoundationClinicalTrial
ESR1	ESR1 - estrogen receptor 1	EC145	n/a	ClearityFoundationClinicalTrial
ESR1	ESR1 - estrogen receptor 1	EC145	n/a	ClearityFoundationClinicalTrial
ESR1	ESR1 - estrogen receptor 1	TAMOXIFEN	n/a	ClearityFoundationClinicalTrial
ESR1	ESR1 - estrogen receptor 1	N/A	n/a	ClearityFoundationClinicalTrial
ESR1	ESR1 - estrogen receptor 1	FULVESTRANT	antagonist	TALC
ESR1	ESR1 - estrogen receptor 1	TAMOXIFEN	n/a	ClearityFoundationBiomarkers
ESR1	ESR1 - estrogen receptor 1	ANASTROZOLE	n/a	ClearityFoundationBiomarkers
ESR1	ESR1 - estrogen receptor 1	LETROZOLE	n/a	ClearityFoundationBiomarkers
ESR2	ESR2 - estrogen receptor 2 (ER beta)	ESTRADIOL	n/a	TEND
ESR2	ESR2 - estrogen receptor 2 (ER beta)	RALOXIFENE	n/a	TEND
ESR2	ESR2 - estrogen receptor 2 (ER beta)	TAMOXIFEN	n/a	TEND
ESR2	ESR2 - estrogen receptor 2 (ER beta)	2-(5-HYDROXY-NAPHTHALEN-1-YL)-1,3-BENZOOXAZOL-6-OL	n/a	DrugBank
ESR2	ESR2 - estrogen receptor 2 (ER beta)	ESTRADIOL	agonist	DrugBank
ESR2	ESR2 - estrogen receptor 2 (ER beta)	GENISTEIN	n/a	DrugBank
ESR2	ESR2 - estrogen receptor 2 (ER beta)	TAMOXIFEN	agonist/antagonist	DrugBank

Supplemental Table 9. DT Network of primary screen target genes

ESR2	ESR2 - estrogen receptor 2 (ER beta)	(9AS)-4-BROMO-9A-BUTYL-7-HYDROXY-1,2,9,9A-TETRA...	n/a	DrugBank
ESR2	ESR2 - estrogen receptor 2 (ER beta)	4-(6-HYDROXY-BENZO[D]ISOXAZOL-3-YL)BENZENE-1,3-...	n/a	DrugBank
ESR2	ESR2 - estrogen receptor 2 (ER beta)	4-(2-{[4-{[3-(4-CHLOROPHENYL)PROPYL]SULFANYL}-6...	n/a	DrugBank
ESR2	ESR2 - estrogen receptor 2 (ER beta)	5-ALPHA-ANDROSTANE-3-BETA,17BETA-DIOL	agonist	DrugBank
ESR2	ESR2 - estrogen receptor 2 (ER beta)	[5-HYDROXY-2-(4-HYDROXYPHENYL)-1-BENZOFURAN-7-Y...	n/a	DrugBank
ESR2	ESR2 - estrogen receptor 2 (ER beta)	ESTRAMUSTINE	other/unknown	DrugBank
ESR2	ESR2 - estrogen receptor 2 (ER beta)	(3AS,4R,9BR)-4-(4-HYDROXYPHENYL)-6-(METHOXYMETH...	n/a	DrugBank
ESR2	ESR2 - estrogen receptor 2 (ER beta)	N-BUTYL-11-[(7R,8R,9S,13S,14S,17S)-3,17-DIHYDRO...	n/a	DrugBank
ESR2	ESR2 - estrogen receptor 2 (ER beta)	4-(4-HYDROXYPHENYL)-1-NAPHTHALDEHYDE OXIME	n/a	DrugBank
ESR2	ESR2 - estrogen receptor 2 (ER beta)	1-CHLORO-6-(4-HYDROXYPHENYL)-2-NAPHTHOL	n/a	DrugBank
ESR2	ESR2 - estrogen receptor 2 (ER beta)	(3AS,4R,9BR)-4-(4-HYDROXYPHENYL)-1,2,3,3A,4,9B-...	n/a	DrugBank
ESR2	ESR2 - estrogen receptor 2 (ER beta)	ESTROPIPATE	agonist	DrugBank
ESR2	ESR2 - estrogen receptor 2 (ER beta)	3-BROMO-6-HYDROXY-2-(4-HYDROXYPHENYL)-1H-INDEN-...	n/a	DrugBank
ESR2	ESR2 - estrogen receptor 2 (ER beta)	(3AS,4R,9BR)-2,2-DIFLUORO-4-(4-HYDROXYPHENYL)-6...	n/a	DrugBank
ESR2	ESR2 - estrogen receptor 2 (ER beta)	(3AS,4R,9BR)-2,2-DIFLUORO-4-(4-HYDROXYPHENYL)-1...	n/a	DrugBank
ESR2	ESR2 - estrogen receptor 2 (ER beta)	PARA-MERCURY-BENZENESULFONIC ACID	n/a	DrugBank
ESR2	ESR2 - estrogen receptor 2 (ER beta)	(16ALPHA,17ALPHA)-ESTRA-1,3,5(10)-TRIENE-3,16,1...	n/a	DrugBank
ESR2	ESR2 - estrogen receptor 2 (ER beta)	5-HYDROXY-2-(4-HYDROXYPHENYL)-1-BENZOFURAN-7-CA...	n/a	DrugBank
ESR2	ESR2 - estrogen receptor 2 (ER beta)	DIETHYLSTILBESTROL	agonist	DrugBank
ESR2	ESR2 - estrogen receptor 2 (ER beta)	RALOXIFENE	agonist	DrugBank
ESR2	ESR2 - estrogen receptor 2 (ER beta)	3-(6-HYDROXY-NAPHTHALEN-2-YL)-BENZO[D]ISOOXAZOL...	n/a	DrugBank
ESR2	ESR2 - estrogen receptor 2 (ER beta)	2-(3-FLUORO-4-HYDROXYPHENYL)-7-VINYL-1,3-BENZOX...	n/a	DrugBank
ESR2	ESR2 - estrogen receptor 2 (ER beta)	3-(3-FLUORO-4-HYDROXYPHENYL)-7-HYDROXY-1-NAPHTH...	n/a	DrugBank
ESR2	ESR2 - estrogen receptor 2 (ER beta)	TRILOSTANE	allosteric modulator	DrugBank
ESR2	ESR2 - estrogen receptor 2 (ER beta)	2-(4-HYDROXY-PHENYL)BENZOFURAN-5-OL	n/a	DrugBank
ESR2	ESR2 - estrogen receptor 2 (ER beta)	ESTRIOL	agonist	DrugBank
ESR2	ESR2 - estrogen receptor 2 (ER beta)	CONJUGATED ESTROGENS	antagonist	TTD
ESR2	ESR2 - estrogen receptor 2 (ER beta)	FULVESTRANT	n/a	PharmGKB
ESR2	ESR2 - estrogen receptor 2 (ER beta)	EXEMESTANE	n/a	PharmGKB
ESR2	ESR2 - estrogen receptor 2 (ER beta)	ALENDRONATE	n/a	PharmGKB
ESR2	ESR2 - estrogen receptor 2 (ER beta)	TAMOXIFEN	n/a	PharmGKB
F9	F9 - coagulation factor IX	MENADIONE	n/a	TEND
F9	F9 - coagulation factor IX	COAGULATION FACTOR VIIA	n/a	TEND
F9	F9 - coagulation factor IX	MENADIONE	n/a	TEND
F9	F9 - coagulation factor IX	MENADIONE	activator	DrugBank
F9	F9 - coagulation factor IX	ANTIHEMOPHILIC FACTOR	cofactor	DrugBank
F9	F9 - coagulation factor IX	GAMMA-CARBOXY-GLUTAMIC ACID	n/a	DrugBank
FAS	FAS - fatty acid synthase	CERULENIN	inhibitor	TTD
FAS	FAS - fatty acid synthase	FSA2	inhibitor	TTD
FAS	FAS - fatty acid synthase	FAS1	inhibitor	TTD
FGFR1	FGFR1 - fibroblast growth factor receptor 1	PALIFERMIN	n/a	DrugBank
FGFR1	FGFR1 - fibroblast growth factor receptor 1	SU4984	n/a	DrugBank
FGFR1	FGFR1 - fibroblast growth factor receptor 1	(E)-[4-(3,5-DIFLUOROPHENYL)-3H-PYRROLO[2,3-B]PY...	n/a	DrugBank
FGFR1	FGFR1 - fibroblast growth factor receptor 1	3-[(3-(2-CARBOXYETHYL)-4-METHYLPYRROL-2-YL)METH...	n/a	DrugBank
FGFR1	FGFR1 - fibroblast growth factor receptor 1	3-(3-METHOXYBENZYL)-1H-PYRROLO[2,3-B]PYRIDINE	n/a	DrugBank
FGFR1	FGFR1 - fibroblast growth factor receptor 1	BRIVANIB	n/a	PharmGKB
FGFR1	FGFR1 - fibroblast growth factor receptor 1	E-3810	inhibitor	MyCancerGenome
FGFR1	FGFR1 - fibroblast growth factor receptor 1	ORANTINIB	inhibitor	MyCancerGenome

Supplemental Table 9. DT Network of primary screen target genes

FGFR1	FGFR1 - fibroblast growth factor receptor 1	XL228	inhibitor	MyCancerGenome
FGFR1	FGFR1 - fibroblast growth factor receptor 1	DOVITINIB	inhibitor	MyCancerGenome
FGFR1	FGFR1 - fibroblast growth factor receptor 1	BGJ398	inhibitor	MyCancerGenome
FGFR1	FGFR1 - fibroblast growth factor receptor 1	PONATINIB	inhibitor	MyCancerGenome
FGFR1	FGFR1 - fibroblast growth factor receptor 1	MASITINIB	inhibitor	MyCancerGenome
FGFR1	FGFR1 - fibroblast growth factor receptor 1	AZD4547	inhibitor	MyCancerGenome
FGFR1	FGFR1 - fibroblast growth factor receptor 1	NINTEDANIB	inhibitor	MyCancerGenome
FGFR1	FGFR1 - fibroblast growth factor receptor 1	DOVITINIB	n/a	ClearityFoundationClinicalTrial
FGFR1	FGFR1 - fibroblast growth factor receptor 1	ENMD-2076	n/a	ClearityFoundationClinicalTrial
FGFR1	FGFR1 - fibroblast growth factor receptor 1	NINTEDANIB	n/a	ClearityFoundationClinicalTrial
FGFR1	FGFR1 - fibroblast growth factor receptor 1	NINTEDANIB	n/a	ClearityFoundationClinicalTrial
FGFR1	FGFR1 - fibroblast growth factor receptor 1	VARGATEF	inhibitor	TALC
FGFR1	FGFR1 - fibroblast growth factor receptor 1	AZD4547	inhibitor	TALC
FGFR1	FGFR1 - fibroblast growth factor receptor 1	AZD4547	inhibitor	TALC
FGFR1	FGFR1 - fibroblast growth factor receptor 1	FP-1039	antibody	TALC
FGFR1	FGFR1 - fibroblast growth factor receptor 1	XL228	inhibitor	TALC
FGFR1	FGFR1 - fibroblast growth factor receptor 1	BGJ398	inhibitor	TALC
FGFR1	FGFR1 - fibroblast growth factor receptor 1	PONATINIB	inhibitor	TALC
FGFR1	FGFR1 - fibroblast growth factor receptor 1	DOVITINIB	inhibitor	TALC
FGFR1	FGFR1 - fibroblast growth factor receptor 1	BGJ398	inhibitor	CancerCommons
FXYD2	FXYD2 - FXYD domain containing ion transport regulator 2	CYCLOTHIAZIDE	n/a	TEND
FXYD2	FXYD2 - FXYD domain containing ion transport regulator 2	CYCLOTHIAZIDE	inhibitor	DrugBank
GABRA2	GABRA2 - gamma-aminobutyric acid (GABA) A receptor, alpha 2	PENTOBARBITAL	n/a	TEND
GABRA2	GABRA2 - gamma-aminobutyric acid (GABA) A receptor, alpha 2	CINOLAZEPAM	n/a	TEND
GABRA2	GABRA2 - gamma-aminobutyric acid (GABA) A receptor, alpha 2	CHLORDIAZEPOXIDE	n/a	TEND
GABRA2	GABRA2 - gamma-aminobutyric acid (GABA) A receptor, alpha 2	NITRAZEPAM	n/a	TEND
GABRA2	GABRA2 - gamma-aminobutyric acid (GABA) A receptor, alpha 2	FLUNITRAZEPAM	n/a	TEND
GABRA2	GABRA2 - gamma-aminobutyric acid (GABA) A receptor, alpha 2	SECOBARBITAL	n/a	TEND
GABRA2	GABRA2 - gamma-aminobutyric acid (GABA) A receptor, alpha 2	ADINAZOLAM	n/a	TEND
GABRA2	GABRA2 - gamma-aminobutyric acid (GABA) A receptor, alpha 2	TALBUTAL	n/a	TEND
GABRA2	GABRA2 - gamma-aminobutyric acid (GABA) A receptor, alpha 2	FLURAZEPAM	n/a	TEND
GABRA2	GABRA2 - gamma-aminobutyric acid (GABA) A receptor, alpha 2	HALAZEPAM	n/a	TEND
GABRA2	GABRA2 - gamma-aminobutyric acid (GABA) A receptor, alpha 2	CLOBAZAM	n/a	TEND
GABRA2	GABRA2 - gamma-aminobutyric acid (GABA) A receptor, alpha 2	FLUMAZENIL	n/a	TEND
GABRA2	GABRA2 - gamma-aminobutyric acid (GABA) A receptor, alpha 2	MEPROBAMATE	n/a	TEND
GABRA2	GABRA2 - gamma-aminobutyric acid (GABA) A receptor, alpha 2	METHYLPHENOBARBITAL	n/a	TEND
GABRA2	GABRA2 - gamma-aminobutyric acid (GABA) A receptor, alpha 2	TRIAZOLAM	n/a	TEND
GABRA2	GABRA2 - gamma-aminobutyric acid (GABA) A receptor, alpha 2	HEXOBARBITAL	n/a	TEND
GABRA2	GABRA2 - gamma-aminobutyric acid (GABA) A receptor, alpha 2	LORAZEPAM	n/a	TEND

Supplemental Table 9. DT Network of primary screen target genes

GABRA2	GABRA2 - gamma-aminobutyric acid (GABA) A receptor, alpha 2	ZOPICLONE	n/a	TEND
GABRA2	GABRA2 - gamma-aminobutyric acid (GABA) A receptor, alpha 2	PRIMIDONE	n/a	TEND
GABRA2	GABRA2 - gamma-aminobutyric acid (GABA) A receptor, alpha 2	BROMAZEPAM	n/a	TEND
GABRA2	GABRA2 - gamma-aminobutyric acid (GABA) A receptor, alpha 2	PHENOBARBITAL	n/a	TEND
GABRA2	GABRA2 - gamma-aminobutyric acid (GABA) A receptor, alpha 2	ALPRAZOLAM	n/a	TEND
GABRA2	GABRA2 - gamma-aminobutyric acid (GABA) A receptor, alpha 2	MIDAZOLAM	n/a	TEND
GABRA2	GABRA2 - gamma-aminobutyric acid (GABA) A receptor, alpha 2	CLOTIAZEPAM	n/a	TEND
GABRA2	GABRA2 - gamma-aminobutyric acid (GABA) A receptor, alpha 2	CLORAZEPATE	n/a	TEND
GABRA2	GABRA2 - gamma-aminobutyric acid (GABA) A receptor, alpha 2	PRAZEPAM	n/a	TEND
GABRA2	GABRA2 - gamma-aminobutyric acid (GABA) A receptor, alpha 2	THIOPENTAL	n/a	TEND
GABRA2	GABRA2 - gamma-aminobutyric acid (GABA) A receptor, alpha 2	QUAZEPAM	n/a	TEND
GABRA2	GABRA2 - gamma-aminobutyric acid (GABA) A receptor, alpha 2	ZOLPIDEM	n/a	TEND
GABRA2	GABRA2 - gamma-aminobutyric acid (GABA) A receptor, alpha 2	OXAZEPAM	n/a	TEND
GABRA2	GABRA2 - gamma-aminobutyric acid (GABA) A receptor, alpha 2	BUTETHAL	n/a	TEND
GABRA2	GABRA2 - gamma-aminobutyric acid (GABA) A receptor, alpha 2	CLONAZEPAM	n/a	TEND
GABRA2	GABRA2 - gamma-aminobutyric acid (GABA) A receptor, alpha 2	METHARBITAL	n/a	TEND
GABRA2	GABRA2 - gamma-aminobutyric acid (GABA) A receptor, alpha 2	TEMAZEPAM	n/a	TEND
GABRA2	GABRA2 - gamma-aminobutyric acid (GABA) A receptor, alpha 2	ESTAZOLAM	n/a	TEND
GABRA2	GABRA2 - gamma-aminobutyric acid (GABA) A receptor, alpha 2	BUTABARBITAL	n/a	TEND
GABRA2	GABRA2 - gamma-aminobutyric acid (GABA) A receptor, alpha 2	BUTALBITAL	n/a	TEND
GABRA2	GABRA2 - gamma-aminobutyric acid (GABA) A receptor, alpha 2	FLUDIAZEPAM	n/a	TEND
GABRA2	GABRA2 - gamma-aminobutyric acid (GABA) A receptor, alpha 2	DIAZEPAM	n/a	TEND
GABRA2	GABRA2 - gamma-aminobutyric acid (GABA) A receptor, alpha 2	TALBUTAL	potentiator	DrugBank
GABRA2	GABRA2 - gamma-aminobutyric acid (GABA) A receptor, alpha 2	DIAZEPAM	potentiator	DrugBank
GABRA2	GABRA2 - gamma-aminobutyric acid (GABA) A receptor, alpha 2	FLURAZEPAM	potentiator	DrugBank
GABRA2	GABRA2 - gamma-aminobutyric acid (GABA) A receptor, alpha 2	FLUMAZENIL	n/a	DrugBank
GABRA2	GABRA2 - gamma-aminobutyric acid (GABA) A receptor, alpha 2	ETHCHLORVYNOL	agonist	DrugBank
GABRA2	GABRA2 - gamma-aminobutyric acid (GABA) A receptor, alpha 2	METHARBITAL	potentiator	DrugBank
GABRA2	GABRA2 - gamma-aminobutyric acid (GABA) A receptor, alpha 2	MEPROBAMATE	agonist	DrugBank
GABRA2	GABRA2 - gamma-aminobutyric acid (GABA) A receptor, alpha 2	QUINIDINE BARBITURATE	potentiator	DrugBank
GABRA2	GABRA2 - gamma-aminobutyric acid (GABA) A receptor, alpha 2	ZOPICLONE	potentiator	DrugBank
GABRA2	GABRA2 - gamma-aminobutyric acid (GABA) A receptor, alpha 2	ZOLPIDEM	agonist	DrugBank
GABRA2	GABRA2 - gamma-aminobutyric acid (GABA) A receptor, alpha 2	BARBITURIC ACID DERIVATIVE	potentiator	DrugBank
GABRA2	GABRA2 - gamma-aminobutyric acid (GABA) A receptor, alpha 2	ADINAZOLAM	potentiator	DrugBank
GABRA2	GABRA2 - gamma-aminobutyric acid (GABA) A receptor, alpha 2	LORAZEPAM	potentiator	DrugBank
GABRA2	GABRA2 - gamma-aminobutyric acid (GABA) A receptor, alpha 2	ESTAZOLAM	potentiator	DrugBank
GABRA2	GABRA2 - gamma-aminobutyric acid (GABA) A receptor, alpha 2	MIDAZOLAM	potentiator	DrugBank
GABRA2	GABRA2 - gamma-aminobutyric acid (GABA) A receptor, alpha 2	PENTOBARBITAL	potentiator	DrugBank
GABRA2	GABRA2 - gamma-aminobutyric acid (GABA) A receptor, alpha 2	AMOBARBITAL	potentiator	DrugBank

Supplemental Table 9. DT Network of primary screen target genes

GABRA2	GABRA2 - gamma-aminobutyric acid (GABA) A receptor, alpha 2	QUAZEPAM	potentiator	DrugBank
GABRA2	GABRA2 - gamma-aminobutyric acid (GABA) A receptor, alpha 2	BUTALBITAL	potentiator	DrugBank
GABRA2	GABRA2 - gamma-aminobutyric acid (GABA) A receptor, alpha 2	BROMAZEPAM	potentiator	DrugBank
GABRA2	GABRA2 - gamma-aminobutyric acid (GABA) A receptor, alpha 2	HALAZEPAM	potentiator	DrugBank
GABRA2	GABRA2 - gamma-aminobutyric acid (GABA) A receptor, alpha 2	HEPTABARBITAL	potentiator	DrugBank
GABRA2	GABRA2 - gamma-aminobutyric acid (GABA) A receptor, alpha 2	APROBARBITAL	potentiator	DrugBank
GABRA2	GABRA2 - gamma-aminobutyric acid (GABA) A receptor, alpha 2	FLUNITRAZEPAM	potentiator	DrugBank
GABRA2	GABRA2 - gamma-aminobutyric acid (GABA) A receptor, alpha 2	NITRAZEPAM	potentiator	DrugBank
GABRA2	GABRA2 - gamma-aminobutyric acid (GABA) A receptor, alpha 2	OXAZEPAM	potentiator	DrugBank
GABRA2	GABRA2 - gamma-aminobutyric acid (GABA) A receptor, alpha 2	METHYLPHENOBARBITAL	potentiator	DrugBank
GABRA2	GABRA2 - gamma-aminobutyric acid (GABA) A receptor, alpha 2	ESZOPICLONE	agonist	DrugBank
GABRA2	GABRA2 - gamma-aminobutyric acid (GABA) A receptor, alpha 2	ALPRAZOLAM	potentiator	DrugBank
GABRA2	GABRA2 - gamma-aminobutyric acid (GABA) A receptor, alpha 2	BUTABARBITAL	potentiator	DrugBank
GABRA2	GABRA2 - gamma-aminobutyric acid (GABA) A receptor, alpha 2	PHENOBARBITAL	potentiator	DrugBank
GABRA2	GABRA2 - gamma-aminobutyric acid (GABA) A receptor, alpha 2	TRIAZOLAM	potentiator	DrugBank
GABRA2	GABRA2 - gamma-aminobutyric acid (GABA) A receptor, alpha 2	CINOLAZEPAM	potentiator	DrugBank
GABRA2	GABRA2 - gamma-aminobutyric acid (GABA) A receptor, alpha 2	FLUDIAZEPAM	potentiator	DrugBank
GABRA2	GABRA2 - gamma-aminobutyric acid (GABA) A receptor, alpha 2	PRIMIDONE	potentiator	DrugBank
GABRA2	GABRA2 - gamma-aminobutyric acid (GABA) A receptor, alpha 2	SECOBARBITAL	potentiator	DrugBank
GABRA2	GABRA2 - gamma-aminobutyric acid (GABA) A receptor, alpha 2	HEXOBARBITAL	potentiator	DrugBank
GABRA2	GABRA2 - gamma-aminobutyric acid (GABA) A receptor, alpha 2	CLONAZEPAM	potentiator	DrugBank
GABRA2	GABRA2 - gamma-aminobutyric acid (GABA) A receptor, alpha 2	BARBITAL	potentiator	DrugBank
GABRA2	GABRA2 - gamma-aminobutyric acid (GABA) A receptor, alpha 2	PRAZEPAM	potentiator	DrugBank
GABRA2	GABRA2 - gamma-aminobutyric acid (GABA) A receptor, alpha 2	TEMAZEPAM	potentiator	DrugBank
GABRA2	GABRA2 - gamma-aminobutyric acid (GABA) A receptor, alpha 2	CLOTIAZEPAM	potentiator	DrugBank
GABRA2	GABRA2 - gamma-aminobutyric acid (GABA) A receptor, alpha 2	THIOPENTAL	potentiator	DrugBank
GABRA2	GABRA2 - gamma-aminobutyric acid (GABA) A receptor, alpha 2	BUTETHAL	potentiator	DrugBank
GABRA2	GABRA2 - gamma-aminobutyric acid (GABA) A receptor, alpha 2	CLORAZEPATE	potentiator	DrugBank
GABRA2	GABRA2 - gamma-aminobutyric acid (GABA) A receptor, alpha 2	CHLORDIAZEPOXIDE	potentiator	DrugBank
GABRA2	GABRA2 - gamma-aminobutyric acid (GABA) A receptor, alpha 2	CLOBAZAM	potentiator	DrugBank
GABRE	GABRE - gamma-aminobutyric acid (GABA) A receptor, epsilon	OXAZEPAM	n/a	TEND
GABRE	GABRE - gamma-aminobutyric acid (GABA) A receptor, epsilon	LORAZEPAM	n/a	TEND
GABRE	GABRE - gamma-aminobutyric acid (GABA) A receptor, epsilon	HALAZEPAM	n/a	TEND
GABRE	GABRE - gamma-aminobutyric acid (GABA) A receptor, epsilon	CLOBAZAM	n/a	TEND
GABRE	GABRE - gamma-aminobutyric acid (GABA) A receptor, epsilon	NITRAZEPAM	n/a	TEND
GABRE	GABRE - gamma-aminobutyric acid (GABA) A receptor, epsilon	ALPRAZOLAM	n/a	TEND
GABRE	GABRE - gamma-aminobutyric acid (GABA) A receptor, epsilon	TRIAZOLAM	n/a	TEND
GABRE	GABRE - gamma-aminobutyric acid (GABA) A receptor, epsilon	FLURAZEPAM	n/a	TEND
GABRE	GABRE - gamma-aminobutyric acid (GABA) A receptor, epsilon	QUAZEPAM	n/a	TEND

Supplemental Table 9. DT Network of primary screen target genes

GABRE	GABRE - gamma-aminobutyric acid (GABA) A receptor, epsilon	ADINAZOLAM	n/a	TEND
GABRE	GABRE - gamma-aminobutyric acid (GABA) A receptor, epsilon	DIAZEPAM	n/a	TEND
GABRE	GABRE - gamma-aminobutyric acid (GABA) A receptor, epsilon	CHLORDIAZEPOXIDE	n/a	TEND
GABRE	GABRE - gamma-aminobutyric acid (GABA) A receptor, epsilon	CLORAZEPATE	n/a	TEND
GABRE	GABRE - gamma-aminobutyric acid (GABA) A receptor, epsilon	TEMAZEPAM	n/a	TEND
GABRE	GABRE - gamma-aminobutyric acid (GABA) A receptor, epsilon	CLOTIAZEPAM	n/a	TEND
GABRE	GABRE - gamma-aminobutyric acid (GABA) A receptor, epsilon	CLONAZEPAM	n/a	TEND
GABRE	GABRE - gamma-aminobutyric acid (GABA) A receptor, epsilon	CINOLAZEPAM	n/a	TEND
GABRE	GABRE - gamma-aminobutyric acid (GABA) A receptor, epsilon	BROMAZEPAM	n/a	TEND
GABRE	GABRE - gamma-aminobutyric acid (GABA) A receptor, epsilon	ESTAZOLAM	n/a	TEND
GABRE	GABRE - gamma-aminobutyric acid (GABA) A receptor, epsilon	MIDAZOLAM	n/a	TEND
GABRE	GABRE - gamma-aminobutyric acid (GABA) A receptor, epsilon	FLUDIAZEPAM	n/a	TEND
GABRE	GABRE - gamma-aminobutyric acid (GABA) A receptor, epsilon	PRAZEPAM	n/a	TEND
GABRE	GABRE - gamma-aminobutyric acid (GABA) A receptor, epsilon	ESTAZOLAM	potentiator	DrugBank
GABRE	GABRE - gamma-aminobutyric acid (GABA) A receptor, epsilon	FLURAZEPAM	potentiator	DrugBank
GABRE	GABRE - gamma-aminobutyric acid (GABA) A receptor, epsilon	CLORAZEPATE	potentiator	DrugBank
GABRE	GABRE - gamma-aminobutyric acid (GABA) A receptor, epsilon	CLOTIAZEPAM	potentiator	DrugBank
GABRE	GABRE - gamma-aminobutyric acid (GABA) A receptor, epsilon	NITRAZEPAM	potentiator	DrugBank
GABRE	GABRE - gamma-aminobutyric acid (GABA) A receptor, epsilon	TRIAZOLAM	potentiator	DrugBank
GABRE	GABRE - gamma-aminobutyric acid (GABA) A receptor, epsilon	KETAZOLAM	potentiator	DrugBank
GABRE	GABRE - gamma-aminobutyric acid (GABA) A receptor, epsilon	BROMAZEPAM	potentiator	DrugBank
GABRE	GABRE - gamma-aminobutyric acid (GABA) A receptor, epsilon	ALPRAZOLAM	potentiator	DrugBank
GABRE	GABRE - gamma-aminobutyric acid (GABA) A receptor, epsilon	OXAZEPAM	potentiator	DrugBank
GABRE	GABRE - gamma-aminobutyric acid (GABA) A receptor, epsilon	QUAZEPAM	potentiator	DrugBank
GABRE	GABRE - gamma-aminobutyric acid (GABA) A receptor, epsilon	HALAZEPAM	potentiator	DrugBank
GABRE	GABRE - gamma-aminobutyric acid (GABA) A receptor, epsilon	ADINAZOLAM	potentiator	DrugBank
GABRE	GABRE - gamma-aminobutyric acid (GABA) A receptor, epsilon	DIAZEPAM	potentiator	DrugBank
GABRE	GABRE - gamma-aminobutyric acid (GABA) A receptor, epsilon	TEMAZEPAM	potentiator	DrugBank
GABRE	GABRE - gamma-aminobutyric acid (GABA) A receptor, epsilon	CLOBAZAM	potentiator	DrugBank
GABRE	GABRE - gamma-aminobutyric acid (GABA) A receptor, epsilon	CHLORDIAZEPOXIDE	potentiator	DrugBank
GABRE	GABRE - gamma-aminobutyric acid (GABA) A receptor, epsilon	CLONAZEPAM	potentiator	DrugBank
GABRE	GABRE - gamma-aminobutyric acid (GABA) A receptor, epsilon	PRAZEPAM	potentiator	DrugBank
GABRE	GABRE - gamma-aminobutyric acid (GABA) A receptor, epsilon	FLUDIAZEPAM	potentiator	DrugBank
GABRE	GABRE - gamma-aminobutyric acid (GABA) A receptor, epsilon	CINOLAZEPAM	potentiator	DrugBank
GABRE	GABRE - gamma-aminobutyric acid (GABA) A receptor, epsilon	MIDAZOLAM	potentiator	DrugBank
GABRE	GABRE - gamma-aminobutyric acid (GABA) A receptor, epsilon	LORAZEPAM	potentiator	DrugBank
GAD2	GAD2 - glutamate decarboxylase 2 (pancreatic islets and brain, 65kDa)	PYRIDOXAL PHOSPHATE	cofactor	DrugBank
GAD2	GAD2 - glutamate decarboxylase 2 (pancreatic islets and brain, 65kDa)	PYRIDOXAL PHOSPHATE	cofactor	DrugBank
GAD2	GAD2 - glutamate decarboxylase 2 (pancreatic islets and brain, 65kDa)	PHENELZINE	inhibitor	DrugBank

Supplemental Table 9. DT Network of primary screen target genes

GAD2	GAD2 - glutamate decarboxylase 2 (pancreatic islets and brain, 65kDa)	L-GLUTAMIC ACID	n/a	DrugBank
GALE	GALE - UDP-galactose-4-epimerase	URIDINE DIPHOSPHATE GALACTOSE	n/a	DrugBank
GALE	GALE - UDP-galactose-4-epimerase	TETRAMETHYLAMMONIUM ION	n/a	DrugBank
GALE	GALE - UDP-galactose-4-epimerase	NICOTINAMIDE-ADENINE-DINUCLEOTIDE	n/a	DrugBank
GALE	GALE - UDP-galactose-4-epimerase	GLUCOSE-URIDINE-C1,5'-DIPHOSPHATE	n/a	DrugBank
GALE	GALE - UDP-galactose-4-epimerase	URIDINE-DIPHOSPHATE-N-ACETYLGALACTOSAMINE	n/a	DrugBank
GALE	GALE - UDP-galactose-4-epimerase	URIDINE-DIPHOSPHATE-N-ACETYLGLUCOSAMINE	n/a	DrugBank
GALE	GALE - UDP-galactose-4-epimerase	URIDINE-5'-MONOPHOSPHATE GLUCOPYRANOSYL-MONOPHO...	n/a	DrugBank
GAMT	GAMT - guanidinoacetate N-methyltransferase	S-ADENOSYL-L-HOMOCYSTEINE	n/a	DrugBank
GAMT	GAMT - guanidinoacetate N-methyltransferase	N-[AMINO(IMINO)METHYL]GLYCINE	n/a	DrugBank
GAMT	GAMT - guanidinoacetate N-methyltransferase	GUANIDINE	n/a	DrugBank
GAMT	GAMT - guanidinoacetate N-methyltransferase	CREATINE	product of	DrugBank
GAPDH	GAPDH - glyceraldehyde-3-phosphate dehydrogenase	NICOTINAMIDE-ADENINE-DINUCLEOTIDE	n/a	DrugBank
GAPDH	GAPDH - glyceraldehyde-3-phosphate dehydrogenase	THIONICOTINAMIDE-ADENINE-DINUCLEOTIDE	n/a	DrugBank
GAPDH	GAPDH - glyceraldehyde-3-phosphate dehydrogenase	ADENOSINE-5-DIPHOSPHORIBOSE	n/a	DrugBank
GAPDH	GAPDH - glyceraldehyde-3-phosphate dehydrogenase	4-(2-AMINOETHYL)BENZENESULFONYL FLUORIDE	n/a	DrugBank
GAPDH	GAPDH - glyceraldehyde-3-phosphate dehydrogenase	NADH	n/a	DrugBank
GCAT	GCAT - glycine C-acetyltransferase	PYRIDOXAL PHOSPHATE	cofactor	DrugBank
GCAT	GCAT - glycine C-acetyltransferase	GLYCINE	n/a	DrugBank
GCLM	GCLM - glutamate-cysteine ligase, modifier subunit	L-GLUTAMIC ACID	n/a	DrugBank
GCLM	GCLM - glutamate-cysteine ligase, modifier subunit	L-CYSTEINE	n/a	DrugBank
GLRX	GLRX - glutaredoxin (thioltransferase)	GLUTATHIONE	n/a	DrugBank
GMDS	GMDS - GDP-mannose 4,6-dehydratase	GUANOSINE-5'-DIPHOSPHATE-RHAMNOSE	n/a	DrugBank
GMDS	GMDS - GDP-mannose 4,6-dehydratase	GUANOSINE-5'-DIPHOSPHATE	n/a	DrugBank
GNAS	GNAS - GNAS complex locus	2',5'-DIDEOXY-ADENOSINE 3'-MONOPHOSPHATE	n/a	DrugBank
GNAS	GNAS - GNAS complex locus	FORSKOLIN	n/a	DrugBank
GNAT1	GNAT1 - guanine nucleotide binding protein (G protein), alpha transducing activity polypeptide 1	TETRAFLUOROALUMINATE ION	n/a	DrugBank
GNAT1	GNAT1 - guanine nucleotide binding protein (G protein), alpha transducing activity polypeptide 1	GUANOSINE-5'-DIPHOSPHATE	n/a	DrugBank
GNAT1	GNAT1 - guanine nucleotide binding protein (G protein), alpha transducing activity polypeptide 1	HYDROXYDIMETHYLARSINE OXIDE	n/a	DrugBank
GRIN2D	GRIN2D - glutamate receptor, ionotropic, N-methyl D-aspartate 2D	ORPHENADRINE	n/a	TEND
GRIN2D	GRIN2D - glutamate receptor, ionotropic, N-methyl D-aspartate 2D	TENOCYCLIDINE	antagonist	DrugBank
GRIN2D	GRIN2D - glutamate receptor, ionotropic, N-methyl D-aspartate 2D	MEPERIDINE	antagonist	DrugBank
GRIN2D	GRIN2D - glutamate receptor, ionotropic, N-methyl D-aspartate 2D	L-GLUTAMIC ACID	n/a	DrugBank
GRIN2D	GRIN2D - glutamate receptor, ionotropic, N-methyl D-aspartate 2D	ORPHENADRINE	antagonist	DrugBank
GRK5	GRK5 - G protein-coupled receptor kinase 5	BETA BLOCKING AGENTS	n/a	PharmGKB
GRK5	GRK5 - G protein-coupled receptor kinase 5	TRANDOLAPRIL	n/a	PharmGKB
GRK5	GRK5 - G protein-coupled receptor kinase 5	VERAPAMIL	n/a	PharmGKB
GRK5	GRK5 - G protein-coupled receptor kinase 5	ATENOLOL	n/a	PharmGKB
GRK5	GRK5 - G protein-coupled receptor kinase 5	HYDROCHLOROTHIAZIDE	n/a	PharmGKB
GRM5	GRM5 - glutamate receptor, metabotropic 5	ACAMPROSATE	antagonist	DrugBank

Supplemental Table 9. DT Network of primary screen target genes

GRM5	GRM5 - glutamate receptor, metabotropic 5	LY467711	antagonist	TTD
GRM5	GRM5 - glutamate receptor, metabotropic 5	ADX10059	modulator	TTD
GRM5	GRM5 - glutamate receptor, metabotropic 5	AZD2516	antagonist	TTD
GRM5	GRM5 - glutamate receptor, metabotropic 5	ADX-48621	modulator	TTD
GRM5	GRM5 - glutamate receptor, metabotropic 5	MGLURIII AGONIST AND MGLU5 ANTAGONIST	antagonist	TTD
GRM5	GRM5 - glutamate receptor, metabotropic 5	LY525327	antagonist	TTD
GRM5	GRM5 - glutamate receptor, metabotropic 5	AZD2066	antagonist	TTD
GRM5	GRM5 - glutamate receptor, metabotropic 5	MCN3377	modulator	TTD
GSTA5	GSTA5 - glutathione S-transferase alpha 5	GLUTATHIONE	n/a	DrugBank
GSTK1	GSTK1 - glutathione S-transferase kappa 1	GLUTATHIONE	n/a	DrugBank
GSTK1	GSTK1 - glutathione S-transferase kappa 1	GLUTATHIONE SULFINATE	n/a	DrugBank
GSTM1	GSTM1 - glutathione S-transferase mu 1	ZINC TRIHYDROXIDE	n/a	DrugBank
GSTM1	GSTM1 - glutathione S-transferase mu 1	FLUOROTRYPTOPHANE	n/a	DrugBank
GSTM1	GSTM1 - glutathione S-transferase mu 1	(9S,10S)-9-(S-GLUTATHIONYL)-10-HYDROXY-9,10-DIH...	n/a	DrugBank
GSTM1	GSTM1 - glutathione S-transferase mu 1	(9R,10R)-9-(S-GLUTATHIONYL)-10-HYDROXY-9,10-DIH...	n/a	DrugBank
GSTM1	GSTM1 - glutathione S-transferase mu 1	GLUTATHIONE S-(2,4 DINITROBENZENE)	n/a	DrugBank
GSTM1	GSTM1 - glutathione S-transferase mu 1	GLUTATHIONE	n/a	DrugBank
HAGH	HAGH - hydroxyacylglutathione hydrolase	S-(N-HYDROXY-N-BROMOPHENYLCARBAMOYL)GLUTATHIONE	n/a	DrugBank
HAGH	HAGH - hydroxyacylglutathione hydrolase	GLUTATHIONE	n/a	DrugBank
HLA-DQA1	HLA-DQA1 - major histocompatibility complex, class II, DQ alpha 1	INTERFERON BETA-1B	n/a	PharmGKB
HLA-DQA1	HLA-DQA1 - major histocompatibility complex, class II, DQ alpha 1	INTERFERON BETA-1A	n/a	PharmGKB
HNF4G	HNF4G - hepatocyte nuclear factor 4, gamma	PALMITIC ACID	n/a	DrugBank
HNMT	HNMT - histamine N-methyltransferase	S-ADENOSYL-L-HOMOCYSTEINE	n/a	DrugBank
HNMT	HNMT - histamine N-methyltransferase	AMODIAQUINE	inhibitor	DrugBank
HNMT	HNMT - histamine N-methyltransferase	4-(DIMETHYLAMINO)BUTYL IMIDOTHIOCARBAMATE	n/a	DrugBank
HNMT	HNMT - histamine N-methyltransferase	METOPRINE, METHODICHLOROPHEN	n/a	DrugBank
HNMT	HNMT - histamine N-methyltransferase	AMODIAQUINE	inhibitor	TTD
HNMT	HNMT - histamine N-methyltransferase	ANTHRACYCLINES AND RELATED SUBSTANCES	n/a	PharmGKB
HSD11B1	HSD11B1 - hydroxysteroid (11-beta) dehydrogenase 1	2-(2-CHLORO-4-FLUOROPHENOXY)-2-METHYL-N-[(1R,2S...	n/a	DrugBank
HSD11B1	HSD11B1 - hydroxysteroid (11-beta) dehydrogenase 1	(3,3-DIMETHYLPIPERIDIN-1-YL)(6-(3-FLUORO-4-METH...	n/a	DrugBank
HSD11B1	HSD11B1 - hydroxysteroid (11-beta) dehydrogenase 1	(5S)-2-(CYCLOOCTYLAMINO)-5-METHYL-5-PROPYL-1,3-...	n/a	DrugBank
HSD11B1	HSD11B1 - hydroxysteroid (11-beta) dehydrogenase 1	(5S)-2-{[(1S)-1-(2-FLUOROPHENYL)ETHYL]AMINO}-5-...	n/a	DrugBank
HSD11B1	HSD11B1 - hydroxysteroid (11-beta) dehydrogenase 1	(5R)-2-[(2-FLUOROPHENYL)AMINO]-5-(1-METHYLETHYL...	n/a	DrugBank
HSD11B1	HSD11B1 - hydroxysteroid (11-beta) dehydrogenase 1	(11-BETA)-11,21-DIHYDROXY-PREGN-4-ENE-3,20-DIONE	n/a	DrugBank
HSD11B1	HSD11B1 - hydroxysteroid (11-beta) dehydrogenase 1	(5S)-2-{[(1S)-1-(4-FLUOROPHENYL)ETHYL]AMINO}-5-...	n/a	DrugBank
HSD11B1	HSD11B1 - hydroxysteroid (11-beta) dehydrogenase 1	2-(6-{[(3-CHLORO-2-METHYLPHENYL)SULFONYL]AMINO}...	n/a	DrugBank
HSD11B1	HSD11B1 - hydroxysteroid (11-beta) dehydrogenase 1	(2R)-1-[(4-TERT-BUTYLPHENYL)SULFONYL]-2-METHYL-...	n/a	DrugBank
HSD11B1	HSD11B1 - hydroxysteroid (11-beta) dehydrogenase 1	2'-MONOPHOSPHOADENOSINE 5'-DIPHOSPHORIBOSE	n/a	DrugBank
HSD11B1	HSD11B1 - hydroxysteroid (11-beta) dehydrogenase 1	NADH	n/a	DrugBank
HSD11B1	HSD11B1 - hydroxysteroid (11-beta) dehydrogenase 1	N-CYCLOPROPYL-N-(TRANS-4-PYRIDIN-3-YLCYCLOHEXYL...	n/a	DrugBank
HSD11B1	HSD11B1 - hydroxysteroid (11-beta) dehydrogenase 1	(1S,3R,4S,5S,7S)-4-{[2-(4-METHOXYPHENOXY)-2-MET...	n/a	DrugBank

Supplemental Table 9. DT Network of primary screen target genes

HSD11B1	HSD11B1 - hydroxysteroid (11-beta) dehydrogenase 1	1-{[(3R)-3-METHYL-4-({4-[(1S)-2,2,2-TRIFLUORO-1...	n/a	DrugBank
HSD11B1	HSD11B1 - hydroxysteroid (11-beta) dehydrogenase 1	2-(N-MORPHOLINO)-ETHANESULFONIC ACID	n/a	DrugBank
HSD11B1	HSD11B1 - hydroxysteroid (11-beta) dehydrogenase 1	N-{1-[(1-CARBAMOYLCYCLOPROPYL)METHYL]PIPERIDIN-...	n/a	DrugBank
HSD11B1	HSD11B1 - hydroxysteroid (11-beta) dehydrogenase 1	CARBENOXOLONE	inhibitor	DrugBank
HSD11B1	HSD11B1 - hydroxysteroid (11-beta) dehydrogenase 1	AZD8329	inhibitor	TTD
HSD11B1	HSD11B1 - hydroxysteroid (11-beta) dehydrogenase 1	INCB13739	inhibitor	TTD
HSD11B1	HSD11B1 - hydroxysteroid (11-beta) dehydrogenase 1	AZD4017	inhibitor	TTD
HSD17B3	HSD17B3 - hydroxysteroid (17-beta) dehydrogenase 3	NADH	n/a	DrugBank
HTR1A	HTR1A - 5-hydroxytryptamine (serotonin) receptor 1A, G protein-coupled	METHYSERGIDE	n/a	TEND
HTR1A	HTR1A - 5-hydroxytryptamine (serotonin) receptor 1A, G protein-coupled	ROTIGOTINE	n/a	TEND
HTR1A	HTR1A - 5-hydroxytryptamine (serotonin) receptor 1A, G protein-coupled	MIRTAZAPINE	n/a	TEND
HTR1A	HTR1A - 5-hydroxytryptamine (serotonin) receptor 1A, G protein-coupled	ARIPIPRAZOLE	n/a	TEND
HTR1A	HTR1A - 5-hydroxytryptamine (serotonin) receptor 1A, G protein-coupled	CLOZAPINE	n/a	TEND
HTR1A	HTR1A - 5-hydroxytryptamine (serotonin) receptor 1A, G protein-coupled	QUETIAPINE	n/a	TEND
HTR1A	HTR1A - 5-hydroxytryptamine (serotonin) receptor 1A, G protein-coupled	BUSPIRONE	n/a	TEND
HTR1A	HTR1A - 5-hydroxytryptamine (serotonin) receptor 1A, G protein-coupled	ZIPRASIDONE	n/a	TEND
HTR1A	HTR1A - 5-hydroxytryptamine (serotonin) receptor 1A, G protein-coupled	LISURIDE	n/a	TEND
HTR1A	HTR1A - 5-hydroxytryptamine (serotonin) receptor 1A, G protein-coupled	TRAZODONE	n/a	TEND
HTR1A	HTR1A - 5-hydroxytryptamine (serotonin) receptor 1A, G protein-coupled	DOXEPIN	antagonist	DrugBank
HTR1A	HTR1A - 5-hydroxytryptamine (serotonin) receptor 1A, G protein-coupled	PINDOLOL	antagonist	DrugBank
HTR1A	HTR1A - 5-hydroxytryptamine (serotonin) receptor 1A, G protein-coupled	CLOZAPINE	antagonist	DrugBank
HTR1A	HTR1A - 5-hydroxytryptamine (serotonin) receptor 1A, G protein-coupled	OLANZAPINE	antagonist	DrugBank
HTR1A	HTR1A - 5-hydroxytryptamine (serotonin) receptor 1A, G protein-coupled	PROPRANOLOL	other/unknown	DrugBank
HTR1A	HTR1A - 5-hydroxytryptamine (serotonin) receptor 1A, G protein-coupled	ELETRIPTAN	agonist	DrugBank
HTR1A	HTR1A - 5-hydroxytryptamine (serotonin) receptor 1A, G protein-coupled	SUMATRIPTAN	agonist	DrugBank
HTR1A	HTR1A - 5-hydroxytryptamine (serotonin) receptor 1A, G protein-coupled	MOLINDONE	antagonist	DrugBank
HTR1A	HTR1A - 5-hydroxytryptamine (serotonin) receptor 1A, G protein-coupled	THIOPROPERAZINE	antagonist	DrugBank
HTR1A	HTR1A - 5-hydroxytryptamine (serotonin) receptor 1A, G protein-coupled	CINITAPRIDE	agonist	DrugBank
HTR1A	HTR1A - 5-hydroxytryptamine (serotonin) receptor 1A, G protein-coupled	ACEPROMAZINE	antagonist	DrugBank
HTR1A	HTR1A - 5-hydroxytryptamine (serotonin) receptor 1A, G protein-coupled	LISURIDE	agonist	DrugBank
HTR1A	HTR1A - 5-hydroxytryptamine (serotonin) receptor 1A, G protein-coupled	NORTRIPTYLINE	antagonist	DrugBank
HTR1A	HTR1A - 5-hydroxytryptamine (serotonin) receptor 1A, G protein-coupled	CABERGOLINE	agonist	DrugBank
HTR1A	HTR1A - 5-hydroxytryptamine (serotonin) receptor 1A, G protein-coupled	BUSPIRONE	partial agonist	DrugBank
HTR1A	HTR1A - 5-hydroxytryptamine (serotonin) receptor 1A, G protein-coupled	CHLORPROMAZINE	antagonist	DrugBank
HTR1A	HTR1A - 5-hydroxytryptamine (serotonin) receptor 1A, G protein-coupled	ZOLMITRIPTAN	agonist	DrugBank
HTR1A	HTR1A - 5-hydroxytryptamine (serotonin) receptor 1A, G protein-coupled	ROTIGOTINE	n/a	DrugBank
HTR1A	HTR1A - 5-hydroxytryptamine (serotonin) receptor 1A, G protein-coupled	AMITRIPTYLINE	other/unknown	DrugBank
HTR1A	HTR1A - 5-hydroxytryptamine (serotonin) receptor 1A, G protein-coupled	NEFAZODONE	antagonist	DrugBank
HTR1A	HTR1A - 5-hydroxytryptamine (serotonin) receptor 1A, G protein-coupled	ALPRENOLOL	antagonist	DrugBank

Supplemental Table 9. DT Network of primary screen target genes

HTR1A	HTR1A - 5-hydroxytryptamine (serotonin) receptor 1A, G protein-coupled	APOMORPHINE	agonist	DrugBank
HTR1A	HTR1A - 5-hydroxytryptamine (serotonin) receptor 1A, G protein-coupled	PERGOLIDE	agonist	DrugBank
HTR1A	HTR1A - 5-hydroxytryptamine (serotonin) receptor 1A, G protein-coupled	BOPINDOLOL	n/a	DrugBank
HTR1A	HTR1A - 5-hydroxytryptamine (serotonin) receptor 1A, G protein-coupled	TRAZODONE	partial agonist	DrugBank
HTR1A	HTR1A - 5-hydroxytryptamine (serotonin) receptor 1A, G protein-coupled	METHYSERGIDE	agonist	DrugBank
HTR1A	HTR1A - 5-hydroxytryptamine (serotonin) receptor 1A, G protein-coupled	ERGOLOID MESYLATE	other/unknown	DrugBank
HTR1A	HTR1A - 5-hydroxytryptamine (serotonin) receptor 1A, G protein-coupled	PIPOTIAZINE	antagonist	DrugBank
HTR1A	HTR1A - 5-hydroxytryptamine (serotonin) receptor 1A, G protein-coupled	BROMOCRIPTINE	agonist	DrugBank
HTR1A	HTR1A - 5-hydroxytryptamine (serotonin) receptor 1A, G protein-coupled	VILAZODONE	n/a	DrugBank
HTR1A	HTR1A - 5-hydroxytryptamine (serotonin) receptor 1A, G protein-coupled	ROPINIROLE	agonist	DrugBank
HTR1A	HTR1A - 5-hydroxytryptamine (serotonin) receptor 1A, G protein-coupled	PALIPERIDONE	antagonist	DrugBank
HTR1A	HTR1A - 5-hydroxytryptamine (serotonin) receptor 1A, G protein-coupled	ARIPIPRAZOLE	partial agonist/antagonist	DrugBank
HTR1A	HTR1A - 5-hydroxytryptamine (serotonin) receptor 1A, G protein-coupled	QUETIAPINE	antagonist	DrugBank
HTR1A	HTR1A - 5-hydroxytryptamine (serotonin) receptor 1A, G protein-coupled	RISPERIDONE	antagonist	DrugBank
HTR1A	HTR1A - 5-hydroxytryptamine (serotonin) receptor 1A, G protein-coupled	PRAMIPEXOLE	other/unknown	DrugBank
HTR1A	HTR1A - 5-hydroxytryptamine (serotonin) receptor 1A, G protein-coupled	ZIPRASIDONE	antagonist	DrugBank
HTR1A	HTR1A - 5-hydroxytryptamine (serotonin) receptor 1A, G protein-coupled	YOHIMBINE	partial agonist	DrugBank
HTR1A	HTR1A - 5-hydroxytryptamine (serotonin) receptor 1A, G protein-coupled	ONDANSETRON	other/unknown	DrugBank
HTR1A	HTR1A - 5-hydroxytryptamine (serotonin) receptor 1A, G protein-coupled	NARATRIPTAN	agonist	DrugBank
HTR1A	HTR1A - 5-hydroxytryptamine (serotonin) receptor 1A, G protein-coupled	TRIMIPRAMINE	antagonist	DrugBank
HTR1A	HTR1A - 5-hydroxytryptamine (serotonin) receptor 1A, G protein-coupled	ALVERINE	antagonist	DrugBank
HTR1A	HTR1A - 5-hydroxytryptamine (serotonin) receptor 1A, G protein-coupled	PAROXETINE	n/a	PharmGKB
HTR1A	HTR1A - 5-hydroxytryptamine (serotonin) receptor 1A, G protein-coupled	ANTIDEPRESSANTS	n/a	PharmGKB
HTR1A	HTR1A - 5-hydroxytryptamine (serotonin) receptor 1A, G protein-coupled	FLUVOXAMINE	n/a	PharmGKB
HTR1A	HTR1A - 5-hydroxytryptamine (serotonin) receptor 1A, G protein-coupled	SERTRALINE	n/a	PharmGKB
HTR1A	HTR1A - 5-hydroxytryptamine (serotonin) receptor 1A, G protein-coupled	MILNACIPRAN	n/a	PharmGKB
IDH2	IDH2 - isocitrate dehydrogenase 2 (NADP), mitochondrial	ISOCITRIC ACID	n/a	DrugBank
IL1R1	IL1R1 - interleukin 1 receptor, type I	ANAKINRA	n/a	TEND
IL1R1	IL1R1 - interleukin 1 receptor, type I	ANAKINRA	antagonist	DrugBank
IL1R1	IL1R1 - interleukin 1 receptor, type I	ANAKINRA	antagonist	TTD
IL8RB	CXCR2 - chemokine (C-X-C motif) receptor 2	SCH-527123	antagonist	TTD
IL8RB	CXCR2 - chemokine (C-X-C motif) receptor 2	SB-656933	antagonist	TTD
IL8RB	CXCR2 - chemokine (C-X-C motif) receptor 2	SB-265610	antagonist	TTD
IL8RB	CXCR2 - chemokine (C-X-C motif) receptor 2	PS-938285	antagonist	TTD
IL8RB	CXCR2 - chemokine (C-X-C motif) receptor 2	REPARIXIN	antagonist	TTD
IL8RB	CXCR2 - chemokine (C-X-C motif) receptor 2	SB-656933	antagonist	TTD
IL8RB	CXCR2 - chemokine (C-X-C motif) receptor 2	BEVACIZUMAB	n/a	PharmGKB
IL8RB	CXCR2 - chemokine (C-X-C motif) receptor 2	CYCLOPHOSPHAMIDE	n/a	PharmGKB
IL9	IL9 - interleukin 9	MEDI-528	inhibitor	TTD
JAK2	JAK2 - Janus kinase 2	3-{(3R,4R)-4-METHYL-3-[METHYL(7H-PYRROLO[2,3-D]...	n/a	DrugBank
JAK2	JAK2 - Janus kinase 2	4-(3-AMINO-1H-INDAZOL-5-YL)-N-TERT-BUTYLBENZENE...	n/a	DrugBank

Supplemental Table 9. DT Network of primary screen target genes

JAK2	JAK2 - Janus kinase 2	2-(1,1-DIMETHYLETHYL)9-FLUORO-3,6-DIHYDRO-7H-BE...	n/a	DrugBank
JAK2	JAK2 - Janus kinase 2	4-[(2-{4-[(CYCLOPROPYLCARBAMOYL)AMINO]-1H-PYRAZ...	n/a	DrugBank
JAK2	JAK2 - Janus kinase 2	5-PHENYL-1H-INDAZOL-3-AMINE	n/a	DrugBank
JAK2	JAK2 - Janus kinase 2	INCB018424	inhibitor	TTD
JAK2	JAK2 - Janus kinase 2	INCB28050	inhibitor	TTD
JAK2	JAK2 - Janus kinase 2	XL019	inhibitor	TTD
JAK2	JAK2 - Janus kinase 2	ITF2357	inhibitor	TTD
JAK2	JAK2 - Janus kinase 2	TG101348	inhibitor	TTD
JAK2	JAK2 - Janus kinase 2	INCB18424	inhibitor	TTD
JAK2	JAK2 - Janus kinase 2	LY2784544	inhibitor	MyCancerGenome
JAK2	JAK2 - Janus kinase 2	SAR302503	inhibitor	MyCancerGenome
JAK2	JAK2 - Janus kinase 2	RUXOLITINIB	inhibitor	MyCancerGenome
JAK2	JAK2 - Janus kinase 2	AT9283	inhibitor	MyCancerGenome
JAK2	JAK2 - Janus kinase 2	AZD1480	inhibitor	MyCancerGenome
JAK2	JAK2 - Janus kinase 2	AZD1480	inhibitor	TALC
JAK2	JAK2 - Janus kinase 2	AT9283	inhibitor	TALC
JAK3	JAK3 - Janus kinase 3	3-{(3R,4R)-4-METHYL-3-[METHYL(7H-PYRROLO[2,3-D]...	n/a	DrugBank
JAK3	JAK3 - Janus kinase 3	2-(1,1-DIMETHYLETHYL)9-FLUORO-3,6-DIHYDRO-7H-BE...	n/a	DrugBank
JAK3	JAK3 - Janus kinase 3	R348	inhibitor	TTD
JAK3	JAK3 - Janus kinase 3	CP-690,550	inhibitor	TTD
JAK3	JAK3 - Janus kinase 3	TOFACITINIB CITRATE	inhibitor	MyCancerGenome
JAK3	JAK3 - Janus kinase 3	AT9283	inhibitor	TALC
LDHA	LDHA - lactate dehydrogenase A	NICOTINAMIDE	n/a	DrugBank
LDHA	LDHA - lactate dehydrogenase A	OXAMIC ACID	n/a	DrugBank
LDHA	LDHA - lactate dehydrogenase A	ETHENO-NAD	n/a	DrugBank
LDHA	LDHA - lactate dehydrogenase A	NICOTINAMIDE-ADENINE-DINUCLEOTIDE	n/a	DrugBank
LDHA	LDHA - lactate dehydrogenase A	NADH	n/a	DrugBank
LIPF	LIPF - lipase, gastric	ORLISTAT	n/a	TEND
LIPF	LIPF - lipase, gastric	UNDECYL-PHOSPHINIC ACID BUTYL ESTER	n/a	DrugBank
LIPF	LIPF - lipase, gastric	ORLISTAT	inhibitor	DrugBank
LMAN1	LMAN1 - lectin, mannose-binding, 1	ANTIHEMOPHILIC FACTOR	chaperone	DrugBank
MAPK7	MAPK7 - mitogen-activated protein kinase 7	SORAFENIB	n/a	PharmGKB
MCFD2	MCFD2 - multiple coagulation factor deficiency 2	ANTIHEMOPHILIC FACTOR	modulator	DrugBank
MR1	MR1 - major histocompatibility complex, class I-related	ANTITHYMOCYTE GLOBULIN	antagonist	DrugBank
MS4A1	MS4A1 - membrane-spanning 4-domains, subfamily A, member 1	IBRITUMOMAB	n/a	TEND
MS4A1	MS4A1 - membrane-spanning 4-domains, subfamily A, member 1	TOSITUMOMAB	n/a	TEND
MS4A1	MS4A1 - membrane-spanning 4-domains, subfamily A, member 1	RITUXIMAB	n/a	TEND
MS4A1	MS4A1 - membrane-spanning 4-domains, subfamily A, member 1	RITUXIMAB	antibody	DrugBank
MS4A1	MS4A1 - membrane-spanning 4-domains, subfamily A, member 1	IBRITUMOMAB	antibody	DrugBank
MS4A1	MS4A1 - membrane-spanning 4-domains, subfamily A, member 1	TOSITUMOMAB	antibody	DrugBank
MS4A1	MS4A1 - membrane-spanning 4-domains, subfamily A, member 1	RITUXIMAB	antibody	MyCancerGenome
MS4A1	MS4A1 - membrane-spanning 4-domains, subfamily A, member 1	OFATUMUMAB	antibody	MyCancerGenome
MS4A1	MS4A1 - membrane-spanning 4-domains, subfamily A, member 1	IBRITUMOMAB TIUXETAN	antibody	MyCancerGenome
MTR	MTR - 5-methyltetrahydrofolate-homocysteine methyltransferase	HYDROXOCOBALAMIN	cofactor	DrugBank
MTR	MTR - 5-methyltetrahydrofolate-homocysteine methyltransferase	TETRAHYDROFOLIC ACID	cofactor	DrugBank
MTR	MTR - 5-methyltetrahydrofolate-homocysteine methyltransferase	CYANOCOBALAMIN	cofactor	DrugBank
MTR	MTR - 5-methyltetrahydrofolate-homocysteine methyltransferase	L-METHIONINE	product of	DrugBank
MTR	MTR - 5-methyltetrahydrofolate-homocysteine methyltransferase	DACTINOMYCIN	n/a	PharmGKB
MTR	MTR - 5-methyltetrahydrofolate-homocysteine methyltransferase	HYDROXYCHLOROQUINE	n/a	PharmGKB
MTR	MTR - 5-methyltetrahydrofolate-homocysteine methyltransferase	DOXORUBICIN	n/a	PharmGKB
MTR	MTR - 5-methyltetrahydrofolate-homocysteine methyltransferase	VINCRISTINE	n/a	PharmGKB

Supplemental Table 9. DT Network of primary screen target genes

MTR	MTR - 5-methyltetrahydrofolate-homocysteine methyltransferase	SULFASALAZINE	n/a	PharmGKB
MTR	MTR - 5-methyltetrahydrofolate-homocysteine methyltransferase	FOLIC ACID	n/a	PharmGKB
MTR	MTR - 5-methyltetrahydrofolate-homocysteine methyltransferase	CYCLOPHOSPHAMIDE	n/a	PharmGKB
NFKB1	NFKB1 - nuclear factor of kappa light polypeptide gene enhancer in B-cells 1	THALIDOMIDE	n/a	TEND
NFKB1	NFKB1 - nuclear factor of kappa light polypeptide gene enhancer in B-cells 1	PRANLUKAST	other/unknown	DrugBank
NFKB1	NFKB1 - nuclear factor of kappa light polypeptide gene enhancer in B-cells 1	TRIFLUSAL	antagonist	DrugBank
NFKB1	NFKB1 - nuclear factor of kappa light polypeptide gene enhancer in B-cells 1	THALIDOMIDE	antagonist	DrugBank
NFKB1	NFKB1 - nuclear factor of kappa light polypeptide gene enhancer in B-cells 1	BORTEZOMIB	n/a	ClearityFoundationClinicalTrial
NGFR	NGFR - nerve growth factor receptor	SAR164877	antagonist	TTD
NGFR	NGFR - nerve growth factor receptor	PF-4383119	antagonist	TTD
NR1H2	NR1H2 - nuclear receptor subfamily 1, group H, member 2	BENZENESULFONYL	n/a	DrugBank
NR1H2	NR1H2 - nuclear receptor subfamily 1, group H, member 2	N-(2,2,2-TRIFLUOROETHYL)-N-{4-[2,2,2-TRIFLUORO-...	n/a	DrugBank
NR1H2	NR1H2 - nuclear receptor subfamily 1, group H, member 2	1,1,1,3,3,3-HEXAFLUORO-2-{4-[(2,2,2-TRIFLUOROET...	n/a	DrugBank
NR1H2	NR1H2 - nuclear receptor subfamily 1, group H, member 2	(3-{3-[[2-CHLORO-3-(TRIFLUOROMETHYL)BENZYL](2,2...	n/a	DrugBank
NR1I3	NR1I3 - nuclear receptor subfamily 1, group I, member 3	(5BETA)-PREGNANE-3,20-DIONE	n/a	DrugBank
NR1I3	NR1I3 - nuclear receptor subfamily 1, group I, member 3	16,17-ANDROSTENE-3-OL	n/a	DrugBank
NR1I3	NR1I3 - nuclear receptor subfamily 1, group I, member 3	MECLIZINE	modulator	TTD
NR1I3	NR1I3 - nuclear receptor subfamily 1, group I, member 3	EFAVIRENZ	n/a	PharmGKB
NR1I3	NR1I3 - nuclear receptor subfamily 1, group I, member 3	CARBAMAZEPINE	n/a	PharmGKB
NT5E	NT5E - 5'-nucleotidase, ecto (CD73)	PENTOXIFYLLINE	inhibitor	DrugBank
OPRD1	OPRD1 - opioid receptor, delta 1	NALBUPHINE	n/a	TEND
OPRD1	OPRD1 - opioid receptor, delta 1	NALTREXONE	n/a	TEND
OPRD1	OPRD1 - opioid receptor, delta 1	OXYCODONE	n/a	TEND
OPRD1	OPRD1 - opioid receptor, delta 1	BUTORPHANOL	n/a	TEND
OPRD1	OPRD1 - opioid receptor, delta 1	MORPHINE	n/a	TEND
OPRD1	OPRD1 - opioid receptor, delta 1	HYDROCODONE	n/a	TEND
OPRD1	OPRD1 - opioid receptor, delta 1	NALOXONE	n/a	TEND
OPRD1	OPRD1 - opioid receptor, delta 1	FENTANYL	n/a	TEND
OPRD1	OPRD1 - opioid receptor, delta 1	CODEINE	n/a	TEND
OPRD1	OPRD1 - opioid receptor, delta 1	HYDROMORPHONE	n/a	TEND
OPRD1	OPRD1 - opioid receptor, delta 1	PROPOXYPHENE	n/a	TEND
OPRD1	OPRD1 - opioid receptor, delta 1	CODEINE	agonist	DrugBank
OPRD1	OPRD1 - opioid receptor, delta 1	DIPRENORPHINE	antagonist	DrugBank
OPRD1	OPRD1 - opioid receptor, delta 1	BUTORPHANOL	agonist	DrugBank
OPRD1	OPRD1 - opioid receptor, delta 1	LOPERAMIDE	agonist	DrugBank
OPRD1	OPRD1 - opioid receptor, delta 1	HYDROCODONE	agonist	DrugBank
OPRD1	OPRD1 - opioid receptor, delta 1	CARFENTANIL	agonist	DrugBank
OPRD1	OPRD1 - opioid receptor, delta 1	BUPRENORPHINE	antagonist	DrugBank
OPRD1	OPRD1 - opioid receptor, delta 1	PROPOXYPHENE	agonist	DrugBank
OPRD1	OPRD1 - opioid receptor, delta 1	3-METHYLFENTANYL	agonist	DrugBank
OPRD1	OPRD1 - opioid receptor, delta 1	SUFENTANIL	agonist	DrugBank
OPRD1	OPRD1 - opioid receptor, delta 1	OXYCODONE	agonist	DrugBank
OPRD1	OPRD1 - opioid receptor, delta 1	DIMETHYLTHIAMBUTENE	agonist	DrugBank
OPRD1	OPRD1 - opioid receptor, delta 1	HYDROMORPHONE	partial agonist	DrugBank
OPRD1	OPRD1 - opioid receptor, delta 1	NALBUPHINE	antagonist	DrugBank
OPRD1	OPRD1 - opioid receptor, delta 1	NALTREXONE	antagonist	DrugBank
OPRD1	OPRD1 - opioid receptor, delta 1	3-METHYLTHIOFENTANYL	agonist	DrugBank
OPRD1	OPRD1 - opioid receptor, delta 1	REMIFENTANIL	agonist	DrugBank
OPRD1	OPRD1 - opioid receptor, delta 1	DIPHENOXYLATE	agonist	DrugBank
OPRD1	OPRD1 - opioid receptor, delta 1	OXYMORPHONE	antagonist	DrugBank
OPRD1	OPRD1 - opioid receptor, delta 1	NALOXONE	antagonist	DrugBank
OPRD1	OPRD1 - opioid receptor, delta 1	ALVIMOPAN	antagonist	DrugBank
OPRD1	OPRD1 - opioid receptor, delta 1	HEROIN	agonist	DrugBank
OPRD1	OPRD1 - opioid receptor, delta 1	MORPHINE	agonist	DrugBank
OPRD1	OPRD1 - opioid receptor, delta 1	FENTANYL	agonist	DrugBank
OPRD1	OPRD1 - opioid receptor, delta 1	ETORPHINE	agonist	DrugBank
OPRD1	OPRD1 - opioid receptor, delta 1	DIHYDROMORPHINE	agonist	DrugBank
OPRD1	OPRD1 - opioid receptor, delta 1	LEVORPHANOL	agonist	DrugBank
OPRD1	OPRD1 - opioid receptor, delta 1	TRAMADOL	agonist	DrugBank
OPRD1	OPRD1 - opioid receptor, delta 1	AMITRIPTYLINE	agonist	DrugBank
OPRD1	OPRD1 - opioid receptor, delta 1	METHADONE	agonist	DrugBank
OPRD1	OPRD1 - opioid receptor, delta 1	BIO-306	agonist	TTD

Supplemental Table 9. DT Network of primary screen target genes

OPRD1	OPRD1 - opioid receptor, delta 1	BUTORPHANOL	agonist	TTD
OPRD1	OPRD1 - opioid receptor, delta 1	HYDROCODONE	agonist	TTD
OPRD1	OPRD1 - opioid receptor, delta 1	CODEINE	agonist	TTD
OPRD1	OPRD1 - opioid receptor, delta 1	NALBUPHINE	antagonist	TTD
OPRD1	OPRD1 - opioid receptor, delta 1	LOPERAMIDE	binder	TTD
OPRD1	OPRD1 - opioid receptor, delta 1	DIVERS DRUG	agonist	TTD
OPRD1	OPRD1 - opioid receptor, delta 1	HYDROMORPHONE	binder	TTD
OPRD1	OPRD1 - opioid receptor, delta 1	OXYCODONE	agonist	TTD
OPRD1	OPRD1 - opioid receptor, delta 1	NALTREXONE	antagonist	TTD
PARP1	PARP1 - poly (ADP-ribose) polymerase 1	3,4-DIHYDRO-5-METHYL-ISOQUINOLINONE	n/a	DrugBank
PARP1	PARP1 - poly (ADP-ribose) polymerase 1	TRANS-4-(7-CARBAMOYL-1H-BENZIMIDAZOL-2-YL)-1-PR...	n/a	DrugBank
PARP1	PARP1 - poly (ADP-ribose) polymerase 1	(2R)-2-(7-CARBAMOYL-1H-BENZIMIDAZOL-2-YL)-2-MET...	n/a	DrugBank
PARP1	PARP1 - poly (ADP-ribose) polymerase 1	5-FLUORO-1-[4-(4-PHENYL-3,6-DIHYDROPYRIDIN-1(2H...	n/a	DrugBank
PARP1	PARP1 - poly (ADP-ribose) polymerase 1	NU1025	n/a	DrugBank
PARP1	PARP1 - poly (ADP-ribose) polymerase 1	2-{3-[4-(4-FLUOROPHENYL)-3,6-DIHYDRO-1(2H)-PYRI...	n/a	DrugBank
PARP1	PARP1 - poly (ADP-ribose) polymerase 1	NICOTINAMIDE	binder	DrugBank
PARP1	PARP1 - poly (ADP-ribose) polymerase 1	3-METHOXYBENZAMIDE	n/a	DrugBank
PARP1	PARP1 - poly (ADP-ribose) polymerase 1	2-(3'-METHOXYPHENYL) BENZIMIDAZOLE-4-CARBOXAMIDE	n/a	DrugBank
PARP1	PARP1 - poly (ADP-ribose) polymerase 1	6-AMINO-BENZO[DE]ISOQUINOLINE-1,3-DIONE	n/a	DrugBank
PARP1	PARP1 - poly (ADP-ribose) polymerase 1	CARBA-NICOTINAMIDE-ADENINE-DINUCLEOTIDE	n/a	DrugBank
PARP1	PARP1 - poly (ADP-ribose) polymerase 1	2-(4-CHLOROPHENYL)-5-QUINOXALINECARBOXAMIDE	n/a	DrugBank
PARP1	PARP1 - poly (ADP-ribose) polymerase 1	INO-1001	inhibitor	TTD
PARP1	PARP1 - poly (ADP-ribose) polymerase 1	BSI-201	inhibitor	TTD
PARP1	PARP1 - poly (ADP-ribose) polymerase 1	NICOTINAMIDE	binder	TTD
PARP1	PARP1 - poly (ADP-ribose) polymerase 1	KU-0059436	inhibitor	TTD
PARP1	PARP1 - poly (ADP-ribose) polymerase 1	AG140699	inhibitor	TTD
PARP1	PARP1 - poly (ADP-ribose) polymerase 1	MK-4827	inhibitor	TTD
PARP1	PARP1 - poly (ADP-ribose) polymerase 1	OLAPARIB	inhibitor	TTD
PARP1	PARP1 - poly (ADP-ribose) polymerase 1	AG-14699	inhibitor	TTD
PARP1	PARP1 - poly (ADP-ribose) polymerase 1	INIPARIB	other/unknown	MyCancerGenome
PARP1	PARP1 - poly (ADP-ribose) polymerase 1	RUCAPARIB	other/unknown	MyCancerGenome
PARP1	PARP1 - poly (ADP-ribose) polymerase 1	VELIPARIB	other/unknown	MyCancerGenome
PARP1	PARP1 - poly (ADP-ribose) polymerase 1	MK4827	other/unknown	MyCancerGenome
PARP1	PARP1 - poly (ADP-ribose) polymerase 1	OLAPARIB	other/unknown	MyCancerGenome
PARP1	PARP1 - poly (ADP-ribose) polymerase 1	OLAPARIB	n/a	ClearityFoundationClinicalTrial
PARP1	PARP1 - poly (ADP-ribose) polymerase 1	VELIPARIB	n/a	ClearityFoundationClinicalTrial
PARP1	PARP1 - poly (ADP-ribose) polymerase 1	NIRAPARIB	n/a	ClearityFoundationClinicalTrial
PARP1	PARP1 - poly (ADP-ribose) polymerase 1	VELIPARIB	n/a	ClearityFoundationClinicalTrial
PARP1	PARP1 - poly (ADP-ribose) polymerase 1	VELIPARIB	n/a	ClearityFoundationClinicalTrial
PARP1	PARP1 - poly (ADP-ribose) polymerase 1	OLAPARIB	n/a	ClearityFoundationClinicalTrial
PARP1	PARP1 - poly (ADP-ribose) polymerase 1	VELIPARIB	n/a	ClearityFoundationClinicalTrial
PARP1	PARP1 - poly (ADP-ribose) polymerase 1	VELIPARIB	n/a	ClearityFoundationClinicalTrial
PARP1	PARP1 - poly (ADP-ribose) polymerase 1	RUCAPARIB	n/a	ClearityFoundationClinicalTrial
PARP1	PARP1 - poly (ADP-ribose) polymerase 1	VELIPARIB	n/a	ClearityFoundationClinicalTrial

Supplemental Table 9. DT Network of primary screen target genes

PARP1	PARP1 - poly (ADP-ribose) polymerase 1	VELIPARIB	n/a	ClearityFoundationClinicalTrial
PARP1	PARP1 - poly (ADP-ribose) polymerase 1	OLAPARIB	n/a	ClearityFoundationClinicalTrial
PARP1	PARP1 - poly (ADP-ribose) polymerase 1	VELIPARIB	n/a	ClearityFoundationClinicalTrial
PARP1	PARP1 - poly (ADP-ribose) polymerase 1	VELIPARIB	n/a	ClearityFoundationClinicalTrial
PARP1	PARP1 - poly (ADP-ribose) polymerase 1	N/A	n/a	ClearityFoundationClinicalTrial
PARP1	PARP1 - poly (ADP-ribose) polymerase 1	VELIPARIB	n/a	ClearityFoundationClinicalTrial
PARP1	PARP1 - poly (ADP-ribose) polymerase 1	VELIPARIB	n/a	ClearityFoundationClinicalTrial
PARP1	PARP1 - poly (ADP-ribose) polymerase 1	N/A	n/a	ClearityFoundationClinicalTrial
PARP1	PARP1 - poly (ADP-ribose) polymerase 1	OLAPARIB	n/a	ClearityFoundationClinicalTrial
PARP1	PARP1 - poly (ADP-ribose) polymerase 1	1207456-01-6	n/a	ClearityFoundationClinicalTrial
PARP1	PARP1 - poly (ADP-ribose) polymerase 1	OLAPARIB	inhibitor	TALC
PARP1	PARP1 - poly (ADP-ribose) polymerase 1	AG014699	inhibitor	TALC
PARP1	PARP1 - poly (ADP-ribose) polymerase 1	VELIPARIB	inhibitor	TALC
PARP1	PARP1 - poly (ADP-ribose) polymerase 1	MK4827	inhibitor	TALC
PARP1	PARP1 - poly (ADP-ribose) polymerase 1	INIPARIB	inhibitor	TALC
PARP1	PARP1 - poly (ADP-ribose) polymerase 1	ABT-888	inhibitor	CancerCommons
PC	PC - pyruvate carboxylase	PYRUVIC ACID	n/a	DrugBank
PC	PC - pyruvate carboxylase	BIOTIN	n/a	DrugBank
PC	PC - pyruvate carboxylase	5-(HEXAHYDRO-2-OXO-1H-THIENO[3,4-D]IMIDAZOL-6-Y...	n/a	DrugBank
PCSK2	PCSK2 - proprotein convertase subtilisin/kexin type 2	INSULIN, PORCINE	n/a	DrugBank
PCSK2	PCSK2 - proprotein convertase subtilisin/kexin type 2	INSULIN RECOMBINANT	n/a	DrugBank
PDE4B	PDE4B - phosphodiesterase 4B, cAMP-specific	PAPAVERINE	n/a	TEND
PDE4B	PDE4B - phosphodiesterase 4B, cAMP-specific	AMRINONE	n/a	TEND
PDE4B	PDE4B - phosphodiesterase 4B, cAMP-specific	CAFFEINE	n/a	TEND
PDE4B	PDE4B - phosphodiesterase 4B, cAMP-specific	PENTOXIFYLLINE	n/a	TEND
PDE4B	PDE4B - phosphodiesterase 4B, cAMP-specific	THEOPHYLLINE	n/a	TEND
PDE4B	PDE4B - phosphodiesterase 4B, cAMP-specific	ENPROFYLLINE	n/a	TEND
PDE4B	PDE4B - phosphodiesterase 4B, cAMP-specific	DYPHYLLINE	n/a	TEND
PDE4B	PDE4B - phosphodiesterase 4B, cAMP-specific	CIS-4-CYANO-4-[3-(CYCLOPENTYLOXY)-4-METHOXYPHEN...	n/a	DrugBank
PDE4B	PDE4B - phosphodiesterase 4B, cAMP-specific	(S)-ROLIPRAM	inhibitor	DrugBank
PDE4B	PDE4B - phosphodiesterase 4B, cAMP-specific	8-BROMO-ADENOSINE-5'-MONOPHOSPHATE	n/a	DrugBank
PDE4B	PDE4B - phosphodiesterase 4B, cAMP-specific	DYPHYLLINE	inhibitor	DrugBank
PDE4B	PDE4B - phosphodiesterase 4B, cAMP-specific	AMRINONE	inhibitor	DrugBank
PDE4B	PDE4B - phosphodiesterase 4B, cAMP-specific	S,S-(2-HYDROXYETHYL)THIOCYSTEINE	n/a	DrugBank
PDE4B	PDE4B - phosphodiesterase 4B, cAMP-specific	1-ETHYL-N-(PHENYLMETHYL)-4-(TETRAHYDRO-2H-PYRAN...	n/a	DrugBank
PDE4B	PDE4B - phosphodiesterase 4B, cAMP-specific	4-[8-(3-NITROPHENYL)-1,7-NAPHTHYRIDIN-6-YL]BENZ...	n/a	DrugBank
PDE4B	PDE4B - phosphodiesterase 4B, cAMP-specific	ILOPROST	inducer	DrugBank
PDE4B	PDE4B - phosphodiesterase 4B, cAMP-specific	KETOTIFEN	inhibitor	DrugBank
PDE4B	PDE4B - phosphodiesterase 4B, cAMP-specific	THEOPHYLLINE	inhibitor	DrugBank
PDE4B	PDE4B - phosphodiesterase 4B, cAMP-specific	PAPAVERINE	inhibitor	DrugBank

Supplemental Table 9. DT Network of primary screen target genes

PDE4B	PDE4B - phosphodiesterase 4B, cAMP-specific	PICLAMILAST	n/a	DrugBank
PDE4B	PDE4B - phosphodiesterase 4B, cAMP-specific	IBUDILAST	inhibitor	DrugBank
PDE4B	PDE4B - phosphodiesterase 4B, cAMP-specific	4-[3-(CYCLOPENTYLOXY)-4-METHOXYPHENYL]-2-PYRROL...	n/a	DrugBank
PDE4B	PDE4B - phosphodiesterase 4B, cAMP-specific	(R)-MESOPRAM	n/a	DrugBank
PDE4B	PDE4B - phosphodiesterase 4B, cAMP-specific	ADENOSINE MONOPHOSPHATE	product of	DrugBank
PDE4B	PDE4B - phosphodiesterase 4B, cAMP-specific	THEOBROMINE	inhibitor	DrugBank
PDE4B	PDE4B - phosphodiesterase 4B, cAMP-specific	(R)-ROLIPRAM	inhibitor	DrugBank
PDE4B	PDE4B - phosphodiesterase 4B, cAMP-specific	FILAMINAST	n/a	DrugBank
PDE4B	PDE4B - phosphodiesterase 4B, cAMP-specific	ENPROFYLLINE	inhibitor	DrugBank
PDE4B	PDE4B - phosphodiesterase 4B, cAMP-specific	CAFFEINE	inhibitor	DrugBank
PDE4B	PDE4B - phosphodiesterase 4B, cAMP-specific	PENTOXIFYLLINE	inhibitor	DrugBank
PDE4B	PDE4B - phosphodiesterase 4B, cAMP-specific	1-(2-CHLOROPHENYL)-3,5-DIMETHYL-1H-PYRAZOLE-4-C...	n/a	DrugBank
PDE4B	PDE4B - phosphodiesterase 4B, cAMP-specific	3,5-DIMETHYL-1-(3-NITROPHENYL)-1H-PYRAZOLE-4-CA...	n/a	DrugBank
PDXK	PDXK - pyridoxal (pyridoxine, vitamin B6) kinase	ADENOSINE-5'-[BETA, GAMMA-METHYLENE]TRIPHOSPHATE	n/a	DrugBank
PDXK	PDXK - pyridoxal (pyridoxine, vitamin B6) kinase	PYRIDOXAL	n/a	DrugBank
PDXK	PDXK - pyridoxal (pyridoxine, vitamin B6) kinase	O6-(R)-ROSCOVITINE, R-2-(6-BENZYLOXY-9-ISOPROPY...	n/a	DrugBank
PDXK	PDXK - pyridoxal (pyridoxine, vitamin B6) kinase	PYRIDOXINE	ligand	DrugBank
PDXK	PDXK - pyridoxal (pyridoxine, vitamin B6) kinase	ADENOSINE-5'-DIPHOSPHATE	n/a	DrugBank
PDXK	PDXK - pyridoxal (pyridoxine, vitamin B6) kinase	N6-METHYL-(R)-ROSCOVITINE, R-2-[6-(BENZYL-METHY...	n/a	DrugBank
PLA2G2D	PLA2G2D - phospholipase A2, group IID	STEARIC ACID	n/a	DrugBank
PLD1	PLD1 - phospholipase D1, phosphatidylcholine-specific	CHOLINE	product of	DrugBank
PLK1	PLK1 - polo-like kinase 1	1-[5-METHYL-2-(TRIFLUOROMETHYL)FURAN-3-YL]-3-[(...	n/a	DrugBank
PLK1	PLK1 - polo-like kinase 1	3-[3-CHLORO-5-(5-{[(1S)-1-PHENYLETHYL]AMINO}ISO...	n/a	DrugBank
PLK1	PLK1 - polo-like kinase 1	3-[3-(3-METHYL-6-{[(1S)-1-PHENYLETHYL]AMINO}-1H...	n/a	DrugBank
PLK1	PLK1 - polo-like kinase 1	(1S,6BR,9AS,11R,11BR)-9A,11B-DIMETHYL-1-[(METHY...	n/a	DrugBank
PLK1	PLK1 - polo-like kinase 1	4-(4-METHYLPIPERAZIN-1-YL)-N-[5-(2-THIENYLACETY...	n/a	DrugBank
PLK1	PLK1 - polo-like kinase 1	ON 019190.NA	inhibitor	TTD
PLK1	PLK1 - polo-like kinase 1	HMN-214	inhibitor	TTD
PLK1	PLK1 - polo-like kinase 1	GSK461364	inhibitor	TTD
PLK1	PLK1 - polo-like kinase 1	CYC-800	inhibitor	TTD
PLK1	PLK1 - polo-like kinase 1	DAP-81	inhibitor	TTD
PLK1	PLK1 - polo-like kinase 1	GSK461364	inhibitor	TTD
PLK1	PLK1 - polo-like kinase 1	ZK-THIAZOLIDINONE	inhibitor	TTD
PLK1	PLK1 - polo-like kinase 1	BI 2536	inhibitor	TTD
PLK1	PLK1 - polo-like kinase 1	LC-445	inhibitor	TTD
PLK1	PLK1 - polo-like kinase 1	NMS-1	inhibitor	TTD
PLK1	PLK1 - polo-like kinase 1	BI 6727	inhibitor	TTD
PLK1	PLK1 - polo-like kinase 1	VOLASERTIB	inhibitor	MyCancerGenome
PLK1	PLK1 - polo-like kinase 1	NMS-1286937	inhibitor	MyCancerGenome
PLK1	PLK1 - polo-like kinase 1	GSK461364	inhibitor	MyCancerGenome
PLK1	PLK1 - polo-like kinase 1	BI2536	inhibitor	MyCancerGenome
PLK1	PLK1 - polo-like kinase 1	NMS1286937	inhibitor	TALC
PLK1	PLK1 - polo-like kinase 1	BI-2536	inhibitor	TALC
PLK1	PLK1 - polo-like kinase 1	VOLASERTIB	inhibitor	TALC
PLK1	PLK1 - polo-like kinase 1	GSK461364	inhibitor	TALC
POLA2	POLA2 - polymerase (DNA directed), alpha 2, accessory subunit	DACARBAZINE	n/a	TEND
POLA2	POLA2 - polymerase (DNA directed), alpha 2, accessory subunit	DACARBAZINE	other/unknown	DrugBank
PPP1CA	PPP1CA - protein phosphatase 1, catalytic subunit, alpha isozyme	2,6,8-TRIMETHYL-3-AMINO-9-BENZYL-9-METHOXYNONAN...	n/a	DrugBank

Supplemental Table 9. DT Network of primary screen target genes

PPP1R13L	PPP1R13L - protein phosphatase 1, regulatory subunit 13 like	THALIDOMIDE	n/a	PharmGKB
PPP1R13L	PPP1R13L - protein phosphatase 1, regulatory subunit 13 like	CYCLOPHOSPHAMIDE	n/a	PharmGKB
PPP1R13L	PPP1R13L - protein phosphatase 1, regulatory subunit 13 like	PLATINUM COMPOUNDS	n/a	PharmGKB
PPP1R13L	PPP1R13L - protein phosphatase 1, regulatory subunit 13 like	CISPLATIN	n/a	PharmGKB
PPP1R13L	PPP1R13L - protein phosphatase 1, regulatory subunit 13 like	PLATINUM	n/a	PharmGKB
PRKAA1	PRKAA1 - protein kinase, AMP-activated, alpha 1 catalytic subunit	PHENFORMIN	n/a	TEND
PRKAA1	PRKAA1 - protein kinase, AMP-activated, alpha 1 catalytic subunit	ADENOSINE TRIPHOSPHATE	n/a	DrugBank
PRKAA1	PRKAA1 - protein kinase, AMP-activated, alpha 1 catalytic subunit	PHENFORMIN	activator	DrugBank
PRKAA1	PRKAA1 - protein kinase, AMP-activated, alpha 1 catalytic subunit	ADENOSINE MONOPHOSPHATE	activator	DrugBank
PRKAA1	PRKAA1 - protein kinase, AMP-activated, alpha 1 catalytic subunit	METFORMIN HYDROCHLORIDE	n/a	ClearityFoundationClinicalTrial
PRKAA1	PRKAA1 - protein kinase, AMP-activated, alpha 1 catalytic subunit	METFORMIN HYDROCHLORIDE	n/a	ClearityFoundationClinicalTrial
PRKCE	PRKCE - protein kinase C, epsilon	KAI-1455	activator	TTD
PRKCE	PRKCE - protein kinase C, epsilon	SOPHORETIN	inhibitor	MyCancerGenome
PRKCE	PRKCE - protein kinase C, epsilon	ENZASTAURIN	inhibitor	MyCancerGenome
PRKCG	PRKCG - protein kinase C, gamma	MIDOSTAURIN	inhibitor	TTD
PRKCG	PRKCG - protein kinase C, gamma	SOPHORETIN	inhibitor	MyCancerGenome
PRKCG	PRKCG - protein kinase C, gamma	ENZASTAURIN	inhibitor	MyCancerGenome
PSMA5	PSMA5 - proteasome (prosome, macropain) subunit, alpha type, 5	(3AR,6R,6AS)-6-((S)-((S)-CYCLOHEX-2-ENYL)(HYDRO...	n/a	DrugBank
PSMA5	PSMA5 - proteasome (prosome, macropain) subunit, alpha type, 5	N-[(1R)-1-(DIHYDROXYBORYL)-3-METHYLBUTYL]-N-(PY...	n/a	DrugBank
PSMA5	PSMA5 - proteasome (prosome, macropain) subunit, alpha type, 5	CARFILZOMIB	other/unknown	MyCancerGenome
PSMA5	PSMA5 - proteasome (prosome, macropain) subunit, alpha type, 5	BORTEZOMIB	other/unknown	MyCancerGenome
PTHR1	PTH1R - parathyroid hormone 1 receptor	TERIPARATIDE	n/a	TEND
PTHR1	PTH1R - parathyroid hormone 1 receptor	TERIPARATIDE	binder	DrugBank
PTHR1	PTH1R - parathyroid hormone 1 receptor	PREOTACT	activator	DrugBank
PTHR1	PTH1R - parathyroid hormone 1 receptor	TERIPARATIDE	binder	TTD
PTPN22	PTPN22 - protein tyrosine phosphatase, non-receptor type 22 (lymphoid)	METHOTREXATE	n/a	PharmGKB
SCNN1B	SCNN1B - sodium channel, non-voltage-gated 1, beta subunit	TRIAMTERENE	n/a	TEND
SCNN1B	SCNN1B - sodium channel, non-voltage-gated 1, beta subunit	AMILORIDE	n/a	TEND
SCNN1B	SCNN1B - sodium channel, non-voltage-gated 1, beta subunit	AMILORIDE	inhibitor	DrugBank
SCNN1B	SCNN1B - sodium channel, non-voltage-gated 1, beta subunit	TRIAMTERENE	inhibitor	DrugBank
SCNN1B	SCNN1B - sodium channel, non-voltage-gated 1, beta subunit	INSULIN RECOMBINANT	n/a	PharmGKB
SCNN1B	SCNN1B - sodium channel, non-voltage-gated 1, beta subunit	GLIBENCLAMIDE	n/a	PharmGKB
SCNN1B	SCNN1B - sodium channel, non-voltage-gated 1, beta subunit	ALDOSTERONE	n/a	PharmGKB
SCNN1B	SCNN1B - sodium channel, non-voltage-gated 1, beta subunit	INSULINS AND ANALOGUES	n/a	PharmGKB
SCNN1B	SCNN1B - sodium channel, non-voltage-gated 1, beta subunit	FARGLITAZAR	n/a	PharmGKB
SELP	SELP - selectin P (granule membrane protein 140kDa, antigen CD62)	HEPARIN	inhibitor	DrugBank
SELP	SELP - selectin P (granule membrane protein 140kDa, antigen CD62)	DALTEPARIN	inhibitor	DrugBank
SELP	SELP - selectin P (granule membrane protein 140kDa, antigen CD62)	O-SIALIC ACID	n/a	DrugBank
SELP	SELP - selectin P (granule membrane protein 140kDa, antigen CD62)	NADROPARIN	inhibitor	DrugBank
SELP	SELP - selectin P (granule membrane protein 140kDa, antigen CD62)	RPSGL-IG	inhibitor	TTD
SELP	SELP - selectin P (granule membrane protein 140kDa, antigen CD62)	SELG1	antibody	TTD
SLC1A4	SLC1A4 - solute carrier family 1 (glutamate/neutral amino acid transporter), member 4	L-ALANINE	n/a	DrugBank

Supplemental Table 9. DT Network of primary screen target genes

SLC16A3	SLC16A3 - solute carrier family 16, member 3 (monocarboxylic acid transporter 4)	PYRUVIC ACID	n/a	DrugBank
SRD5A2	SRD5A2 - steroid-5-alpha-reductase, alpha polypeptide 2 (3-oxo-5 alpha-steroid delta 4-dehydrogenase alpha 2)	DUTASTERIDE	n/a	TEND
SRD5A2	SRD5A2 - steroid-5-alpha-reductase, alpha polypeptide 2 (3-oxo-5 alpha-steroid delta 4-dehydrogenase alpha 2)	FINASTERIDE	inhibitor	DrugBank
SRD5A2	SRD5A2 - steroid-5-alpha-reductase, alpha polypeptide 2 (3-oxo-5 alpha-steroid delta 4-dehydrogenase alpha 2)	DUTASTERIDE	inhibitor	DrugBank
SRD5A2	SRD5A2 - steroid-5-alpha-reductase, alpha polypeptide 2 (3-oxo-5 alpha-steroid delta 4-dehydrogenase alpha 2)	AZELAIC ACID	inhibitor	DrugBank
SSTR5	SSTR5 - somatostatin receptor 5	LANREOTIDE	n/a	TEND
SSTR5	SSTR5 - somatostatin receptor 5	OCTREOTIDE	n/a	TEND
SSTR5	SSTR5 - somatostatin receptor 5	OCTREOTIDE	n/a	DrugBank
SSTR5	SSTR5 - somatostatin receptor 5	VAPREOTIDE	agonist	DrugBank
SSTR5	SSTR5 - somatostatin receptor 5	PASIREOTIDE	agonist	TTD
TAP1	TAP1 - transporter 1, ATP-binding cassette, sub-family B (MDR/TAP)	ADENOSINE-5'-DIPHOSPHATE	n/a	DrugBank
TLR2	TLR2 - toll-like receptor 2	S-(DIMETHYLARSENIC)CYSTEINE	n/a	DrugBank
TLR2	TLR2 - toll-like receptor 2	OSPA LIPOPROTEIN	other/unknown	DrugBank
TLR8	TLR8 - toll-like receptor 8	IMIQUIMOD	agonist	DrugBank
TLR8	TLR8 - toll-like receptor 8	CPG 52364	antagonist	TTD
TLR8	TLR8 - toll-like receptor 8	RESIQUIMOD	agonist	TTD
TLR8	TLR8 - toll-like receptor 8	SURECN254984	n/a	ClearityFoundationClinicalTrial
TNFRSF1A	TNFRSF1A - tumor necrosis factor receptor superfamily, member 1A	6-[3-(4-MORPHOLINYL)PROPYL]-2-(3-NITROPHENYL)-5...	n/a	DrugBank
TNFRSF8	TNFRSF8 - tumor necrosis factor receptor superfamily, member 8	BRENTUXIMAB VEDOTIN	n/a	PharmGKB
TNFRSF8	TNFRSF8 - tumor necrosis factor receptor superfamily, member 8	BRENTUXIMAB	antibody	MyCancerGenome
TNFRSF11B	TNFRSF11B - tumor necrosis factor receptor superfamily, member 11b	ZOLEDRONATE	n/a	PharmGKB
TNFRSF11B	TNFRSF11B - tumor necrosis factor receptor superfamily, member 11b	PAMIDRONATE	n/a	PharmGKB
TNFSF13B	TNFSF13B - tumor necrosis factor (ligand) superfamily, member 13b	1,4-DIETHYLENE DIOXIDE	n/a	DrugBank
TNFSF13B	TNFSF13B - tumor necrosis factor (ligand) superfamily, member 13b	CITRIC ACID	n/a	DrugBank
TNFSF13B	TNFSF13B - tumor necrosis factor (ligand) superfamily, member 13b	BR3-FC	inhibitor	TTD
TNFSF13B	TNFSF13B - tumor necrosis factor (ligand) superfamily, member 13b	AMG 623	inhibitor	TTD
TNFSF13B	TNFSF13B - tumor necrosis factor (ligand) superfamily, member 13b	LYMPHOSTAT B	inhibitor	TTD
TNFSF13B	TNFSF13B - tumor necrosis factor (ligand) superfamily, member 13b	ATACICEPT	inhibitor	TTD
TNFSF13B	TNFSF13B - tumor necrosis factor (ligand) superfamily, member 13b	BELIMUMAB	antibody	TTD
TNFSF13B	TNFSF13B - tumor necrosis factor (ligand) superfamily, member 13b	BELIMUMAB	antibody	MyCancerGenome
TPH2	TPH2 - tryptophan hydroxylase 2	MIRTAZAPINE	n/a	PharmGKB
TPH2	TPH2 - tryptophan hydroxylase 2	ANTIDEPRESSANTS	n/a	PharmGKB
TPH2	TPH2 - tryptophan hydroxylase 2	VENLAFAXINE	n/a	PharmGKB
TUBA8	TUBA8 - tubulin, alpha 8	EPOTHILONE B	n/a	DrugBank
TUBA8	TUBA8 - tubulin, alpha 8	EPOTHILONE D	n/a	DrugBank
