# Supplementary material for: Functional genetics-directed identification of novel pharmacological inhibitors of FAS- and TNF-dependent apoptosis that protect mice from acute liver failure
Source: Cell Death Dis. 2016 Mar 17;7(3):e2145–. doi: 10.1038/cddis.2016.45 (PMC4823946; doi:10.1038/cddis.2016.45)
Supplement: Supplementary Figures [file cddis201645x2.pdf]

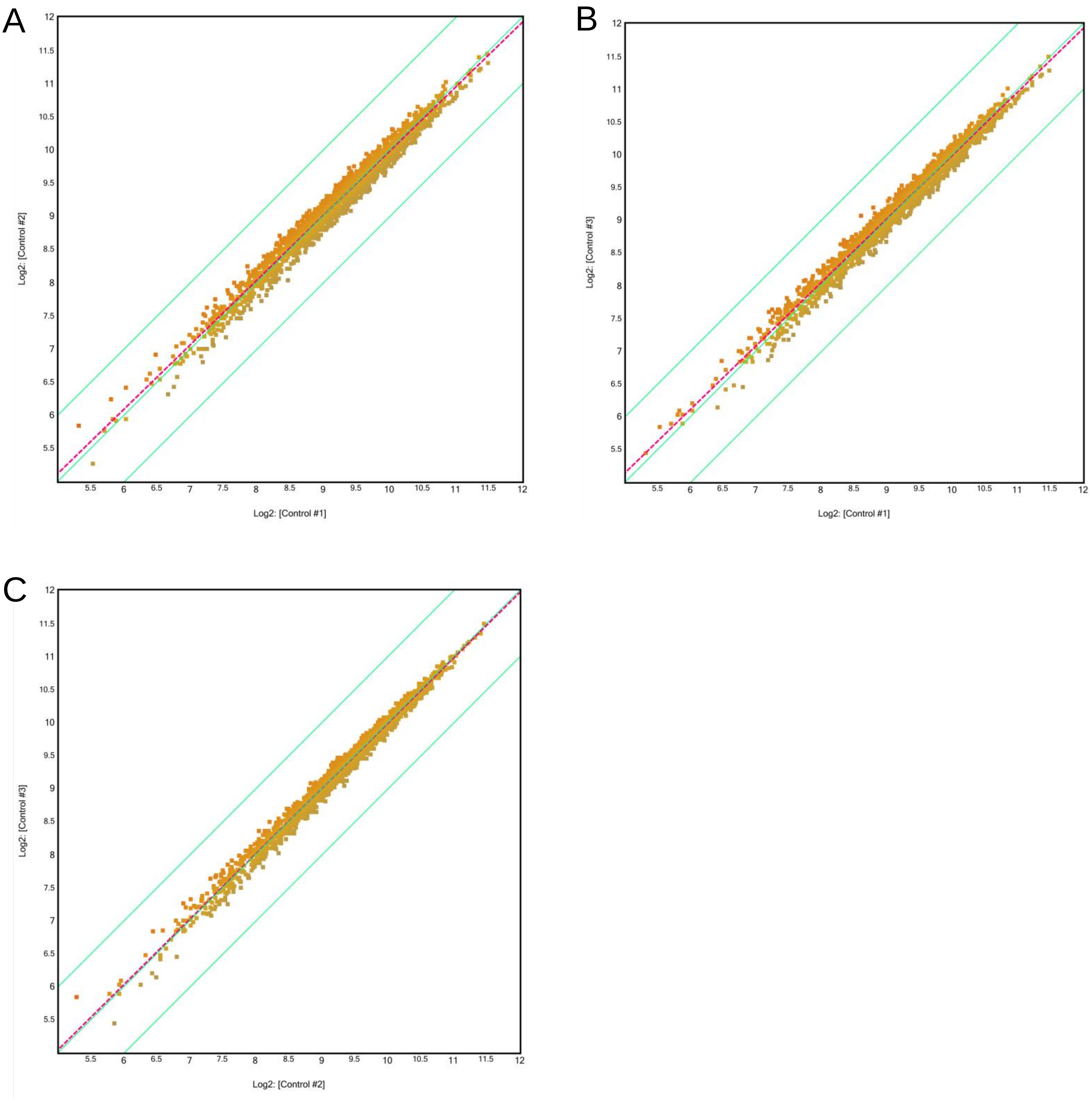

Supplemental Figure 1. (A-C) Correlation of reads per shRNA between replicates.

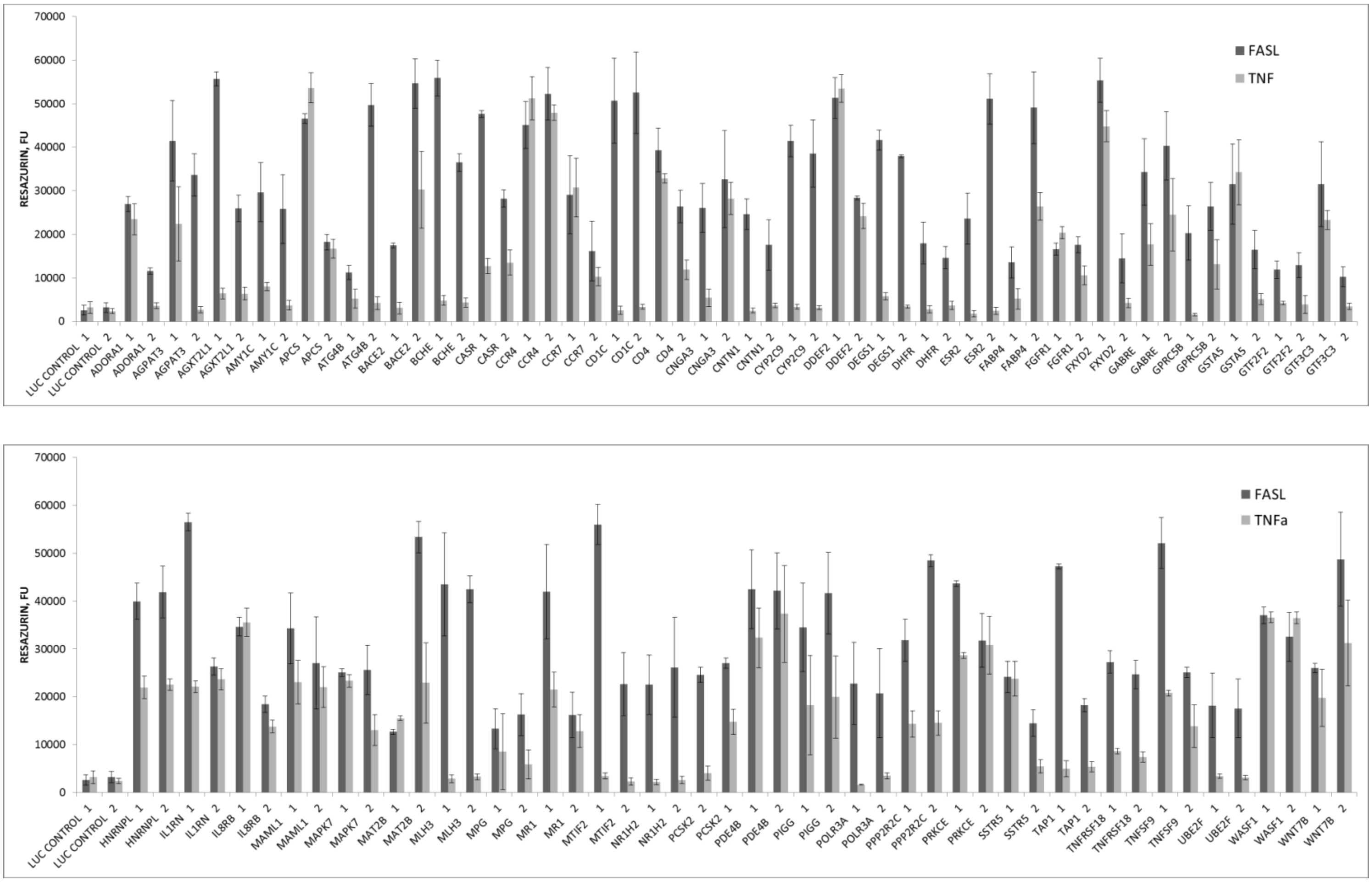

Supplemental Figure 2. Confirmation of individual shRNA hits (data for 104 shRNAs targeting 52 non-canonical FAS/TNF pathway components). Two hundred individual shRNAs targeting non-canonical candidates identified as hits in the primary screen (2 shRNA constructs per gene) were cloned into the original lentiviral expression vector in an arrayed library format and transduced into individual HeLa cell populations. After puromycin selection, transduced cells were exposed to cycloheximide (2.5 µg/ml) + 50 ng/ml FASL (blue bars) or 50 ng/ml TNF (red bars) and cell viability was measured 24 hours later by Resazurin assay, with fluorescence units (FU) quantitatively correlated with cell viability. shRNAs against luciferase (LUC) were used as negative controls. shRNAs were confirmed as hits if they increased survival of FASL- or TNF-treated cells by at least 2-fold relative to the luciferase controls. shRNAs against canonical members of the FAS and TNF pathways isolated in the screen served as positive controls in this assay (see Figure 2). Data for 104 shRNAs targeting 52 novel non-canonical hits that were confirmed in this assay (~50% of the 200 tested) are shown. The bars indicate relative survival of cells (%) vs untreated control cells.

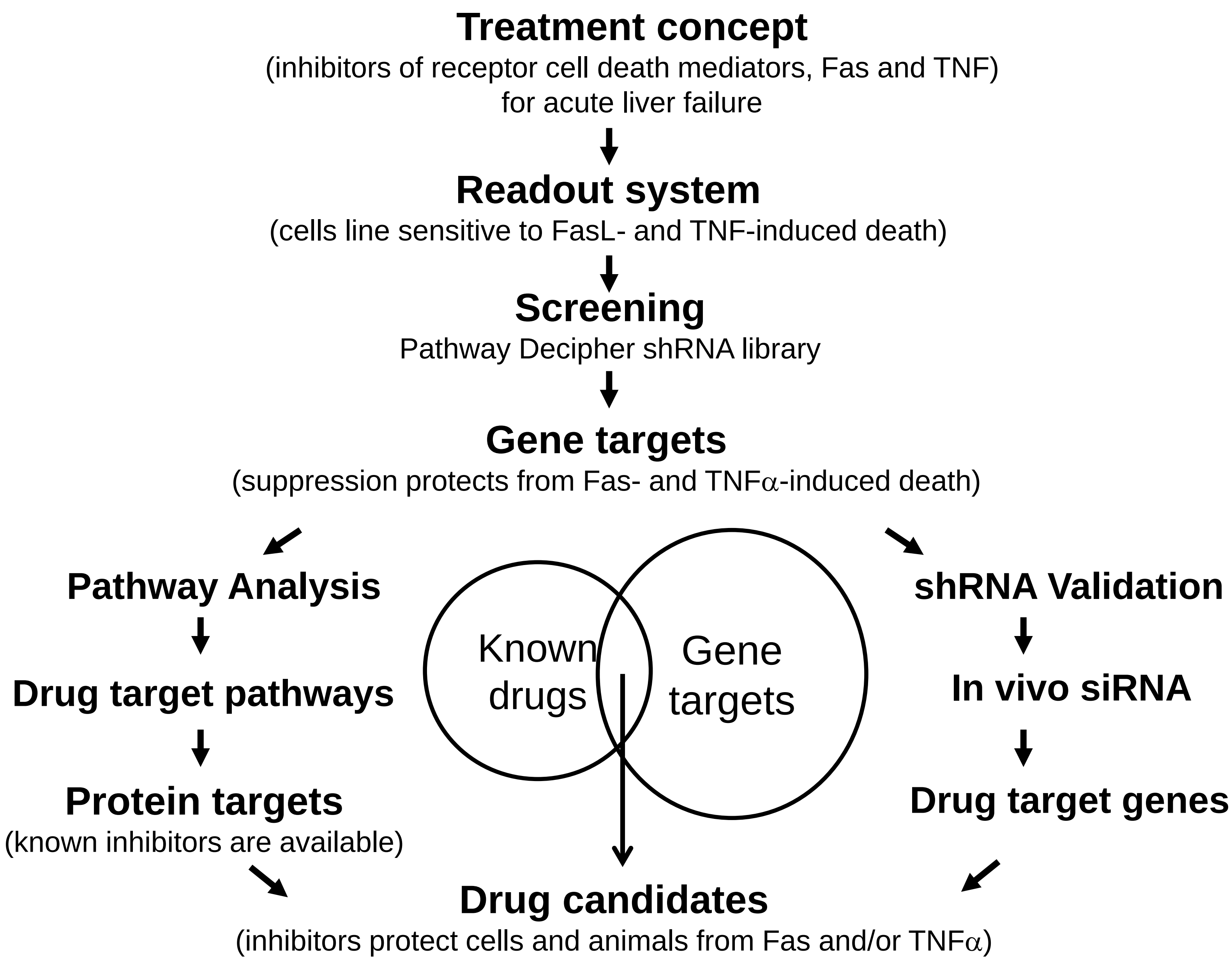

Supplemental Figure 3. The scheme of the workflow for functional genetics-directed identification of novel pharmacological inhibitors of FAS- and TNF-dependent apoptosis that protect mice from acute liver failure.

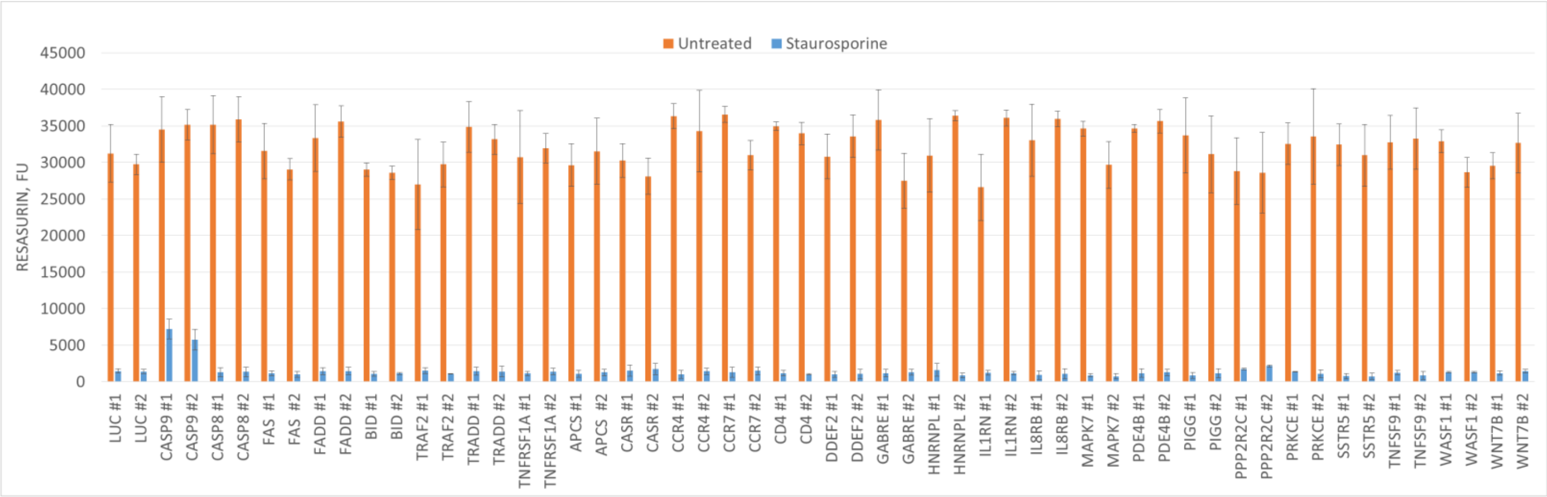

Supplemental Figure 4. Individual shRNA hits confirmed for protection from FAS/TNF extrinsic apoptosis do not protect from intrinsic apoptosis. 16 shRNAs targeting 8 canonical and 40 shRNAs targeting 20 non-canonical FAS/TNF pathway components were tested for their protective effect from intrinsic staurosporine-induced apoptosis. shRNA constructs were transduced into individual HeLa cell populations. After puromycin selection, transduced cells were exposed to staurosporine (0.1 uM) and cell viability was measured 24 hours later by Resazurin assay, with fluorescence units (FU) quantitatively correlated with cell viability. shRNAs against luciferase (LUC) were used as controls, and shRNAs against Caspase 9 served as positive control in this assay.
